# Reducing stillbirths: prevention and management of medical disorders and infections during pregnancy

**DOI:** 10.1186/1471-2393-9-S1-S4

**Published:** 2009-05-07

**Authors:** Esme V Menezes, Mohammad Yawar Yakoob, Tanya Soomro, Rachel A Haws, Gary L Darmstadt, Zulfiqar A Bhutta

**Affiliations:** 1 Division of Maternal and Child Health, The Aga Khan University, Karachi-74800, Pakistan; 2 Department of International Health, Bloomberg School of Public Health, Johns Hopkins University, Baltimore, Maryland, USA

## Abstract

**Background:**

An estimated two-thirds of the world's 3.2 million stillbirths occur antenatally, prior to labour, and are often overlooked in policy and programs. Poorly recognised, untreated or inadequately treated maternal infections such as syphilis and malaria, and maternal conditions including hypertensive disorders, are known risk factors for stillbirth.

**Methods:**

We undertook a systematic review of the evidence for 16 antenatal interventions with the potential to prevent stillbirths. We searched a range of sources including PubMed and the Cochrane Library. For interventions with prior Cochrane reviews, we conducted additional meta-analyses including eligible newer randomised controlled trials following the Cochrane protocol. We focused on interventions deliverable at the community level in low-/middle-income countries, where the burden of stillbirths is greatest.

**Results:**

Few of the studies we included reported stillbirth as an outcome; most that did were underpowered to assess this outcome. While Cochrane reviews or meta-analyses were available for many interventions, few focused on stillbirth or perinatal mortality as outcomes, and evidence was frequently conflicting. Several interventions showed clear evidence of impact on stillbirths, including heparin therapy for certain maternal indications; syphilis screening and treatment; and insecticide-treated bed nets for prevention of malaria. Other interventions, such as management of obstetric intrahepatic cholestasis, maternal anti-helminthic treatment, and intermittent preventive treatment of malaria, showed promising impact on stillbirth rates but require confirmatory studies. Several interventions reduced known risk factors for stillbirth (e.g., anti-hypertensive drugs for chronic hypertension), yet failed to show statistically significant impact on stillbirth or perinatal mortality rates. Periodontal disease emerged as a clear risk factor for stillbirth but no interventions have reduced stillbirth rates.

**Conclusion:**

Evidence for some newly recognised risk factors for stillbirth, including periodontal disease, suggests the need for large, appropriately designed randomised trials to test whether intervention can minimise these risks and prevent stillbirths. Existing evidence strongly supports infection control measures, including syphilis screening and treatment and malaria prophylaxis in endemic areas, for preventing antepartum stillbirths. These interventions should be incorporated into antenatal care programs based on attributable risks and burden of disease.

## Introduction

Of the world's 3.2 million annual stillbirths, at least 98% occur in low-/middle-income countries, and on average, as many as two-thirds of these stillbirths are thought to occur antenatally, prior to labour [1,2]. Proportions of antenatal and intrapartum stillbirths may vary in different low- and middle-income country settings depending on the prevalence of risk factors and quality of antenatal and obstetric care. Antenatal stillbirths typically show signs of maceration, and result from an insult occurring *in utero *[3]. Interventions targeting this period can play a major part in reducing the burden of stillbirths. Many known causes of antenatal stillbirths, including infections and maternal conditions including gestational diabetes and hypertension, are potentially preventable or treatable; relatively simple interventions may reduce their incidence. Known risk factors include syphilis, ascending bacterial vaginal infections, pre-existing and gestational maternal conditions like diabetes, inherited thrombophilias, and intrahepatic cholestasis; pre-eclampsia, placental abruption and other placental dysfunction; and maternal Rhesus disease [4].

Interventions targeting these risk factors may reduce stillbirth rates, especially in low- and middle-income countries. However, the evidence for impact of these interventions on stillbirth and perinatal mortality rates has not been extensively reviewed. This paper systematically examines the evidence for interventions to address or ameliorate the impact of known or biologically plausible clinical risk factors for stillbirth that are treatable or preventable during the antenatal period. Many of the risk factors examined are more prevalent in low- and middle-income countries than in high-income countries, including infections such as syphilis and malaria. Lack of access to health facilities providing antenatal care (ANC) or emergency obstetric care, especially in rural or otherwise remote areas, is associated with poor obstetric outcomes [5,6]. Many of the interventions examined in this review are deliverable via outreach services at the community level, but some require equipped and functional facilities.

## Methods

The methodology of the literature search, along with the data analysis strategy, has been detailed previously [7]. We consulted PubMed and the Cochrane Library, including all human studies published after 1980. We included gray literature wherever possible. General search terms were used like "stillbirth", "fetal death" and "perinatal mortality", along with targeted terms like "antibiotics AND pregnancy" and "periodontal AND pregnancy". Interventions delivered during pregnancy for prevention or treatment of maternal infections or conditions are analyzed in this paper for their effect on stillbirths or perinatal mortality as shown in Table 1.

**Table 1:** Interventions implemented antenatally to prevent or treat maternal infections or conditions reviewed in this paper

**Prevention and management of problems during pregnancy**
Management of chronic and pregnancy-induced hypertension and prevention of pre-eclampsia
• Calcium supplementation to prevent pregnancy-induced hypertension and pre-eclampsia
• Anti-hypertensives for chronic maternal hypertension
Anti-platelet agents
Heparin and other anti-coagulants
Anti-oxidants
Management of intrahepatic cholestasis
Maternal plasma exchange
Cervical cerclage

**Infection control and treatment**
Syphilis screening and treatment
Antibiotics and antisepsis in high-risk pregnancies (BV, asymptomatic bacteriuria, and GBS colonisation)
Antibiotics for preterm premature rupture of membranes
Anti-helminthics during pregnancy
Prophylactic anti-malarials
Insecticide-treated nets during pregnancy
Prevention of mother-to-child transmission of HIV
Periodontal care

A total of 345 papers (35 systematic reviews and 310 individual studies) met the study criteria and were included in this paper.

## Results

### Prevention and management of problems in pregnancy

#### Calcium supplementation to prevent pregnancy-induced hypertension (PIH) and pre-eclampsia

##### Background

High blood pressure with or without proteinuria is a major cause of maternal morbidity and mortality [8], as well as perinatal morbidity and mortality, worldwide. Hypertension has been estimated to complicate 5% of all pregnancies and 11% of first pregnancies, half associated with pre-eclampsia, and accounting for up to 40,000 maternal deaths annually [9].

Pre-term birth, commonly associated with hypertensive disorders, is the leading cause of early neonatal death and infant mortality globally [10]. Inverse relationships between calcium intake and hypertensive disorders of pregnancy were observed among the Mayan Indians in Guatemala [11], whose diet includes corn soaked in lime, and in Ethiopia, where dietary intake of calcium is also high [12]; both populations have low incidence of pre-eclampsia and eclampsia. This evidence, with other epidemiological and clinical studies [13,14], suggested that increased calcium intake during pregnancy might reduce the incidence of hypertensive disorders, including pre-eclampsia, among women with low calcium intake. As supplementation is inexpensive and relatively straightforward, calcium supplementation is attractive as a potential intervention to reduce the risk of pre-eclampsia. The mechanisms involved in calcium-mediated effects on blood pressure reduction are not well understood, but it has been posited that parathyroid hormone might be involved in regulating this relationship [15].

##### Literature-based evidence

Our literature search identified two systematic reviews and one Cochrane protocol, along with one other RCT (Table 2). Trumbo et al. [16] evaluated 7 moderate- to high-quality studies assessing the relationship between calcium intake and pregnancy-induced hypertension and/or pre-eclampsia (Additional file 1). The purpose of this review by the Food and Drug Administration (FDA) was to make recommendations for the US population and thus studies carried out in populations with low calcium intakes and undernutrition were specifically not included. Four studies showed no reduction in the incidence of pregnancy-induced hypertension as a result of calcium supplementation and three showed a reduction. Five of these studies also examined pre-eclampsia as an outcome and three of the five showed no reduction in the incidence of pre-eclampsia from calcium supplementation while the remaining two showed a reduction. Based on this review, the US Food and Drug Administration concluded that the relationship between calcium and risk of hypertension is inconsistent and inconclusive, and an impact of calcium intake on the risk of pregnancy-induced hypertension and pre-eclampsia is unlikely.

**Table 2:** Impact of calcium supplementation for prevention of PIH and pre-eclampsia on stillbirth and perinatal mortality

**Source**	**Location and Type of Trial**	**Intervention**	**Stillbirths and perinatal outcomes**
***Reviews & meta-analyses***			

Hofmeyr et al. 2007 [17,188]	Argentina, USA, Australia, Ecuador, India, Egypt, Peru, South Africa, Vietnam. Meta-analysis (Cochrane) 10 RCTs included, N = 15,103 women.	To assess the effects of calcium supplementation during pregnancy vs. placebo on hypertensive disorders of pregnancy and related maternal and child outcomes.	SB or death before discharge from hospital: RR = 0.89 (95% CI: 0.73–1.09) **[NS]**

Trumbo et al. 2007 [16]	Argentina, USA, Guatemala, Austrália. Review (FDA). 7 RCTs included, N = 6542 women.	To assess the effects of calcium supplementation during pregnancy vs. placebo on hypertensive disorders of pregnancy.	PIH: 3 of 7 RCTs showed reduction in PIH with 2 g/day dose, 4 RCTs showed no impact. Pre-eclampsia: 2 of 5 RCTs showed reduction in pre-eclampsia with 1.8 or 2 g/day dose, 3 RCTs showed no impact.

***Intervention studies***			

Kumar et al. 2009 [19]	India (New Delhi). Lok Nayak Hospital. RCT. N = 524 healthy primigravidas with a blood pressure of less than 140/90 mm Hg between the 12th and 25th weeks of gestation.	Compared the impact of 2 g of elemental calcium (intervention) vs. placebo (controls) from the time of enrollment to delivery.	SBR: 5/251 (2.0%) vs. 6/273 (2.2%) in intervention and control groups, respectively; P = 0.62.

Hofmeyr et al. [17] reviewed 12 studies of good quality, including 5 of the studies in the Trumbo analysis, to assess the impact of calcium supplementation for preventing hypertensive disorders in pregnant women. The review found that calcium supplementation reduced the risk of high blood pressure (11 trials, N = 14,946 women: RR = 0.70, 95% CI: 0.57–0.86) and pre-eclampsia (12 trials, N = 15,206 women: RR = 0.48, 95% CI: 0.33–0.69) compared to placebo. Effect on pre-eclampsia was greatest for high-risk women (5 trials, N = 587 women: RR = 0.22, 95% CI: 0.12–0.42), and those with low baseline calcium intake (7 trials, N = 10,154 women: RR = 0.36, 95% CI: 0.18–0.70) (Additional file 2). Calcium supplementation also reduced the composite outcome "maternal death or serious morbidity" (4 trials, N = 9732 women; RR = 0.80, 95% CI: 0.65–0.97). However, there was no overall effect on the relative risk of a stillbirth or the baby dying before discharge from hospital (10 trials, N = 15,141 women: RR = 0.89, 95% CI: 0.73–1.09) ***[LOE: 1+]***. Another Cochrane review on calcium supplementation other than for preventing or treating hypertension on pregnancy and infant outcomes is currently in progress [18].

In India, an RCT by Kumar et al. [19] found no significant difference in the percentage of stillbirths in healthy primigravidas supplemented with calcium compared to placebo (2.0% vs. 2.2%, respectively).

##### Conclusion

The evidence from a range of reasonable studies and a Cochrane review (Grade B evidence) indicated that calcium supplementation reduced the risk of pre-eclampsia by 31–67% and gestational hypertension by 30% in calcium-deficient individuals, and also had a concomitant benefit to maternal health. There was no significant effect of calcium supplementation in pregnant women on the risk of stillbirth or the baby dying before discharge from hospital, regardless of the mother's risk of hypertensive disorders of pregnancy. The evaluation of the evidence by Trumbo et al. [16] reflects a more guarded view on the role of calcium in prevention of hypertension in low-risk women. Both the Trumbo and the Cochrane review included a large NIH study (N = 4590) [20] in a well-nourished US population that found no evidence of benefit in preventing pre-eclampsia. However, the NIH participants made up a much larger proportion of the sample assessed in the Trumbo review than the Cochrane review, heavily influencing its findings. Reduction in pre-term birth due to calcium supplementation remains plausible, particularly given the reduction in the risk of pre-term birth observed among women at high risk of pre-eclampsia, but this impact remains unproven. The evidence for an impact of calcium supplementation varies based on the nutritional status of the population under study. It is plausible that calcium supplementation is effective in preventing pregnancy-induced hypertension and/or pre-eclampsia only in calcium-deficient individuals; the Cochrane review [17] included a number of studies among undernourished populations and found that the risk of pre-eclampsia was reduced by half. There are currently insufficient trials of calcium supplementation in calcium-deficient populations to assess impact on stillbirths conclusively. Future studies must evaluate the impact of calcium supplementation on a range of perinatal outcomes including stillbirths, focused wherever possible on calcium-deficient populations.

#### Anti-hypertensives for chronic maternal hypertension

##### Background

Alone, high blood pressure has little impact on pregnancy outcomes–nearly 10% of normotensive women experience abnormally elevated blood pressure at some point during pregnancy–but sustained elevated blood pressure may be associated with other complications [21,22]. Between 10 and 15% of maternal deaths in low-/middle-income countries are associated with hypertensive disorders of pregnancy [23-25], and these conditions also increase the risk of perinatal mortality as a consequence of prematurity, poor fetal growth, and other unknown mechanisms [26-28]. There are several major categories of hypertensive disorders, including gestational hypertension or pregnancy-induced hypertension (hypertension without proteinuria); pre-eclampsia (hypertension with proteinuria); chronic or essential hypertension (pre-existing hypertension); and chronic hypertension with superimposed pre-eclampsia [29]. Hypertension, hypothesised to cause fetal distress due to vasoconstriction that reduces the blood supply across the placenta, or placental abruption, may result in poor growth or premature delivery, even when it is mild to moderate. Of all hypertensive disorders of pregnancy, pre-eclampsia/eclampsia has the highest impact on morbidity and mortality, including renal or liver failure, clotting disorders, stroke, pre-term delivery, stillbirth or neonatal death [30].

The pathophysiology of adverse pregnancy outcomes associated with hypertensive disorders is poorly understood. In pre-eclampsia, the prevailing theory is that poor placentation results in an ischemic placenta, which restricts blood and nutrient flow to the fetus. The dysfunctional placenta also releases factors into the mother's bloodstream that damage maternal organs and vasculature, and cause proteinuria [31,32]. There are no reliable ways of predicting which women will develop pre-eclampsia, or which cases of pre-eclampsia will become eclamptic [33]. However, women with severe hypertension prior to pregnancy, women who are hypertensive during the first trimester despite use of antihypertensives, and women who have had a prior adverse pregnancy outcome are known to be at high risk of super-imposed pre-eclampsia (50%–75%), fetal growth restriction (25%–40%), and placental abruption (10%–20%) [34].

The care of women with hypertensive disorders is complex [21]. The rationale for administering anti-hypertensive drugs to pregnant women with high blood pressure is that these drugs could prevent progression of high blood pressure to pre-eclampsia, and pre-eclampsia to eclampsia (seizures), thus reducing the risk of pre-term delivery and placental abruption and improve fetal growth [33]. The optimal treatment of chronic hypertension in pregnancy, including the effectiveness and safety of other anti-hypertensive drugs, such as calcium channel blockers and alpha agonists, which act primarily by causing vasodilatation, remains unclear [29].

##### Literature-based evidence

Our literature search identified 6 Cochrane reviews and 2 other intervention/observational studies (Table 3). Abalos et al. [29] undertook a Cochrane review of all RCTs evaluating any anti-hypertensive drug treatment for mild to moderate hypertension during pregnancy defined using objective criteria. Comparisons were of one or more anti-hypertensive drug(s) with placebo, with no anti-hypertensive drug, or with another anti-hypertensive drug, where treatment was planned to continue for at least 7 days (Additional file 3). Forty-six trials (N = 4282 women) were included. Twenty-eight trials compared an anti-hypertensive drug with placebo/no anti-hypertensive drug (N = 3200 women), and found a 50% reduction in risk of low-/middle-income severe hypertension (19 trials, N = 2409 women; RR = 0.50; 95% CI: 0.41–0.61; risk difference (RD) -0.10 (-0.12 to -0.07); number needed to treat (NNT) to prevent a case of severe hypertension was 10 (range: 8–13) but no evidence was found for a difference in the risk of pre-eclampsia (22 trials, N = 2702 women; RR = 0.97, 95% CI: 0.83–1.13). No significant impact on stillbirths was found (RR = 1.14, 95% CI: 0.60–2.17) ***[LOE: 1++]***. Another Cochrane review by Magee et al. [35] compared the effect on perinatal mortality of beta-blocker vs. placebo/no beta-blocker. Based on 13 trials (N = 1429 women), there was no effect of beta-blocker usage on PMR (RR = 1.01, 95% CI: 0.46–2.22) ***[LOE: 1++] ***(Additional file 4). Similarly, Duley et al. [21] reported a non-significant effect of labetalol vs. hydralazine (RR = 0.50, 95% CI: 0.05–4.94) or similar calcium channel blockers vs. hydralazine (RR = 1.36, 95% CI: 0.42–4.41) on risk of fetal or neonatal deaths in pregnant women ***[LOE: 1++] ***(Additional file 5).

**Table 3:** Impact of anti-hypertensive drugs to treat chronic maternal hypertension on stillbirth and perinatal mortality

**Source**	**Location and Type of Study**	**Intervention**	**Stillbirths/Perinatal Outcomes**
***Reviews and meta-analyses***			

Abalos et al. 2007 [29]	Brazil, Caribbean Islands, Ireland, Israel, Italy, South Africa, Sweden, UK, USA, Sudan, Argentina, Australia, France, India, Venezuela. Meta-analysis (Cochrane). 43 RCTs included.	To assess the effects of anti-hypertensive drug treatments for women with mild to moderate hypertension during pregnancy on pregnancy outcomes.	SBR: RR = 1.14 (95% CI: 0.60, 2.17) **[NS]** PMR: RR = 0.96 (95% CI: 0.60–1.54) **[NS]**

Duley et al. 2006 [21]	UK (Northern Ireland, England), South Africa, USA, Brazil, The Netherlands, Germany, Australia. Meta-analysis (Cochrane). 13 RCTs included.	To compare the impact of different anti-hypertensive drugs for very high blood pressure during pregnancy on pregnancy outcomes.	PMR: RR = 0.50 (95% CI: 0.05–4.94) **[NS] **in labetalol vs. hydralazine groups, respectively. PMR: RR = 1.36 (95% CI: 0.42–4.41) **[NS] **in calcium channel blockers vs. hydralazine groups, respectively.

King et al. 2003 [36]	USA, Spain, France, Israel, The Netherlands, Thailand. Meta-analysis. 10 RCTs RCTs included (N = 810 participants).	To assess the effects on maternal, fetal and neonatal outcomes of calcium channel blockers, administered as a tocolytic agent, to women in pre-term labour.	PMR: RR = 1.65 (95% CI: 0.74–3.64).

Magee et al. 2003 [35]	England, Caribbean Islands, Israel, France, Scotland, Sweden, USA, Argentina, Australia, India, Venezuela. Meta-analysis (Cochrane). 27 RCTs included.	To assess whether oral beta-blockers are better than placebo, or no beta-blocker, and have advantages over other anti-hypertensives, for women with mild to moderate pregnancy hypertension.	PMR: RR = 1.01 (95% CI: 0.46–2.22) **[NS] **in beta-blocker vs. placebo/no beta-blocker groups, respectively.

Meher et al. 2007 [41]	Italy. Meta-analysis. 4 RCTs included.	Compared the impact of nitric oxide vs. placebo/no intervention in treatment of hypertension in pregnancy.	PMR + NMR: RR = 0.25 (95% CI: 0.03–2.34) **[NS]** [0/65 vs. 2/49 in the nitric oxide group vs. the placebo group, respectively.]

Say et al. 1996 [37]	The Netherlands. 1 RCT included (N = 100 participants).	Assessed the effects of calcium channel blockers on fetal growth and neonatal morbidity and mortality in pregnancies where impaired fetal growth was suspected.	PMR: OR = 0.14 (95% CI: 0.00–6.82).

***Intervention studies***			

Hennessy et al. 2007 [40]	Australia, Sydney, tertiary referral maternity hospital. RCT. N = 124 hypertensive women.	Compared the impact of IV hydralazine (5 mg doses) to mini-bolus diazoxide (15 mg doses) on pregnancy outcomes.	PMR: 3 vs. 1 perinatal deaths in hydralazine vs. diazoxide groups, respectively. No statistical significance data given.

***Observational studies***			

Kanner et al. 1980 [189]	Israel, Tel Aviv University Medical School. Prospective cohort study. N = 13 patients with longstanding hypertension during 15 pregnancies.	Measured pregnancy outcomes after administering a combination of propranolol and hydralazine to subjects with essential hypertension in pregnancy.	SB: 1/15 in subjects given propranolol+hydralazine. No controls.

In another Cochrane review comparing the impact of calcium channel blockers to any other tocolytic agent in inhibiting pre-term labour, King et al. 2003 [36] found no statistically significant difference in PMR (10 trials; N = 810 pregnant women, RR = 1.65, 95% CI: 0.74–3.64) ***[LOE: 1+] ***(Additional file 6). Similarly, Say et al. 1996 [37] compared flunarizine vs. no treatment in pregnant women either at high risk or with suspected impaired fetal growth and found a large but non-significant reduction in PMR (OR = 0.14, 95% CI: 0.00–6.82) ***[LOE: 1+] ***(Additional file 7). Other Cochrane reviews of the effect of hypertensive treatments on pregnancy outcome are in progress [38,39].

Among other studies, an RCT in an Australian tertiary referral maternity hospital by Hennessy et al [40] compared different anti-hypertensive drugs in hypertensive women (N = 124) and reported three perinatal deaths in the group given IV hydralazine (5 mg doses) compared to one perinatal death in the group given mini-bolus diazoxide (15 mg doses), but the study was too small for the results to reach statistical significance ***[LOE: 1+]***. Another meta-analysis by Meher et al. [41] assessed the impact of nitric oxide in preventing pre-eclampsia (N=4 RCTs) and computed a non-significant decrease in risk of perinatal and neonatal mortality combined compared to untreated controls (RR = 0.25, 95% CI: 0.03–2.34 [NS]) ***[LOE: 1+] ***(Additional file 8).

##### Conclusion

While optimal pharmacological therapy of chronic maternal hypertension is highly desirable to improve maternal outcomes, uncertainty remains about the most optimal agent and duration of therapy. Among women with mild to moderate hypertension, anti-hypertensives are effective in reducing the risk of severe hypertensive episodes in pregnant women and the evidence from available studies is of reasonable quality (Grade B evidence). However, if reductions in severe hypertension were clinically important, a downstream impact of reduced perinatal mortality, pre-term births and caesarean section could be anticipated, yet there is no evidence of such an effect [29]. One possible explanation for this lack of impact is the possibility of over-treatment with anti-hypertensives. The degree to which placental blood flow is autoregulated is still unknown, and some anti-hypertensives are known to increase the risk of fetal distress arising from reduced uteroplacental, umbilical or fetal blood flow [42,43]. Because of these side effects, particularly when treating severe hypertension, it is possible that modest reductions in blood pressure could be of greater benefit to the fetus than achievement of normal maternal blood pressure using anti-hypertensives.

Women allocated to an anti-hypertensive, especially beta-blockers, were less likely to need another agent but more likely to experience side effects than those allocated placebo or no anti-hypertensive treatment. Between 8 and 13 women need to be treated with an anti-hypertensive drug to prevent an episode of severe hypertension. Whether anti-hypertensive treatment is worthwhile depends on whether there are associated reductions in the consequences of severe hypertension, such as pre-eclampsia, eclampsia, maternal stroke, and pre-term birth or associated outcomes, but the limited data suggest a lack of impact on stillbirths and perinatal mortality, and preclude any firm conclusions about intermediate outcomes such as pre-eclampsia and eclampsia.

The impact of various pharmacological agents on perinatal outcomes and stillbirths is unclear and further studies are needed to define optimal approaches to prevention and treatment of hypertension in pregnant women in appropriate community settings. Despite the wealth of studies and information available on the impact of anti-hypertensive therapy on maternal outcomes, there is no evidence that these drugs reduce the risk of stillbirth.

#### Anti-platelet agents

##### Background

Pre-eclampsia is a common and serious complication of pregnancy that can lead to maternal renal or liver failure, placental abruption, maternal seizures, and fetal complications from placental dysfunction and hypoxia including LBW, SGA, stillbirth, and pre-term delivery [44]. Theorised to arise from a poorly implanted or otherwise abnormal placenta that becomes hypoxic, the factors released by the damaged placenta into the maternal circulation are thought to activate platelets and the maternal clotting system [45-47]. This platelet activity may occur before symptoms of pre-eclampsia develop. Pre-eclampsia has also been characterised by insufficient levels of the vasodilator prostacyclin, and excessive production of thromboxane, a platelet-derived vasoconstrictor which stimulates platelet aggregation [47]. The role of platelets in pre-eclampsia suggests that anti-platelet agents, including aspirin, could prevent or slow development of pre-eclampsia and its associated adverse pregnancy outcomes.

Biochemically, the anti-thrombotic effect of aspirin resluts from its suppression of cyclo-oxygenase. This disruption interferes with the production of thromboxane A2 (TXA2) and inhibits TXA2-dependent platelet aggregation [48]. The reduced thromboxane production is thought to protect against vasoconstriction that can cause ischaemia, fetal growth restriction, and pathologic placental blood clots. Aspirin also carries risks: it crosses the placenta, it inhibits platelet function (thus increasing the risk of maternal and fetal bleeding), and has been linked with an increased risk of vascular disruptions including gastroschisis and possible premature closure of the ductus arteriosus [48]. Given safety concerns, as well as clinical evidence that a daily dose as small as 30 to 50 mg of aspirin results in virtually complete suppression of platelet TXA2 synthesis after 7 to 10 days in normal subjects, low doses of aspirin have been considered sufficient to achieve anti-platelet activity with minimal risk [49]. Indeed, large trials have consistently found low-dose aspirin in pregnancy to be relatively safe. There is no standard definition for "low-dose" aspirin, named in reference to standard aspirin (300 mg), but low-dose regimens typically range from 50–120 mg/day in pregnant patients.

##### Literature-based evidence

Our literature search identified one Cochrane, two other systematic reviews and five other observational/intervention studies on the use of anti-platelet agents to improve pregnancy outcomes (Table 4). Duley et al. [44] reviewed 59 RCTs (N = 37,560 women) comparing the use of any anti-platelet agent (e.g., low-dose aspirin or dipyridamole) with either placebo or no anti-platelet agent in pregnant women at risk of pre-eclampsia (Additional file 9). Anti-platelet agents were associated with a statistically significant 17% reduction in the risk of pre-eclampsia (46 trials, N = 32,891 women, RR = 0.83, 95% CI: 0.77–0.89), with statistically significantly greater risk reduction for high-risk compared to moderate-risk women [risk difference (RD) 5.2% (-7.5 to -2.9%) vs. RD -0.84% (-1.37 to -0.3%), respectively]. Higher doses of anti-platelet agents appeared more effective in preventing pre-eclampsia, as 75 mg/day or less of aspirin (21 trials, N = 26,984 women) reduced risk of pre-eclampsia by 12% (RR = 0.88, 95% CI: 0.81–0.95), compared to reductions of 36% (RR = 0.64, 95% CI: 0.51–0.80) and 70% (RR = 0.30, 95% CI: 0.15–0.60) for trials evaluating more than 75 mg/day of aspirin, and trials combining high doses of aspirin with dipyridamole, respectively. Anti-platelet agents statistically significantly reduced the RR of pre-term birth (8% reduction), PMR (14% reduction), and SGA (10% reduction). However, rates of fetal loss were not statistically different between the two groups (RR = 0.96, 95% CI: 0.78–1.18) ***[LOE: 1++]***.

**Table 4:** Impact of anti-platelet agents on stillbirth and perinatal mortality

**Source**	**Location and Type of Study**	**Intervention**	**Stillbirths/Perinatal Outcomes**
***Reviews and meta-analyses***			

Askie et al. 2007 [50]	USA, Zimbabwe, Italy, Brazil, Australia, Jamaica, Spain, UK, South Africa, China, Barbados, Israel, Japan, France, Belgium, Finland. Meta-analysis (Lancet). 31 RCTs (N = 30 563 women) were included.	To assess the effectiveness and safety of anti-platelet drugs for prevention of pre-eclampsia and its consequences vs. placebo.	SBR+neonatal death before discharge (23 trials): RR = 0.91 (95% CI: 0.81–1.03) **[NS]** [484/15412 vs. 524/15260 in intervention vs. control groups, respectively.]

Duley et al. 2007 [44], Duley et al. 2001 [190]	Australia, Austria, Barbados, Brazil, Finland, France, Israel, Italy, Netherlands, Russia, South Africa, UK, USA, Jamaica, Zimbabwe, China, Spain, India, Belgium. Meta-analysis (Cochrane). 42 RCTs (N = 37,560 women) included.	To assess the effectiveness and safety of anti-platelet agents (intervention group) vs. placebo (controls) for women at risk of developing pre-eclampsia.	Fetal loss (miscarriage+SB): RR = 0.96 (95% CI: 0.78–1.18) **[NS]** [169/9109 vs. 172/8960 in intervention vs. control groups, respectively.] PMR: RR = 0.89 (95% CI: 0.74–1.08) **[NS] **[190/8294 vs. 212/8256 in intervention vs. control groups, respectively.] ***2001 findings (30 RCTs)***: Fetal loss (miscarriage+SB): RR = 0.86 (95% CI: 0.75–0.98).

***Intervention studies***			

Beaufils et al. 1991 [191]	France. RCT. N = 323 women at 15–18 wks amenorrhea at 25 centres with prior history of FGR or placental abruption.	Compared impact of aspirin vs. placebo on birth weight, FGR, placental abruption, and stillbirth.	SBR: 1% vs. 5% in intervention vs. control groups, respectively. Mean birth weight difference: 225 g (95% CI: 129–321 g, P = 0.029) [mean birth weight 2751 (SD = 670) vs. 2526 (SD = 848) g in intervention vs. control groups, respectively.] FGR: 13% (N = 20) vs. 26% (N = 19); P < 0.02). Placental abruption: 5% vs. 8% in intervention vs. control groups, respectively.

Tempfer et al. 2006 [192]	Austria. Prospective case-control study. N = 102 women, N = 50 intervention group, N = 52 controls, all with a history of idiopathic recurrent miscarriage, defined as ≥ 3 consecutive miscarriages < 20 wks gestation without associated anatomic, cytogenetic, hormonal, and infectious pathologies or anti-phospholipid syndrome.	To compare a combination treatment of prednisone (20 mg/d) and progesterone (20 mg/d) for the first 12 weeks of gestation, aspirin (100 mg/d) for 38 weeks of gestation, and folate (5 mg every second day) throughout their pregnancies (intervention group) with no treatment (controls).	Live birth rate: 77% (40/52) vs. 35% (18/52) in intervention vs. control groups, respectively (P = 0.04).

***Observational studies***			

Backos et al. 1999 [193]	UK, tertiary referral clinic. Prospective observational study. N = 150 women with history of recurrent miscarriage associated with persistently positive tests for anti-phospholipid antibodies.	Assessed impact of administration of low dose aspirin and low dose heparin.	Live births: 71% (107/150, 71%). Miscarriage: 27% SBR: 1% NND: 1% Pre-term: 24% (N = 26)

Deligiannidis et al. 2007 [66]	Greece. Prospective study (N = 52 women, N = 29 intervention, N = 23 controls who declined intervention).	Anti-thrombotic therapy (low-dose aspirin and low molecular weight heparin) vs. controls.	Fetal death rate (miscarriage+SB): OR = 0.10 (95% CI: 0.002–0.98, Fisher exact test, 0.04) [1/29 vs. 17/23 in intervention vs. control groups, respectively].

Leduc et al. 2007 [194]	Canada (hospital records). Retrospective cohort study. N = 110 pregnancies (N = 50 intervention, N = 60 controls) among women (N = 43) with ≥ 1 pregnancy complicated by severe early-onset pre-eclampsia, placental abruption, fetal growth restriction (FGR), or fetal death.	Anti-coagulant prophylaxis was administered using dalteparin in 13 pregnancies, ASA with dalteparin in 26, and ASA alone in 11.	SB: No deaths occurred.

Askie et al. [50] in a recent meta-analysis evaluated individual patient data (N = 32,217 women, N = 32,819 offspring) involved in 31 RCTs of pre-eclampsia primary prevention (Additional file 10). Comparing women receiving anti-platelet agents to controls, they confirmed a significant reduction in risk of pre-eclampsia (RR = 0.90, 95% CI: 0.84–0.97), very pre-term delivery (< 34 weeks) (RR = 0.90, 95% CI: 0.83–0.98), and pregnancy with a serious adverse outcome (RR = 0.90, 95% CI: 0.85–0.96). Anti-platelet agents had no significant impact on PMR (RR = 0.91, 95% CI: 0.81–1.03) [NS]), though a slight trend toward reduced risk was observed ***[LOE: 1++]***.

##### Conclusion

The overall evidence of anti-platelet agents for pre-eclampsia is Grade B. Among high-risk women, there is some evidence from the Cochrane meta-analysis by Duley et al. [44] that use of low-dose aspirin reduces risk of PIH, but more research is needed to determine those women for whom aspirin therapy would be effective. There is strong evidence that anti-platelet treatment for high-risk women reduces the risk of pre-eclampsia, though the number needed to treat is high. The optimal choice and dosage of anti-platelet agent is unclear, but dosages of low-dose aspirin appear to be important, and higher doses in the low-dose range may be more efficacious. In the Cochrane meta-analysis, dosages of low-dose aspirin of 75 mg/day or more were associated with greater reductions in risk of pre-eclampsia, pre-term birth, and small-for-gestational age babies than preparations with 75 mg/day or less. The safety of dosages of aspirin exceeding this low-dose range remains unproven and requires further research.

The impact of anti-platelet agents on risk of stillbirths is less convincing, although the analysis shows a slight trend in the direction of benefit. Combination treatment with aspirin and heparin leads to a higher live birth rate among women with recurrent miscarriage and anti-phospholipid antibodies, but the results are statistically non-significant. Given the many small studies with poor allocation concealment and inadequate blinding, there is a need for more well-designed RCTs in varied settings and populations to assess the true effectiveness of aspirin in preventing fetal loss.

#### Heparin and other anti-coagulants in high-risk pregnancies

##### Background

Pregnancy and the puerperium are associated with an increased risk of venous thromboembolism, which may be treated or prevented using anti-coagulants including unfractionated or low molecular weight heparin (LMWH) administered intravenously or subcutaneously. Anti-coagulants may also be used prophylactically for women with a history of deep venous thrombosis, clotting disorders, pulmonary embolism, anti-phospholipid antibodies, or systemic lupus erythematosis, or to preserve pregnancy in women with a history of unexplained spontaneous pregnancy losses. Thrombosis of placental vessels associated with many of these conditions can cause placental insufficiency and consequent fetal death [51].

A chief concern regarding anti-coagulant therapy in pregnancy is that warfarin, the anti-coagulant of choice for many chronic conditions, is known to be teratogenic. Historically, many pregnant women receiving heparin treatment have also had underlying conditions associated with stillbirth and adverse fetal outcomes, making clear data scarce on the impact of heparin (whether unfractionated or LMWH) in pregnancy, as well as the efficacy of heparin and other anti-coagulants in preventing pregnancy loss in women with coagulation disorders or a history of pregnancy loss.

##### Literature-based evidence

Our literature search identified three Cochrane reviews, one Cochrane protocol, and 19 other intervention/observational studies (Tables 5, 6). Most studies tested the efficacy of any form of heparin to other anti-coagulants for indications including anti-phospholipid antibodies with prior loss, thrombophilias, unexplained repeated pregnancy loss, and cardiac indications.

**Table 5:** Systematic reviews/meta-analyses and intervention studies of the impact of heparin and other anti-coagulants in pregnancy on stillbirth and perinatal mortality

**Source**	**Location and Type of Trial**	**Intervention**	**Stillbirths/Perinatal Outcomes**
***Reviews and meta-analyses***			

**Anti-phospholipid antibodies**			

Empson et al. 2005 [51]	UK, USA, Italy, New Zealand, Finland. Meta-analysis (Cochrane). Aspirin: 13 RCTs (N = 849 women) included. Heparin: 8 RCTs included (Women with prior miscarriage and anti-phospholipid antibody-positive). N = 98 in trial of LMWH; N = 140 in trial of unfractionated heparin.	To assess the impact on pregnancy loss of: 1. LMWH plus aspirin (intervention) vs. aspirin alone (controls). 2. Unfractionated heparin plus aspirin (intervention) vs. aspirin (controls). 3. Aspirin (intervention) vs. placebo or standard care (control)	1. Pregnancy loss: RR = 0.78 (95% CI: 0.39–1.57) **[NS]** [11/51 vs. 13/47 in intervention vs. control groups, respectively]. 2. Pregnancy loss: RR = 0.46 (95% CI: 0.29–0.71). [18/70 vs. 40/70 in intervention vs. control groups, respectively]. 3. Fetal loss (miscarriage+SB): RR = 1.05 (95% CI: 0.66–1.68) **[NS] **in intervention vs. control groups, respectively.

**Thrombophilias**			

Di Nisio et al. 2005 [60]	Finland, France. Meta-analysis (Cochrane). 1 quasi-RCT included (N = 20 women). 2 RCTs and quasi-RCTs (N = 74 women) included.	To evaluate the efficacy and safety of anti-coagulant agents, such as aspirin compared to placebo and enoxaparin vs. aspirin, in women with a history of ≥ 2 spontaneous miscarriages or one later intrauterine fetal death without apparent causes other than inherited thrombophilias. 1. Assessed the impact of aspirin vs. no treatment on live birth rate. 2. Assessed the effects on live birth rate of subcutaneous enoxaparin (40 mg/daily) vs. aspirin (100 mg/daily) from the 8^th ^week of amenorrhoea after positive pregnancy test.	1. Live-birth rate: RR = 1.00 (95% CI: 0.78–1.29) ** [NS] **in intervention (aspirin) vs. control groups (placebo), respectively. 2. Live-birth rate: RR = 10.00 (95% CI: 1.56–64.20). [10/10 vs. 1/10 in enoxaparin vs. aspirin groups, respectively].

Gates et al. 2002 [62]	UK, Finland. Meta-analysis (Cochrane). 3 RCTs included (N = 40 women).	To assess the effects of unfractionated heparin (intervention) vs. no treatment (controls) on the incidence of venous thromboembolic disease.	Fetal death (miscarriage + SB): RR = 1.00 (95% CI: 0.07–14.90) **[NS] ** [1/20 vs. 1/20 in both groups].

***Intervention studies***			

**Anti-phospholipid antibodies**			

Bar et al. 2000 [54]	Israel. High Risk Pregnancy Clinic, tertiary hospital. Case series. Pregnant women (N = 46) with a history of recurrent abortions, intrauterine fetal death or IUGR and severe early-onset pre-eclampsia.	Compared the impact of LMWH (enoxaparin sodium, 40 mg daily) in combination with low-dose aspirin (100 mg daily) in the first trimester (intervention group 1, n = 14) vs. the second trimester (intervention group 2, n = 17) vs. low-dose aspirin alone (controls).	Abortions: 14% vs. 0% vs. 0% in intervention group 1, intervention group 2, and controls, respectively **[NS] **

Glasnovic et al. 2007 [55]	Croatia. Case series with non-pregnant controls. Pregnant women (N = 62) with suspected anti-phospholipid syndrome (N = 36) vs. non-pregnant women (N = 26) with secondary anti-phospholipid syndrome and previous bad reproductive anamnesis.	Studied the impact of treatment with LMWH plus low-dose aspirin during pregnancy.	Fetal deaths: 0 in all groups.

Goel et al. 2006 [52]	India (New Delhi). RCT. Pregnant women (N = 550) with poor obstetric history and raised anti-cardiolipin antibodies IgG.	Compared the impact of a combination of low-dose aspirin (80 mg/day) and 5000 IU of unfractionated heparin subcutaneously every 12 hrs under hospital surveillance (intervention) vs. low-dose aspirin (80 mg/day; controls) on pregnancy outcomes.	Live birth rate: 28/33 (84.8%) vs. 24/39 (61.5%) in intervention vs. control groups, respectively (P < 0.05).

Malinowski et al. 2003 [56]	Poland (Lodz). RCT. Pregnant women (N = 148) suffering from recurrent abortion with presence of lupus anti-coagulant antibodies and/or high moderate concentration of anti-cardiolipin antibodies.	Compared the impact of low-dose aspirin + LMWH simultaneously (Group 1) vs. LMWH 20 g daily (Group 2) vs. low-dose aspirin 75 mg daily (Group 3).	Live birth (%): 92.5% vs. 81.1% vs. 89.3% in Groups 1, 2 and 3, respectively.

Noble et al. 2005 [57]	USA. Academically based reproductive health centers. Prospective, controlled pilot study. Pregnant women (N = 50) with ≥ 3 pregnancy losses and positive anti-phospholipid antibody.	Compared the impact of LMWH plus low-dose aspirin (Group 1) vs. unfractionated heparin plus low-dose aspirin (Group 2).	Miscarriage: 4/25 (16%) vs. 5/25 (20%) in Group 1 and Group 2, respectively. P = 1.00) **[NS] ** Live births: 21/25 (84%) vs. 20/25 (80%) in Group 1 and Group 2, respectively. (P = 1.00) **[NS] **

Stephenson et al. 2004 [53]	Vancouver. Tertiary referral centre. RCT. Pregnant women (N = 28) with anti-phospholipid syndrome.	Compared the impact of LMWH (dalteparin; intervention) vs. unfractionated heparin (control) preconceptionally or early in pregnancy on live birth rate. All women also received low-dose aspirin, initiated preconceptionally.	Live birth rate: 9/13 (69%) vs. 4/13 (31%) in intervention vs. control groups, respectively.

**Thrombophilias**			

Brenner, LIVE-ENOX Investigators 2005. [195]	Israel. Multicentre. RCT. Pregnant women (N = 180) with thrombophilia and a history of recurrent pregnancy loss.	Compared the impact of enoxaparin 80 mg/day (40 mg 2× daily; intervention) vs. enoxaparin 40 mg/day (40 mg 1× daily; controls).	Live birth rate: 65/83 (78.3%) vs. 70/83 (84.3%) vs. in the intervention vs. comparison groups, respectively.

Dendrinos et al. 2007 [65]	Greece (Athens). RCT. Women (N = 62) with a history of recurrent pregnancy loss and at least one factor of thrombophilic disorder.	Compared the impact of 50 IU/kg of tinzaparin sodium daily (intervention) vs. 100 mg of aspirin daily (controls).	New abortions: 6/31 vs. 11/31 in intervention vs. control groups, respectively; (P = 0.04).

Sarig et al. 2005 [196]	Israel. Non-matched case-control study. Pregnant women (N = 87; N = 47 intervention, N = 40 controls with normal pregnancies) with thrombophilia and recurrent pregnancy loss.	Compared the impact of LMWH (enoxaparin) 40 mg daily (intervention group 1) vs. 40 mg 2× daily (intervention group 2) vs. no treatment (controls).	Live birth: 38/48 (79%) vs. 32/39 (82%) in groups 1 and 2, respectively **[NS] **

**Unexplained prior losses**			

Dolitzky et al. 2006 [70]	Israel. University hospitals, general hospital, and community health clinic. Multi-centre randomised comparative cohort. Pregnant women (N = 107) with ≥ 3 consecutive 1st trimester miscarriages or ≥ 2 consecutive 2nd trimester miscarriages	Compared the impact of LMWH enoxaparin (intervention) vs. aspirin (controls) on the live birth rate.	Live birth rate: RR = 0.92 (95% CI: 0.58–1.46) **[NS] ** [44/54 (81.5%) vs. 42/50 (84%) in intervention vs. control groups, respectively]. Live birth rate in primary aborters: [17/18 (94%) vs. 18/22 (81%) in intervention vs. control groups, respectively].

**Cardiac indications**			

Lee et al. 2007 [68]	Korea (Daegu). Retrospective study. Pregnant women (N = 25) with mechanical heart valve replacement between 1997 and 2005.	Compared the impact of LMWH nadroparin (7,500 U 2× daily) 6–12 wks of gestation and close-to-term only, and coumarin derivatives were used with aspirin at other times (exposed) vs. coumarin derivatives throughout pregnancy (unexposed).	Fetal death (miscarriage + SB): 2/23 (8.7%) vs. 4/8 (50%) in the exposed and unexposed groups, respectively (P = 0.011).

**Table 6:** Observational studies on the impact of heparin and other anti-coagulants in pregnancy on stillbirth and perinatal mortality

**Source**	**Location and Type of Trial**	**Intervention**	**Stillbirths/Perinatal Outcomes**
***Observational studies***			

**Antiphospholipid antibodies**			

Carp et al. 2003 [58]	Israel. Cohort study. Pregnant women (N = 85) with ≥ 3 consecutive pregnancy losses and a hereditary thrombophilia who conceived (N = 85 delivered; N = 38 miscarried).	Compared the impact of enoxaparin 40 mg (intervention) vs. no treatment (controls) on pregnancy outcomes.	Live births: OR = 3.03 (95% CI: 1.12–8.36); P < 0.02. [26/37 (70.2%) vs. 21/48 (43.8%) in intervention vs. control groups, respectively]. (Among primary aborters) Live birth rate: OR = 9.75 (95% CI: 1.59–52.48; P < 0.008). (Among primary aborters ≥ 5 miscarriages) Live birth rate: 61.6% vs. 18.2% in intervention vs. control groups, respectively **[NS]**.

Franklin and Kutteh 2002 [197]	USA. 2 centres. Prospective cohort study. Pregnant women (N = 79) with ≥ 2 consecutive pregnancy losses and anti-phospholipid antibodies (2 intervention groups: group 1 had recurrent pregnancy loss + anti-phospolipid antibodies; group 2 had other positive anti-phospholipid antibodies).	Compared the impact of heparin and aspirin (intervention) vs. aspirin alone (group 3; controls). Intervention group 1 was treated with heparin and aspirin; intervention group 2 was treated with heparin or aspirin; group 3 received aspirin alone.	Viable infants: 19/25 (76%) vs. 18/28 (64%) vs. 12/26 (46%) in groups 1, 2 and 3, respectively (P = 0.03 for group 1 vs. group 3).

Ruffatti et al. 1997 [59]	Italy (Padova). Prospective cohort study. Pregnant women (N = 53) with ≥ 2 consecutive miscarriages during first trimester and/or 1 fetal death during last two trimesters.	Compared the pregnancy success rate with calcium heparin alone, self-administered subcutaneously 3× daily at dosages 15,000–37,500 IU vs. rate prior to therapy.	Live birth: 100% vs. 24.52% in the calcium heparin vs. prior to therapy (P < 0.0001). Malformations: 0/53 30/37 examined placentas (81.08%) showed signs of thrombotic events.

**Thrombophilias**			

Deligiannidis et al. 2007 [66]	Greece. Cohort study. Pregnant women (N = 52) with thrombophilia.	Compared the impact of LMWH plus low-dose aspirin (intervention) vs. no treatment (controls).	Fetal death (miscarriage+SB): OR = 0.10 (95% CI: 0.002–0.98). [1/29 vs. 17/23 in intervention vs. control groups, respectively].

Folkeringa et al. 2007 [63]	Netherlands. Prospective, family cohort study. Pregnant women (N = 376) with (N = 37) and without (N = 18) hereditary deficiencies of antithrombin protein C or protein S.	Compared the impact of thromboprophylaxis with unfractionated or LMWH < 16 wks and > 36 wks of gestation, and a vitamin K antagonist from 16–36 wks and after delivery (intervention #1) vs. no treatment (controls). Additionally compared same treatment in women deficient for antithrombin protein C or protein S (intervention #2) vs. no treatment in non-deficient women (controls).	Fetal death (miscarriage + SB): adj. RR = 0.07 (95% CI: 0.001–0.7, P = 0.02) in intervention #1 group vs. controls, respectively. Fetal death (miscarriage+SB): 0% in deficient women with thromboprophylaxis versus 45% in deficient women without (P = 0.001) and 7% in non-deficient women without thromboprophylaxis (P = 0.37).

**Cardiac indications**			

Kawamata et al. 2007 [69]	Japan. Retrospective study. Women (N = 12; N = 16 pregnancies) with mechanical heart valve replacement.	Assessed the impact of changing warfarin treatment to heparin at 6–13 wks of gestational age; administration continuously adjusted according to the activated partial thromboplastin time level up to the time of delivery.	Fetal death (miscarriage + SB): 1/16 (at 30 wks).

Kim et al. 2007 [67]	South Korea (Seoul). Retrospective study. Women (N = 27; N = 41 pregnancies) with a mechanical valve replacement.	Compared the impact among three groups: group 1 (N = 5) took warfarin throughout the pregnancy, group 2 (N = 18) took heparin throughout the pregnancy, and group 3 (N = 18) took heparin in the 1^st ^trimester and warfarin from 12–20 wks gestation.	SBR: 2/5 (40%) vs. 1/18 (5.6%) vs. 8/18 (44.4%) in groups 1, 2, and 3, respectively.

**Safety**			

Sorensen et al. 2000 [71]	Denmark. Population-based. Retrospective cohort study using national databases. Pregnant women receiving LMWH (N = 66) or no drugs (N = 17,259) between 1991–98.	Compared the impact of LMWH (exposed) vs. no prescriptive drugs (unexposed).	SBR: 0/66 (0%) vs. 204/17,259 (1.2%) in the exposed vs. unexposed groups, respectively. Pre-term: OR = 2.11 (95% CI: 0.96–4.65) **[NS]** LBW and malformations: no increased risk.

Anti-phospholipid antibodies (including lupus coagulant, anti-cardiolipids, etc.)

A Cochrane review by Empson et al. [51] evaluated trials of heparin (N = 13) in pregnant women (N = 849) with a history of pregnancy loss and anti-phospholipid antibodies or lupus coagulant (Additional file 11). Intravenous immunoglobulin in conjunction with heparin (3 trials; N = 58) was associated with higher rates of stillbirth and prematurity compared to unfractionated heparin or LMWH alone (RR = 2.51, 95% CI: 1.27–4.95), similar to prednisone or aspirin (1 trial; N = 82) ***[LOE: 1+]***. For reducing pregnancy loss, unfractionated heparin appeared superior to LMWH when combined with aspirin compared to aspirin alone (RR = 0.46, 95% CI: 0.29–0.71 for unfractionated heparin vs. RR = 0.78, 95% CI: 0.39–1.57 for LMWH). Varying the dosage of heparin had no impact on pregnancy loss (1 trial; N = 50). In India, a more recent RCT [52] also documented higher live birth rates and birth weights with heparin in combination with aspirin versus aspirin alone in a study population with poor obstetric history and elevated anti-cardiolipin antibodies. However, when comparing the relative impact on pregnancy loss (miscarriages + stillbirths) of LMWH versus unfractionated heparin by Stephenson et al. (1 RCT, N = 26 women) [53] in women with anti-phospholipid antibodies or lupus anti-coagulant, while a trend towards reduced pregnancy loss using LMWH was observed (RR = 0.44, 95% CI: 0.18–1.08 [NS]), the results did not reach statistical significance. Several other small studies comparing heparin plus low-dose aspirin to aspirin alone reported no difference in live birth rates or fetal loss between groups [54-57].

Several studies compared heparin treatment to no treatment for anti-phospholipid antibodies. A cohort study in Israel of enoxaparin versus no treatment [58] showed a three-fold higher odds of having a live birth among women given enoxaparin compared to controls (OR = 3.03; 95% CI: 1.12–8.36). Among primary aborters, the odds of live birth were improved even more dramatically with enoxaparin compared to controls. Ruffatti et al [59] assessed the efficacy and safety of calcium heparin versus no treatment to prevent fetal loss in women with a history of pregnancy loss and anti-phospholipid antibodies (N = 53), and reported a 100% live birth rate in patients receiving heparin versus 24.52% live birth rate in patients receiving no treatment (P < 0.001) ***[LOE: 3]***.

Two reviews included analyses of the impact of aspirin versus placebo or no treatment in patients with anti-phospholipid antibodies. Di Nisio et al. [60] reviewed RCTs and quasi-RCTs that assessed the effect of anti-coagulant treatment (either aspirin, unfractionated heparin and low molecular weight heparin compared to placebo or other treatment) on the live-birth rate in women with a history of at least two spontaneous miscarriages or one later intrauterine fetal death without apparent causes other than inherited thrombophilias (Additional file 12). Extracting relevant data from two eligible trials (N = 242), the study found no impact of aspirin vs. placebo on live birth rate (RR = 1.00, 95% CI: 0.78–1.29) ***[LOE: 1+]***. Empson et al. [51] examined outcomes of aspirin given to maintain pregnancy in women with prior miscarriage and anti-phospholipid syndrome (APL) (N = 11 RCTs), and found no statistical difference in impact compared with placebo or usual care (RR = 1.05, 95% CI: 0.66–1.68) ***[LOE: 1+]***.

Thrombophilias

A Cochrane protocol by Dodd et al. [61] indicates that a review evaluating anti-thrombotic therapy for improving maternal or infant health outcomes in women at risk of placental dysfunction is in progress, and another Cochrane review by Gates et al. [62] of heparin as prophylaxis for thromboembolic disease did not identify enough deaths to conduct statistical analysis (Additional file 13). We also reviewed an observational study of anti-thrombotic therapy [63] including antithrombin, protein C or protein S deficient women (N = 37), which compared thromboprophylaxis with either LMWH or unfractionated heparin before 16 weeks and after 36 weeks' gestation plus a Vitamin K antagonist from 16 to 36 weeks until after delivery. The study documented no fetal losses among antithrombin, protein C or protein S deficient women who received treatment, 7% losses among non-deficient women who received treatment, and 45% in untreated antithrombin-deficient women (adj. RR = 0.07, 95% CI: 0.001–0.7, P = 0.02 in women who received treatment vs. women who did not, respectively).

Di Nisio et al. [60] conducted a Cochrane review of RCTs and quasi-RCTs of anti-coagulant treatments in women with a history of fetal loss without apparent causes other than inherited thrombophilias (2 trials; N = 242 women with inherited thrombophilias and prior loss). The review included a study in France by Gris et al. [64] that determined that enoxaparin was superior to low-dose aspirin in preventing stillbirth, as the live birth rate among women with prior pregnancy losses was 10 times higher in the enoxaparin group compared to controls given low-dose aspirin (RR = 10.00, 95% CI: 1.56–64.20) ***[LOE: 1-]***. Another RCT [65] testing tinzaparin sodium (an LMWH) versus aspirin showed a lower rate of miscarriage in the LMWH group (N = 31) compared to the aspirin group (N = 31), though statistically insignificant (RR = 0.55, 95% CI: 0.23–1.29) ***[LOE: 2+]***. When compared to no treatment, LMWH was associated with dramatically lower risk of fetal death. Significantly lower risk of fetal death was reported by a small study (N = 52) comparing LMWH plus aspirin to no treatment (OR = 0.10, 95% CI: 0.002–0.98) [66]***[LOE:2-]***. In a cohort study in the Netherlands [63], thromboprophylaxis with heparin or LMWH reduced the fetal death rate to 0% in antithrombin-protein-C- or protein-S-deficient women, compared with 45% in deficient women given no treatment (P = 0.01) ***[LOE:2-]***.

Cardiac indications

Other observational studies have evaluated fetal outcomes after anti-coagulant therapy for cardiac problems or following cardiac surgery. Several studies explored pregnancies in women after cardiac valve replacement, including a Korean study [67] which compared the impact on birth outcomes of warfarin (N = 5), heparin (N = 18) and heparin for the first trimester followed by warfarin until 20 weeks gestation (N = 18). The lowest SBR occurred in the heparin-only group (5.6% in the heparin versus 40.0% and 44.4% in the warfarin and heparin-warfarin groups, respectively) ***[LOE:2-]***. In a safety study of nadroparin among women with replaced heart valves, Lee et al. [68] reported a significantly lower fetal death rate (8.7% vs 50%, P = 0.01) among women treated with nadroparin (N = 23) compared with coumarin plus aspirin (N = 8) ***[LOE: 3]***. Kawamata et al. [69] reported that among women with heart valve replacements (N = 12; N = 16 pregnancies) switched from warfarin to heparin treatment from 6–13 weeks of gestation until delivery, only one fetal death occurred ***[LOE: 3]***.

Repeated unexplained prior loss(es)

Only one study was identified that tested low molecular weight heparin in women with repeated unexplained losses excluding women with thrombophilias or anti-phospholipid antibodies. This RCT by Dolitzky et al. in Israel among women with at least 3 prior first-trimester or at least 2 prior second-trimester losses [70] (N = 107) found no difference in live birth rates between enoxaparin and aspirin.

Safety

Heparin appears to be safe throughout pregnancy. In Denmark, Sorensen et al. [71] used national prescription, birth registry, and hospital discharge data to analyze the safety of LMWH use in pregnancy, finding no increased risk of stillbirth, malformations, or LBW among pregnant women treated with LMWH compared with women who received no prescription drugs in pregnancy ***[LOE: 2+]***.

##### New meta-analyses

**Figure 1:**
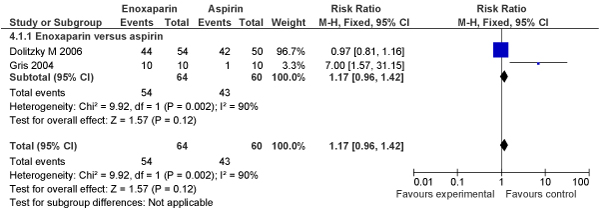
**Meta-analysis (Forest plot) of impact of LMWH versus aspirin on live birth rate in women with recurrent pregnancy loss (Fixed model)**.

**Figure 2:**
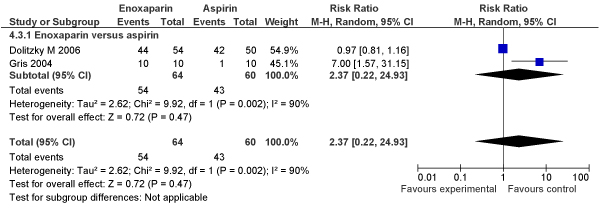
**Meta-analysis (Forest plot) of impact of LMWH versus aspirin on live birth rate in women with recurrent pregnancy loss (Random model)**.

We conducted independent meta-analyses on available RCTs (N = 2) comparing the efficacy of LMWH enoxaparin (N = 64) to aspirin (N = 60) on live birth rate in women with recurrent pregnancy loss; finding no improvement of LMWH over aspirin in live birth rates (RR [fixed] = 1.17, 95% CI: 0.96–1.42) (RR [random] = 2.37, 95% CI: 0.22–24.93) (Figures 1, 2).

**Figure 3:**
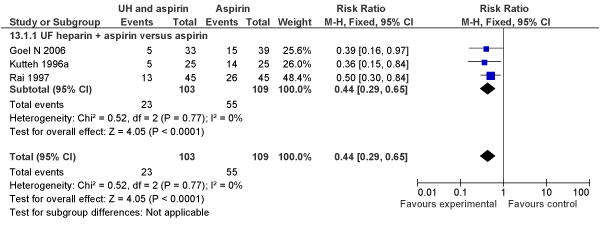
**Meta-analysis (Forest plot) of impact of unfractionated heparin and aspirin versus aspirin alone on pregnancy loss (miscarriages plus stillbirths) in women with antiphospholipid antibody or lupus anticoagulant (Fixed model)**.

**Figure 4:**
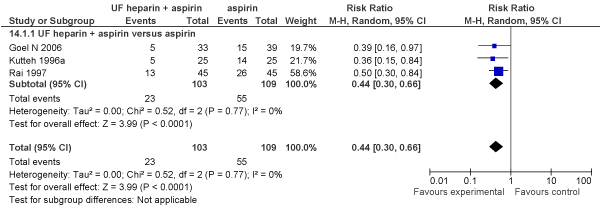
**Meta-analysis (Forest plot) of impact of unfractionated heparin and aspirin versus aspirin alone on pregnancy loss (miscarriages plus stillbirths) in women with antiphospholipid antibody or lupus anticoagulant (Random model)**.

We also compared the impact on pregnancy loss of unfractionated heparin plus aspirin versus aspirin alone (3 RCTs; N = 212 women) in women with antiphospholipid antibody or lupus anticoagulant. The combination treatment with unfractionated heparin was associated with a 56% reduction in pregnancy loss (RR [Fixed] = 0.44, 95% CI: 0.29–0.65)(RR [Random] = 0.44, 95% CI: 0.30–0.66) (Figures 3 and 4).

##### Conclusion

The use of thromboprophylaxis in women with a history of at least two miscarriages or one later intrauterine fetal death without apparent causes other than inherited thrombophilia remains a matter of debate (overall evidence Grade C). While some clinicians prescribe anti-thrombotic agents as the last therapeutic resort in these women, this approach is largely based on data from non-randomised trials and on indirect evidence of an anti-coagulation benefit in women with anti-phospholipid antibody syndrome and recurrent pregnancy loss. According to a recent review [60], insufficient evidence exists (only 2 poorly controlled RCTs with questionable external validity, within which only a small subgroup of women met the inclusion criteria) to recommend the use of anti-thrombotic agents in women with unexplained recurrent pregnancy losses without anti-phospholipid antibodies. While aspirin appears not to improve gestational outcomes in these women [72,73], LMWH appeared to increase the live birth rate relative to aspirin [64].

Despite the small size of the study groups, our results support the assertion that heparin, whether unfractionated or LMWH, is not teratogenic and can be safely used throughout pregnancy where indicated for maternal conditions including thrombophilias. For women with anti-phospholipid antibodies, unfractionated heparin plus aspirin is superior to aspirin alone [51]. In women without anti-phospholipid syndrome, LMWH alone was more effective than aspirin [60]. Used appropriately as anti-coagulant therapy for clotting disorders and anti-phospholipid syndrome, heparin shows strong evidence of benefit in reducing stillbirths and perinatal mortality.

#### Anti-oxidant treatment to prevent or treat pre-eclampsia

##### Background

Anti-oxidants, including free radical scavengers and enzymes that inhibit peroxidase reactions which produce free radicals [74], protect proteins and enzymes from oxidation and destruction by free radicals, and help to maintain cellular membrane integrity. Examples include vitamin C (ascorbate), vitamin E (tocopherols), carotenoids, glutathione, glutathione peroxidase, catalase, and superoxide dismutase. Women with pre-eclampsia have been documented to have lower plasma and placental concentrations of anti-oxidants [75,76], hypothesised to be due to placental underperfusion. Theoretically, anti-oxidants could increase women's resistance to oxidative stress, which depletes maternal stores of anti-oxidants. In cases where placental ischaemia causes subsequent oxidative stress, such supplements might limit systemic and uteroplacental endothelial damage to maternal vasculature and organs, thus slowing or preventing the progression of pre-eclampsia.

##### Literature-based evidence

Our literature search identified three related Cochrane reviews, all conducted by the same researcher, and one RCT (Table 7). Rumbold et al. [77] (10 RCTs, 5 high-quality, N = 6533 women) identified trials of anti-oxidants and found that most tested combined vitamin C and E therapy (Additional file 14). There was no significant difference between anti-oxidant and control groups in risk of pre-eclampsia (RR = 0.73, 95% CI: 0.51–1.06; 9 RCTs, N = 5446 women) or any other primary outcome, including severe pre-eclampsia (RR = 1.25, 95% CI: 0.89–1.76; 2 RCTs, N = 2495 women), pre-term birth (< 37 weeks) (RR = 1.10, 95% CI: 0.99–1.22; 5 RCTs, N = 5198 women), SGA infants (RR = 0.83, 95% CI: 0.62–1.11; 5 RCTs, N = 5271 babies) or pregnancy loss (RR 1.32, 95% CI 0.92 to 1.90; four trials, 5144 babies). Women allocated to anti-oxidants were more likely to self-report abdominal pain late in pregnancy (RR = 1.61, 95% CI: 1.11–2.34; 1 RCT, N = 1745 women), require anti-hypertensive therapy (RR = 1.77, 95% CI: 1.22–2.57; 2 RCTs, N = 4272 women) and require an antenatal hospital admission for hypertension (RR = 1.54, 95% CI: 1.00–2.39 [NS]; 1 RCT, N = 1877 women). However, for the latter two outcomes, this was not clearly reflected in an increase in any other hypertensive complications. An earlier Cochrane review by Rumbold and Crowther [78] (Additional file 15) assessed the impact of Vitamin C supplementation alone during pregnancy (5 RCTs, N = 766 women). No difference was seen between women supplemented with vitamin C alone or in combination with other supplements compared with placebo for the risk of stillbirth (RR = 0.87, 95% CI: 0.41–1.87, 3 RCTs, N = 539 women), PMR (RR = 1.16, 95% CI: 0.61–2.18, 2 RCTs, N = 238 women), birth weight (weighted mean difference (WMD) -139.00 g, 95% CI: -517.68 to 239.68, 1 RCT, N = 100 women) or IUGR (RR = 0.72, 95% CI: 0.49–1.04, 2 RCTs, N = 383 women). Women supplemented with vitamin C compared with placebo were at increased risk of pre-term birth (RR = 1.38, 95% CI: 1.04–1.82, 3 RCTs, N = 583 women). Rumbold and Crowther [79] (Additional file 15) also examined the impact of Vitamin E supplementation (4 RCTs, N = 566 women at risk of, or with, pre-eclampsia). All trials assessed vitamin E in combination with other supplements and two trials were published as abstracts only. Meta-analysis showed no impact of Vitamin E on risk of stillbirth (RR = 0.77, 95% CI: 0.35–1.71, 2 RCTs, N = 339 women), neonatal death (RR = 5.00, 95% CI: 0.64–39.06, 1 RCT, N = 40 women), PMR (RR = 1.29, 95% CI: 0.67–2.48, 1 RCT, N = 56 women), pre-term birth (RR = 1.29, 95% CI: 0.78–2.15, 2 RCTs, N = 383 women), IUGR (RR = 0.72, 95% CI: 0.49–1.04, 2 RCTs, N = 383 women) or birth weight (weighted mean difference -139.00 g, 95% CI: (-)517.68-239.68 g, 1 RCT, N = 100 women), using fixed-effect models.

**Table 7:** Impact of anti-oxidant supplementation on stillbirth and perinatal mortality

**Source**	**Location and Type of Study**	**Intervention**	**Stillbirths/Perinatal Outcomes**
***Reviews and meta-analyses***			

Rumbold et al. 2008 [77]	UK, Australia, South Africa. Meta-analysis (Cochrane). 4 RCTs included.	Compared impact of supplementation with any anti-oxidants vs. control/placebo.	Fetal death rate (miscarriage+SB): RR = 1.32 (95% CI: 0.92–1.90) **[NS]** [66/2569 vs. 50/2575 in intervention vs. control groups, respectively.]

Rumbold et al. 2005 [78]	UK, South Africa Meta-analysis (Cochrane). 3 RCTs included	Compared impact of supplementation with Vitamin C vs. control/placebo.	SBR: RR = 0.87 (95% CI: 0.41–1.87) **[NS]** [9/268 vs. 11/271 in intervention vs. control groups, respectively.]

Rumbold et al. 2005 [79]	UK, South Africa Meta-analysis (Cochrane). 2 RCTs included.	Compared impact of supplementation with Vitamin E vs. control/placebo.	SBR: RR = 0.77 (95% CI: 0.35–1.71) [8/168 vs. 11/171 in intervention vs. control groups, respectively.]

***Intervention studies***			

Roberts et al. 2008 [80]	USA. RCT. N = 9969 low-risk nulliparous women 9–16 weeks gestation at enrolment.	Compared impact of supplementation with Vitamin C (1000 mg/day) plus Vitamin E (400 IU/day) vs. placebo.	Severe hypertension **or **pregnancy-related hypertension with at least one of the following: SGA, hepatic or renal dysfunction, eclampsia, stillbirth, or neonatal death before discharge: 6.1% vs. 5.8% in antioxidant vs. placebo groups, respectively/ Pre-eclampsia (7.2% vs. 6.7% in antioxidant vs. placebo groups, respectively).

Roberts et al. [80] reported results from a double-blind RCT of Vitamins C and E versus placebo administered to low-risk nulliparous women (N = 9969). The study found no differences in the primary outcome, defined as either severe hypertension or pregnancy-related hypertension with at least one of the following: SGA, hepatic or renal dysfunction, eclampsia, stillbirth, or neonatal death before discharge (6.1% vs. 5.8% in antioxidant vs. placebo groups, respectively). Additionally, there was no impact on the incidence of pre-eclampsia (7.2% vs. 6.7% in antioxidant vs. placebo groups, respectively).

##### Conclusion

The overall level of evidence for these studies for impact on pregnancy outcomes is Grade D. Based on this limited evidence, with no trials conducted in under-nourished populations, there appears to be little benefit of anti-oxidants for the prevention of pre-eclampsia and its related complications, as studies have not reported consistent impact. There is no evidence suggesting that anti-oxidant supplements, specifically vitamins C or E or both, avert stillbirths or other perinatal complications such as SGA or neonatal death. Given that the studies reviewed here may not be generalisable to under-nourished populations, studies are needed that test anti-oxidant supplementation in such populations to ascertain whether treatment can impact rates of adverse pregnancy outcomes. Interventions included in this section and paper have aimed to prevent pre-eclampsia/eclampsia; the management of pre-eclampsia/eclampsia in pregnancy has been dealt with in Paper 5 [81].

#### Management of intrahepatic cholestasis in pregnancy

##### Background

Maternal intrahepatic chlolestasis is a liver disorder that causes maternal morbidity during pregnancy, usually manifest as itching (pruritis) as well as an increased risk of associated perinatal mortality. Cholestasis is the most common liver disorder specific to pregnancy and is a principal cause of jaundice in the third trimester. Cholestasis is associated with significantly increased risk of fetal distress, pre-term delivery and stillbirth; however, the mechanisms by which cholestasis causes these complications are still unclear, and treatment strategies are thus largely empiric. The risks of adverse pregnancy outcomes associated with cholestasis increase near term and are unrelated to the severity of symptoms. Early delivery (at 36 weeks gestation) is an intervention that may be considered in cases of severe intrahepatic cholestasis, particularly if there is suspected fetal distress and lung maturity has been confirmed. Administering Vitamin K to the baby immediately after birth may help prevent intracranial bleeding. There is limited evidence about the effectiveness of other treatments, including guar gum, activated charcoal, S-adenosyl-L-methionine (SAMe) and ursodeoxycholic acid (UDCA), in alleviating maternal itching or improving perinatal outcomes.

##### Literature-based evidence

Our literature search identified no Cochrane reviews and four trials assessing interventions for maternal intrahepatic cholestasis in pregnancy (Table 8). Three of these four were RCTs and compared the impact of different treatments for intrahepatic cholestasis on stillbirths or perinatal mortality. Binder et al. [82] compared S-adenosyl-L-methionine (SAMe) monotherapy (N = 78), ursodeoxycholic acid (UDCA) (N = 78) and combined therapy (N = 78) and documented no perinatal deaths in any group. Comparing UDCA, dexamethasone, and placebo in a cohort of women (N = 130), Glantz et al. [83] recorded only one intrauterine fetal death, which occurred in the placebo group. In Chile, Palma et al. [84] tested UDCA versus placebo, reporting only one perinatal death in the placebo group (0/8 in the UDCA group versus 1/7 in the placebo group).

**Table 8:** Impact of management of intrahepatic cholestasis on stillbirth and perinatal mortality

**Source**	**Location and Type of Study**	**Intervention**	**Stillbirths/Perinatal Outcomes**
***Intervention studies***			

Binder et al. 2006 [82]	Czech Republic. RCT. Singleton pregnancies (N = 78) < 36 wks with a moderate or severe form of cholestasis recruited 1999–2004.	Compared the impact among three groups [SAMe (S-adenosyl-L-methionine) monotherapy (group 1), UDCA (ursodeoxycholic acid) (group 2), and combined therapy (group 3)] on PMR.	PMR: 0/25 vs. 0/26 vs. 0/27 in groups 1, 2 and 3, respectively **[NS]**

Glantz et al. 2005 [83]	Sweden. Double-blind, placebo-controlled RCT. Pregnant women (N = 130; N = 47 UDCA, N = 36 dexamethasone, N = 47 placebo) with cholestasis.	Compared the impact on perinatal outcomes of treatment of cholestasis with UDCA (intervention #1), or dexamethasone (intervention #2), vs. placebo (controls).	Fetal death (miscarriage + SB): 0/47 vs. 0/36 vs. 1/47 in intervention group #1, intervention group #2, and controls, respectively.

Palma et al. 1997 [84]	Chile. Secondary case-referral center. RCT. Pregnant women (N = 15) with early-onset obstetric cholestasis.	Compared the impact on perinatal outcomes of treatment with UDCA (intervention) vs. placebo (controls).	SBR: 0/8 vs. 1/7 in intervention vs. control groups, respectively.

Roncaglia et al. 2002 [85]	Italy (Milan). University hospital. Intervention trial using prospective cases. Pregnant women (N = 218) with obstetric cholestasis and historical series data.	Compared the effect of a management protocol for cholestasis incorporating transcervical amnioscopy, standard monitoring of fetal well-being with 2× weekly non-stress testing and AFI indices, and induction of labour at 37 weeks if high-risk (intervention) vs. historical controls on obstetric outcome.	SBR: 0/218 vs. 14/888 in intervention vs. control groups, respectively (P = 0.045).

The fourth trial that we identified [85] prospectively evaluated the effect of a management protocol for cholestasis including surveillance for presence of meconium using transcervical amnioscopy after 36 weeks, semi-weekly non-stress testing and amniotic fluid indices, and induction of labour at 37 weeks. Comparing the study's rate of fetal death with historical series data using expectancy and conventional monitoring of fetal well-being in pregnancies complicated by cholestasis, the fetal death rate was significantly lower in the study group than in the series data (1/218 vs. 14/888, respectively, P = 0.045), and the Caesarean section rate was not significantly different ***[LOE: 2+]***.

##### Conclusion

The intervention trial reviewed above that compared a management protocol in patients with intrahepatic obstetric cholestasis to standard monitoring of fetal well-being suggests that such protocols can reduce the SBR without increasing the cesarean delivery rate. However, this evidence is inconclusive (overall Grade D), and more studies are needed. There remains a need for studies to identify the mechanisms by which cholestasis causes perinatal mortality, as well as safety and efficacy studies for optimal pharmacological treatment regimens to treat cholestasis. The impact of different treatments for cholestasis vis-à-vis stillbirths or perinatal mortality remains inconclusive, as there are only a few trials on the subject.

#### Maternal plasma exchange

##### Background

Maternal autoimmune or coagulation disorders may trigger the production of embryotoxic antibodies, which jeopardise pregnancy and lead to intrauterine death. These conditions include anti-D antibodies produced following maternal Rhesus alloimmunisation, lupus anti-coagulant and anti-cardiolipin that mark anti-phospholipid syndrome, and anti-Ro/SSA and anti-La/SSB antibodies in systemic lupus erythematosus, among others [86]. Plasma exchange, or plasmapheresis, often in conjunction with steroid treatment or intravenous administration of immunoglobulins, is a means to remove or suppress circulating autoimmune or anti-coagulation factors in the blood and have been employed in a range of situations [87]. Reducing these embryotoxic factors could prevent pregnancy loss, and provide an important alternative when intrauterine transfusion for Rhesus alloimmunisation, or anti-coagulant therapy (e.g., heparin +/- low dose aspirin) for anti-phospholipid syndrome fails. In recurrent abortion involving the presence of anti-P antibodies in mothers with the rare P blood group [88], plasmapheresis has proven effective in reducing pregnancy loss.

##### Literature-based evidence

Our review identified two observational studies. No Cochrane or other systematic review was identified on the subject. El-Haieg et al. [89] conducted a study which assessed plasmapheresis plus low dose prednisone on obstetric and neonatal outcomes among unsuccessfully treated pregnant women with documented anti-phospholipid syndrome. There were no perinatal deaths ***[LOE: 3]***. According to the study of Angela et al. [90], fourteen high-risk cases of Rh alloimmunised women were treated by intensive plasma exchange on the cell separator throughout their pregnancies. The expected stillbirth rate in this series as determined by their past obstetric histories and anti-D levels was 62%. Intrauterine transfusion was given to only two of the infants and both were later stillborn ***[LOE: 3]***.

##### Conclusion

Only two case series were identified, one on plasma exchange for severe Rh disease and the other (prednisone and plasmapheresis) for anti-phospholipid syndrome (Grade D evidence). Further large-scale studies are needed to investigate the effect of plasma exchange on stillbirths/perinatal mortality. Although robust evidence of an impact on stillbirths and perinatal deaths is lacking, plasma exchange commenced early in pregnancy may be an alternative therapy for severe Rh haemolytic disease and anti-phospholipid syndrome and may be useful in preventing stillbirths.

#### Cervical cerclage

##### Background

Cervical incompetence is estimated to occur in approximately 1% of the obstetric population, and 8% of women who experience repeated spontaneous abortions [91]; true rates are difficult to assess given a range of definitions but are likely higher. Cervical incompetence is increasingly recognised as associated with a continuum of complications including second-trimester miscarriage as well as pre-term birth. Incompetent cervix may be diagnosed presumptively following recurrent unexplained second trimester losses or pre-term births. However, increasingly, incompetent cervix is assumed when a short cervix is diagnosed via transvaginal ultrasound. Cervical cerclage, the surgical process of stitching the cervix closed to prevent further shortening and dilatation, can be either planned (history-indicated), urgent (ultrasound-indicated) or emergent (exam-indicated, generally in an emergency situation) [92]. In women with 3 or more pre-term births or second-trimester losses, history-indicated cerclage is typically placed between 12–14 weeks of gestation. Evidence suggests that ultrasound-indicated cerclage, on the other hand, may be most effective when placed after 14 weeks but before 24 weeks of gestation [93].

The two most common transvaginal methods for placement of a cervical stitch are modifications of procedures developed by Shirodkar and McDonald [94,95]. The Shirodkar technique involves an incision in the cervix at the level of the internal os and requires dissecting the bladder free; the cervix is then encircled with a purse-string suture through the broad cervical ligament. The McDonald technique does not require dissecting the bladder free, using an encircling suture around the cervix to approximate the level of the internal os. Ultrasound examination post-cerclage has shown that the Shirodkar procedure achieves longer cervical length than the McDonald procedure [96], but no differences in rates of pre-term birth have been observed between the two methods [97]. In women with short and/or scarred cervixes, or where vaginal cervical cerclage has not worked, placement of the stitch can be difficult; in these women, transabdominal cervical cerclage can be performed, but patients require caesarean section delivery.

Despite being widely practiced, cerclage is controversial because its benefits in maintaining pregnancy and preventing pre-term birth have been marginal. A number of meta-analyses and reviews including pooled estimates of cerclage impact have found conflicting evidence of impact on pre-term birth rates associated with cerclage [92,98]. However, recent good-quality trials of ultrasound-indicated cerclage for short cervix, including a large, rigorous RCT, have demonstrated a statistically significant impact of cervical cerclage in reducing pre-term birth rates [99]. Cerclage has been studied primarily for its impact on pre-term birth. The procedure may also reduce stillbirth, though this has not been a principal focus of cerclage studies to date.

##### Literature-based evidence

For this review, we identified two systematic reviews and 5 other intervention and observational studies of cervical cerclage that reported impact on stillbirths and/or perinatal mortality (Table 9). One Cochrane review by Drakeley et al. [92] compiled all adequately randomised RCTs (6 trials, N = 2175 women) comparing cervical cerclage with expectant management or no cerclage during pregnancy, as well as trials comparing different cerclage techniques, alone or with other interventions (Additional file 16). In the 4 included trials of prophylactic cerclage versus no cerclage, pooled results revealed no overall reduction in perinatal death (RR = 0.80, 95% CI: 0.48–1.36), pregnancy loss, or pre-term delivery rates before 28 and before 34 weeks. Cervical cerclage was associated with mild pyrexia, increased tocolytic use, and increased hospital admissions, but had no associated serious morbidities ***[LOE: 1++]***. Another meta-analysis of RCTs of cerclage [100] (N = 7 trials, N = 2091 women) found no significant impact of cerclage on pregnancy loss or death before discharge from hospital in singleton pregnancies (OR = 0.81; 95% CI: 0.60–1.10 [NS]), though the small sample size indicated a need for additional large trials (Additional file 17). However, in the logistic regression analysis, an adverse association of cerclage with perinatal deaths was observed for multiple pregnancies (OR 5.88; 95% CI 1.14–30.19), suggesting cerclage should be avoided in multiple pregnancies. Neither indication for cerclage nor obstetric history was found to have a statistically significant impact on the effect of cerclage ***[LOE: 1++]***. The only other RCT compared the impact of outpatient versus inpatient cerclage [101]; live birth rate was slightly higher among the inpatient group but the difference was not statistically significant.

**Table 9:** Impact of cervical cerclage on stillbirth and perinatal outcomes

**Source**	**Location and Type of Study**	**Intervention**	**Stillbirths/Perinatal Outcomes**
***Reviews and meta-analyses***			

Drakeley et al. 2003 [92]	Netherlands, France, UK, South Africa. Meta-analysis (Cochrane). 6 RCTs included (N = 2059 women).	Assessed the effects of cervical cerclage (intervention) vs. no cerclage (controls).	PMR: RR = 0.80 (95% CI: 0.48–1.36) **[NS]** [24/1035 vs. 31/1024 in intervention vs. control groups, respectively.

Jorgensen et al. 2007 [100]	Netherlands, USA, Nigeria, UK, France, Hungary, Norway, Italy, Belgium, Zimbabwe, South Africa, Iceland, Ireland, Canada, Brazil Slovenia, Greece and Chile. Meta-analysis. 7 RCTs included (N = 2091 women).	Assessed the effects of cervical cerclage (intervention) vs. no cerclage (controls).	(Singleton gestations) Pregnancy loss or death before hospital discharge: OR = 0.81 (95% CI: 0.60–1.10) **[NS]** (Multiple gestations) Pregnancy loss or death before hospital discharge: OR = 5.88 (95% CI: 1.14–30.19).

***Intervention studies***			

Blair et al. 2002 [101]	West Indies. RCT. Pregnant women (N = 50) with cervical incompetence.	Compared the impact of cervical cerclage between inpatient care for 3 days post-procedure, spending 3 days in hospital post-procedure (intervention) vs. outpatient bed rest (controls). Both groups given salbutamol tablets postoperatively for tocolysis.	Live birth rate: 20/23 vs. 18/23 (86.9% vs. 78.3%) in intervention vs. control groups, respectively **[NS]**

Jaswal et al. 2006 [198]	India. Quasi-RCT. Pregnant women (N = 37) being expectantly managed for placenta previa.	Compared the impact of cervical cerclage (intervention) versus no cerclage (controls).	PMR: 0/18 vs. 8/19 in intervention vs. control groups, respectively (P < 0.01).

***Observational studies***			

Debbs et al. 2007 [104]	USA. Retrospective case series. Pregnant women (N = 75) with negative evaluation for recurrent pregnancy loss and ≥ 1 previous unsuccessful transvaginal cerclage.	Assessed the impact of transabdominal cerclage on birth outcomes.	Live birth rate: 96% after transabdominal cerclage.

Fick et al. 2007 [102]	USA. A cohort study. Pregnant women (N = 88 women; N = 9 pregnancies) with transabdominal cerclage.	Compared the live birth rate before and after transabdominal cerclage.	Live birth rate: 93% vs. 18% after vs. cerclage, respectively; P < 0.001).

Gesson-Paute et al. 2007 [103]	France. Retrospective study. Transabdominal cerclages (N = 12) performed from 1988–2005.	Compared the live birth rate during the period where transabdominal cerclage was performed vs. the pre-cerclage period.	Live birth rate: 93% vs. 17% after vs. before cerclage, respectively.

A retrospective study of prophylactic cerclage in multiple gestation by Strauss et al. reported no impact on perinatal mortality, and a non-statistically significantly elevated risk of perinatal morbidity in higher-order gestations (quadruplets and quintuplets) ***[LOE: 2-]***.

Three observational studies highlighted the effectiveness of transabdominal cerclage as an alternative after unsuccessful transvaginal cerclage. Fick et al. [102]*** [LOE: 2+]***, and Gesson-Paute et al. [103]***[LOE: 2-] ***reported live birth rates after transabdominal cerclage of 96%, and 93% respectively, compared to 18% [102] and 17% [103] before the procedure. Debbs et al. [104] conducted a similar study and reported a 93% live birth rate after the procedure ***[LOE: 3]***.

##### Conclusion

The systematic reviews on cervical cerclage are of sufficiently high quality to permit a Grade B assessment, but are concerned primarily with impact on pre-term birth, and report statistically non-significant impact on stillbirth. Perinatal mortality outcomes reported by the large meta-analysis on cerclage [92] showed no conclusive evidence of benefit on stillbirth. The largest reviewed study in this meta-analysis by the MRC/RCOG [105] reported a significant reduction in very pre-term deliveries, and more recent data from a large RCT [99] has demonstrated a positive impact on pre-term birth in pregnant women with ultrasound-confirmed short cervix. This definitive trial indicates that ultrasound-indicated cervical cerclage is of benefit to reduce risk of preterm birth. Cerclage is not routinely indicated in multiple pregnancies [100]. It is possible that cervical cerclage may reduce pregnancy loss in singleton pregnancies [100], particularly high-risk pregnancies, but it is unlikely that another large RCT like the Owen study [99] will be conducted to evaluate the impact of cerclage on stillbirth. It should be noted that measurement of the impact of cerclage on pregnancy outcomes is biased by the frequent exclusion from studies of women with true cervical incompetence. Cervical cerclage is currently inconclusive evidence of benefit for preventing stillbirth despite its proven impact on pre-term birth. Future studies may confirm reductions in perinatal mortality and help identify the sub-groups of women in whom it is most effective (e.g., ultrasound-indicated cerclage).

### Infection control and treatment

#### Anti-helminthics during pregnancy in hookworm-endemic regions

##### Background

Two species of hookworm, *Ancylostoma duodenale *and *Necator Americanus*, typically infect humans. Found throughout the tropics and subtropics, hookworm infestation is significantly associated with anaemia in women and children in endemic areas. Anaemia during pregnancy is associated with premature delivery, LBW, maternal ill health, and maternal death [106]. In areas endemic for hookworm, routine antenatal anti-helminthic therapy could reduce the prevalence of anaemia in pregnancy, hypothetically preventing pregnancy losses associated with anaemia. In two studies of anti-helminthic treatment of women in pregnancy, the mean decrease in haemoglobin concentration from the first to the third trimester in women receiving a single dose of albendazole was 6.6 g/L less than women receiving placebo (P = 0.003) [107,108], but another study showed no impact on haemoglobin level [109]. Where the prevalence of hookworm is more than 20% to 30%, the WHO recommends that pregnant women receive treatment (mebendazole, albendazole, levamisole or pyrantel) after the first trimester. At present, however, this is not a widely accepted control strategy in part because of a lack of data on the safety of anti-helminthics. Potential teratogenicity is the principal concern surrounding use of anti-helminthics, as side effects are few [110]. The limited studies on this subject have found no evidence of fetal malformations attributable to anti-helminthics used during pregnancy, but some studies have recommended that anti-helminthics not be used during the first trimester as a precaution until larger safety studies in pregnancy are available [111-113].

##### Literature-based evidence

Several studies of deworming in pregnancy have demonstrated benefits on maternal nutrition outcomes and anaemia [109,114,115]. Our literature review identified two intervention studies and one observational study (Table 10).

**Table 10:** Impact of anti-helminthics on stillbirth and perinatal mortality

**Source**	**Location and Type of Study**	**Intervention**	**Stillbirth/Perinatal mortality outcomes**
***Intervention study***			

Gyorkos et al. 2006 [113]	Peru (Amazon region). RCT. Data on adverse birth outcomes (N = 1042 births).	To compare of the impact of antenatal mebendazole (500 mg single dose; intervention) plus iron supplement vs. placebo plus iron supplement (controls) on the occurrence of adverse birth outcomes.	SBR: 8 vs. 4 in intervention vs. control groups, respectively. **[NS]** ENND: 3 vs. 6 in intervention vs. control groups, respectively. **[NS]** PMR: 22/1000 vs. 20.2/1000 in intervention and control groups, respectively (P = 0.840). **[NS]** Pre-term: 28 vs. 31 respectively (P = 0.664). **[NS]**

Christian et al. 2004 [117]	Nepal (Sarlahi). RCT.	Compared the impact of two doses of albendazole in pregnancy (intervention) vs. no treatment (controls) on birth outcomes.	NMR: OR = 0.54 (95% CI: 0.37–0.78) [unpublished data]

***Observational Study***			

De Silva et al. 1999 [116]	Sri Lanka, hospital based. Cross-sectional survey.	Compared impact on major congenital defects, stillbirth, perinatal death, and LBW among babies of mothers who had taken mebendazole during pregnancy (intervention) with those whose mothers had not taken an anti-helmintic (controls).	PMR: OR = 0.55 (95% CI: 0.4–0.77) [19/1000 vs 33/1000 in intervention vs. control groups, respectively]. LBW: OR = 0.47 (95% CI: 0.32–0.71) [1.1 vs 2.3% in intervention vs. control groups, respectively]. Major congenital malformations: OR = 1.24 (95% CI: 0.8–1.91, P = 0.39) **[NS]** [97/5275 vs 26/1737 in intervention vs. control groups, respectively].

De Silva et al. [116] assessed the effect of mebendazole therapy during pregnancy on birth outcomes in a cross-sectional retrospective study in Sri Lanka in 1995. Stillbirths and perinatal deaths were significantly less common in the mebendazole group (19/1000 vs. 33/1000; RR = 0.55, 95% CI: 0.4–0.77), as was the proportion of LBW infants (1.1 vs. 2.3%, RR = 0.47, 95% CI: 0.32–0.71). A slightly higher, but statistically non-significant, rate of congenital defects was found in women who had taken the drug in the first trimester ***[LOE: 2+]***. In contrast, another study by Gyorkos et al. [113] comparing antenatal mebendazole (500 mg single dose) given with iron supplements vs. placebo with iron supplementation did not show any statistically significant differences between the mebendazole group (N = 28) and the placebo group (N = 31) in rates of miscarriages, malformations, stillbirths, early neonatal deaths and pre-term births ***[LOE: 2+]***. However, unpublished data from a study of anti-helminthic treatment, maternal anaemia, and birth weight in Nepal [117] showed a statistically significant reduction in neonatal mortality associated with 2 doses of albendazole during pregnancy compared to controls (OR = 0.54, 95% CI: 0.37–0.78) ***[LOE: 2++]***.

##### Conclusion

Mebendazole therapy should be avoided during the first trimester pending further evidence on safety. The overall quality of evidence from the literature reviewed is Grade C, however there is some evidence of impact of deworming on stillbirths and other perinatal outcomes. Maternal deworming holds promise for improving the health of pregnant women in low-/middle-income countries where intestinal helminthiases are endemic. Consonant with the findings of the studies we reviewed, the Lancet *Maternal and Child Undernutrition Series *recommends deworming during pregnancy in specific, situational contexts (i.e., where prevalence of helminthiases and iron-deficiency anaemia are high) [118]. Given the promising but inconclusive findings of a recent Cochrane review [110], further RCTs, especially effectiveness trials, of maternal anti-helminthic therapy reporting a range of pregnancy and infant outcomes are needed.

#### Syphilis screening and treatment

##### Background

Reported rates of syphilis vary greatly worldwide. Prevalence is generally lower in high-income countries than in low-/middle-income countries. In 1990, the incidence of primary and secondary syphilis in the United States was 20 per 100,000 population, far less than the disease burden of 360 per 100,000 estimated for parts of Africa [119,120]. Syphilis remains a major cause of avoidable perinatal death in many low-/middle-income countries despite being treatable, and despite the WHO recommendation that all pregnant women be tested as part of routine ANC. Among pregnant women with untreated early syphilis, 25% of pregnancies result in stillbirth and 14% in neonatal death, an overall perinatal mortality of about 40% [121]. In early untreated syphilis, a pregnant woman has a 70% chance of transmitting the infection to her fetus [122]. Even after effective screening, many pregnant women with syphilis remain inadequately treated because of delays in initiating treatment or patient non-adherence. Testing for syphilis within antenatal clinics is a strategy that makes test results available immediately so that early treatment can be administered, which could improve treatment of syphilis and reduce perinatal mortality [123].

##### Literature-based evidence

We identified 1 Cochrane review and 8 interventional, quasi-experimental, or observational studies (Table 11) addressing the impact of syphilis screening and treatment on perinatal outcomes, the effectiveness of recommended penicillin treatment regimens, and issues associated with testing and treatment adherence. Unfortunately, the Cochrane review by Walker [124] assessing the impact of antibiotic treatment of syphilis in pregnancy did not report perinatal mortality outcomes, as none of the studies identified (N = 29) met the review's eligibility criteria for comparability.

**Table 11:** Impact of syphilis testing and treatment on stillbirth and perinatal mortality

**Source**	**Location and Type of Study**	**Intervention**	**Stillbirths/Perinatal Outcomes**
***Reviews and meta-analyses***			

Walker et al. 2001 [124]	None. Meta-analysis (Cochrane). 29 RCTs and quasi-RCTs reviewed, 0 studies included.	Assessed the impact of antibiotic treatment for syphilis during pregnancy.	No data, 0 eligible studies.

***Intervention studies***			

Bique et al. 2000 [130]	Mozambique, ANC clinics. Case-control study. 4 suburban ANC clinics (2 intervention clinics, 2 control clinics). Pregnant women (N = 929; N = 453 intervention, N = 476 controls) with positive RPR.	Compared the impact of an intervention offering RPR testing with immediate on-site treatment of seropositive cases with 2.4 million IU benzathine penicillin by specially trained nurse-midwives. The study offered to treat partners of intervention group as well. Controls were offered routine syphilis screening protocol requiring testing at a separate lab, return visits, and payment for treatment (< 50% compliance), with no partner treatment option.	PMR: 13/1000 vs 34/1000 in intervention vs. control groups, respectively (P = 0.03). Fetal death (miscarriage+SB): 13/1000 vs. 26/1000 (P = 0.159) **[NS]**

Donders et al. 1997 [128]	South Africa (Pretoria), hospital-based. Prospective cohort study. HIV-, RPR+ black urban pregnant women (N = 212; N = 135 received ≥ 1 injection)	Assessed dosage impact of 0–3 weekly IM injections of benzathine penicillin G on perinatal outcomes.	PMR secondary to congenital syphilis: RR = 20.5 (95% CI: 2.3–184, P = 0.0015). [0 injections: 7/55 (13%) 1 injection: 2/19 (11%) 2 injections: 1/24 (4.2%) 3 injections: 2/82 (2.4%)] Adjusted when Treponemicidal coverage was < 3 wks

Myer et al. 2003 [123]	South Africa (Hlabisa district, rural KwaZulu Natal), PHC clinics. Cluster RCT. 7 pairs of clinics. Pregnant women (N = 549).	Compared the impact of on-site syphilis testing complemented by laboratory confirmation vs. laboratory testing alone.	PMR: adj. RD: -0.9%; 95% CI: (-) 4.4-2.7, P = 0.31) **[NS]** [33/1000 (18/549) vs. 51/1000 (8/157) in intervention clinics vs. control clinics, respectively.]

Rotchford et al. 2000 [129]	South Africa (Hlabisa district, rural KwaZulu Natal), ANC clinics. Cluster RCT. 12 clinics. Pregnant women (N = 1783) screened for syphilis (N = 158 RPR+; 9% prevalence) at first ANC visit (mean: 24 wks); RPR+ women followed for pregnancy outcome (data available for N = 142 (90%); N = 30 had no treatment; N = 96 had all 3 doses penicillin)	Assessed impact on PMR of inadequate maternal syphilis treatment in presence of adequate screening.	PMR: 15/142 [0 or 1 dose penicillin: 11/43 (260/1000) ≥ 2 doses penicillin: 4/99 (40/1000)] Dose-response relationship observed. (P = 0.0001) PMR risk reduction: 1 dose: 41% reduction 2 doses: 65% reduction 3 doses: 79% reduction

Watson Jones et al. 2002 [127]	Tanzania (Mwanza), ANC clinic. Case-control study. Pregnant women (N = 1688; N = 133 high-titre [RPR titre ≥ 1:8, TPHA/FTA+]; N = 249 low-titre [RPR titre < 1:8, TPHA/FTA+], N = 950 seronegative controls).	To examine the effectiveness of treatment for maternal syphilis with single-dose IM benzathine penicillin (2.4 million units).	Birth outcomes were compared SBR: 23/1000 vs. 25/1000 in treated high-titer women vs. seronegative women. LBW: 6.3% vs. 9.2% in treated high-titer women vs. seronegative women. Adverse pregnancy outcome (combined SBR+LBW): OR = 0.76 (95% CI: 0.4–1.4) **[NS]**.

***Observational studies***			

Delport et al. 1993 [199]	South Africa (Pretoria), ANC clinic. Descriptive study. Kalafong Hospital. Pregnant women (N = 1237) attending ANC.	Assessed the sensitivity, specificity, negative and positive predictive values of the RPR test at ANC compared with gold-standard laboratory Treponema pallidum haemagglutination test.	RPR test: Sensitivity: 92.8% Specificity: 96.3% Negative predictive value: 99.5% Positive predictive value: 64.7%.

Guinness et al. 1988 [125]	Swaziland (Mbabane), Public health unit Prospective cohort study. Pregnant women (N = 283) tested at ANC enrollment: N = 37 (13.1%) TPHA+ and RPR+; N = 87 (30.7%) TPHA+ and RPR-.	Assessed the impact of antenatal screening on perinatal mortality attributable to syphilis. Mothers were tested prenatally and again at delivery; prenatal test found to have sensitivity = 36% and predictive accuracy = 48%.	PMR (untreated active syphilis): 219/1000 (7/32). 12/172 seronegative women had active syphilis (late seroconversion or false negative prenatal test results): 4/12 experienced perinatal death. PMR: 46/1000 (4/87) vs. 28/1000 (4/415) in TPHA+/RPR- vs. syphilis-seronegative women. Screening reduced expected syphilis-attributable PMR from 3.5% to 2.3% (65% of mothers with active syphilis missed treatment; sexual partners were not treated).

Temmerman et al. 2000 [126]	Kenya (Nairobi), maternity hospital. Prospective case control study. Women (N = 12414) delivering at Pumwani Hospital were RPR tested (3%, N = 377 were RPR-positive). TPHA testing confirmed syphilis infection (N = 296). Equal numbers of seronegative women also enrolled; records examined for syphilis testing and treatment during pregnancy.	Assessed the impact of an antenatal syphilis control programme on pregnancy outcome.	Adverse obstetric outcome (LBW or SB): OR = 4.1 (95% CI: 2.3–7.5, P < 0.001). [22.5% vs. 6.6% in untreated syphilis-positive vs. uninfected mothers, respectively.] LBW: OR = 4.0 (P < 0.0001) in untreated syphilis-seropositive mothers vs. uninfected mothers, respectively. SBR: OR = 3.3 (P = 0.028) in untreated syphilis-seropositive mothers vs. uninfected mothers, respectively. OR = 2.5 in treated syphilis-seropositive mothers vs. uninfected mothers, respectively (P < 0.05).

Impact of screening and treatment on perinatal mortality

In a Swaziland hospital, Guinness et al [125] assessed how testing results and subsequent treatment for maternal prenatal syphilis serostatus impacted outcomes. Almost one-third of women (87/283; 31%) were possibly syphilis seropositive (TPHA-positive and RPR-negative); a smaller proportion (37/283; 13%) showed conclusive syphilis infection (TPHA-positive and RPR-positive) at prenatal testing. Perinatal mortality was highest among women with untreated active syphilis (7/24; PMR = 290/1000). Among women with possibly active syphilis, PMR was 50/1000, compared with 30/1000 among seronegative women. Screening and treatment reduced PMR by 34%, but the intervention would have been even more effective if treatment had reached the 65% of mothers with active syphilis who were missed, as well as study participants' sexual partners ***[LOE: 3]***.

In Kenya, Temmerman et al. [126] assessed the impact of an antenatal syphilis control programme on pregnancy outcome, confirming the adverse effect of syphilis infection on stillbirth. Of patients tested for syphilis (N = 12,414), 3% (N = 377) were RPR-positive; 296 of these were also TPHA-positive. Syphilis seropositive women had a higher risk of adverse obstetric outcome, defined as stillbirth or LBW (OR 4.1, 95% CI: 2.4–7.2). Antenatal treatment of RPR reactive women with single-dose benzathine penicillin G (2.4 million units) reduced the risk of adverse outcome, but women who remained RPR-positive after treatment had worse outcomes than uninfected mothers (OR = 2.5, P < 0.05). There was no statistical difference in pregnancy outcome based on gestational age at treatment ***[LOE: 3]***.

Syphilis treatment strategies

Treatment for maternal syphilis with single-dose IM benzathine penicillin is being implemented in many parts of sub-Saharan Africa in conformity with the 1993 Centers for Disease Control and Prevention (CDC) treatment guideline recommending a single dose of benzathine penicillin G (2.4 million units) for primary, secondary, and early latent syphilis in pregnancy, and three doses one week apart for late latent syphilis and latent syphilis of unknown duration. Watson Jones et al. [127] studied the effectiveness of this regimen in a Tanzanian cohort (N = 1688 pregnant women), comparing birth outcomes between high-titer, low-titer, and seronegative women. When treated, there was no statistically significant increased risk of adverse pregnancy outcome (stillbirth or LBW) comparing high-titer (OR = 0.76, 95% CI: 0.4–1.4) or low-titer women (OR = 0.95, 95% CI: 0.6–1.5) to seronegative women. ***[LOE: 2+]***.

Donders et al. [128] administered 0, 1, 2 or 3 benzathine penicillin G intramuscular (IM) injections to HIV-negative black urban women with syphilis (N = 180) in South Africa. Pregnancy outcomes were significantly better among women receiving 2 or more doses of penicillin than women receiving 0 or 1 dose. However, excluding patients treated with oral penicillin derivatives and adjusting for duration of treponemicidal levels, the study found an association of treponemicidal coverage of 3 weeks or less with decreased birth weight (2748 vs. 3130 g, P = 0.004) and higher rates of perinatal mortality (RR = 20.5; 95% CI: 2.3–184) and pre-term birth (RR = 8.5; 95% CI: 2.5–28) compared with treponemicidal coverage lasting longer than 3 weeks ***[LOE: 2-]***.

Rotchford et al. [129] demonstrated the impact on perinatal mortality of inadequate treatment for maternal syphilis despite adequate screening. In a study population with 9% syphilis prevalence, Rotchford used linear and categorical modeling to confirm a dose response protective relationship of penicillin on PMR. One dose reduced the risk by 41%, two doses by 65% and three doses by 79%, compared with no doses. Among those receiving at least one dose, mean delay to the first dose was 20 days. Among those fully treated, mean delay to treatment completion was 34 days ***[LOE: 2++]***.

On-site testing and treatment compliance

On-site syphilis testing in ANC clinics may prove an effective strategy to circumvent problems initiating and completing antibiotic treatment. Myer et al. [123] studied the impact of on-site syphilis testing on treatment delays and rates as well as perinatal mortality. Among women seeking ANC with available test results (N = 7134), 793 (11.1%) tested positive for syphilis. Women at intervention clinics completed treatment 16 days sooner on average (95% CI: 11–21 days), though there was no significant difference in the proportion receiving adequate treatment at intervention and control clinics (64 vs. 69%, respectively), or in PMR (3.3% vs 5.1% in intervention vs. control clinics; adjusted risk difference [RD]: (-)0.9%; 95% CI: (-)4.4-2.7) ***[LOE: 2++]***.

Bique et al. [130] assessed treatment initiation and compliance following RPR testing in seropositive pregnant women (N = 929) attending intervention or control clinics. Women attending intervention clinics were given health education about syphilis, on-site RPR testing and post-test counseling from a specially trained nurse-midwife, with encouragement to notify partners to come for free treatment. Women attending control clinics had standard RPR testing, entailing a multi-step, multi-provider process over a longer time period, and had to pay for their own treatment, resulting in compliance below 50%. PMR was significantly higher in the control group than in the intervention group (1.3% vs. 3.4% in intervention vs. control groups, respectively; P = 0.030) ***[LOE: 2-]***.

##### Conclusion

The nature of studies available for syphilis screening and treatment do not permit more than a Grade D evidence rating and it is recognised that classic RCTs are not possible. However, syphilis during pregnancy is a major cause of avoidable stillbirth and other adverse birth outcomes in many low-/middle-income countries, and interventions in terms of screening and treating syphilis are recommended for inclusion in endemic populations, as syphilis testing and treatment shows some evidence of impact on stillbirth. When treated with the appropriate dose of intramuscular benzathine penicillin, treatment effectively reduces perinatal mortality [129,128] without risk of drug resistance. However, operational issues associated with treatment complicate the formulation of screening and treatment recommendations. Allergy to penicillin may pose problems for acceptability of treatment, but can be addressed with desensitisation therapy. There are a few studies on antibiotics other than penicillin for treatment of maternal syphilis; other drugs may offer alternative treatment options. In terms of service delivery, the studies reported did not support using on-site syphilis testing in addition to laboratory-based testing, although on-site testing may be the only feasible way to offer syphilis interventions in settings where laboratory facilities are lacking or of poor quality. More studies are needed to test the effectiveness of on-site syphilis testing and treatment and its impact on perinatal outcomes, as this strategy appears to improve compliance with treatment. Additionally, given interactions between syphilis and HIV co-infections, current testing and treatment guidelines may be inappropriate for individuals co-infected with HIV [120].

#### Antibiotics and antisepsis for urinary and reproductive tract infections (bacterial vaginosis, asymptomatic bacteriuria, and Group B streptococcus)

##### Background

Maternal genital tract infection or colonisation by pathogenic organisms predisposes women to maternal and perinatal mortality and morbidity. Antibiotics and antiseptic agents have applications during pregnancy to prevent and treat reproductive tract infections, particularly presumptively for suspected or proven bacterial vaginosis (BV) and treatment of asymptomatic bacteriuria, as well as during the intrapartum period for Group B streptococcus (GBS) colonisation to prevent perinatal infection.

Bacterial vaginosis, an imbalance of normal vaginal flora with an overgrowth of anaerobic bacteria and a lack of normal lactobacillary flora, has been associated with poor perinatal outcome, particularly pre-term birth. Documented prevalence in pregnant women is common, ranging from 14% to 21% in Western countries; studies from Asian countries have reported prevalence ranges from 13.6–18% [131]. Asymptomatic bacteriuria, generally defined as true bacteriuria in the absence of specific symptoms of acute urinary tract infection (UTI), occurs in 2% to 10% of all pregnancies in high-income countries [132]. There is considerable evidence that bacteriuria and occult UTIs are more common in low-/middle-income countries, though estimates of prevalence are prone to wide margins of error. A urinalysis study from rural Tanzania using a variety of diagnostic methods found that 40% of nitrite tests and 65% of leukocyte esterase tests were positive at the first antenatal visit [133]. One study reported significant bacteriuria in 86.6% of samples in an antenatal population in rural Nigeria, [134,133]; other African studies have reported prevalences of bacteriuria in similar populations of 10–24% [135-137]. Some evidence from low- and middle-income countries that suggests that UTIs may be frequently missed, misdiagnosed, or improperly treated pharmacologically in these settings [138]. Asymptomatic bacteriuria can lead to symptomatic UTIs, maternal pyelonephritis and septicemia, and is associated with higher rates of pre-term rupture of membranes (PROM) and chorioamnionitis.

Maternal colonisation with GBS infection is a recognised risk factor for neonatal colonisation and sepsis. GBS-colonised women who experience either a long duration of membrane rupture, premature delivery or intrapartum fever are at particularly high risk for transmitting GBS to their infants. Prevalence of colonisation with GBS is closely associated with socioeconomic status and is similar in both pregnant and non-pregnant women [132,139].

##### Literature-based evidence

We identified four Cochrane reviews, one other review, and 2 observational studies (Table 12). King et al. [140] conducted a Cochrane meta-analysis (N = 11 RCTs, N = 7428 women), including the ORACLE study, of the effect of maternal antibiotics in pre-term labour with intact membranes (Additional file 18). The Cochrane meta-analysis found that prophylactic antibiotics reduced rates of maternal infection (RR = 0.74, 95% CI: 0.64–0.87) but had no impact on PMR (RR = 1.22, 95% CI: 0.88–1.70) ***[LOE: 1++]***. In another Cochrane review, McDonald et al. [141] compared antibiotic treatment to placebo/no treatment in cases of BV or intermediate vaginal flora, as well as comparisons between two or more antibiotic regimens (N = 15 RCTs, N = 5888 women) (Additional file 19). Antibiotic therapy effectively eradicated BV (OR = 0.17, 95% CI: 0.15–0.20; 10 RCTs, N = 4357 women), but did not reduce rates of pre-term premature rupture of membranes (PPROM) (OR = 0.88, 95% CI: 0.61–1.28; 4 RCTs, N = 2579 women). Antibiotics did not reduce the risk of pre-term birth before 37 weeks (OR = 0.91, 95% CI: 0.78–1.06; 15 RCTs, N = 5888 women), unless treatment was administered prior to 20 weeks' gestation (OR = 0.63, 95% CI: 0.48–0.84; 5 RCTs, N = 2387 women) or women had confirmed intermediate flora or BV (OR = 0.51, 95% CI: 0.32–0.81; 2 RCTs, N = 894 women). In women with previous pre-term birth, treatment did not affect the risk of subsequent pre-term birth (OR = 0.83, 95% CI: 0.59–1.17, 5 RCTs, N = 622 women). The three studies assessing the impact of metronidazole treatment for BV on perinatal mortality found no significant impact (OR = 0.96, 95% CI: 0.53–1.73) ***[LOE: 1++]***

**Table 12:** Impact of antibiotics for high-risk pregnancies on stillbirth and perinatal mortality

**Source**	**Location and Type of Study**	**Intervention**	**Stillbirths/Perinatal Outcomes**
***Reviews and meta-analyses***			

King and Flenady 2002 [140]	Chile, Denmark, USA, South Africa, UK. Meta-analysis (Cochrane). 9 RCTs included.	Assessed the impact of any antibiotic (intervention) vs. no antibiotic (controls) in women in pre-term labour with intact membranes.	PMR: RR = 1.22 (95% CI: 0.88–1.70) **[NS]**

Lumbiganon et al. 2004 [142]	USA. Meta-analysis (Cochrane). 2 RCTs included.	Assessed the impact of chlorhexidine vaginal wash (intervention) vs. placebo (controls) in preventing maternal and neonatal infections including chorioamnionitis and sepsis.	PMR: RR = 1.00 (0.17–5.79) **[NS]**

Thinkhamrop et al. 2002 [146]	Kenya, Belgium, USA, The Netherlands. Meta-analysis (Cochrane). 3 RCTs included.	Assessed the impact of prophylactic antibiotic administration in the second and third trimester (intervention) vs. no antibiotic (controls), particularly in reference to women with prior pre-term birth and women with BV.	PMR: OR = 0.5 (95% CI: 0.16–1.71) **[NS]** PROM: OR = 0.32 (95% CI: 0.14–0.73) LBW: OR = 0.48 (95% CI: 0.27–0.84) *in women with a previous pre-term birth* Pre-term: OR = 1.06 (95% CI: 0.68–1.64) **[NS]** Pre-term: OR = 0.48 (95% CI: 0.28–0.81) *in women with confirmed BV*.

Goldenberg et al. 2006 [143]	Malawi, Egypt. Review. 2 non-randomised, non-blinded trials included. N = 11,380 women.	Assessed the impact of chlorhexidine vaginal wash (intervention) vs. placebo (controls) in preventing maternal and neonatal infections and neonatal death.	No pooled estimates given. ENND [Malawi]: RR = 0.78 [29/1000 vs 37/1000 in intervention vs. control groups, respectively], RR = 0.78) NM due to infections [Malawi]: OR = 0.5 (95% CI: 0.29–0.88, P < 0.005) [2.4 vs. 7.3 per 1000 in intervention vs. control groups, respectively]. Infant death [Egypt]: 2.8 vs. 4.2% in intervention vs. control groups, respectively (P = 0.01) Infant death due to infection: 0.22% vs. 0.84% in intervention vs. control groups, respectively (P = 0.004)

McDonald et al. 2007 [141]	Australia, UK, USA, South Africa. Meta-analysis (Cochrane). 5 RCTs included.	Assessed the impact of oral antibiotics (intervention) vs. placebo/no treatment (controls).	PMR: OR = 0.94 (95% CI: 0.52–1.70) **[NS]**

***Observational studies***			

Watson-Jones et al. 2007 [187] .	Tanzania (Mwanza). Prospective cohort study. Women (N = 1688) attending ANC.	As part of a study of the effectiveness of syphilis screening and treatment, assessed the impact of screening and treatment for reproductive tract infections (RTIs) during pregnancy on SB, IUGR, LBW, and pre-term birth.	SBR: 27/1000. No statistical significance data. Pre-term: 12% LBW: 8% SB risk factors: past history of stillbirth, short maternal stature and anaemia. No association between treated RTIs and adverse pregnancy outcomes.

Tita et al. 2007 [200]	USA (Alabama). RCT, subgroup analysis. Center for Women's Reproductive Health, University of Alabama at Birmingham. N = 241 nonpregnant women with reproductive tract infections; N = 124 conceived with birth outcome data (N = 59 intervention; N = 65 controls).	Compared impact of 2 doses of azithromycin 1.0 g given 4 days apart plus sustained-release metronidazole 750 mg daily for 7 days (intervention) vs. placebo (controls). Treatment was repeated 3x/yr until conception or until study termination. Reevaluation after randomisation with cultures and histopathology. Followed up 5 years.	Adverse pregnancy outcome (pre-term birth or fetal death): 66.1% (39/59) vs. 61.5% (40/65) **[NS] **in intervention vs. control groups, respectively. RR = 1.25 (99% CI: 0.42–3.7) **[NS] **in women colonised with any microbe at baseline vs. women without any colonisation. [62.7% vs 50%, respectively]. RR = 0.87 (0.50–1.5) **[NS] **in women with plasma cell endometritis vs. women without. [61.9% vs. 70.8%, respectively]. RR = 0.60 (95% CI = 0.3–1.2) **[NS] **in women without *Gardnerella vaginalis *colonisation vs. women with colonisation RR = 0.66 (95% CI = 0.4–1.2) **[NS] **in women without Gram-negative rod colonisation vs women with colonisation. RR = 1.5 (95% CI: 1.1–2.0) in intervention vs. control groups, respectively, in the presence of *Gardnerella vaginalis *(crossover interaction). RR = 1.5 (95% CI: 1.1–2.1) in intervention vs. control groups, respectively, in the presence of Gram-negative rod colonisation (crossover interaction).

A Cochrane review by Lumbiganon et al. [142] found that vaginal chlorhexidine washing had no effect on perinatal mortality (RR = 1.00, 95% CI: 0.17–5.79) due to maternal and neonatal infections (excluding GBS and HIV) compared to placebo ***[LOE: 1++] ***(Additional file 20). A systematic review by Goldenberg et al. [143] of the use of vaginal chlorhexidine treatment included two large, non-randomised, non-blinded trials from low-/middle-income countries that reported neonatal outcomes ***[LOE: 2+] ***(Additional file 21). One quasi-experimental trial (N = 6965 women) included in this review from Malawi by Taha et al. [144], found that 0.25% chlorhexidine wipes at each vaginal examination during labour, followed by a neonatal wipe of the same concentration soon after birth, was associated with significant reductions in early neonatal deaths (29/1000 vs 37/1000 in intervention vs. control groups, respectively, RR = 0.78) and neonatal mortality due to infections (OR = 0.5, CI 0.29–0.88, 2.4 versus 7.3 per 1000, *P *< 0.005). A trial in Egypt included in the Goldenberg review using an almost identical study design and treatment plan as the Malawi study [145] (N = 4415 women), found significant decreases in infant death (2.8 vs. 4.2% in intervention vs. control groups, respectively, P = 0.01), and infant death due to infection (0.22% vs. 0.84% in intervention vs. control groups, respectively, P = 0.004). Neither study reported intrapartum stillbirth rates, which chlorhexidine treatment could theoretically impact by reducing infectious causes.

Thinkhamrop et al. [146] conducted a Cochrane review of the effects of prophylactic antibiotic administration in the second and third trimester (N = 6 RCTs, N = 2184 women) (Additional file 22). Antibiotic prophylaxis clearly reduced the risk of PROM (OR = 0.32, 95% CI: 0.14–0.73). Among women with a previous pre-term birth, antibiotics were associated with reduced odds of LBW (OR = 0.48, 95% CI: 0.27–0.84), as well as pre-term delivery (OR = 0.48, 95% CI: 0.28–0.81) in the subgroup of women with BV. Women with previous pre-term birth but without BV had no reduction in odds of pre-term birth (OR = 1.06, 95% CI: 0.68–1.64). The 3 trials that reported PMR yielded only non-significant effects (OR = 0.52, 95% CI: 0.16–1.71). ***[LOE: 1++]***.

##### Conclusion

The evidence to date does not suggest any benefit in screening and treating all pregnant women for asymptomatic BV to prevent pre-term birth (Grade D evidence); however, there is limited evidence that treatment with antibiotics early in pregnancy, particularly for women with confirmed intermediate flora or BV, may reduce pre-term births. The results of treatment trials for pregnant women with BV have been mixed, ranging from an 80% reduction to a two-fold increase in pre-term birth among women who received treatment, despite large numbers of women in the included trials. This heterogeneity of effect may be attributable to differences in participants' genetic background, diagnosis criteria, timing of treatment and antibiotic choice.

Chlorhexidine washes have long been linked to decreased maternal positivity for GBS [147], but this intervention also appears effective against other organisms. There is highly promising evidence from Egypt and Malawi to support the use of vaginal chlorhexidine washing during labour (and of the neonate after birth) in preventing adverse maternal and neonatal outcomes associated with infection, including neonatal death from sepsis and overall neonatal mortality, but stillbirths were not reported by studies to date. Two RCTs of maternal vaginal plus newborn skin cleansing have just been completed in South Africa and in Pakistan, but results of these trials have not yet been reported.

Although it appears that antibiotic prophylaxis during the second or third trimester of pregnancy may reduce the risk of PROM, LBW, and transmission of infection to the neonate in high-risk pregnant women, more data are needed to assess the risks of routine use of antibiotics in pregnancy and to confirm whether their use can prevent these and other substantive adverse pregnancy outcomes.

#### Antibiotics for pre-term premature rupture of membranes (PPROM) and premature rupture of membranes (PROM) in high-risk pregnancies

##### Background

Premature rupture of membranes (PROM) is a condition where the placental membranes rupture at term prior to the onset of labour, which is of clinical concern if labour does not quickly become established thereafter, as the risk of maternal and fetal infection mounts. A related problem is when membranes rupture prior to 37 weeks (term). This condition precedes one-third of pre-term births, and has proven difficult to prevent: despite socio-economic improvements and the development of a large range of therapeutic interventions in high-income countries, the rate of pre-term births remains at 6 to 8 percent.

The causes of PROM and PPROM are multifactorial. Infection appears to play a major role, either as a cause or as a consequence of PROM/PPROM. Some organisms may produce collagenases, mucinases and proteases, which weaken the amnion and chorion and may lead to membrane rupture. Infection may also occur secondary to membrane rupture if ascending infection leads to occult deciduitis, intra-amniotic infection or fetal infection. A possible mechanism for the link between infection and pre-term delivery is bacterial stimulation of the biosynthesis of prostaglandins, either directly via phospholipase A2 and C [148], or indirectly via substances such as interleukin-1, tumour necrosis factor and platelet activating factor, all of which have been identified in infected amniotic fluid [149].

Theoretically, antibiotic therapy could improve perinatal outcome by two processes. Prevention or treatment of infection may minimise maternal or fetal infectious morbidity, which would have a direct impact on stillbirth incidence. Secondly, in cases of PPROM, suppression of the prostaglandin response may slow the progression to pre-term birth described above, which could provide a window of time for fetal lung maturation and other fetal changes that could impact incidence of complications of prematurity.

##### Literature-based evidence

The literature search identified two Cochrane reviews, and one other intervention study (Table 13). Kenyon et al. [150] assessed the effects of administering antibiotics to women with PROM on fetal and neonatal morbidity and mortality, including both trials that compared antibiotic to placebo, as well as trials without placebo (22 trials, N = 6000 women) (Additional file 23). Antibiotic administration following PROM was associated with a statistically significant reduction in chorioamnionitis (RR = 0.57, 95% CI: 0.37–0.86), births <48 hours after membrane rupture (RR = 0.71, 95% CI: 0.58–0.87), and births < 7 days after randomisation (RR = 0.80, 95% CI: 0.71–0.90). There was no apparent impact on perinatal mortality or death before discharge (13 trials; RR = 0.90, 95% CI: 0.74–1.10) ***[LOE: 1++]***.

**Table 13:** Impact of antibiotics for PPROM/PROM on stillbirth and perinatal outcomes

**Source**	**Location and Type of Study**	**Intervention**	**Stillbirths/Perinatal Outcomes**
***Reviews and meta-analyses***			

Flenady and King 2002 [151] .	Spain. Meta-analysis (Cochrane). 2 RCTs included (N = 838 women).	Compared the impact of any antibiotic (intervention) vs. no antibiotic (controls) in cases of PROM ≥ 36 wks' gestation.	PMR: RR = 0.98 (95% CI: 0.14 – 6.89) **[NS]** [2/426 vs. 2/412 in intervention vs. control groups, respectively].

Kenyon et al. 2003 [150]	Mozambique, Norway, Sweden, USA, Spain, UK, Finland, Chile, Denmark. Meta-analysis (Cochrane). 19 RCTs included (N = 6411).	Compared the impact of any antibiotic (intervention) vs. placebo (controls) in cases of PPROM.	PMR/death before hospital discharge: RR = 0.90 (95% CI: 0.74–1.10) **[NS] **[data from 13 RCTs]. [281/4374 vs. 148/2037 in intervention vs. control groups, respectively].

***Intervention studies***			

Kenyon et al. 2002 [201]	UK and other international sites. Multicentre. RCT. Pregnant women (N = 4826) with PPROM.	Assessed the effects of co-amoxiclav and erythromycin singly and in combination: 325 mg co-amoxiclav plus 250 mg erythromycin (group 1), co-amoxiclav plus erythromycin placebo (group 2), erythromycin plus co-amoxiclav placebo (group 3), or co-amoxiclav placebo plus erythromycin placebo (group 4). Antibiotics given 4× daily for 10 d or until delivery.	NMR: 5.2% vs. 5.7% vs. 6.2% in erythromycin (group 3), co-amoxiclav (group 2) and placebo (group 4), respectively. Major neonatal cerebral abnormality lower with erythromycin vs. placebo or co-amoxiclav.

Another Cochrane review by Flenady and King [151] examined trials that tested the impact on maternal, fetal, and neonatal outcomes of antibiotics administered prophylactically to women with PROM ≥ 36 weeks' gestation (2 trials, N = 838 women) (Additional file 24). Antibiotic use was significantly associated with a decreased incidence of maternal infectious morbidity (chorioamnionitis or endometritis) (RR = 0.43, 95% CI: 0.23–0.82), RD = (-)4%, 95% CI: (-)7%, (-)1%), number needed to treat [NNT] = 25, 95% CI: 14–100). The two studies reporting perinatal outcomes showed no impact of antibiotic treatment on PMR (RR = 0.98, 95% CI: 0.14–6.89) ***[LOE: 1+]***.

##### Conclusion

Although the two Cochrane reviews on the subject were given a Grade B rating, they report no benefit of antibiotic administration in cases of PROM to reduce stillbirths. Antibiotic treatment following PPROM was associated with a statistically significant delay in women giving birth and reductions in major markers of neonatal morbidity (although not perinatal mortality). This delay in delivery would allow sufficient time for prophylactic prenatal corticosteroids to take effect. These data support the routine use of antibiotics in this clinical situation. The increase in the numbers of babies who developed neonatal necrotising enterocolitis with prenatal co-amoxiclav treatment suggests that erythromycin rather than co-amoxiclav is the antibiotic of choice in women at risk of pre-term delivery [150]. In cases of term PROM, the Cochrane review by Flenady and King [151] indicates that there is insufficient evidence to justify the routine use of antibiotics prior to the onset of labour. Although antibiotics reduced rates of chorioamnionitis and endometritis, the low rate of maternal infection in the control population (~7 percent) suggests that exposure of all women with term PROM to antibiotics is unwarranted if treatment can be restricted to those who develop clinical indications for antibiotic treatment. Because these studies do show that antibiotics have a statistically significant impact on chorioamnionitis, which is a known risk factor for stillbirth, more research is required to evaluate the potential impact of antibiotic administration after membrane rupture.

#### Anti-malarials in malaria-endemic areas

##### Background

Globally, malaria affects almost 10% of the world's population, and of the nearly 500 million annual cases, approximately 1 million people die annually [152]. Particularly in areas where malaria is endemic, malaria is a key cause of maternal illness and anaemia in pregnancy, especially in primiparas [153,154]. Administration of anti-malarial drugs, whether presumptively through strategies such as intermittent preventive treatment (IPT) or as treatment for diagnosed parasitemia in pregnancy, is generally recommended in areas endemic for malaria.

##### Literature-based evidence

The literature review identified one Cochrane review comprised of nine RCTs, as well as two other intervention and observational studies (Table 14). In the Cochrane review (N = 16 RCTs, N = 12,638 women), Garner and Gülmezoglu [155] found that anti-malarials reduced antenatal parasitemia when given to all pregnant women, but had no impact on PMR (RR = 1.02, 95% CI: 0.73–1.43, 4 RCTs, N = 2890 participants) or stillbirths specifically (RR = 1.51, 95% CI: 0.80–2.84) (Additional file 25). However, among women in their first or second pregnancy, a strategy of IPT reduced the risk of perinatal mortality (RR = 0.73, 95% CI: 0.53–0.99; 1 RCT, N = 1986 participants) but not stillbirth (RR = 0.87, 95% CI: 0.62–1.21, N = 3454 participants) ***[LOE: 1+]***.

**Table 14:** Impact of anti-malarials in pregnancy in malaria-endemic areas on stillbirth and perinatal mortality

**Source**	**Location and Type of Study**	**Intervention**	**Stillbirths/Perinatal Outcomes**
***Reviews and meta-analyses***			

Garner et al. 2006 [155]	Burkina Faso, Cameroon, Gambia, Nigeria, Uganda, Thailand, Kenya. Meta-analysis (Cochrane). 9 RCTs included.	Assessed 1) the impact of treating malaria with any anti-malarial drug (intervention #1) vs. no drug (control #1), and 2) preventing malaria with any anti-malarial drug (intervention #2) vs. no drug (control #2).	PMR (treatment): RR = 1.02 (95% CI: 0.73–1.43) **[NS] **in intervention group #1 vs. control group #1, respectively. PMR (prevention): RR = 0.73 (95% CI: 0.53–0.99) in intervention group #2 vs. control group #2, respectively.

***Intervention studies***			

Hamer et al. 2007 [156]	Zambia (Ndola), urban setting. Cluster RCT. N = 456 HIV-positive mothers (N = 224 intervention group, N = 232 controls).	Compared 2 dosing schedules for malaria prevention: 1 treatment course of SP per month (intervention) vs. 1 course of SP per trimester (controls).	SBR: RR = 0.43 (95% CI: 0.1–2.2) **[NS]** [2/191 vs. 5/203 in intervention vs. control groups, respectively.]

***Observational studies***			

Verhoeff et al. 1999 [202]	Malawi (Chikwawa district), rural setting. Prospective cohort study. N = 1523 women.	Assessed the impact of antenatal screening and treatment for malaria and anaemia.	SBR: 3.7% ENND (< 48 h postpartum): 1.7%

An additional double-blind, placebo-controlled RCT not included in the reviews on malaria treatment during pregnancy in endemic areas was conducted by Hamer et al. [156] in which HIV-positive mothers were randomised in blocks of 20 to one of two dosing schedules: one treatment course of sulfadoxine-pyrimethamine (SP) per month; or one course of SP per trimester. There were slightly more stillbirths among the group receiving monthly SP compared to the once-per-trimester group, but this difference was not significant (2/191 vs. 5/203 total deliveries; RR = 0.43, 95% CI: 0.1–2.2) ***[LOE: 1+]***.

##### Conclusion

Prophylaxis with a variety of anti-malarial drugs or IPT with SP in women having their first or second baby is associated with a 38% lower incidence of severe anaemia and 27% fewer perinatal deaths, according to the Cochrane review on IPT and chemoprophylaxis [155] (Grade B evidence). There was no obvious impact on anaemia or perinatal outcomes when prophylaxis or IPT was given to all women regardless of the number of previous pregnancies. Malaria parasitaemia in the blood or the placenta is also less common with prophylaxis or IPT.

This evidence relates to a reasonable number of good quality studies although the data show no significant evidence of direct impact on stillbirths, largely owing to the studies being underpowered to detect differences. However, the myriad maternal and perinatal benefits observed justify the use of malaria chemoprophylaxis in women of low parity in malaria endemic regions. SP is currently the first-line drug for IPT in many countries according to the WHO Treatment Guidelines [157], but patterns of drug resistance are emerging rapidly. Although the WHO guidelines now recommend artesunate for most parasitemias, many new treatment drugs and regimens are available for the treatment of malaria; however, little information is available about their pharmacokinetics, safety, and effectiveness in pregnant women, as trials have excluded these women for fear of embryotoxicity [158]. More research is underway to expand the arsenal of anti-malarial drugs, potentially to include other artemisinin-based treatments than artesunate, which are also safe to use in pregnancy [159].

#### Use of insecticide-treated nets (ITNs) during pregnancy

##### Background

Every year, an estimated 50 million pregnant women are exposed to malaria. Pregnancy renders women more susceptible to malaria, and both the mother and fetus are at risk of adverse consequences of malarial infection, including maternal death from malaria, maternal anaemia and LBW in the fetus [160]. Although the use of insecticide-treated nets (ITNs) during pregnancy has been touted as a key strategy for preventing malaria in pregnancy, the evidence of benefit has been mixed [161]. The use of ITNs is known to decrease maternal and placental parasitemia, and raise haemoglobin concentrations in women with their first to fourth pregnancies [161]. ITNs are recommended by the WHO in conjunction with IPT and case management of malarial infection [162] to prevent malaria in pregnant women living in malaria-endemic areas of Africa and Asia.

##### Literature-based evidence

One systematic review, published in two journals and comprised of 5 RCTs, was identified in our literature search (Table 15). This review by Gamble et al. [161] assessed ITN use during pregnancy (N = 5 RCTs, with cluster- and individual-randomised designs) (Additional file 26). Four trials from sub-Saharan Africa compared the use of ITNs with no nets, and one trial from Asia compared ITNs with untreated nets. In Africa, ITNs reduced placental malaria in all pregnancies compared with no nets (RR = 0.79, 95% CI: 0.63–0.98). The use of ITNs also reduced LBW incidence (RR = 0.77, 95% CI: 0.61–0.98) and fetal loss (miscarriages plus stillbirths) in the first to fourth pregnancy (RR = 0.67, 95% CI: 0.47–0.97), but not in fifth and higher-order pregnancies. ITNs also appeared to have a beneficial impact on anaemia and clinical malaria but these trends were not statistically significant ***[LOE: 1++]***. The updated systematic review by the same author in 2007 mentions four trials in which stillbirth outcomes are reported (Additional file 26). Summary estimates for the effect of ITN use were estimated from 3 studies [163-165] reporting fetal death (miscarriage plus stillbirths). ITNs compared to no nets reduced fetal death (by 33% (RR = 0.67, 95% CI: 0.47–0.97) in the first or second pregnancy ***[LOE: 1++]***.

**Table 15:** Impact of ITNs on stillbirth and perinatal mortality

**Source**	**Location and Type of Study**	**Intervention**	**Stillbirths/Perinatal Outcomes**
***Reviews and meta-analyses***			

Gamble et al. 2006 Gamble et al. 2007 [161,203]	Kenya. Meta-analysis (Cochrane). 5 RCTs included.	To compare the impact of ITNs (permethrin 500 g/m^2 ^except in one trial that used cyfluthrin; intervention) vs. untreated nets or no nets (control) in preventing malaria in pregnancy. All African trials gave double- or family-sized nets to each household, vs. single-sized nets in Thailand.	Fetal death (3 RCTs): RR = 0.68 (95% CI: 0.48–0.98, P = 0.04) in all gravidae RR = 0.67 (95% CI: 0.47–0.97, P = 0.03) in low gravidae (1–2 pregnancies) RR = 1.02 (95% CI: 0.17–6.23, P = 0.98) [NS] in high gravidae (> 4 pregnancies) Birth weight: Mean increase: 50 g (95% CI: 20–90 g) in women with 1–2 prior pregnancies. LBW (1 RCT): RR = 0.77, 95% CI: 0.61–0.98) in women with 1–2 prior pregnancies Placental malaria: RR = 0.79, 95% CI: 0.63–0.98). Fetal death (with untreated nets as control group, 1 RCT): RR = 0.21 (95% CI: 0.05–0.92) in all gravidae [2/102 vs. 10/97 in intervention vs. control groups, respectively.]

##### Conclusion

The available studies and Cochrane review provide strong evidence of benefit of ITN use in pregnancy (Grade A evidence). ITNs significantly decreased maternal and placental parasitaemia, increased mean birth weight in most trials, and reduced rates of fetal loss and other negative pregnancy outcomes in women in their first or second pregnancy, with some benefit likely through the fourth pregnancy. Thus, ITNs are recommended for pregnant women, particularly primigravidae, in malaria-endemic areas. As four of the five trials considered were conducted in Africa, and the only trial conducted in Asia compared ITNs with untreated nets on the Thai-Burmese border, further efficacy studies of ITNs to prevent malaria in pregnancy are recommended in areas with less intense (as well as *Plasmodium vivax*) transmission in Asia and Latin America.

#### Prevention of mother-to-child transmission of HIV

##### Background

High maternal viral load, which measures the level of HIV RNA in the plasma, is a strong independent risk factor for vertical (mother-to-child) transmission of HIV. In addition, breastfeeding, young maternal age, other STDs, advanced maternal HIV disease, low CD4 cell count, chorioamnionitis, prolonged rupture of membranes, vaginal delivery and associated events increasing maternal bleeding, and history of stillbirth also increase the risk of vertical transmission [166]. Anti-retroviral (ARV) drugs, primarily zidovudine, nevirapine, or a combination of the two, are recommended during pregnancy to reduce mother-to-child transmission of HIV because by reducing viral replication, they effectively reduce maternal viral load. Additionally, the drugs provide some measure of pre-exposure prophylaxis for the fetus because they cross the placenta, and can be administered directly to the baby after delivery (usually within 72 hours of birth) as post-exposure prophylaxis. In developed countries, rates are around 1–2% thanks to the availability of highly active ARV therapy (HAART) [166]. HAART availability remains limited in low and middle-income countries, but simpler and less expensive ARVs are now widely available. While numerous studies have been conducted on the efficacy of ARVs for PMTCT, few have reported stillbirth outcomes.

##### Literature-based evidence

We identified three systematic reviews (3 reviews and meta-analyses comprised of 18 RCTs) and 8 other studies (Table 16). The studies assessed a range of different ARV drug choices, dosages, and timing of administration to mother, infant, or both. In a Cochrane review by Volmink et al. comparing ARVs administered to mothers and their infants to placebo [166], all 6 eligible RCTs reported statistically non-significant reductions in stillbirths (results were not pooled: see Table 16 for RRs and CIs) (Additional file 27). Comparing longer vs. shorter ARV regimens, 3 RCTs showed statistically non-significant reductions in stillbirths for longer regimens (results were not pooled, see Table 16 for RRs and CIs) ***[LOE: 1++]***. Another systematic review by Suksomboon et al. [167] supported the efficacy of zidovudine to prevent vertical HIV transmission (RR = 0.57, 95% CI: 0.45–0.71 in zidovudine vs. placebo groups, respectively) as well as to reduce LBW (Pooled RR = 0.75, 95% CI: 0.57–0.99, P = 0.039), but found no significant effects of zidovudine on stillbirth or pre-term delivery (Additional file 28). The Suksomboon et al. review also found no differences between short-long and long-long regimens of zidovudine therapy in rates of perinatal HIV transmission, infant death, stillbirth, or pre-term birth. However, in the African studies in this review, zidovudine plus lamivudine combination therapy was very effective in preventing vertical transmission (adj. OR = 0.23, P < 0.0001). One included trial found that 2-dose nevirapine prophylaxis (intrapartum plus newborn dose, given in combination with zidovudine) reduced the death rate of live-born babies by 80% (RR = 0.20, 95% CI: 0.05–0.90) versus nevirapine placebo plus zidovudine; however, this regimen had no impact on SBR (RR = 1.11, 95% CI: 0.48–2.56) ***[LOE: 1++]***.

**Table 16:** Impact of PMTCT on stillbirth and perinatal mortality

**Source**	**Location and Type of Trial**	**Intervention**	**Stillbirths/Perinatal Outcomes**
**Reviews and meta-analyses**			

Suksomboon et al. 2007 [167]	USA, France, Côte d'Ivoire, Burkina Faso, Thailand, Bahamas, Brazil. Meta-analysis (Cochrane). 15 trials, 5 reporting SBR.	Assessed a variety of regimens and dosing schedules for PMTCT for efficacy in preventing vertical transmission, infant death, and adverse pregnancy outcomes.	***Zidovudine vs. placebo: *** Vertical transmission: Pooled RR = 0.57 (95% CI: 0.45–0.71) LBW: Pooled RR = 0.75 (95% CI: 0.57–0.99, P = 0.039) SBR: **[NS]** Pre-term: **[NS]** ***2-dose maternal+infant nevirapine therapy vs. nevirapine placebo (both given zidovudine) ***[1 RCT]: Infant death: RR = 0.20 (95% CI: 0.05–0.90) SBR: RR = 1.11 (95% CI: 0.48–2.56) **[NS]** [10/826 vs.9/835 in intervention vs. control groups, respectively (pooled)].

Volmink et al. 2007 [166]	Thailand, Côte d'Ivoire, Burkina Faso, Uganda, Kenya, USA, France, Brazil, Bahamas, Tanzania, Zimbabwe. Meta-analysis (Cochrane). 13 RCTs included that reported stillbirth rates.	Assessed the impact of zidovudine alone (intervention #1), nevirapine alone (intervention #2), and combination zidovudine-nevirapine therapy (intervention #3) vs. placebo (controls) on MTCT. Subgroups also analyzed according to breastfeeding.	Pooled analysis not given for SBR. ***ARV vs. placebo:*** Vertical transmission: RR = 0.46 (95% CI: 0.35–0.60) SBR: RR = 0.14 (95% CI: 0.02–1.17); RR = 0.33 (95% CI: 0.01–8.11); RR = 0.40 (95% CI: 0.07–2.15); RR = 0.80 (95% CI: 0.20–3.18); RR = 3.02 (95% CI: 0.12–73.57); RR = 3.50 (95% CI: 0.74–16.55) for the 6 trials, respectively. ***Long vs. short-course zidovudine:*** SBR: RR = 0.25, 95% CI: 0.05–1.17; RR = 0.33, 95% CI: 0.11–1.01; RR = 0.55, 95% CI: 0.23–1.33, for the 3 trials, respectively

Wiysonge et al. 2005 [173]	Tanzania, Zimbabwe, South Africa, and Malawi. Meta-analysis (Cochrane). 4 RCTs included (N = 2855 participants).	Assessed the impact of vitamin A supplementation (intervention) vs. placebo (controls) on MTCT.	SBR: OR = 0.99 (95% CI: 0.67–1.46) **[NS]**

***Intervention studies***			

Bussmann et al. 2007 [169]	Botswana. RCT.	Assessed the impact of 6 HAART regimens, 3 of which contained efavirenz (intervention), vs. non-efavirenz regimens (controls).	SBR: No difference between efavirenz and non-efavirenz-exposed pregnancies (P = 0.7).

Sharma et al. 2007 [172]	USA (New York). Prospective cohort study (before-after design). Women's Interagency HIV study, data collected pre-HAART (1994–95) and during HAART (2001–02).	Assessed the impact of HAART on live birth rates among HIV-positive women (intervention) compared to HIV-negative women (comparison).	Live birth rate: 150% vs. 5% higher in intervention vs. comparison groups after introduction of HAART (P = 0.001).

Sperling et al. 1996 [204]	USA. RCT. Mother-infant pairs (N = 402).	Compared the impact of maternal zidovudine treatment (intervention) vs. placebo (controls).	Rate of HIV-1 transmission: 7.6% (95% CI: 4.3–12.3%) vs. 22.6% (95% CI: 17.0–29.0%) in intervention vs. control groups, respectively (P < 0.001). Transmission occurred at a wide range of maternal plasma HIV-1 RNA levels. No SBR reported.

Stiehm et al. 1999 [205]	USA, Puerto Rico. RCT. Women (N = 501) at 53 centers.	Assessed the impact of HIV immunoglobulin 200 mg/kg IV infusion every 4 wks beginning between 20 – 30 wks gestation and during delivery plus 200 mg/kg IV infusion to baby within 12 hours of birth, plus maternal+infant zidovudine standard course (intervention) vs. standard polyvalent HIV antibody-negative IVIG to mother and baby as above, plus maternal+infant zidovudine standard course (controls).	SBR: RR = 0.33 (95% CI: 0.01–8.03) **[NS]** [0/231 vs. 1/228 in intervention vs. control groups, respectively.]

Tonwe-Gold et al. 2007 [171]	Cote d'Ivoire (Abidjan). Observational cohort study.	Assessed the impact of HAART vs. short-course anti-retroviral (scARV) PMTCT regimens to which women were allocated according to their clinical and immunological status.	SBR: **[NS]** [4 (3.9%) vs. 6 (4.3%) in HAART vs. scARV for PMTCT groups, respectively (P = 1.00)].

Townsend et al. 2007 [170]	UK, Ireland. Retrospective study. Pregnancies in women notified to the National Study of HIV in Pregnancy and Childhood (NSHPC).	Compared women on HAART (intervention) to women on mono/dual therapy (controls).	SBR: adj. OR = 2.27 (95% CI: 0.96–5.41; P = 0.063) **[NS]** [12.7/1000 births (43/3384) vs. 5.7/1000 (6/1061) in HAART vs. mono/dual therapy groups].

Tuomala et al. 2002 [168]	USA (Miami, Florida & Southern California). Case control study. 2 multisite studies + 3 single site studies.	Compared impact of combination ART (cases) vs. no ART (controls)	SBR: 12/2123 (1%) vs.7/1143 (1%) in intervention vs. control groups, respectively. Adjusted rate (for CD4, tobacco, alcohol, illicit drug use): 1% (P = 0.92) **[NS]**

Onah et al. 2007 [206]	Nigeria (Enugu). Retrospective case-control study. Pregnant women (N = 162; N = 62 HIV-positive women, N = 100 HIV-negative controls) delivering in the University of Nigeria Teaching Hospital from 2002–2004.	Compared incidence of stillbirth in untreated HIV-positive women (cases) vs. HIV-negative women (controls).	SBR: No difference (P > 0.05). 4.8/1000 vs. 1.0/1000 in cases vs. controls, respectively. Maternal and fetal morbidities: higher in HIV-positive group.

Similarly, comparing HIV-1-infected pregnant women given monotherapy, combination therapy without protease inhibitors, and combination therapy with protease inhibitors to women who did not receive ARVs, Tuomala et al. [168] reported similar SBRs between all groups ***[LOE: 2+]***. Efavirenz therapy also appears to be ineffective in preventing stillbirths; in a comparison of 6 treatment regimens, 3 of which contained efavirenz, Bussmann et al. [169] reported no impact of efavirenz on SBR (P = 0.7) ***[LOE: 1-]***.

HAART, the administration strategy of choice for HIV disease where available, is associated with improved maternal and infant outcomes, but does not appear to impact stillbirth incidence. Townsend et al. [170] compared women on HAART with women on mono/dual therapy. In comparison with exposure to mono/dual therapy, exposure to HAART was associated with a non-significant increased risk of stillbirth (adj. OR = 2.27, 95% CI: 0.96–5.41; P = 0.063) ***[LOE 2+]***; this trend was not observed by Tonwe-Gold et al. [171], who found that compared to women on short-course anti-retroviral (scARV) PMTCT regimens, women on HAART had no difference in SBR (P = 1.00) ***[LOE: 2+]***. Sharma et al. [172] found that the total live birth rate increased by 150% (*P *= 0.001) in HIV-positive women given HAART compared to the pre-HAART era, compared with a mere 5% increase during the same time period in HIV uninfected women, but the study design was unable to determine stillbirth rates before or during HAART administration ***[LOE: 3]***.

A third PMTCT-related Cochrane review by Wiysonge et al. [173] reviewed the potential for vitamin A supplementation to reduce vertical transmission of HIV infection (4 RCTs, N = 3033 HIV-positive pregnant women), but found no evidence of impact of vitamin A supplementation on stillbirths (OR = 0.99, 95% CI: 0.67–1.46) ***[LOE: 1++] ***(Additional file 29).

##### Conclusion

Overall, while there is no consensus on the ideal regimen for preventing vertical transmission, there is considerable evidence that in resource-poor settings, zidovudine alone or in combination with other ARVs such as lamivudine or nevirapine reduces the risk of vertical transmission and infant death, as well as positively impacts birth weight (overall Grade C evidence). In mothers already receiving zidovudine prophylaxis, a single dose of nevirapine to mothers during labour and to infants after birth further decreases the risk of vertical transmission and infant death. Wherever available, HAART appears to be associated with statistically significant improvement in live birth rate. However, there is no evidence that any regimen of PMTCT impacts stillbirth incidence. Additionally, there is no evidence for any impact of vitamin A supplementation on stillbirths in HIV-positive mothers. As PMTCT regimens are of clear benefit to maternal health (when administered to pregnant women) and infants (when administered during pregnancy and the postnatal period), we recommend their inclusion in ANC programs based on these benefits alone, with the caveat that there is no evidence of their efficacy in preventing stillbirths.

#### Periodontal care during pregnancy

##### Background

Periodontal disease, including gingivitis and periodontitis, is one of the most common chronic disorders of infectious origin known in humans, with a reported prevalence varying between 10 and 60% in adults, depending on diagnostic criteria. Periodontal disease is initiated by overgrowth of certain bacterial species, with a majority of Gram-negative, anaerobic bacteria growing in subgingival sites and producing lipopolysaccharide (LPS) endotoxins and other bacterial substances. The host response to periodontal pathogens causes persistent inflammation with high levels of proinflammatory cytokines, contributing locally to periodontal disease as well as to systemic effects including increased risk of atherosclerosis, myocardial infarction, stroke, diabetes mellitus. Several studies have highlighted the relationship between maternal periodontal disease and birth outcomes, including prematurity [174,175]. A large number of studies have explored the mechanisms by which periodontal disease might impact LBW, miscarriage, and pre-eclampsia [176]. Most of these studies have centered on prematurity, and although several RCTs have reported an association between periodontal care and reduced rates of pre-term birth or pre-term LBW, systematic reviews and meta-analyses on the subject have been unable to determine conclusively whether there are any associations with these outcomes [177]. There has been much less attention to date as to whether periodontal care could prevent stillbirth.

##### Literature-based evidence

Our literature search identified 2 systematic reviews, and 4 other observational/intervention studies, that reported stillbirth or perinatal mortality outcomes (Table 17). None of the systematic reviews reported a conclusive association between periodontal disease and stillbirth or perinatal mortality. In a systematic review by Xiong et al. [176], only one cohort study [178] reported stillbirth or miscarriage as an outcome. Regression analysis revealed no significant relationships between the severity of periodontal disease and pre-term birth or LBW, but did elucidate a significant correlation between poorer periodontal health and those that experienced a late miscarriage (adj. OR = 2.54, 95% CI: 1.20–5.39) (Additional file 30). An update of this analysis [179] which included 2 studies reported an effect size ranging from 2.54–3.84 times increased risk of fetal loss attributable to periodontal disease (Additional file 31). Tarannum et al. [180] also reported a convincing link between periodontal disease and adverse pregnancy outcome (OR = 5.5, 95% CI: 1.4–21.2; P = 0.014). A more recent prospective cohort study from Pakistan [181] involving a periodontal examination at 20–26 weeks' gestation for study subjects (N = 1152 women) found that 76% of participants had moderate to severe periodontal disease. Stillbirth rates tended to be higher in the fourth quartile versus the first quartile of severity of periodontal disease (for stillbirth: 19/1000 vs. 41/1000 in 1st vs. 4th quartiles, respectively, P = 0.069). The same was true for perinatal and neonatal mortality rates. While some evidence supported an association between periodontal disease and early pre-term birth, the association was not statistically significant. Late pre-term birth and LBW were not associated with measures of periodontal disease.

**Table 17:** Impact of interventions for periodontal disease on stillbirth and perinatal mortality

**Source**	**Location and Type of Study**	**Intervention**	**Stillbirths/Perinatal Outcomes**
***Reviews and meta-analyses***			

Xiong et al. 2007 [179]	UK. Review. 2 cohort studies included.	Assessed the association of periodontal disease with fetal death.	Fetal death (miscarriage or SB): Effect size ranged from 2.54–3.84.

Xiong et al. 2006 [176]	UK. Review. 1 cohort study included (N = 3738 participants).	Assessed any association between periodontal disease and adverse pregnancy outcome.	Fetal death (Miscarriage+SB): adj OR = 2.54 (95% CI: 1.20–5.39)

***Intervention studies***			

Michalowicz et al. 2006 [184]	USA. RCT. Women (N = 823 women; N = 413 intervention, N = 410 controls) at 13–17 wks' gestation	Used competing-risks analysis to assess the impact of scaling and root planing before 21 wks' gestation, plus monthly tooth polishing and oral hygiene instruction (intervention) vs. scaling and root planing after delivery (controls).	SBR (20–37 wks): [3/413 vs. 10/410 in intervention vs. control groups, respectively, P = 0.04 **[NS]**] Pre-term: P = 0.51 **[NS]** Fetal death (miscarriage+SB): [5/413 vs. 14/410 in intervention vs. control groups, respectively, P = 0.08].

Macones et al. 2008 [182]	USA. RCT. Multicentre. Women (N = 757; N = 378 intervention, N = 379 controls) < 20 weeks' gestation with periodontal disease identified through screening.	Assessed the impact of periodontal care (scaling and root planing; intervention) vs. tooth polishing (controls) on pre-term birth and its complications.	Major neonatal morbidity/mortality: RR = 1.30 (95% CI; 0.83–2.03) **[NS]** [10.6% vs. 8.2% in intervention vs. control groups, respectively] Pre-term (< 35 wks): RR = 1.55 (95% CI: 0.90–2.67) **[NS]** [8.6% vs. 5.6% in intervention vs. control groups, respectively]

***Observational studies***			

Oittinen et al. 2005 [207]	Finland. Observational study. Women who became pregnant (N = 130) out of a total cohort of women who had discontinued contraception in order to become pregnant.	Assessed the association between maternal disease status, including periodontal disease and BV, with and adverse pregnancy outcome.	Adverse pregnancy outcome (pre-term birth or fetal death): OR = 5.5 (95% CI: 1.4–21.2, P = 0.014). [26/130 (20%) of sample. Univariate analysis also showed significant association of periodontal disease with adverse outcome (P = 0.012)]. Fetal death (miscarriage+SB): 17/130. Pre-term: N = 9.

Mobeen et al. 2008 [181]	Pakistan. Community setting. Prospective cohort study. Pregnant women enrolled at 20–26 weeks gestation and given dental exam, then followed until delivery.	Assessed the association between maternal periodontal disease severity with adverse pregnancy outcomes.	SBR: 19/1000 vs. 41/1000 (P = 0.069) between the first and the fourth periodontal quartiles (least severe disease vs. most severe disease). 26/1000 vs. 42/1000 (P = 0.131 **[NS]**) with increasing severity of the clinical attachment measures. 22/1000 vs. 50/1000 stillbirths (P = .033) with increasing severity of plaque index measures, 24/1000 vs. 51/1000 (P = 0.019) with increasing severity of the gingival index measures.

While published data convincingly demonstrate a linkage between periodontal disease and stillbirth incidence, the limited literature on interventions for periodontal disease has not yet shown any evidence of impact on pre-term birth or perinatal mortality. An RCT by Macones et al. [182], which provided scaling and root planing to an intervention group versus tooth polishing for controls, reported no impact of periodontal disease treatment on pre-term birth rate at less than 35 weeks gestation (RR = 1.55, 95% CI: 0.90–2.67), and no difference in rates of the composite outcome of major neonatal mortality or morbidity between the intervention and control groups (RR = 1.30, 95% CI: 0.83–2.03) [183]. In a competing-risks analysis by Michalowicz et al. [184], neither the risk of pre-term birth (P = 0.51) nor of spontaneous abortion or stillbirth (P = 0.08) differed significantly between women who were given periodontal care (scaling and root planing) at 21 weeks' gestation versus women whose care was delayed until after they gave birth.

##### Conclusion

There appears to be a relatively consistent association between periodontal disease and risk of adverse pregnancy outcome in some populations [174]. Many studies have explored the potential association of periodontal disease and periodontal care with pre-term birth or pre-term LBW, but the evidence is conflicting. The observational studies that reported statistically significant results lack the design strength to eliminate confounding and definitively attribute causation. Variability in definition of periodontal disease, as well as design flaws in published studies, suggest that the available findings should be viewed with caution (overall Grade C evidence). Very few RCTs have assessed the impact of interventions for periodontal disease on stillbirth and perinatal mortality, and most have been designed with pre-term birth as the primary outcome and have been underpowered to detect impact on perinatal deaths. Although clinical trial data suggest that non-surgical periodontal treatment in the second trimester is a safe and effective strategy to improve periodontal indicators, there is currently insufficient evidence to recommend routine periodontal care as an effective strategy to prevent stillbirths or pre-term birth [183,184,182]. This emerging area of research warrants further study, however, particularly in low-/middle-income countries. Care for periodontal disease provided prior to pregnancy may offer more significant benefits, but this strategy has not yet been tested.

An additional mechanism by which periodontal care could prevent stillbirths is the emerging and clinically important link between periodontal disease and pre-eclampsia. A recent meta-analysis by Vergnes et al. 2008 reported that women with periodontal disease had almost double the risk of pre-eclampsia compared with women without periodontal disease (OR = 1.76, 95% CI: 1.43–2.18) [185]. This linkage, if it can be confirmed, would be particularly relevant to low-/middle-income country settings given high rates of periodontal disease as well as higher incidence of pre-eclampsia than in high-income countries. Verifying this association and identifying the physiological mechanisms involved will require further studies.

## Summary

While high-quality Cochrane or other published reviews or meta-analyses were available for many interventions, few of these reviews or their component studies reported stillbirths specifically, and evidence from different studies and different reviews was frequently conflicting. Additionally, comparing findings even for the same intervention was often difficult due to differing study objectives and design issues including study incommensurability, differences in outcome definitions, and differences in allocation technique. Even rigorous intervention trials were usually conducted in tertiary care centres where even controls received ANC, often of better quality than women who do not enroll in such trials; in such trials with appropriate care and monitoring, stillbirths are more rare than in the general population (e.g., use of anti-hypertensive agents in pregnant women with hypertension). This bias limits generalisability of findings and further hinders demonstration of impact attributable to an intervention. To bridge the evidence gap, there is a need for large research trials to report stillbirths–disaggregated from the perinatal mortality composite measure–as an outcome whenever feasible, and for rigorous RCTs in low-resource settings. The recommendation of each intervention for inclusion in programs is given in Table 18.

**Table 18:** Summary of evidence grading for all interventions prior to and during pregnancy to prevent stillbirth and perinatal mortality reviewed in this paper

	**Evidence of no or negative impact** (leave out of programs)	**Uncertain evidence** (need for additional research before including in programs)	**Some evidence** (may include in programs, but further evaluation is warranted)	**Clear evidence** (merits inclusion in programs)
Calcium supplementation for pregnancy-induced hypertension		X^1^		

Anti-hypertensives		X		

Anti-platelet agents		X		

Anti-oxidants	X			

Heparin				X (for certain conditions)

Management of intrahepatic cholestasis			X	

Plasma exchange		X		

Cervical cerclage		X		

Anti-helminthics			X	

Syphilis screening and treatment			X	

Antibiotics for BV, asymptomatic bacteriuria, and GBS		X		

Antibiotics for PROM/PPROM		X		

Anti-malarials			X^2^	

ITNs				X

PMTCT for HIV	X^2^			

Periodontal care		X		

^1^Possibly effective in some low-resource settings

^2^Maternal and infant benefit

In many low-/middle-income countries, stillbirths associated with infection comprise a large proportion of stillbirths. Preventing stillbirth associated with maternal infection is appealing because it targets a large proportion of the burden, and interventions and service delivery needs are relatively straightforward, short-term, and noninvasive compared with behavioural, surgical, or nutritional interventions. While the evidence base for prevention of stillbirths with interventions prior to or during pregnancy is limited, evidence strongly supports a benefit of syphilis screening and treatment and malaria prophylaxis in endemic areas, and these interventions should be incorporated into ANC programs where epidemiological evidence indicates women are at risk. Some estimates suggest that better syphilis screening and treatment could prevent as many as half of all stillbirths in areas with high syphilis prevalence [186]. When women are treated for syphilis successfully, their risk of stillbirth is similar to uninfected women [187]. The logistical challenges of providing syphilis screening and treatment, particularly at the community level, are many, including training workers to perform a complex test or tests, evaluating ambiguities in diagnosis, preventing test and pharmaceutical supply stock outs, managing the occasional penicillin sensitivity, and ensuring that clients receive both test results and treatment. However, the high rates of ANC attendance (one or more visits) in much of sub-Saharan Africa and South Asia provide a potential infrastructural framework to provide access to this important intervention.

Given the endemicity of malaria in much of sub-Saharan Africa and South Asia, where stillbirth rates are concomitantly high, the three-pronged approach to malaria prevention and treatment advocated by the WHO (ITNs, IPT, and treatment of parasitemia with artemisinin-based therapies) appears prudent, though further research is needed about the safety of artemisinin-based drugs other than artesunate and the impact of timing of dosage on stillbirth rates.

Other interventions, such as anti-helminthic treatment, showed promising impact on stillbirth rates but require more studies to confirm effectiveness. While we recommend the continued practice of PMTCT for HIV, and malaria chemoprophylaxis in women of low parity, these recommendations are based primarily on demonstrated benefit for maternal or other fetal/neonatal outcomes, rather than definitive impact on stillbirths or perinatal mortality.

### Research gaps

The area of stillbirth prevention, particularly the development of interventions that address the multifactorial causes of antepartum stillbirths, is a ripe area for new research (Table 19). In this review, we found evidence that several interventions brought about reductions in known risk factors for stillbirth, yet failed to bring about statistically significant improvements in stillbirth rates. For example, anti-platelet agents clearly reduced rates of PIH and pre-eclampsia, yet no statistically significant impact on stillbirths was noted. Similarly, antibiotic treatment for PROM was associated with a significant reduction in chorioamnionitis, yet yielded no statistically significant reduction in stillbirth rates. These findings are most likely attributable to studies underpowered to detect impact of the intervention on stillbirth rates, but could also be due to intervention at a point too distal in a multi-causal pathway, a deleterious effect of the intervention itself, including adverse effects of drugs, etc. For these interventions and others (Table 19), further research specifically designed to measure stillbirth as an outcome is needed.

**Table 19:** Research gaps

***Basic science and physiological studies***
• Mechanisms of causation of hypertensive disorders of pregnancy and how these cause stillbirths*
• Auto-immune pathophysiology in stillbirth causation
• Association of periodontal disease with pre-eclampsia and pathophysiology of subsequent stillbirth*
• Dynamics of stillbirth causation in intrahepatic cholestasis
• Drug safety and efficacy studies:
◦ Anti-malarials in pregnancy
◦ Drugs for intrahepatic cholestasis
◦ Non-penicillin treatments for syphilis
◦ Antibiotic use in pregnancy
• Identification of other unknown risk factors
• Prevalence of uterine abnormalities in low-/middle-income countries
***Pilot/cohort studies of interventions***
• New approaches for PIH and chronic hypertension management in community settings*
• Management protocols for HIV and syphilis co-infection
• Management of penicillin drug allergy in community settings
• Diagnosis and surgical repair of uterine abnormalities in women with recurrent fetal loss
• Intravenous immunoglobulin treatment (compared to heparin, aspirin, or other anti-coagulants) in selected populations with recurrent pregnancy loss and antiphospholipid antibodies
***Well-designed large RCTs of interventions powered to detect stillbirths ***
• Periodontal care studies powered to detect impact on stillbirth rates*
• Calcium supplementation to prevent PIH and pre-eclampsia in deficient populations
• Anti-oxidant supplementation in deficient populations
• Management of intrahepatic cholestasis including nonstress test, amniotic fluid index, meconium screening and early delivery
• Calcium supplementation in high-risk pregnancies*
• Antibiotics for pPROM*
***Effectiveness trials in large populations/at scale***
• Maternal anti-helminthic treatment: impact on maternal anaemia and stillbirth*
• On-site syphilis testing and treatment
• ITNs for *Plasmodium vivax* and in lower-transmission settings (Asia and Latin America)

*Priority areas

Large gaps remain in the basic science of stillbirth causation and many of the maternal conditions and pathologies that lead to stillbirth, including pathophysiological mechanisms involved in pre-eclampsia, autoimmune and inflammatory responses that threaten pregnancy, still-unknown risk factors, and drug safety and efficacy studies. These studies will guide development of appropriate interventions for maternal conditions and infections associated with stillbirth causation.

Evidence for some newly recognised risk factors for stillbirth, such as periodontal disease, suggests the need for large, appropriately designed randomised trials to assess whether effective interventions can be designed to minimise these risks.

### Conclusion

Knowledge and evidence gaps in the dynamics and prevention of antepartum stillbirth are numerous and deep. At present, there is no standard international classification system for stillbirth causation, and disagreement persists about the lower benchmarks of birth weight and gestational age used to define stillbirth. These ambiguities complicate measurement of stillbirths, comparison of different studies measuring stillbirth, and tracking trends over time [186]. Development of practical, standardised templates to assess causes of stillbirth is underway, which, if adopted internationally, will help to more accurately measure the burden of intrapartum versus antepartum stillbirths and their associated causes. These data are crucial to inform the development of appropriate interventions. Meanwhile, the numerous gaps in the growing list of stillbirth risk factors, the pathophysiology of certain maternal infections and conditions, and the many interventions for which there is conflicting evidence of benefit suggest the need for greater investment in large studies powered to measure stillbirth rate reductions. Armed with more definitive knowledge of which interventions are effective and prevent the most stillbirths in particular settings, we can more effectively select and implement these interventions and prevent unnecessary loss of life.

Programmatically, the clearest evidence of impact in stillbirth prevention is adequate prevention and treatment of maternal infections such as syphilis and malaria. Given the enormous human burden of both of these diseases in many countries with the world's highest stillbirth rates, effective prevention and treatment of syphilis and malaria have the potential to greatly reduce global stillbirth rates.

Efforts prior to and during pregnancy to prevent stillbirth will be most effective in conjunction with effective monitoring interventions in pregnancy, prompt recognition and management of high-risk pregnancies and complications, and a system of interlinked facilities capable of prompt referral to ensure that complications are capably managed to prevent unnecessary loss of life for mothers or their babies.

## Abbreviations

ANC, antenatal care; APL, anti-phospholipid; ART, anti-retroviral therapy; CI, confidence interval; DBP, diastolic blood pressure; ENMR, early neonatal mortality rate; FDA, Food and Drug Administration; FGM, female genital mutilation; FGR, fetal growth restriction; GBS, Group B Streptococcus; HAART, highly active anti-retroviral therapy; HIV, human immunodeficiency virus; HR, hazard ratio; ITN, insecticide-treated net; IUGR, intrauterine growth restriction; IVIG, intravenous immunoglobulin; LBW, low birth weight; LDA, low-dose aspirin; LMW, low molecular weight; LOE, level of evidence; NIH, National Institutes of Health; NMR, neonatal mortality rate; NS, nonsignificant; NTD, neural tube defect; OR, odds ratio; PIH, pregnancy-induced hypertension; PMR, perinatal mortality rate; PMTCT, prevention of mother-to-child transmission; PPROM, pre-term premature rupture of membranes; PROM, premature rupture of membranes; PTT, partial thromboplastin time; RCT, randomised controlled trial; RD, risk difference; RDA, recommended dietary allowance; RPR, rapid plasma reagin; SAMe, S-adenosyl-L-methionine; SB, stillbirth; SBP, systolic blood pressure; SBR, stillbirth rate; SGA, small for gestational age; SP, sulphadoxine pyrimethamine; TPHA, *Treponema pallidum* haemagglutination test; UDCA, ursodeoxycholic acid; VBAC, vaginal birth after Caesarean; WHO, World Health Organization

## Competing interests

The authors declare that they have no competing interests.

## Authors' contributions

The paper was written and reviewed by all the authors.

## Supplementary Material

Additional file 1 **Web Table 1. Component studies in Trumbo et al. 2007 meta-analysis: Impact of calcium supplementation for prevention of PIH and pre-eclampsia on stillbirths/perinatal mortality**. Component studies in Trumbo et al. 2007 meta-analysis showing impact on stillbirths/perinatal mortality Click here for file

Additional file 2 **Web Table 2. Component studies in Hofmeyr et al. 2007 meta-analysis: Impact of calcium supplementation for prevention of PIH**. Component studies in Hofmeyr et al. 2007 meta-analysis reporting impact on stillbirths/perinatal mortality Click here for file

Additional file 3 **Web Table 3. Component studies in Abalos et al. 2007 meta-analysis: impact of antihypertensive drugs for chronic maternal hypertension**. Component studies in Abalos et al. 2007 meta-analysis reporting impact on stillbirths/perinatal mortality Click here for file

Additional file 4 **Web Table 4. Component studies in Magee et al. 2003 meta-analysis: impact of anti-hypertensive drugs for chronic maternal hypertension**. Component studies in Magee et al. 2003 meta-analysis reporting impact on stillbirths/perinatal mortality Click here for file

Additional file 5 **Web Table 5. Component studies in Duley et al. 2006 meta-analysis: impact of anti-hypertensive drugs for chronic maternal hypertension**. Component studies in Duley et al. 2006 meta-analysis reporting impact on stillbirths/perinatal mortality Click here for file

Additional file 6 **Web Table 6. Component studies in King et al. 2003 meta-analysis: impact of anti-hypertensive drugs for chronic maternal hypertension**. Component studies in King et al. 2003 meta-analysis reporting impact on stillbirths/perinatal mortality Click here for file

Additional file 7 **Web Table 7. Component studies in Say et al. 1996 meta-analysis: impact of anti-hypertensive drugs for chronic maternal hypertension**. Component studies in Say et al. 1996 meta-analysis reporting impact on stillbirths/perinatal mortality Click here for file

Additional file 8 **Web Table 8. Component studies in Meher and Duley. 2007 meta-analysis: impact of anti-hypertensive drugs for chronic maternal hypertension**. Component studies in Meher and Duley. 2007 meta-analysis reporting impact on stillbirths/perinatal mortality Click here for file

Additional file 9 **Web Table 9. Component studies in Duley et al. 2007 meta-analysis: impact of anti-platelet agents**. Component studies in Duley et al. 2007 meta-analysis reporting impact on stillbirths/perinatal mortality Click here for file

Additional file 10 **Web Table 10. Component studies in Askie et al. 2007 meta-analysis: impact of anti-platelet agents**. Component studies in Askie et al. 2007 meta-analysis reporting impact on stillbirths/perinatal mortality Click here for file

Additional file 11 **Web Table 11. Component studies in Empson et al. 2005 meta-analysis: Impact of treatment during pregnancy for lupus anti-coagulant or anti-phospholipid syndrome**. Component studies in Empson et al. 2005 meta-analysis reporting impact on stillbirths/perinatal mortality Click here for file

Additional file 12 **Web Table 12. Component studes in Di Nisio et al. 2005: Impact of LMWH (enoxaparin) versus aspirin**. Component studies in Di Nisio et al. 2005 review reporting impact on stillbirths/perinatal mortality Click here for file

Additional file 13 **Web Table 13. Component studies in Gates et al. 2002 meta-analysis: Impact of thromboprophylaxis during pregnancy**. Component studies in Gates et al. 2002 meta-analysis reporting impact on stillbirths/perinatal mortality Click here for file

Additional file 14 **Web Table 14. Component studies in Rumbold et al. 2008: impact of anti-oxidant supplements**. Component studies in Rumbold et al. 2008 meta-analysis reporting impact on stillbirths/perinatal mortality Click here for file

Additional file 15 **Web Table 15. Component studies in Rumbold and Crowther 2005: Impact of anti-oxidants**. Component studies in Rumbold **and Crowther **2005 meta-analysis reporting impact on stillbirths/perinatal mortality Click here for file

Additional file 16 **Web Table 16. Component studies in Drakeley et al. 2003 meta-analysis: Impact of cervical cerclage on stillbirths and perinatal mortality**. Component studies in Drakeley et al. 2003 meta-analysis reporting impact on stillbirths/perinatal mortality Click here for file

Additional file 17 **Web Table 17. Component studies in Jorgensen et al. 2007 meta-analysis: Impact of cervical cerclage**. Component studies in Jorgensen et al. 2007 meta-analysis reporting impact on stillbirths/perinatal mortality Click here for file

Additional file 18 **Web Table 18. Component studies in King and Flenady 2002 meta-analysis: impact of anti-biotics in high-risk pregnancy**. Component studies in King and Flenady 2002 meta-analysis reporting impact on stillbirths/perinatal mortality Click here for file

Additional file 19 **Web Table 19. Component studies in McDonald et al. 2007 meta-analysis: impact of anti-biotics in high-risk pregnancy**. Component studies in McDonald et al. 2007 meta-analysis reporting impact on stillbirths/perinatal mortality Click here for file

Additional file 20 **Web Table 20. Component studies in Lumbiganon et al. 2004 meta-analysis: impact of anti-biotics in high-risk pregnancy**. Component studies in Lumbiganon et al. 2004 meta-analysis reporting impact on stillbirths/perinatal mortality Click here for file

Additional file 21 **Web Table 21. Component studies in Goldenberg et al. 2006 meta-analysis: Impact of vaginally administered chlorhexidine during labour on stillbirths/perinatal mortality**. Component studies in Goldenberg et al. 2006 meta-analysis reporting impact on stillbirths/perinatal mortality Click here for file

Additional file 22 **Web Table 22. Component studies in Thinkhamrop et al. 2002 meta-analysis: impact of anti-biotics in high-risk pregnancy**. Component studies in Thinkhamrop et al. 2002 meta-analysis reporting impact on stillbirths/perinatal mortality Click here for file

Additional file 23 **Web Table 23. Component studies in Kenyon et al. 2003 meta-analysis: Impact of antibiotics for PPROM/PROM**. Component studies in Kenyon et al. 2003 meta-analysis reporting impact on stillbirths/perinatal mortality Click here for file

Additional file 24 **Web Table 24. Component studies in Flenady and King 2002 meta-analysis: Impact of anti-biotics for PROM at or near term**. Component studies in Flenady and King 2002 meta-analysis reporting impact on stillbirths/perinatal mortality Click here for file

Additional file 25 **Web Table 25. Component studies in Garner and Gulmezoglu 2006 meta-analysis: impact of anti-malarials in malaria-endemic areas**. Component studies in Garner **and Gulmezoglu** 2006 meta-analysis reporting impact on stillbirths/perinatal mortality Click here for file

Additional file 26 **Web Table 26. Component studies in Gamble et al. 2006 & 2007 meta-analyses: impact of ITNs**. Component studies in Gamble et al. 2006, 2007 meta-analysis reporting impact on stillbirths/perinatal mortality Click here for file

Additional file 27 **Web Table 27. Component studies in Volmink et al. 2007 meta-analysis: impact of PMTCT**. Component studies in Volmink et al. 2007 meta-analysis reporting impact on stillbirths/perinatal mortality Click here for file

Additional file 28 **Web Table 28. Component studies in Suksomboon et al. 2007 meta-analysis: impact of PMTCT**. Component studies in Suksomboon et al. 2007 meta-analysis reporting impact on stillbirths/perinatal mortality Click here for file

Additional file 29 **Web Table 29. Component studies in Wiysonge et al. 2005 meta-analysis: impact of PMTCT**. Component studies in **Wiysonge** et al. 2005 meta-analysis reporting impact on stillbirths/perinatal mortality Click here for file

Additional file 30 **Web Table 30. Component studies in Xiong et al. 2006**: **impact of periodontal disease**. Component studies in Xiong et al. 2006 reporting impact on stillbirths/perinatal mortality Click here for file

Additional file 31 **Web Table 31. Component studies in Xiong et al. 2007**: **impact of periodontal disease**. Component studies in Xiong et al. 2007 reporting impact on stillbirths/perinatal mortality Click here for file
