# Supplementary material for: Reducing stillbirths: prevention and management of medical disorders and infections during pregnancy
Source: BMC Pregnancy Childbirth. 2009 May 7;9(Suppl 1):S4. doi: 10.1186/1471-2393-9-S1-S4 (PMC2679410; doi:10.1186/1471-2393-9-S1-S4)
Supplement: Additional file 1 — Web Table 1. Component studies in Trumbo et al. 2007 meta-analysis: Impact of calcium supplementation for prevention of PIH and pre-eclampsia on stillbirths/perinatal mortality. Component studies in Trumbo et al. 2007 meta-analysis showing impact on stillbirths/perinatal mortality [file 1471-2393-9-S1-S4-S1.doc]

**Web Table 1. Component studies in Trumbo et al. 2007 [1] meta-analysis: Impact of calcium supplementation for prevention of PIH and pre-eclampsia on stillbirths/perinatal mortality**

| **Source** | **Location and Type of Study** | **Intervention** | **Stillbirths / Perinatal Outcomes** |
| --- | --- | --- | --- |
| Levine et al. 1997 [2] | USA.  Double-blind RCT. N=4589 pregnant nulliparas 31 to 21 weeks of gestation (N = 2295 calcium, N = 2294 placebo). | Assessed impact of administering 2 g/day elemental calcium as calcium carbonate (intervention), or placebo (controls) taken until delivery, development of pre-eclampsia or suspicion of urolithiasis. All women took 50 mg calcium per day as normal supplementation and were asked to drink 6 glasses of water per day. | SB or death before discharge from hospital: RR=1.08 (95% CI: 0.63-1.86) **[NS]**  [27/2163 vs. 25/2173 in intervention vs. control groups, respectively].  Pre-eclampsia: RR = 0.94 (95% CI: 0.76 – 1.16) **[NS]**  [158/2295 (6.9%) vs. 168/2294 (7.3%) in intervention and control groups, respectively]  PIH (without pre-eclampsia): RR = 0.88 (95% CI: 0.78 – 1.01) **[NS]**  [351/2295 (15.3%) vs. 397/2294 (17.3%) in intervention and control groups, respectively] |
| Sanchez-Ramos et al. 1994 [3] | USA (Jacksonville, Florida). University hospital (prenatal clinics) serving low-income population.  RCT. Normotensive nulliparas at 20-24 weeks’ gestation. | Assessed impact of calcium supplementation with 2 g per day elemental calcium as 500 mg calcium carbonate tablets (intervention) vs. placebo (controls). Compliance (79% vs. 81%) checked with electronic pillboxes. | SB or death before discharge from hospital: RR=0.39 (95% CI: 0.02-9.20)**[NS]**  [0/29 vs. 1/34 in intervention vs. control groups, respectively].  Pre-eclampsia: RR = 0.37 (95% CI: 0.15 – 0.92); P = 0.01  [4/29 (13.8%) vs. 15/34 (44.1%) in intervention and control groups, respectively].  PIH: RR = 0.46 (95% CI: 0.25 – 0.86); P = 0.01  [9/29 (31.0%) vs. 22/34 (64.7%) in intervention and control groups, respectively]. |
| Belizan et al. 1991 [4] | Argentina. Multicentred.  RCT. N = 1194 pregnant women (N=593 intervention, N=601 controls). | Assessed impact of administering 2 g calcium as 500 mg calcium carbonate tablets (intervention) vs. placebo (controls). Compliance was 84% (calcium) and 86% (placebo). | SB or death before discharge from hospital: RR=0.87 (95% CI: 0.29-2.58)**[NS]**  [6/558 vs. 7/567 in intervention vs. control groups, respectively].  Pre-eclampsia: OR = 0.65 (95% CI: 0.35 – 1.25) **[NS]**  [2.6% vs. 3.9% in intervention and control groups, respectively]  Gestational hypertension: OR = 0.64 (95% CI: 0.43 – 0.96)  [7.2% vs. 10.7% in intervention and control groups, respectively]. |
| Villar et al. 1987 [5] | USA (Baltimore, Maryland) and Argentina (Rosario)  RCT. 1983-1985. Nulliparous or primiparous women age 18-30 with singleton pregnancy, known menstrual dates, negative roll-over test. N=34 black women from Johns Hopkins Hospital, Baltimore; N=18 white women from Rosario, Argentina. | Assessed impact of calcium supplementation with calcium carbonate 1.5 g (500 mg tablets) from 26 weeks' gestation (intervention) vs. placebo (controls). Women at John Hopkins Hospital also received vitamin preparations containing 200 mg Ca and 100 mg Mg/day. | SB or death before discharge from hospital: [0/25 vs. 0/27 in intervention vs. control groups, respectively]. No statistical significance data.  PIH: 4.01% vs. 11.1% in the intervention and control groups, respectively. |
| Belizan et al. 1983 [6] |  |  | Full text not available |
| Crowther et al. 1999 [7] | Australia.  RCT. N=456 nulliparous women with singleton pregnancy < 24 weeks' gestation, blood pressure < 140/90 mmHg who expected to give birth at a collaborating centre. | Assessed impact of administering calcium carbonate 1.8 g daily (intervention) vs. placebo (controls), from 20-24 wks until birth. | SB or death before discharge from hospital: RR= 2.02 (95% CI: 0.18-22.09) **[NS]**  [2/227 vs. 1/229 in intervention vs. control groups, respectively].  Pre-eclampsia: RR = 0.44 (95% CI: 0.21 – 0.90); P = 0.02  [10/227 (4.4%) vs. 23/229 (10.0%) in intervention and control groups, respectively].  PIH: RR = 0.90 (95% CI: 0.59 – 1.38); P = 0.64  [34/227 (15.0%) vs. 38/229 (16.6%) in intervention and control groups, respectively]. |
| Villar and Repke 1990 [8] | USA (Baltimore, Maryland)  RCT. 1985-1988. N=189 healthy women enrolled by wk 23 of gestation; age ≤17 years. | Assessed impact of calcium supplementation with 2 g elemental calcium as 500 mg calcium carbonate tablets (intervention), vs. placebo (controls). All women were prescribed prenatal vitamin tablets containing 200 mg calcium and 100 mg magnesium per day. | SB or death before discharge from hospital: [0/94 vs. 0/95 in intervention vs. control groups, respectively]. No statistical significance data. |

**References**

1. Trumbo PR, Ellwood KC: **Supplemental calcium and risk reduction of hypertension, pregnancy-induced hypertension, and preeclampsia: an evidence-based review by the US Food and Drug Administration**. *Nutr Rev* 2007, **65**(2):78-87.

2. Levine RJ, Hauth JC, Curet LB, Sibai BM, Catalano PM, Morris CD, DerSimonian R, Esterlitz JR, Raymond EG, Bild DE *et al*: **Trial of calcium to prevent preeclampsia**. *N Engl J Med* 1997, **337**(2):69-76.

3. Sanchez-Ramos L, Briones DK, Kaunitz AM, Delvalle GO, Gaudier FL, Walker CD: **Prevention of pregnancy-induced hypertension by calcium supplementation in angiotensin II-sensitive patients**. *Obstet Gynecol* 1994, **84**(3):349-353.

4. Belizan JM, Villar J, Gonzalez L, Campodonico L, Bergel E: **Calcium supplementation to prevent hypertensive disorders of pregnancy**. *N Engl J Med* 1991, **325**(20):1399-1405.

5. Villar J, Repke J, Belizan JM, Pareja G: **Calcium supplementation reduces blood pressure during pregnancy: results of a randomized controlled clinical trial**. *Obstet Gynecol* 1987, **70**(3 Pt 1):317-322.

6. Belizan JM, Villar J, Zalazar A, Rojas L, Chan D, Bryce GF: **Preliminary evidence of the effect of calcium supplementation on blood pressure in normal pregnant women**. *Am J Obstet Gynecol* 1983, **146**(2):175-180.

7. Crowther CA, Hiller JE, Pridmore B, Bryce R, Duggan P, Hague WM, Robinson JS: **Calcium supplementation in nulliparous women for the prevention of pregnancy-induced hypertension, preeclampsia and preterm birth: an Australian randomized trial. FRACOG and the ACT Study Group**. *Aust N Z J Obstet Gynaecol* 1999, **39**(1):12-18.

8. Villar J, Repke JT: **Calcium supplementation during pregnancy may reduce preterm delivery in high-risk populations**. *Am J Obstet Gynecol* 1990, **163**(4 Pt 1):1124-1131.
