# Supplementary material for: Reducing stillbirths: prevention and management of medical disorders and infections during pregnancy
Source: BMC Pregnancy Childbirth. 2009 May 7;9(Suppl 1):S4. doi: 10.1186/1471-2393-9-S1-S4 (PMC2679410; doi:10.1186/1471-2393-9-S1-S4)
Supplement: Additional file 2 — Web Table 2. Component studies in Hofmeyr et al. 2007 meta-analysis: Impact of calcium supplementation for prevention of PIH. Component studies in Hofmeyr et al. 2007 meta-analysis reporting impact on stillbirths/perinatal mortality [file 1471-2393-9-S1-S4-S2.doc]

**Web Table 2. Component studies in Hofmeyr et al. 2007 [1] meta-analysis: Impact of calcium supplementation for prevention of PIH**

| **Source** | **Location and Type of Trial** | **Intervention** | **Stillbirths and perinatal outcomes** |
| --- | --- | --- | --- |
| 1. Belizan 1991, 1997, 1992.  Stephens 1998  Villar 1990, 1988 [2, 3] [4-7]. | Argentina.  RCT. Multicentre trial (N=593 intervention, N=601 controls). | Assessed impact of administering 2 g calcium as 500 mg calcium carbonate tablets (intervention) vs. placebo (controls). Compliance was 84% (calcium) and 86% (placebo). | SB or death before discharge from hospital: RR=0.87 (95% CI: 0.29-2.58)**[NS]**  [6/558 vs. 7/567 in intervention vs. control groups, respectively]. |
| 2. CPEP 1997 Hatton et al. 2003.  Levine 1996, 1997, 1997, 1992 [8] [9-12] | USA.  RCT. N=4589 pregnant nulliparas (45% black, 35% non-Hispanic white, 17% Hispanic white women). | Assessed impact of administering 2 g/day elemental calcium as calcium carbonate (intervention), or placebo (controls) taken until delivery, development of pre-eclampsia or suspicion of urolithiasis. All women took 50 mg calcium per day as normal supplementation and were asked to drink 6 glasses of water per day. | SB or death before discharge from hospital: RR=1.08 (95% CI: 0.63-1.86) **[NS]**  [27/2163 vs. 25/2173 in intervention vs. control groups, respectively]. |
| 3. Crowther et al. 1999, 1998 [13, 14] | Australia.  RCT. N=456 nulliparous women with singleton pregnancy < 24 weeks' gestation, blood pressure < 140/90 mmHg who expected to give birth at a collaborating centre. | Assessed impact of administering calcium carbonate 1.8 g daily (intervention) vs. placebo (controls), from 20-24 wks until birth. | SB or death before discharge from hospital: RR= 2.02 (95% CI: 0.18-22.09) **[NS]**  [2/227 vs. 1/229 in intervention vs. control groups, respectively]. |
| 4. Lopez-Jaramillo et al. 1989, 1987 Narvaez 1988 [15-17] | Ecuador (Quito).  RCT. N=106 women (N=55 intervention, N=51 controls). | Assessed impact of calcium 2 g daily as calcium gluconate, from after 23 wks gestation until delivery (intervention), vs placebo (controls). | SB or death before discharge from hospital: [0/49 vs. 0/43 in intervention vs. control groups, respectively]. No statistical significance data. |
| 5. Lopez-Jaramillo et al. 1997 [18] | Ecuador (Quito).  RCT. N=274 women (N= 134 intervention, N=140 controls). | Assessed impact of calcium 2 g daily as calcium carbonate from 20 wks (intervention) vs placebo (controls). | SB or death before discharge from hospital: [0/125 vs. 0/135 in intervention vs. control groups, respectively]. No statistical significance data. |
| 6. Purwar et al. 1996, 1996 [19, 20] | India (Nagpur).  RCT. N=201 women (N=103 intervention, N=98 controls. | Assessed impact of calcium 2 g daily (intervention) vs. placebo (controls), taken orally from 20 wks. | SB or death before discharge from hospital: [0/97 vs. 0/93 in intervention vs. control groups, respectively]. No statistical significance data. |
| 7. Sanchez-Ramos et al. 1994, 1994 [21, 22] | USA (Jacksonville, Florida)  RCT. Normotensive nulliparas visiting university hospital serving low-income population. | Assessed impact of calcium supplementation with 2 g per day elemental calcium as 500 mg calcium carbonate tablets (intervention) vs. placebo (controls). Compliance (79% vs. 81%) checked with electronic pillboxes. | SB or death before discharge from hospital: RR=0.39 (95% CI: 0.02-9.20)**[NS]**  [0/29 vs. 1/34 in intervention vs. control groups, respectively]. |
| 8. Villar 1987  Repke 1989 [23, 24] | USA (Baltimore, Maryland) and Argentina (Rosario)  RCT. 1983-1985. Nulliparous or primiparous women age 18-30 with singleton pregnancy, known menstrual dates, negative roll-over test. N=34 black women from Johns Hopkins Hospital, Baltimore; N=18 white women from Rosario, Argentina. | Assessed impact of calcium supplementation with calcium carbonate 1.5 g (500 mg tablets) from 26 weeks' gestation (intervention) vs placebo (controls). Women at John Hopkins Hospital also received vitamin preparations containing 200 mg Ca and 100 mg Mg/day. | SB or death before discharge from hospital: [0/25 vs. 0/27 in intervention vs. control groups, respectively]. No statistical significance data. |
| 9. Villar 1990, 1990, 1988 [6, 7, 25] | USA (Baltimore, Maryland)  RCT. 1985-1988. N=189 healthy women enrolled by wk 23 of gestation; age ≤17 years. | Assessed impact of calcium supplementation with 2 g elemental calcium as 500 mg calcium carbonate tablets (intervention), vs. placebo (controls). All women were prescribed prenatal vitamin tablets containing 200 mg calcium and 100 mg magnesium per day. | SB or death before discharge from hospital: [0/94 vs. 0/95 in intervention vs. control groups, respectively]. No statistical significance data. |
| 10. WHO 2006. Villar et al. [26] | Argentina, Egypt, India, Peru, South Africa, Vietnam.  RCT. Multicentre trial. N=8325 women. | Assessed impact of calcium supplementation with chewable calcium carbonate tablets with 500 mg elemental calcium, 3x daily (intervention), vs. placebo (controls), from enrolment till delivery. | SB or death before discharge from hospital: RR=0.86 (95% CI: 0.69-1.07) **[NS]**  [142/4181 vs. 166/4197 in intervention vs. control groups, respectively]. |

References

1. Hofmeyr GJ, Duley L, Atallah A: **Dietary calcium supplementation for prevention of pre-eclampsia and related problems: a systematic review and commentary**. *BJOG* 2007, **114**(8):933-943.

2. Belizan JM, Villar J, Gonzalez L, Campodonico L, Bergel E: **Calcium supplementation to prevent hypertensive disorders of pregnancy**. *N Engl J Med* 1991, **325**(20):1399-1405.

3. Belizan JM, Villar J, Bergel E, del Pino A, Di Fulvio S, Galliano SV, Kattan C: **Long-term effect of calcium supplementation during pregnancy on the blood pressure of offspring: follow up of a randomised controlled trial**. *BMJ* 1997, **315**(7103):281-285.

4. Stephens IF: **Effect of calcium supplementation during pregnancy on blood pressure of offspring. Authors cannot be sure of effect's generalisability to all children aged 5-9**. *BMJ* 1998, **316**(7126):234.

5. Belizan JM: **Prevention of hypertensive disorders of pregnancy with calcium supplementation.** In: *8th World Congress on Hypertension in Pregnancy: November 8-12. 1992; Buenos Aires.*; 1992.

6. Villar J, Belizan JM, Repke J: **The effect of calcium supplementation on the incidence of hypertensive disorders of pregnancy and prematurity**. In: *7th World Congress of Hypertension in Pregnancy: 1990; Perugia, Italy*; 1990.

7. Villar J, Belizan JM, Repke JT: **Does calcium supplementation reduce pregnancy-induced hypertension and prematurity?** In: *Advances in the prevention of low birthweight: 1988 May 8-11; Cape Cod, Massachusetts*; 1988 May 8-11: 187-195.

8. Hatton DC, Harrison-Hohner J, Coste S, Reller M, McCarron D: **Gestational calcium supplementation and blood pressure in the offspring**. *Am J Hypertens* 2003, **16**(10):801-805.

9. Levine RJ, Esterlitz JR, Raymond EG, DerSimonian R, Hauth JC, Ben Curet L, Sibai BM, Catalano PM, Morris CD, Clemens JD *et al*: **Trial of Calcium for Preeclampsia Prevention (CPEP): rationale, design, and methods**. *Control Clin Trials* 1996, **17**(5):442-469.

10. Levine R: **Calcium for preeclampsia prevention (CPEP): a double-blind, placebo-controlled trial in healthy nulliparas**. *American Journal of Obstetrics and Gynecology;* CPEP Study Group 1997, **176**:S2.

11. Levine RJ, Hauth JC, Curet LB, Sibai BM, Catalano PM, Morris CD, DerSimonian R, Esterlitz JR, Raymond EG, Bild DE *et al*: **Trial of calcium to prevent preeclampsia**. *N Engl J Med* 1997, **337**(2):69-76.

12. Levine RJ, for the CPEP Study Group: **The trial of calcium for preeclampsia prevention (CPEP).** In: *8th World Congress on Hypertension in Pregnancy - Protagonists and Presentations: 1992 November 8-12.; Buenos Aires, Argentina.*; 1992 November 8-12.

13. Crowther CA, Hiller JE, Pridmore B, Bryce R, Duggan P, Hague WM, Robinson JS: **Calcium supplementation in nulliparous women for the prevention of pregnancy-induced hypertension, preeclampsia and preterm birth: an Australian randomized trial. FRACOG and the ACT Study Group**. *Aust N Z J Obstet Gynaecol* 1999, **39**(1):12-18.

14. Crowther C, Hiller J, Pridmore B, Bryce P, Duggan P, Hague W, al. e: **Calcium supplementation in nulliparous women for the prevention of pregnancy induced hypertension, pre-eclampsia and preterm birth: an Austailian randomized trial.** In: *2nd Annual Congress of the Perinatal Society of Australia and New Zealand: 1998 March 30-April 4.; Alice Springs, Australia.*; 1998 March 30-April 4.

15. Lopez-Jaramillo P, Narvaez M, Weigel RM, Yepez R: **Calcium supplementation reduces the risk of pregnancy-induced hypertension in an Andes population**. *Br J Obstet Gynaecol* 1989, **96**(6):648-655.

16. Lopez-Jaramillo P, Narvaez M, Yepez R: **Effect of calcium supplementation on the vascular sensitivity to angiotensin II in pregnant women**. *Am J Obstet Gynecol* 1987, **156**(1):261-262.

17. Narvaez M, Lopez-Jaramillo P, Weigel M: **Calcium (Ca++) supplementation reduces the risk for pregnancy induced hypertension (PIH).** In: *World Congress of Gynecology and Obstetrics: 1988 October 23-28.; Brazil.*; 1988 October 23-28.: 180-181.

18. Lopez-Jaramillo P, Delgado F, Jacome P, Teran E, Ruano C, Rivera J: **Calcium supplementation and the risk of preeclampsia in Ecuadorian pregnant teenagers**. *Obstet Gynecol* 1997, **90**(2):162-167.

19. Purwar M, Motghare V, Kulkarni H: **Calcium supplementation and prevention of pregnancy induced hypertension: randomized double blind controlled trial**. *Journal of Clinical Epidemiology;* 1996, **49**(Suppl 1):28S.

20. Purwar M, Kulkarni H, Motghare V, Dhole S: **Calcium supplementation and prevention of pregnancy induced hypertension**. *J Obstet Gynaecol Res* 1996, **22**(5):425-430.

21. Sanchez-Ramos L, Delvalle GO, Briones D, Walker C, Delke I, Gaudier F: **Prevention of preeclampsia by calcium supplementation in angiotensin-sensitive patients**. *American Journal of Obstetrics and Gynecology;* 1994, **170**:408.

22. Sanchez-Ramos L, Briones DK, Kaunitz AM, Delvalle GO, Gaudier FL, Walker CD: **Prevention of pregnancy-induced hypertension by calcium supplementation in angiotensin II-sensitive patients**. *Obstet Gynecol* 1994, **84**(3):349-353.

23. Villar J, Repke J, Belizan JM, Pareja G: **Calcium supplementation reduces blood pressure during pregnancy: results of a randomized controlled clinical trial**. *Obstet Gynecol* 1987, **70**(3 Pt 1):317-322.

24. Repke JT, Villar J, Anderson C, Pareja G, Dubin N, Belizan JM: **Biochemical changes associated with blood pressure reduction induced by calcium supplementation during pregnancy**. *Am J Obstet Gynecol* 1989, **160**(3):684-690.

25. Villar J, Repke JT: **Calcium supplementation during pregnancy may reduce preterm delivery in high-risk populations**. *Am J Obstet Gynecol* 1990, **163**(4 Pt 1):1124-1131.

26. Villar J, Abdel-Aleem H, Merialdi M, Mathai M, Ali MM, Zavaleta N, Purwar M, Hofmeyr J, Nguyen TN, Campodonico L *et al*: **World Health Organization randomized trial of calcium supplementation among low calcium intake pregnant women**. *Am J Obstet Gynecol* 2006, **194**(3):639-649.
