# Supplementary material for: Reducing stillbirths: prevention and management of medical disorders and infections during pregnancy
Source: BMC Pregnancy Childbirth. 2009 May 7;9(Suppl 1):S4. doi: 10.1186/1471-2393-9-S1-S4 (PMC2679410; doi:10.1186/1471-2393-9-S1-S4)
Supplement: Additional file 3 — Web Table 3. Component studies in Abalos et al. 2007 meta-analysis: impact of antihypertensive drugs for chronic maternal hypertension. Component studies in Abalos et al. 2007 meta-analysis reporting impact on stillbirths/perinatal mortality [file 1471-2393-9-S1-S4-S3.doc]

**Web Table 3. Component studies in Abalos et al. 2007 [1] meta-analysis: impact of antihypertensive drugs for chronic maternal hypertension**

| **Source** | **Location and Type of Study** | **Intervention** | **Stillbirths / Perinatal Outcomes** |
| --- | --- | --- | --- |
| 1. Blake S, MacDonald D. 1991 [2] | Ireland.  RCT. N=36 women <38 wks gestation with BP ≥140/90 mmHg on two separate days, without proteinuria. | Compared impact of administering choice between atenolol 50-100 mg/day and methyldopa 750-2250 mg/day (intervention). Two drugs combined when monotherapy inadequate. Bendrofluazide 2.5-5.0 mg added as a third agent when necessary. Controls given no anti-hypertensive. | SBR: RR=2.24 (95% CI: 0.22- 22.51)**[NS]**  [2/17 vs. 1/19 in intervention vs. control groups, respectively.] |
| 2. Borghi et al. 2000 [3] | Italy.  RCT. N=20 women with pre-eclampsia. | Compared impact of administration of nifedipine GITS 30-60 mg/day (intervention) to methyldopa 500-1000 mg/day (controls) | SBR+NMR: [0/10 in both groups.] RR not estimable. |
| 3. Bott-Kanner et al. 1992 [4] | Israel.  RCT. N=60 women <35 wks gestation with DBP 85-99 mmHg x 2, 12 hr apart, and no treatment for hypertension during this pregnancy. | Assessed impact of administering pindolol 5 mg 2x/day (intervention). If DBP in intervention group still ≥ 85 mmHg on day 3, increased to 5 mg x 3/day, if no response next day, increased to 10 mg x 2/day. If DBP 100-109 mmHg x2 or > 110 mmHg x1, hydralazine added for pindolol group. Control group given placebo unless DBP elevated; pindolol given first, followed by hydralazine if DBP > 100 mmHg. | SBR: RR=3.00 (95% CI: 0.13-70.83)**[NS]**  [1/30 vs. 0/30 in intervention vs. control groups, respectively.] |
| 4. Butters et al. 1990 [5] | UK.  RCT. N=33 women 12-24 wks gestation with SBP 140-170 mmHg and DBP 90-110 mmHg x 2, 24 hr apart. | Compared the impact of administration of atenolol 50-200 mg/day (intervention) vs. placebo (controls). | SBR: RR=2.81 (95% CI: 0.12-63.83)  [1/15 vs. 0/14 in intervention vs. control groups, respectively.]. |
| 5. Casavilla and Vega 1988. [6] | Argentina.  RCT. N=36 women >14 wks gestation with BP ≥140/90 mmHg and ≤170/110 mmHg. Argentina. | Compared impact of administering mepindolol, increasing weekly doses, from 5-10 mg/day (intervention) vs. methyldopa, increasing weekly doses from 500-2000 mg/day (controls). | Miscarriage+SBR+NMR: RR=1.00 (95% CI: 0.07-14.79) **[NS]**  [1/18 vs. 1/18 in intervention vs. control groups, respectively]. |
| 6. Catalano et al. 1997 [7] | Italy.  RCT. N=100 primigravid women, 26-36 wks gestation with SBP 140-160 mmHg, and DBP 90-110 mmHg in first 24 hr after admission and proteinuria <300 mg/24 hr. | Compared the impact of administering nifedipine 40-120 mg/day orally and bed rest (intervention) vs. bed rest alone (controls). | PMR: [0/50 vs. 0/51 in intervention vs. control groups, respectively.] RR not estimable. |
| 7. Cruickshank et al. 1991/1992. [8] | UK.  RCT. N=114 women with singleton pregnancy at 24-39 wks gestation with DBP > 90 mmHg > 24 hr and no proteinuria. | Compared the impact of administration of labetalol 100 mg 2x/day, increased up to 400 mg 3x/day (intervention) vs. no anti-hypertensive (controls). | SBR: [0/51 vs. 0/63 in intervention vs. control groups, respectively.] RR not estimable.  PMR: RR=0.25 (95% CI: 0.01-5.02)**[NS]**  [0/51 vs. 2/63 in intervention vs. control groups, respectively.] |
| 8. Elhassan et al. 2002. [9] | Sudan.  RCT. N=70 primigravid women with pre-eclampsia (BP ≥90/109 mmHg x 2, 6 hr apart plus 2+ proteinuria in dipsticks) at 28-36 weeks' gestation with singleton pregnancy. | Compared impact of administration of methyldopa 750-4000 mg/day (intervention) with no drug treatment (controls). | PMR: RR=0.71 (95% CI: 0.22-2.29)  [4/34 vs. 6/36 in intervention vs. control groups, respectively.]. |
| 9. Ellenbogen et al. 1986 [10] | Israel.  RCT. N=32 women with singleton pregnancy, 27-33 wks gestation with PIH (DBP ≥ 95 mmHg x 2 at least 6 hr apart). | Compared impact of administering pindolol 15 mg/day (intervention) to methyldopa up to 2000 mg/day (controls). | SBR+NMR: RR=1.00 (95% CI: 0.07-14.04)**[NS]**  [1/16 in both arms.] |
| 10. Eloff 1993 [11] | South Africa.  RCT. N=29 women, 29-36 wks gestation with mild-moderate hypertension (DBP 90-110 mmHg). | Compared impact of administration of nifedipine started at 30 mg/day (intervention) vs. methyldopa started at 750 mg/day (controls). Dose adjustments made every second day until control of BP was obtained'. | SBR+NMR: RR=0.31 (95% CI: 0.04-2.65)  [1/15 vs. 3/14 in intervention vs. control groups, respectively] |
| 11. Faneite et al. 1988. [12] | Venezuela.  RCT. N=31 women >14 wks gestation with either chronic hypertension or mild-moderate PIH (BP 140-169/90-109 mmHg x 2 after 5 min rest). | Compared the impact of administering mepindolol 5 mg/day, increased weekly to 10 mg/day (intervention) vs.  methyldopa 250 mg x 2/day increased weekly to 250 mg x 4/day (controls). | SBR+NMR: RR=0.94 (95% CI: 0.06-13.68)**[NS]**  [1/16 vs. 1/15 in intervention vs. control groups, respectively.]. |
| 12. Fidler et al. 1983a. [13] | UK.  RCT. N=100 women with singleton pregnancy and DBP ≥95 mmHg x 2 at least 24 hr apart, or >105 mmHg x 1. UK. | Compared the impact of administration of oxprenolol 80-320 mg 2x/day (intervention) vs. methyldopa 250-1000 mg 3x/day (controls). If BP uncontrolled, hydralazine added to both groups. | SBR+NMR: RR=1.00 (95% CI: 0.06-15.55)**[NS]**  [1/50 in both groups] |
| 13. Freire et al. 1988. [14] | Brazil.  RCT. N=40 pregnant women with chronic hypertension with DBP ≥95 mmHg, without proteinuria. | Compared impact of administering pindolol 10-30 mg/day (intervention) vs. methyldopa 500-2000 mg/day (controls). | SBR+NMR: RR=2.00 (95% CI: 0.41-9.71)**[NS}**  [4/20 vs. 2/20 in intervention vs. control groups, respectively.] |
| 14. Gallery et al. 1985 [15] | Australia.  RCT. N=183 women with singleton pregnancy and mild hypertension (DBP ≥90 mmHg x 2 24 hr apart, or DBP ≥95 mmHg x 2, 12 hr apart, or DBP ≥100 mmHg x 2, 8 hr apart). | Compared impact of administering oxprenolol 40-320 mg 2x/day (intervention) vs. methyldopa 250 mg 2x/day-1000 mg 3x/day. All subjects with uncontrolled blood pressure also received hydralazine. | SBR+NMR: RR=0.23 (95% CI=0.03-1.99)**[NS]**  [1/96 vs. 4/87 in intervention vs. control groups, respectively.] |
| 15. Gruppo di Studio Ipertensione in Gravidanza 1998 [16]. | Italy.  RCT. N=283 women at 12-34 weeks' gestation, with mild-moderate hypertension (DBP 90-110 mmHg x 2, 4 hours apart). | Compared impact of administration of slow-release nifedipine 20-80 mg 2x/day orally (intervention) to no anti-hypertensive (controls). | SBR: RR=0.47 (95% CI: 0.25- 8.63)**[NS]**  [3/132 vs. 2/129 in intervention vs. control groups, respectively.] |
| 16. Högstedt et al. 1985 [17]. | Sweden.  RCT. N=168 women in antenatal ward with singleton pregnancy at < 37 wks, DBP ≥ 90 mmHg x 2, no proteinuria. | Compared the impact of administration of metoprolol 50-200 mg/day + hydralazine 50-300 mg/day (intervention) vs. no anti-hypertensive (controls). | SBR: RR=2.86 (95% CI: 0.30-26.95)**[NS]**  [3/86 vs. 1/82 in the intervention vs. control groups, respectively.] |
| 17. Jannet et al. 1994 [18] | France.  RCT. N=100 women with singleton pregnancy, >20 wks gestation and mild-moderate hypertension (BP ≥140/90 mmHg x 2). | Compared impact of administering nicardipine 20 mg 3x/day (intervention) to. metroprolol (slow release) 200 mg/day (controls). | SBR+NMR: RR=1.00 (95% CI: 0.06-15.55) **[NS]**  [1/50 in both groups] |
| 18. Kahhale et al. 1985 [19]. | Brazil.  RCT. N=100 women with chronic hypertension. | Compared impact of administering pindolol 10-30 mg/day (intervention) vs. no treatment (controls). | SBR: RR=2.00 (95% CI: 0.19-21.31)  [2/47 vs. 1/47 in intervention vs. control groups, respectively.] |
| 19. Lamming et al. 1980 [20]. | UK.  RCT. N=26 women < 38 wks gestation with PIH and no contraindication to beta blockers. | Compared the impact of administration of labetalol 400-800 mg/day (intervention) vs. methyldopa 750-1500 mg/day (controls). | SBR+NMR: [0/14 vs. 0/12 in intervention vs. control groups, respectively.] RR not estimable. |
| 20. Lardoux et al. 1988 [21] | France.  RCT. N=63 women, 7-36 wks gestation with DBP >90 mmHg x 2, 8 days apart). | Compared impact of administering acebutolol 400-1200 mg (intervention #1), or labetalol 400-1200 mg (intervention #2) to methyldopa 500-1500 mg (controls). | SBR+NMR: RR=0.50 (95% CI: 0.03-7.60) **[NS]**  [1/42 vs. 1/21 in both intervention groups vs. control groups, respectively.] |
| 21. Leather et al. 1968 [22]. | UK.  RCT. N=100 pregnant women with DBP ≥ 90 mmHg or more x 2, 48 hr apart. | Compared the impact of administration of methyldopa 250-1,000 mg 2x/day + bendrofluazide 5-10 mg/day (intervention) vs. no treatment (controls). | PMR: RR=0.87 (95% CI: 0.30- 2.50)**[NS]**  [6/52 vs. 6/45 in intervention vs. control groups, respectively.] |
| 22. Livingstone et al. 1983 [23] | Australia.  RCT. N=28 women in ANC clinics with mild-to-moderate PIH (BP≥140/90 mmHg x 2 ≥24 hr apart). | Compared impact of administering propranolol 30-160 mg/day (intervention) vs. methyldopa 500-1000 mg/day (controls). | SBR+NMR: [0/14 in both intervention and control groups, RR not estimable] |
| 23. Nascimento 2000a [24]. | Brazil.  RCT. N=199 singleton pregnant women with mild/moderate chronic hypertension. | Compared impact of administering verapamil 240 mg 3x/day (intervention) vs. placebo (controls). | SBR: RR=0.35 (95% CI: 0.01- 8.43) **[NS]**  [0/90 vs. 1/94 in intervention vs. control groups, respectively.] |
| 24. Neri et al. 1999 [25]. | Italy.  RCT. N=36 women with singleton pregnancy, gestation > 24 wks and PIH or pre-eclampsia (BP 140/90 mmHg or more, pre-eclampsia if proteinuria > 300 mg/24 hr). | Compared impact of administration of 1) transdermal glyceryl trinitrate 10 mg continuously 24 hr/day (intervention #1), or 2) transdermal glyceryl trinitrate 10 mg intermittently for 16 hr/day (intervention #2) to 3) nifedipine 40 mg/day orally (controls). | SBR+NMR: [0/24 vs. 0/12 in treatment vs. control groups, respectively.]  RR not estimable. |
| 25. Odendaal et al. 1991 [26] | South Africa.  RCT. N=32 women, 12-30 wks gestation with a singleton pregnancy and BP ≥ 140/90 mmHg x 2 ≥6 hr apart, no proteinuria, no anti-hypertensive therapy and no other drug treatment. | Compared impact of administration of prazosin 1-5 mg x 3/day (intervention) to placebo (controls). | SBR: RR=0.56 (95% CI: 0.06- 4.76)**[NS]**  [1/12 vs. 3/20 in intervention vs. control groups, respectively.] |
| 26. Oumachigui et al. 1992 [27]. | India.  RCT. N=30 primigravid women 24-37 wks gestation with mild-moderate PIH (BP ≥140/90 mmHg x 2, 6 hr apart). | Compared impact of administering metoprolol 50-150 mg 2x/day (intervention) vs.methyldopa 250 mg 3x/day, increased to 2000 mg/day (controls). | SBR+NMR: RR= 0.31 (95% CI: 0.04-2.68) **[NS]**  [1/16 vs. 3/15 in intervention vs. control groups, respectively] |
| 27. Paran et al. 1995 [28] | Israel.  RCT. N=51 women with BP 140-160/95-110 mmHg. | Compared the impact of administering 1) hydralazine 60-200 mg/day + propranolol 40-120 mg/day; or 2) hydralazine 60-200 mg/day + pindolol 5-15 mg/day to 3) controls given hydralazine 60-200 mg/day. | PMR: [0/36 vs. 0/15 in both intervention groups vs. controls, respectively.] RR not estimable. |
| 28. Pickles et al. 1992 [29]. | UK.  RCT. 1989. N=152 women from antenatal wards at 20-38 wks gestation with SBP 140-160 mmHg and DBP 90-105 mmHg x 2, 24 hr apart, and no proteinuria. | Compared the impact of administration of labetalol 100-200 mg x 3/day (intervention) vs. placebo (controls). | PMR: [0/70 vs. 0/74 in intervention vs. control groups, respectively.] RR not estimable. |
| 29. Plouin et al. 1990 [30] | Caribbean Islands.  RCT. N=155 women with singleton pregnancy, 20-36 wks gestation, DBP <85 mmHg x 2 before 20 wks and >84 mmHg after 20 wks. | Compared impact of administering oxprenolol 160-320 mg 2x/day (intervention) vs. controls (placebo). Hydralazine 50-100 mg added if necessary to keep DBP < 86 mmHg. | SBR: RR=1.95 (95% CI: 0.18-21.05) **[NS]**  [2/78 vs.1/76 in intervention vs. control groups, respectively.] |
| 30. Plouin et al., the Labetolol Methyldopa Study Group 1988 [31]. | France.  RCT. N=188 women with singleton pregnancy at 12-34 wks gestation, booked < 20 wks and DBP ≥90 mmHg. | Compared impact of administering labetalol 200-600 mg 2x/day (intervention) vs. methyldopa 250-750 mg 2x/day (controls). | SBR+NMR: RR=0.23 (95% CI: 0.03-2.05) **[NS]**  [1/91 vs. 4/85 in intervention vs. control groups, respectively] |
| 31. Redman et al. 1976 [32]. | UK.  RCT. N=247 women with BP ≥ 140/90 mmHg if <28 wks gestation, or ≥150/95 mmHg if >28 wks gestation x 2 24 hr apart. | Compared the impact of administration of methyldopa 750-4000 mg/day (intervention) vs. no anti-hypertensive (controls). Hydralazine given for severe hypertension. | SBR: RR=0.36 (95% CI: 0.04- 3.38)**[NS]**  [1/117 vs. 3/125 in intervention vs. control groups, respectively] |
| 32. Rosenfeld et al. 1986a [33] | Israel.  RCT. N=44 women < 37 wks gestation with BP ≥ 150/90 mmHg x 2 at least 24 hr apart. | Compared impact of administering hydralazine 50-100 mg/day + pindolol 10-25 mg/day (in 2 daily doses) (intervention) vs. hydralazine 50-100 mg/day (in 2 daily doses) (controls). | PMR: [0/21 vs. 0/23 in intervention vs. control groups, respectively.] RR not estimable. |
| 33. Rubin et al. 1983 [34]. | UK.  RCT. N=120 women with PIH in 3rd trimester admitted for bed rest, SBP 140-170 mmHg and DBP 90-110 mmHg x 2, 24 hr apart. | Compared the impact of administration of atenolol 100-200 mg/day (intervention) vs. placebo (controls). | SBR: RR=0.50 (95% CI: 0.05- 5.37)**[NS]**  [1/60 vs. 2/60 in intervention vs. control groups, respectively.] |
| 34. Sibai et al. 1987 [35] | USA  RCT. N=200 primigravid women hospitalised at 26-35 wks gestation with SBP 140-160 mmHg and DBP 90-110 mmHg, proteinuria > 0.3 g/L and uric acid > 4.6 mg/dL. | Compared the impact of hospitalisation + labetalol 300 mg/day, increased every few days to max 2400 mg/day (intervention), vs. hospitalisation alone (controls). | SBR: [0/102 vs. 0/103 in intervention vs. control groups, respectively.] RR not estimable. |
| 35. Sibai et al. 1990 [36]. | USA.  RCT. N=300 women in antenatal ward with chronic mild-moderate hypertension at 6-13 wks gestation. All had chronic hypertension before pregnancy and no associated medical complications. | Compared the impact of administering 1) methyldopa 750-4000 mg/day (intervention #1), or 2) labetalol 300-2400 mg/day (intervention #2) vs. no anti-hypertensive (controls). | SBR: RR=0.51 (95% CI: 0.03- 7.99)**[NS]**  [1/94 vs. 1/98 in both intervention arms vs. control groups, respectively.] |
| 36. Sibai et al. 1992 [37]. | USA.  RCT. N=200 primigravid women 26-36 wks gestation with SBP 140-160 mmHg and/or DBP 90-110 mmHg 24 hr after hospitalisation, proteinuria > 300 mg/24 hr, and/or uric acid > 6 mg/dL. | Compared the impact of administering nifedipine 40-120 mg/day (intervention) vs. bed rest alone (controls). | SBR: [0/99 vs. 0/101 in intervention vs. control groups, respectively.] RR not estimable. |
| 37. Thorley 1984 [38]. | UK.  RCT. N=60 women 18-36 wks gestation with undefined hypertension. | Compared the impact of administration of atenolol 100 mg/day (intervention) vs. methyldopa 250 mg 3x/day (controls). | SBR+NMR: [0/30 in both groups.] RR not estimable. |
| 38. Voto et al.1985 [39] | Argentina.  RCT. N=60 women with SBP ≥160 mmHg and/or DBP ≥100 mmHg x 2, 24 hr apart, with or without proteinuria at trial entry. | Compared impact of administering atenolol 50-250 mg/day (intervention) vs. methyldopa 750-2000 mg/day (controls). | SBR+NMR: RR=2.00 (95% CI: 0.19-20.90) **[NS]**  [2/30 vs. 1/30 in intervention vs. control groups, respectively.] |
| 39. Voto et al. 1987 [40]. | Argentina.  RCT. N=20 women with SBP > 159 mmHg and/or DBP > 99 mmHg x 2, 24 hr apart, +/- proteinuria. | Compared impact of administering ketanserin 20-80 mg/day (intervention) vs. methyldopa 500-2000 mg/day (controls). | SBR+NMR: RR=3.00 (95% CI: 0.14-65.90)**[NS]**  [1/10 vs. 0/10 in intervention vs. control groups, respectively.] |
| 40. Walker et al. 1982 [41]. | UK.  RCT. N=126 women with either chronic hypertension or PIH, and DBP > 95 mmHg if < 20 wks or 95-109 mmHg if > 20 wks. | Compared the impact of administration of labetalol 100 mg 2x/day, increased to maximum of 1200 mg/day (intervention) vs. no anti-hypertensive (controls). If BP uncontrolled, hydralazine 25 mg x 3/day, increased to maximum of 200 mg/day. | SBR: [0/64 vs. 0/62 in intervention vs. control groups, respectively.] RR not estimable. |
| 41. Wichman et al. 1984 [42] | Sweden.  RCT. N=52 women. | Compared impact of administration of metoprolol 100-200 mg 2x/day (intervention) vs. placebo 2x/day (controls). | SBR: [0/26 in both arms.] RR not estimable. |
| 42. Wide-Swensson et al. 1995 [43]. | Sweden.  RCT. N=118 women at 26-37 weeks, with singleton pregnancy and DBP 95-110 mmHg. | Compared the impact of administration of slow-release isradipine 5 mg 2x/day (intervention) vs. placebo 2x/day (controls). | SBR: [0/54 vs. 0/57 in intervention vs. control groups, respectively.] RR not estimable. |
| 43. Weitz et al. 1987a [44] | USA.  RCT. N=25 women <34 weeks' gestation, singleton pregnancy with BP 140/90 mmHg x 2 at least 6 hr apart and no proteinuria. Presumed chronic hypertension. | Compared the impact of administering methyldopa 750 mg x 3/day to 2000 mg x 4/day (intervention) vs. placebo (control). If severe pre-eclampsia, hydralazine or MgSO4 added. | PMR: [0/13 vs. 0/12 in intervention vs. control groups, respectively]. RR not estimable |

References

1. Abalos E, Duley L, Steyn DW, Henderson-Smart DJ: **Antihypertensive drug therapy for mild to moderate hypertension during pregnancy**. *Cochrane Database Syst Rev* 2007(1):CD002252.

2. Blake S, MacDonald D: **The prevention of the maternal manifestations of pre-eclampsia by intensive antihypertensive treatment**. *Br J Obstet Gynaecol* 1991, **98**(3):244-248.

3. Borghi C, Immordino V, Degli Esposti D, Boschi S, Cassani A, Bentivenga C, al. e: **Comparison between nifedipine-gits and methyldopa on blood pressure control, utero-placental hemodynamic and fetal outcome in patients with pre-eclampsia**. *Hypertension in Pregnancy* 2000, **19**:P8.

4. Bott-Kanner G, Hirsch M, Friedman S, Boner G, Ovadia J, Merlob P, al. e: **Antihypertensive therapy in the management of hypertension in pregnancy - a clinical double-blind study of pindolol**. *Clinical and Experimental Hypertension* 1992, **B11**:207-220.

5. Butters L, Kennedy S, Rubin PC: **Atenolol in essential hypertension during pregnancy**. *BMJ* 1990, **301**(6752):587-589.

6. Casavilla F, Vega HR: **Prospective and randomized study on mepindolol and alpha-methyldopa efficacy in arterial hypertension (AH) treatment during pregnancy.** In: *World Congress of Gynecology and Obstetrics: 1988 October 23-28.; Brazil.*; 1988 October 23-28.

7. Catalano D, Ercolano S, Pollio F, Ascione L, Russo C, De Santi B, al. e: **Evaluation of nifedipine monotherapy in the management of pregnancy hypertension [Valuazione della monoterapia con nifedipina nel management della gestosi EPH]**. *Giornale Italiano Di Ostetricia e Ginecologia* 1997, **6**:373-375.

8. Cruickshank DJ, Robertson AA, Campbell DM, MacGillivray I: **Maternal obstetric outcome measures in a randomised controlled study of labetalol in the treatment of hypertension in pregnancy**. *Clinical and Experimental Hypertension;* 1991, **B10**:333-344.

9. Elhassan EM, Mirghani OA, Habour AB, Adam I: **Methyldopa versus no drug treatment in the management of mild pre-eclampsia**. *East Afr Med J* 2002, **79**(4):172-175.

10. Ellenbogen A, Jaschevatzky O, Davidson A, Anderman S, Grunstein S: **Management of pregnancy-induced hypertension with pindolol--comparative study with methyldopa**. *Int J Gynaecol Obstet* 1986, **24**(1):3-7.

11. Eloff W: **The use of nifedipine vs methyldopa in mild to moderate pregnancy associated hypertension.** In: *Proceedings of the 12th Conference on Priorities in Perinatal Care: 1993.; South Africa.*; 1993.: 130-133.

12. Faneite PJ, Gonzalez X, Salazar G: **Evaluation of antihypertensives in pregnancy: prospective randomized study of mepindolol and alpha methyldopa [Evaluación de antihipertensivos en embarazadas: Mepindolol y Alfametildopa. Estudio Prospectivo y randomizado]**. *Revista de Obstetricia y Ginecologia de Venezuela* 1988, **48**:139-143.

13. Fidler J, Smith V, Fayers P, De Swiet M: **Randomised controlled comparative study of methyldopa and oxprenolol in treatment of hypertension in pregnancy**. *Br Med J (Clin Res Ed)* 1983, **286**(6382):1927-1930.

14. Freire S, de França LA, Rau de Almeida Callou M, Alves de Oliveira JE, Barbosa Filho J: **Comparative study with pindolol and methyldopa in pregnant women with chronic hypertension [Estudo comparativo com pindolol e metildopa em gestantes com hipertensão arterial crônica]**. *Jornal Brasileiro de Ginecologia* 1988, **98**:157-160.

15. Gallery EDM, Ross MR, Gyory AZ: **Antihypertensive treatment in pregnancy: analysis of different responses to oxprenolol and methyldopa**. *BMJ* 1985, **291**:563-566.

16. **Nifedipine versus expectant management in mild to moderate hypertension in pregnancy. Gruppo di Studio Ipertensione in Gravidanza**. *Br J Obstet Gynaecol* 1998, **105**(7):718-722.

17. Hogstedt S, Lindeberg S, Axelsson O, Lindmark G, Rane A, Sandstrom B, Lindberg BS: **A prospective controlled trial of metoprolol-hydralazine treatment in hypertension during pregnancy**. *Acta Obstet Gynecol Scand* 1985, **64**(6):505-510.

18. Jannet D, Carbonne B, Sebban E, Milliez J: **Nicardipine versus metoprolol in the treatment of hypertension during pregnancy: a randomized comparative trial**. *Obstet Gynecol* 1994, **84**(3):354-359.

19. Kahhale S, Zugaib M, Carrara W, Paula FJ, Sabbaga E, Neme B: **Comparative study of chronic hypertensive pregnant women treated and non-treated with pindolol [Estudio comparativo de gestantes hipertensas crônicas tratadas e näo tratadas com betabloqueador pindolol]**. *Ginecologia e Obstetrícia Brasileiras;* 1985, **8**:85-89.

20. Lamming GD, Broughton Pipkin F, Symonds EM: **Comparison of the alpha and beta blocking drug, labetalol, and methyl dopa in the treatment of moderate and severe pregnancy-induced hypertension**. *Clin Exp Hypertens* 1980, **2**(5):865-895.

21. Lardoux H, Blazquez G, Leperlier E, Gerard J: **Randomized and comparative study of methyldopa (MD), acebutolol (ACE) and labetalol for the treatment of moderate hypertension during pregnancy (HDP)**. *Archives des Maladies du Coeur* 1988, **91**:137-140.

22. Leather HM, Humphreys DM, Baker P, Chadd MA: **A controlled trial of hypotensive agents in hypertension in pregnancy**. *Lancet* 1968, **2**(7566):488-490.

23. Livingstone I, Craswell PW, Bevan EB, Smith MT, Eadie MJ: **Propranolol in pregnancy three year prospective study**. *Clin Exp Hypertens B* 1983, **2**(2):341-350.

24. Nascimento D: **Avaliaçäo do uso do verapamil em gestantes com formas näo graves de doença hipertensiva vascular crônica [Evaluation of the use of Verapamil with non-serious form of chronic vascular hypertension disease during pregnancy]**. Brazil: Facultade Evangélica de Medicina do Paraná; 2000.

25. Neri I, Valensise H, Facchinetti F, Menghini S, Romanini C, Volpe A: **24-hour ambulatory blood pressure monitoring: a comparison between transdermal glyceryl-trinitrate and oral nifedipine**. *Hypertens Pregnancy* 1999, **18**(1):107-113.

26. Odendaal HJ, Schabort I, Pattinson RC: **Prazosin for the treatment of hypertension in pregnancy: a randomized control trial**. In: *Oxford Database of Perinatal Trials 1991; Vol Version 12, Disk Issue 6* Edited by I C. Oxford: Oxford University Press; Autumn 1991.

27. Oumachigui A, Verghese M, Balachander J: **A comparative evaluation of metoprolol and methyldopa in the management of pregnancy induced hypertension**. *Indian Heart J* 1992, **44**(1):39-41.

28. Paran E, Holzberg G, Mazor M, Zmora E, Insler V: **Beta-adrenergic blocking agents in the treatment of pregnancy-induced hypertension**. *Int J Clin Pharmacol Ther* 1995, **33**(2):119-123.

29. Pickles CJ, Broughton Pipkin F, Symonds EM: **A randomised placebo controlled trial of labetalol in the treatment of mild to moderate pregnancy induced hypertension**. *Br J Obstet Gynaecol* 1992, **99**(12):964-968.

30. Plouin PF, Breart G, Llado J, Dalle M, Keller ME, Goujon H, Berchel C: **A randomized comparison of early with conservative use of antihypertensive drugs in the management of pregnancy-induced hypertension**. *Br J Obstet Gynaecol* 1990, **97**(2):134-141.

31. Plouin PF, Breart G, Maillard F, Papiernik E, Relier JP: **Comparison of antihypertensive efficacy and perinatal safety of labetalol and methyldopa in the treatment of hypertension in pregnancy: a randomized controlled trial**. *Br J Obstet Gynaecol* 1988, **95**(9):868-876.

32. Redman CW: **Fetal outcome in trial of antihypertensive treatment in pregnancy**. *Lancet* 1976, **2**(7989):753-756.

33. Rosenfeld J, Bott-Kanner G, Boner G, Nissenkorn A, Friedman S, Ovadia J, Merlob P, Reisner S, Paran E, Zmora E *et al*: **Treatment of hypertension during pregnancy with hydralazine monotherapy or with combined therapy with hydralazine and pindolol**. *Eur J Obstet Gynecol Reprod Biol* 1986, **22**(4):197-204.

34. Rubin PC, Butters L, Clark DM, Reynolds B, Sumner DJ, Steedman D, Low RA, Reid JL: **Placebo-controlled trial of atenolol in treatment of pregnancy-associated hypertension**. *Lancet* 1983, **1**(8322):431-434.

35. Sibai BM, Gonzalez AR, Mabie WC, Moretti M: **A comparison of labetalol plus hospitalization versus hospitalization alone in the management of preeclampsia remote from term**. *Obstet Gynecol* 1987, **70**(3 Pt 1):323-327.

36. Sibai BM, Mabie WC, Shamsa F, Villar MA, Anderson GD: **A comparison of no medication versus methyldopa or labetalol in chronic hypertension during pregnancy**. *Am J Obstet Gynecol* 1990, **162**(4):960-966; discussion 966-967.

37. Sibai BM, Barton JR, Akl S, Sarinoglu C, Mercer BM: **A randomized prospective comparison of nifedipine and bed rest versus bed rest alone in the management of preeclampsia remote from term**. *Am J Obstet Gynecol* 1992, **167**(4 Pt 1):879-884.

38. Thorley K: **Randomised trial of atenolol and methyl dopa in pregnancy related hypertension**. *Clinical and Experimental Hypertension;* 1984, **133**:168.

39. Voto LS, Lapidus AM, Neira J, Margulies M: **Treatment of hypertension during pregnancy: Atenolol versus Methyldopa [Tratamiento de la hipertensión en el embarazo: Atenolol versus Alfa Metildopa]**. *Obstetricia y Ginecología Latino-Americanas;* 1985, **43**:335-341.

40. Voto LS, Zin C, Neira J, Lapidus AM, Margulies M: **Ketanserin versus alpha-methyldopa in the treatment of hypertension during pregnancy: a preliminary report**. *J Cardiovasc Pharmacol* 1987, **10 Suppl 3**:S101-103.

41. Walker JJ, Crooks A, Erwin L, Calder AA: **Labetalol in pregnancy-induced hypertension: fetal and maternal effects**. In: *International Congress Series 591.* Edited by Symonds EM RA: Excerpta Medica; 1982: 148-160.

42. Wichman K, Ryden G, Karlberg BE: **A placebo controlled trial of metoprolol in the treatment of hypertension in pregnancy**. *Scand J Clin Lab Invest Suppl* 1984, **169**:90-95.

43. Wide-Swensson DH, Ingemarsson I, Lunell NO, Forman A, Skajaa K, Lindberg B, Lindeberg S, Marsal K, Andersson KE: **Calcium channel blockade (isradipine) in treatment of hypertension in pregnancy: a randomized placebo-controlled study**. *Am J Obstet Gynecol* 1995, **173**(3 Pt 1):872-878.

44. Weitz C, Khouzami V, Maxwell K, Johnson JW: **Treatment of hypertension in pregnancy with methyldopa: a randomized double blind study**. *Int J Gynaecol Obstet* 1987, **25**(1):35-40.
