# Supplementary material for: Reducing stillbirths: prevention and management of medical disorders and infections during pregnancy
Source: BMC Pregnancy Childbirth. 2009 May 7;9(Suppl 1):S4. doi: 10.1186/1471-2393-9-S1-S4 (PMC2679410; doi:10.1186/1471-2393-9-S1-S4)
Supplement: Additional file 4 — Web Table 4. Component studies in Magee et al. 2003 meta-analysis: impact of anti-hypertensive drugs for chronic maternal hypertension. Component studies in Magee et al. 2003 meta-analysis reporting impact on stillbirths/perinatal mortality [file 1471-2393-9-S1-S4-S4.doc]

**Web Table 4. Component studies in Magee et al. 2003 [1] meta-analysis: impact of anti-hypertensive drugs for chronic maternal hypertension**

| Source | Location and Type of Trial | Intervention | | Stillbirths/Perinatal Outcomes | |
| --- | --- | --- | --- | --- | --- |
| ***Beta-blocker versus placebo/no beta-blocker*** | | | | | |
| 1. Bott-Kanner et al. 1992 [2] | Israel.  RCT. N=60 women <35 wks gestation with DBP 85-99 mmHg x 2, 12 hr apart, and no treatment for hypertension during this pregnancy. | Assessed impact of administering pindolol 5 mg 2x/day (intervention). If DBP in intervention group still ≥ 85 mmHg on day 3, increased to 5 mg x 3/day, if no response next day, increased to 10 mg x 2/day. If DBP 100-109 mmHg x2 or > 110 mmHg x1, hydralazine added for pindolol group. Control group given placebo unless DBP elevated; pindolol given first, followed by hydralazine if DBP > 100 mmHg. | | PMR: RR=3.00 (95% CI: 0.13-70.83)**[NS]**  [1/30 vs. 0/30 in intervention vs. control groups, respectively.] | |
| 2. Butters et al. 1990 [3] | Scotland.  RCT. N=33 women 12-24 wks gestation with SBP 140-170 mmHg and DBP 90-110 mmHg x 2, 24 hr apart. | Compared the impact of administration of atenolol 50-200 mg/day (intervention) vs. placebo (controls). | | PMR: RR=2.81 (95% CI: 0.12-63.83)  [1/15 vs. 0/14 in intervention vs. control groups, respectively.]. | |
| 3. Cruickshank et al. 1991/1992 [4]. | Scotland.  RCT. N=114 women with singleton pregnancy at 24-39 wks gestation with DBP > 90 mmHg > 24 hr and no proteinuria. | Compared the impact of administration of labetalol 100 mg 2x/day, increased up to 400 mg 3x/day (intervention) vs. no anti-hypertensive (controls). | | PMR: RR=0.25 (95% CI: 0.01-5.02)**[NS]**  [0/51 vs. 2/63 in intervention vs. control groups, respectively.] | |
| 4. Högstedt et al. 1985 [5]. | Sweden.  RCT. N=168 women in antenatal ward with singleton pregnancy at < 37 wks, DBP ≥ 90 mmHg x 2, no proteinuria. | Compared the impact of administration of metoprolol 50-200 mg/day + hydralazine 50-300 mg/day (intervention) vs. no anti-hypertensive (controls). | | PMR: RR=2.86 (95% CI: 0.30-26.95)**[NS]**  [3/86 vs. 1/82 in the intervention vs. control groups, respectively.] | |
| 5. Paran et al. 1995 [6] | Israel.  RCT. N=51 women with BP 140-160/95-110 mmHg. | Compared the impact of administering 1) hydralazine 60-200 mg/day + propranolol 40-120 mg/day; or 2) hydralazine 60-200 mg/day + pindolol 5-15 mg/day to 3) controls given hydralazine 60-200 mg/day. | | PMR: [0/36 vs. 0/15 in both intervention groups vs. controls, respectively.] RR not estimable. | |
| 6. Pickles et al. 1989 [7]. | UK.  RCT. 1989. N=152 women from antenatal wards at 20-38 wks gestation with SBP 140-160 mmHg and DBP 90-105 mmHg x 2, 24 hr apart, and no proteinuria. | Compared the impact of administration of labetalol 100-200 mg x 3/day (intervention) vs. placebo (controls). | | PMR: [0/70 vs. 0/74 in intervention vs. control groups, respectively]. RR not estimable. | |
| 7. Plouin et al. 1990 [8] | Caribbean Islands.  RCT. N=155 women with singleton pregnancy, 20-36 wks gestation, DBP <85 mmHg x 2 before 20 wks and >84 mmHg after 20 wks. | Compared impact of administering oxprenolol 160-320 mg 2x/day (intervention) vs. controls (placebo). Hydralazine 50-100 mg added if necessary to keep DBP < 86 mmHg. | | PMR: RR=0.65 (95% CI: 0.11-3.78) **[NS]**  [2/78 vs. 3/76 in intervention vs. control groups, respectively.] | |
| 8. Rosenfeld et al. 1986a [9] | Israel.  RCT. N=44 women < 37 wks gestation with BP ≥ 150/90 mmHg x 2 at least 24 hr apart. | Compared impact of administering hydralazine 50-100 mg/day + pindolol 10-25 mg/day (in 2 daily doses) (intervention) vs. hydralazine 50-100 mg/day (in 2 daily doses) (controls). | | PMR: [0/23 vs. 0/21 in intervention vs. control groups, respectively.] RR not estimable. | |
| 9. Rubin et al. 1983 [10]. | Scotland.  RCT. N=120 women with PIH in 3rd trimester admitted for bed rest, SBP 140-170 mmHg and DBP 90-110 mmHg x 2, 24 hr apart. | Compared the impact of administration of atenolol 100-200 mg/day (intervention) vs. placebo (controls). | | PMR: RR=0.50 (95% CI: 0.05- 5.37)**[NS]**  [1/60 vs. 2/60 in intervention vs. control groups, respectively.] | |
| 10. Sibai et al. 1987 [11] | USA  RCT. N=200 primigravid women hospitalised at 26-35 wks gestation with SBP 140-160 mmHg and DBP 90-110 mmHg, proteinuria > 0.3 g/L and uric acid > 4.6 mg/dL. | Compared the impact of hospitalisation + labetalol 300 mg/day, increased every few days to max 2400 mg/day (intervention), vs. hospitalisation alone (controls). | | PMR: RR = 3.09 (95% CI: 0.13 – 75.03] **[NS]**.  [1/94 vs. 0/97 in intervention vs. control groups, respectively.] RR not estimable. | |
| 11. Sibai et al. 1990 [12]. | USA.  RCT. N=300 women in antenatal ward with chronic mild-moderate hypertension at 6-13 wks gestation. All had chronic hypertension before pregnancy and no associated medical complications. | Compared the impact of administering 1) methyldopa 750-4000 mg/day (intervention #1), or 2) labetalol 300-2400 mg/day (intervention #2) vs. no anti-hypertensive (controls). | | PMR: RR=1.05 (95% CI: 0.07- 16.47)**[NS]**  [1/86 vs. 1/90 in both intervention arms vs. control groups, respectively.] | |
| 12. Walker et al. 1982 [13]. | Scotland.  RCT. N=126 women with either chronic hypertension or PIH, and DBP > 95 mmHg if < 20 wks or 95-109 mmHg if > 20 wks. | Compared the impact of administration of labetalol 100 mg 2x/day, increased to maximum of 1200 mg/day (intervention) vs. no anti-hypertensive (controls). If BP uncontrolled, hydralazine 25 mg x 3/day, increased to maximum of 200 mg/day. | | PMR: [0/64 vs. 0/62 in intervention vs. control groups, respectively.] RR not estimable. | |
| 13. Wichman et al. 1984 [14] | Sweden.  RCT. N=52 women. | Compared impact of administration of metoprolol 100-200 mg 2x/day (intervention) vs. placebo 2x/day (controls). | | PMR: RR = 0.33 (95% CI: 0.01 – 7.82) **[NS]**.  [0/26 vs. 1/26 in intervention and control groups, respectively]. | |
| ***Beta-blocker versus methyldopa.*** | | | | | |
| 14. Casavilla and Vega 1988 [15]. | Argentina.  RCT. N=36 women >14 wks gestation with BP ≥140/90 mmHg and ≤170/110 mmHg. Argentina. | | Compared impact of administering mepindolol, increasing weekly doses, from 5-10 mg/day (intervention) vs. methyldopa, increasing weekly doses from 500-2000 mg/day (controls). | | PMR: RR=1.00 (95% CI: 0.07-14.79) **[NS]**  [1/18 vs. 1/18 in intervention vs. control groups, respectively]. |
| 15. Ellenbogen et al. 1986 [16] | Israel.  RCT. N=32 women with singleton pregnancy, 27-33 wks gestation with PIH (DBP ≥ 95 mmHg x 2 at least 6 hr apart). | | Compared impact of administering pindolol 15 mg/day (intervention) to methyldopa up to 2000 mg/day (controls). | | PMR: RR=1.00 (95% CI: 0.07-14.04)**[NS]**  [1/16 in both arms.] |
| 16. Faneite et al. 1988 [17]. | Venezuela.  RCT. N=31 women >14 wks gestation with either chronic hypertension or mild-moderate PIH (BP 140-169/90-109 mmHg x 2 after 5 min rest). | | Compared the impact of administering mepindolol 5 mg/day, increased weekly to 10 mg/day (intervention) vs.  methyldopa 250 mg x 2/day increased weekly to 250 mg x 4/day (controls). | | PMR: RR=0.94 (95% CI: 0.06-13.68)**[NS]**  [1/16 vs. 1/15 in intervention vs. control groups, respectively.]. |
| 17. Fidler et al. 1983 [18]. | UK.  RCT. N=100 women with singleton pregnancy and DBP ≥95 mmHg x 2 at least 24 hr apart, or >105 mmHg x 1. UK. | | Compared the impact of administration of oxprenolol 80-320 mg 2x/day (intervention) vs. methyldopa 250-1000 mg 3x/day (controls). If BP uncontrolled, hydralazine added to both groups. | | PMR: RR=1.00 (95% CI: 0.06-15.55) **[NS]**.  [1/50 in both groups]. |
| 18. Gallery et al. 1985 [19]. | Australia.  RCT. N=183 women with singleton pregnancy and mild hypertension (DBP ≥90 mmHg x 2 24 hr apart, or DBP ≥95 mmHg x 2, 12 hr apart, or DBP ≥100 mmHg x 2, 8 hr apart). | | Compared impact of administering oxprenolol 40-320 mg 2x/day (intervention) vs. methyldopa 250 mg 2x/day-1000 mg 3x/day. All subjects with uncontrolled blood pressure also received hydralazine. | | PMR: RR=0.30 (95% CI=0.03-2.85) **[NS]**.  [1/96 vs. 3/87 in intervention vs. control groups, respectively.] |
| 19. Lamming et al. 1980 [20]. | UK.  RCT. N=26 women < 38 wks gestation with PIH and no contraindication to beta blockers. | | Compared the impact of administration of labetalol 400-800 mg/day (intervention) vs. methyldopa 750-1500 mg/day (controls). | | PMR: [0/14 vs. 0/12 in intervention vs. control groups, respectively]. RR not estimable. |
| 20. Lardoux et al. 1988 [21] | France.  RCT. N=63 women, 7-36 wks gestation with DBP >90 mmHg x 2, 8 days apart). | | Compared impact of administering acebutolol 400-1200 mg (intervention #1), or labetalol 400-1200 mg (intervention #2) to methyldopa 500-1500 mg (controls). | | PMR: RR=0.50 (95% CI: 0.03-7.60) **[NS]**  [1/42 vs. 1/21 in both intervention groups vs. control groups, respectively.] |
| 21. Livingstone et al. 1983 [22] | Australia.  RCT. N=28 women in ANC clinics with mild-to-moderate PIH (BP≥140/90 mmHg x 2 ≥24 hr apart). | | Compared impact of administering propranolol 30-160 mg/day (intervention) vs. methyldopa 500-1000 mg/day (controls). | | PMR: [0/14 in both intervention and control groups, RR not estimable] |
| 22. Oumachigui et al. 1992 [23]. | India.  RCT. N=30 primigravid women 24-37 wks gestation with mild-moderate PIH (BP ≥140/90 mmHg x 2, 6 hr apart). | | Compared impact of administering metoprolol 50-150 mg 2x/day (intervention) vs.methyldopa 250 mg 3x/day, increased to 2000 mg/day (controls). | | PMR: RR= 0.31 (95% CI: 0.04-2.68) **[NS]**  [1/16 vs. 3/15 in intervention vs. control groups, respectively] |
| 23. Plouin et al., the Labetolol Methyldopa Study Group 1988 [24]. | France.  RCT. N=188 women with singleton pregnancy at 12-34 wks gestation, booked < 20 wks and DBP ≥90 mmHg. | | Compared impact of administering labetalol 200-600 mg 2x/day (intervention) vs. methyldopa 250-750 mg 2x/day (controls). | | PMR: RR=0.23 (95% CI: 0.03-2.05) **[NS]**  [1/91 vs. 4/85 in intervention vs. control groups, respectively] |
| 24. Redman 1978 [25]. | UK (England)  RCT. N=74 women with singleton pregnancy; DBP ≥170/100 mmHg x 2; ≤36 wks gestation. | | Compared impact of administering labetalol 100 mg po QID (max 1200 mg daily) vs methyldopa 250 mg po QID (up to 4000 mg daily) with treatment goal ~140/90 mmHg (intervention); vs. hydralazine (unspecified dose)(controls). | | PMR: RR=4.49 (95% CI: 0.22-90.30)**[NS]**  [2/38 vs. 0/34 in intervention vs. control groups, respectively.] |
| 25. Thorley 1984 [26]. | UK.  RCT. N=60 women 18-36 wks gestation with undefined hypertension. | | Compared the impact of administration of atenolol 100 mg/day (intervention) vs. methyldopa 250 mg 3x/day (controls). | | PMR: [0/30 in both groups.] RR not estimable. |
| ***Beta-blocker versus hydralazine.*** | | | | | |
| 26. Hjertberg et al. 1993a [27]. | Sweden.  RCT. N=20 pregnant women, DBP ≥100 mmHg. | | Compared administration of labetalol po 300 mg daily (max 1200 mg daily) (intervention) vs hydralazine 25 mg po TID (max 400 mg daily) with treatment goal dBP 90-100 mmHg; (control). Additional comparison made between oral and IV therapy using the same drug to which women were randomised. | | PMR: RR=0.40 (95% CI: 0.02, 8.78)**[NS]**  [0/9 vs. 1/11 in intervention vs. controls, respectively.] |
| ***Beta-blocker versus calcium channel blocker.*** | | | | | |
| 27. Jannet et al. 1994 [28] | France.  RCT. N=100 women with singleton pregnancy, >20 wks gestation and mild-moderate hypertension (BP ≥140/90 mmHg x 2). | | Compared impact of administering metoprolol (slow release) 200 mg/day (intervention) vs. nicardipine 20 mg 3x/day (controls). | | PMR: RR=1.00 (95% CI: 0.06-15.55) **[NS]**  [1/50 in both groups] |

References

1. Magee LA, Duley L: **Oral beta-blockers for mild to moderate hypertension during pregnancy**. *Cochrane Database Syst Rev* 2003(3):CD002863.

2. Bott-Kanner G, Hirsch M, Friedman S, Boner G, Ovadia J, Merlob P, al. e: **Antihypertensive therapy in the management of hypertension in pregnancy - a clinical double-blind study of pindolol**. *Clinical and Experimental Hypertension* 1992, **B11**:207-220.

3. Butters L, Kennedy S, Rubin PC: **Atenolol in essential hypertension during pregnancy**. *BMJ* 1990, **301**(6752):587-589.

4. Cruickshank DJ, Campbell D, Robertson AA, MacGillivray I: **Intra-uterine growth retardation and maternal labetalol treatment in a random allocation controlled study**. *Journal of Obstetrics and Gynaecology;* 1992, **12**:223-227.

5. Hogstedt S, Lindeberg S, Axelsson O, Lindmark G, Rane A, Sandstrom B, Lindberg BS: **A prospective controlled trial of metoprolol-hydralazine treatment in hypertension during pregnancy**. *Acta Obstet Gynecol Scand* 1985, **64**(6):505-510.

6. Paran E, Holzberg G, Mazor M, Zmora E, Insler V: **Beta-adrenergic blocking agents in the treatment of pregnancy-induced hypertension**. *Int J Clin Pharmacol Ther* 1995, **33**(2):119-123.

7. Pickles CJ, Symonds EM, Broughton Pipkin F: **The fetal outcome in a randomized double-blind controlled trial of labetalol versus placebo in pregnancy-induced hypertension**. *Br J Obstet Gynaecol* 1989, **96**(1):38-43.

8. Plouin PF, Breart G, Llado J, Dalle M, Keller ME, Goujon H, Berchel C: **A randomized comparison of early with conservative use of antihypertensive drugs in the management of pregnancy-induced hypertension**. *Br J Obstet Gynaecol* 1990, **97**(2):134-141.

9. Rosenfeld J, Bott-Kanner G, Boner G, Nissenkorn A, Friedman S, Ovadia J, Merlob P, Reisner S, Paran E, Zmora E *et al*: **Treatment of hypertension during pregnancy with hydralazine monotherapy or with combined therapy with hydralazine and pindolol**. *Eur J Obstet Gynecol Reprod Biol* 1986, **22**(4):197-204.

10. Rubin PC, Butters L, Clark DM, Reynolds B, Sumner DJ, Steedman D, Low RA, Reid JL: **Placebo-controlled trial of atenolol in treatment of pregnancy-associated hypertension**. *Lancet* 1983, **1**(8322):431-434.

11. Sibai BM, Gonzalez AR, Mabie WC, Moretti M: **A comparison of labetalol plus hospitalization versus hospitalization alone in the management of preeclampsia remote from term**. *Obstet Gynecol* 1987, **70**(3 Pt 1):323-327.

12. Sibai BM, Mabie WC, Shamsa F, Villar MA, Anderson GD: **A comparison of no medication versus methyldopa or labetalol in chronic hypertension during pregnancy**. *Am J Obstet Gynecol* 1990, **162**(4):960-966; discussion 966-967.

13. Walker J: **Intrauterine growth retardation and maternal labetalol treatment in a random allocation controlled study**. Scotland: University of Glasgow; 1992.

14. Wichman K, Ryden G, Karlberg BE: **A placebo controlled trial of metoprolol in the treatment of hypertension in pregnancy**. *Scand J Clin Lab Invest Suppl* 1984, **169**:90-95.

15. Casavilla F, Vega HR: **Prospective and randomized study on mepindolol and alpha-methyldopa efficacy in arterial hypertension (AH) treatment during pregnancy.** In: *World Congress of Gynecology and Obstetrics: 1988 October 23-28.; Brazil.*; 1988 October 23-28.

16. Ellenbogen A, Jaschevatzky O, Davidson A, Anderman S, Grunstein S: **Management of pregnancy-induced hypertension with pindolol--comparative study with methyldopa**. *Int J Gynaecol Obstet* 1986, **24**(1):3-7.

17. Faneite PJ, Gonzalez X, Salazar G: **Evaluation of antihypertensives in pregnancy: prospective randomized study of mepindolol and alpha methyldopa [Evaluación de antihipertensivos en embarazadas: Mepindolol y Alfametildopa. Estudio Prospectivo y randomizado]**. *Revista de Obstetricia y Ginecologia de Venezuela* 1988, **48**:139-143.

18. Fidler J, Smith V, Fayers P, De Swiet M: **Randomised controlled comparative study of methyldopa and oxprenolol in treatment of hypertension in pregnancy**. *Br Med J (Clin Res Ed)* 1983, **286**(6382):1927-1930.

19. Gallery EDM, Ross MR, Gyory AZ: **Antihypertensive treatment in pregnancy: analysis of different responses to oxprenolol and methyldopa**. *BMJ* 1985, **291**:563-566.

20. Lamming GD, Broughton Pipkin F, Symonds EM: **Comparison of the alpha and beta blocking drug, labetalol, and methyl dopa in the treatment of moderate and severe pregnancy-induced hypertension**. *Clin Exp Hypertens* 1980, **2**(5):865-895.

21. Lardoux H, Blazquez G, Leperlier E, Gerard J: **Randomized and comparative study of methyldopa (MD), acebutolol (ACE) and labetalol for the treatment of moderate hypertension during pregnancy (HDP)**. *Archives des Maladies du Coeur* 1988, **91**:137-140.

22. Livingstone I, Craswell PW, Bevan EB, Smith MT, Eadie MJ: **Propranolol in pregnancy three year prospective study**. *Clin Exp Hypertens B* 1983, **2**(2):341-350.

23. Oumachigui A, Verghese M, Balachander J: **A comparative evaluation of metoprolol and methyldopa in the management of pregnancy induced hypertension**. *Indian Heart J* 1992, **44**(1):39-41.

24. Plouin PF, Breart G, Maillard F, Papiernik E, Relier JP: **Comparison of antihypertensive efficacy and perinatal safety of labetalol and methyldopa in the treatment of hypertension in pregnancy: a randomized controlled trial**. *Br J Obstet Gynaecol* 1988, **95**(9):868-876.

25. Redman C: **A controlled trial of the treatment of hypertension in pregnancy: labetalol compared with methyldopa**. In: *The Investigation of labetalol in the management of hypertension in pregnancy International Congress Series 591.* Edited by Symonds EM RA. Amsterdam: Excerpta Medica; 1978: 101-110.

26. Thorley K: **Randomised trial of atenolol and methyl dopa in pregnancy related hypertension**. *Clinical and Experimental Hypertension;* 1984, **133**:168.

27. Hjertberg R, Faxelius G, Lagercrantz H: **Neonatal adaptation in hypertensive pregnancy--a study of labetalol vs hydralazine treatment**. *J Perinat Med* 1993, **21**(1):69-75.

28. Jannet D, Carbonne B, Sebban E, Milliez J: **Nicardipine versus metoprolol in the treatment of hypertension during pregnancy: a randomized comparative trial**. *Obstet Gynecol* 1994, **84**(3):354-359.
