# Supplementary material for: Reducing stillbirths: prevention and management of medical disorders and infections during pregnancy
Source: BMC Pregnancy Childbirth. 2009 May 7;9(Suppl 1):S4. doi: 10.1186/1471-2393-9-S1-S4 (PMC2679410; doi:10.1186/1471-2393-9-S1-S4)
Supplement: Additional file 5 — Web Table 5. Component studies in Duley et al. 2006 meta-analysis: impact of anti-hypertensive drugs for chronic maternal hypertension. Component studies in Duley et al. 2006 meta-analysis reporting impact on stillbirths/perinatal mortality [file 1471-2393-9-S1-S4-S5.doc]

**Web Table 5. Component studies in Duley et al. 2006 [1] meta-analysis: impact of anti-hypertensive drugs for chronic maternal hypertension**

| **Source** | **Location and Type of Study** | **Intervention** | **Stillbirths / Perinatal Outcomes** |
| --- | --- | --- | --- |
| 1. Ashe et al. 1987 [2]. | South Africa.  RCT. Women (N=20) with DBP ≥110 mmHg not settled after 2 hrs bed rest and 200 mg phenobarbitone, ≥32 wks gestation, no previous hypotensive therapy, not in labour and no imminent eclampsia. | Assessed the impact of administration of labetalol 200 mg in 200 ml 5% dextrose at 20 mg/hr, increased every 20 min by 20 mg/hr until DBP 90-100 mmHg (max dose=160 mg/hr), continued for 1 hr (intervention) vs. hydralazine 25 mg in 200 ml saline at 3.7 mg/hr, increased every 20 min by 3.7 mg/hr until DBP 90-100 mmHg (max dose=15 mg/hr), continued for 1 hr (controls). | SBR+NMR: [0/10 in both groups.] RR not estimable. |
| 2. Bolte et al. 1999 [3] | Netherlands, multiple centres.  Multicenter RCT. 4 centres. Woemn (N=44) 26-32 wks gestation, DBP ≥110 mmHg. All women given plasma volume expansion at trial entry, 27/44 monitored with a pulmonary artery catheter (12 ketanserin, 15 hydralazine). | Compared administration of IV ketanserin 5 mg bolus then 4 mg/hr, increased every 20 min until target BP, max 10 mg/hr. Further 5 mg with every 2 mg/hr increment (intervention) vs IV hydralazine 1 mg/hr, hourly increments of 1 mg/hr until target BP, max 10 mg/hr (controls). MgSO4 for women with impending eclampsia (8 ketanserin, 11 hydralazine). Both groups, if BP not controlled, given other study drug. | PMR: RR=0.16 (95% CI: 0.01-2.87)**[NS]**  [0/17 vs. 3/19 in intervention vs. control groups, respectively.] |
| 3. de Souza et al. 1994 [4] | Brazil.  RCT. Women (N=50) with DBP > 110 mmHg after 60 min rest and > 28 wks gestation. | Compared nifedipine 10 mg administered sublingually with intravenous placebo (intervention) vs. hydralazine 20 mg administered intravenously with sublingual placebo (controls). | SBR+NMR: RR=1.00 (95% CI: 0.15-6.55) **[NS]**  [2/25 in each group]. |
| 4. Hall et al. 2000 [5] | South Africa.  RCT. Women (N=150) with severe early onset pre-eclampsia, and BP not controlled by methyldopa 2 g/day. | Compared impact of prazosin 1 mg 3x/day (max=21 mg/day)(intervention) vs.  nifedipine 10 mg 3x/day (max=60 mg/day)(controls).  If BP still not controlled, crossover. | SBR: RR=0.46 (95% CI: 0.18-1.13]**[NS]**  [6/75 vs. 13/74 in intervention vs. control groups, respectively]. |
| 5. Harper et al. 1991 [6] | UK (Northern Ireland)  RCT. Women (N=30) with singleton pregnancy before labour, no previous anti-hypertensive. BP 140/90 or above, clinical decision to treat - usually because of labile BP, proteinuria and symptoms. | Compared IV administration of labetalol 100 mg (intervention) to IV hydralazine 10 mg (controls). | SBR+NMR: RR=0.50 (95% CI: 0.05-4.94)**[NS]**  [1/15 vs. 2/15 in intervention vs. control groups, respectively.] |
| 6. Mabie et al.1987 [7] | USA.  RCT. Women (N=19) with hypertension during pregnancy [Also included women with postpartum hypertension (N=41), but these are excluded from this review]. | Compared impact of IV labetalol (regimen #1: 20 mg then 10-50 mg every 10 min until DBP ≤100 mmHg; regimen #2: 20 mg then repeat doses of 20 mg, 40 mg, 80 mg, 80 mg every 10 min, until DBP ≤ 100 mgHg (max=300 mg)(intervention) vs. IV hydralazine 5 mg every 10 min until DBP ≤100 mmHg (controls). | SBR: [0/13 vs. 0/6 in both intervention groups vs. controls, respectively.] RR not estimable. |
| 7. Maharaj et al. 1997a [8] | South Africa.  RCT. Primigravid women (N=40) with severe hypertension (DBP ≥110 mmHg) and no signs or symptoms of imminent eclampsia. All had 200 mg phenobarbitone 2 hr before trial entry. | Compared the impact of IV isradipine (0.15 mcg/kg/min, increased by 0.0025 mcg/kg every 15 min until DBP < 95 mmHg)(intervention) vs. IV hydralazine (6.25 mg iover 10 min, repeated once if DBP still > 95 mmHg)(controls). | SBR+NMR: RR=0.95 (95% CI: 0.06-14.22)**[NS]**  [1/21 vs. 1/20 in the intervention vs. control groups, respectively.] |
| 8. Martins-Costa et al. 1992 [9] | Brazil.  RCT. Primigravid women (N=37) >28 wks gestation with singleton live pregnancy and DBP 110 mmHg or more after 60 min rest, and proteinuria > 300 mg in 24 hr. | Assessed the impact of nifedipine 10 mg given orally (intervention) vs. hydralazine 5 mg given intravenously in hypertensive women. | SBR+NMR: RR=4.29 (95% CI: 0.22-83.57)**[NS]**  [2/20 vs. 0/17 in intervention vs. control groups, respectively]. |
| 9. Michael 1986 [10] | Australia.  RCT. Women (N=90) with DBP >105 mmHg after sedation with either phenobarbitone 200 mg or diazepam 10 mg 6 hourly. Delivery planned for soon after treatment. | Compared the impact of administering labetalol 200 mg in 200 ml 5% dextrose iv at 0.5 mg/kg/hr to a maximum of 3 mg/kg/hr, to keep DBP at 85-90 mmHg, continued until 24 hrs after delivery (intervention) vs. diazoxide 75 mg intravenously, repeated every 30 min until BP controlled and continued until 24 hrs after delivery (controls). | PMR: RR=0.14 (95% CI: 0.01-2.69)**[NS]**  [0/45 vs. 3/45 in intervention vs. control groups, respectively.] |
| 10. Moore et al.1982 [11]. | UK (England)  RCT. Women (N=74) with BP ≥170/110 mmHg, < 36 wks gestation. Excluded multiple pregnancy, diabetes, Rh isoimmunisation. | Compared administration of labetalol 100 mg 4x/day (intervention) with methyldopa 250 mg 4x/day (controls). Hydralazine given to both groups if BP not controlled. | SBR: [0/38 vs. 0/34 in intervention vs. control groups.] RR not estimable. |
| 11. Seabe et al. 1989 [12]. | South Africa.  RCT. Primigravid women (N=33); no hypertension, renal disease, or other medical problems; no anti-hypertensive therapy; DBP ≥110 mmHg for 2 hr; ≥ 28 wks gestation. Not needing immediate delivery and no fetal distress. | Assessed the impact of nifedipine 10 mg orally, repeated after 30 min if no response (intervention) vs. hydralazine 6.25 mg in 10 ml water IV over 5-10 min, repeated after 30 min if no response (controls). | SBR+NMR: RR=0.94 (95% CI: 0.06-13.82) **[NS]**  [1/17 vs. 1/16 in intervention vs. control groups, respectively.] |
| 12. Steyn and Odendaal 1997b [13] | South Africa.  RCT. Women (N=88) ≥28 wks gestation, DBP > 110 mmHg or DBP > 100 mmHg for 30 minutes. South Africa. | Compared impact of IV ketanserin (crystalloid 500 ml, then bolus 10 mg ketanserin in 4 ml IV; bolus repeated every 20 min until DBP 90 mmHg, max=4 doses) (intervention) vs. IV hydralazine (crystalloid 500 ml over 15 min, then bolus 5 mg hydralazine in 4 ml IV; bolus repeated every 20 min until DBP 90 mmHg, max= 4 doses)(controls). | PMR: RR=0.45 (95% CI: 0.04-4.79).  [1/42 vs. 2/38 in intervention vs. control groups, respectively.] |
| 13. Wacker et al. 1998 [14]. | Germany.  RCT. Women (N=26) with BP 160/110 mmHg after 3 hr bed rest, 1+ of proteinuria, oedema or hyperreflexia, 26-38 wks gestation. No IV anti-hypertensive before entry. | Compared administration of urapidil 6.25 mg intravenously, repeated after 5 min if BP not decreased, then 2-4 mg/hr until delivery (intervention) vs. hydralazine intravenously, mean 0.13 mg/kg/4 hrs (controls). | SBR: [0/13 in both groups] RR not estimable. |

References

1. Duley L, Henderson-Smart DJ, Meher S: **Drugs for treatment of very high blood pressure during pregnancy**. *Cochrane Database Syst Rev* 2006, **3**:CD001449.

2. Ashe RG, Moodley J, Richards AM, Philpott RH: **Comparison of labetalol and dihydralazine in hypertensive emergencies of pregnancy**. *S Afr Med J* 1987, **71**(6):354-356.

3. Bolte AC, van Eyck J, Kanhai HH, Bruinse HW, van Geijn HP, Dekker GA: **Ketanserin versus dihydralazine in the management of severe early-onset preeclampsia: maternal outcome**. *Am J Obstet Gynecol* 1999, **180**(2 Pt 1):371-377.

4. de Souza MR, Nagib A, Bertini AM: **Use of hydralazine and nifedipine in hypertensive emergency in pregnancy [Empleo de la hidralazina y de la nifedipina en las emergencias hipertensivas en la gestacion]**. *Progresos de Obstetricia y Ginecologia* 1994, **37**:90-96.

5. Hall DR, Odendaal HJ, Steyn DW, Smith M: **Nifedipine or prazosin as a second agent to control early severe hypertension in pregnancy: a randomised controlled trial**. *BJOG* 2000, **107**(6):759-765.

6. Harper A, Murnaghan GA: **Maternal and fetal haemodynamics in hypertensive pregnancies during maternal treatment with intravenous hydralazine or labetalol**. *Br J Obstet Gynaecol* 1991, **98**(5):453-459.

7. Mabie WC, Gonzalez AR, Sibai BM, Amon E: **A comparative trial of labetalol and hydralazine in the acute management of severe hypertension complicating pregnancy**. *Obstet Gynecol* 1987, **70**(3 Pt 1):328-333.

8. Maharaj B, Khedun SM, Moodley J, van der Byl K, Rapiti N: **A comparative study of intravenous isradipine and dihydralazine in the treatment of severe hypertension of pregnancy in black patients**. *Hypertension in Pregnancy;* 1997, **16**:1-9.

9. Martins-Costa S, Ramos JG, Barros E, Bruno RM, Costa CA: **Randomized, controlled trial of hydralazine versus nifedpine in preeclamptic women with acute hypertension**. *Clinical and Experimental Hypertension;* 1992, **B11**:25-44.

10. Michael CA: **Intravenous labetalol and intravenous diazoxide in severe hypertension complicating pregnancy**. *Aust N Z J Obstet Gynaecol* 1986, **26**(1):26-29.

11. Moore MP, Redman CWG: **The treatment of hypertension in pregnancy**. *Current Medical Research and Opinion;* 1982, **8**:S39-S46.

12. Seabe SJ, Moodley J, Becker P: **Nifedipine in acute hypertensive emergencies in pregnancy**. *S Afr Med J* 1989, **76**(6):248-250.

13. Steyn DW, Odendaal HJ: **Dihydralazine or ketanserin for severe hypertension in pregnancy? Preliminary results**. *Eur J Obstet Gynecol Reprod Biol* 1997, **75**(2):155-159.

14. Wacker J, Werner P, Walter-Sack I, Bastert G: **Treatment of hypertension in patients with pre-eclampsia: a prospective parallel-group study comparing dihydralazine with urapidil**. *Nephrol Dial Transplant* 1998, **13**(2):318-325.
