# Supplementary material for: Reducing stillbirths: prevention and management of medical disorders and infections during pregnancy
Source: BMC Pregnancy Childbirth. 2009 May 7;9(Suppl 1):S4. doi: 10.1186/1471-2393-9-S1-S4 (PMC2679410; doi:10.1186/1471-2393-9-S1-S4)
Supplement: Additional file 6 — Web Table 6. Component studies in King et al. 2003 meta-analysis: impact of anti-hypertensive drugs for chronic maternal hypertension. Component studies in King et al. 2003 meta-analysis reporting impact on stillbirths/perinatal mortality [file 1471-2393-9-S1-S4-S6.doc]

**Web Table 6. Component studies in King et al. 2003 [1] meta-analysis: impact of anti-hypertensive drugs for chronic maternal hypertension**

| **Source** | **Location and Type of Study** | **Intervention** | **Stillbirths / Perinatal Outcomes** |
| --- | --- | --- | --- |
| 1. Bracero 1991 [2]. | USA.  RCT. Women (N=49) in pre-term labour, 20-36 wks gestation. | Compared calcium channel blocker (nifedipine 30mgs po initially then 20mgs q6h for 24hrs then 20mgs q8h for 24hrs followed by maintenance 20mgs q8-12h prn)(intervention) to other tocolytic group (ritodrine 100 µg/min increasing by 50µg/min q10min prn to a maximum of 350µg/min, maintenance 10-20mg q4-6 h)(controls). | PMR: RR=2.50 (95% CI: 0.11-58.06)**[NS]**  [1/23 vs. 0/19 in intervention vs. control groups, respectively.] |
| 2. Ferguson 1990 [3]. | USA.  RCT. N=66 women in pre-term labour, 20-36 wks gestation. | Compared calcium channel blocker (nifedipine 10mg capsule s/l repeated in 20 mins oral maintenance 20 mg q4-6h)(intervention) to other tocolytic group (ritodrine, 50 µg/min increasing by 50 µg 15-30 mins up to a maximum of 350µg/min, maintenance 10-20 q4-6 h)(controls). | PMR: RR=7.00 (95% CI: 0.38-130.41)**[NS]**  [3/33 vs. 0/33 in intervention vs. control groups, respectively.] |
| 3. Garcia-Velasco 1998 [4]. | Spain.  RCT. N=52 women in pre-term labour, 26-34 wks gestation. | Compared calcium channel blocker (nifedipine 10mgs s/l and 20mgs po then 10-20 q4-6 h prn)(intervention) vs. other tocolytic group (IV ritodrine, 50 µg/min increasing by 50 ug q20mins to max of 350µg/min maintained for 12 hrs. The oral maintenance 5mgs q3h)(controls). Indomethacin was given for continued uterine activity after 12 h or if treatment was not well tolerated. | PMR: [0/26 in both groups.] RR not estimable. |
| 4. Glock 1993 [5]. | USA.  RCT. N=100 women in pre-term labour < 34 wks gestation. | Compared calcium channel blocker (Nifedipine 10mg s/l repeated prn every 20 mins to max of 40mg in first hr. Once contractions ceased 20mg q4h for 48 h, then maintenance 10mg q8h until 34 wks)(intervention) vs. other tocolytic group (MgSO4 load 6gIV over 30 mins then 2g per hr IV up to 4g per hr as required for 24 h, then weaned at 0.5g every 4-6 hrs, then maintenance therapy of oral terbutaline 5mg q6h until 34 wks)(controls). | PMR: RR=7.00 (95% CI: 0.35-140.60)**[NS]**  [2/29 vs. 0/41 in intervention vs. control groups, respectively.] |
| 5. Janky 1990 [6]. | France (Bordeaux)  RCT. N=62 women in pre-term labour, 28-36 wks gestation, at Service de Gynécologie, Hôpital Pellegrin. | Compared calcium channel blocker (nifedipine 10mgs s/l then 20mgs q8h. Ceased after 7 d)(intervention) vs. other tocolytic group (IV Ritodrine, 200 to 300 µg/min until contractions ceased then 100µg/min for 24 hr then oral maintenance 20mgs 4-6 h for 6 d)(controls). | PMR: [0/30 vs. 0/32 in intervention vs. control groups, respectively.] RR not estimable. |
| 6. Kupferminc 1993 [7]. | Israel.  RCT. N=71 women in pre-term labour, 26-34 wks gestation. | Compared calcium channel blocker (nifedipine 30 mg po then 20mgs after 90 min if required then maintenance 20mgs q8h until 34-35 wks. Switched to Ritodrine if contractions continued after 150 mins.)(intervention) vs. other tocolytic group (IV Ritodrine 50µg/min increasing by 15 µg q15 to a maximum of 300ug/min for 12 hours, oral maintenance 10mgs q3h until 34-35 wks)(controls). | PMR: RR=0.32 (95% CI: 0.01-7.58)**[NS]**  [0/42 vs. 1/40 in intervention vs. control groups, respectively.] |
| 7. Larmon 1999 [8]. | USA.  RCT. N=122 women in pre-term labour 22-34 wks gestation. | Compared calcium channel blocker (nicardipine 40 mg po then 20mgs q2h prn up to 3 doses then oral maintenance 45mgs q12h until 37 wks)(intervention) vs. other tocolytic group (IV MgSO4 loading dose of 6g then 2g/hr increasing up to a maximum of 4g/hr prn. Oral maintenance Mg lactate 4 tabs q12h until 37 wks)(controls) | PMR: [0/57 vs. 0/65 in intervention vs. control groups, respectively.] RR not estimable. |
| 8. Papatsonis 1997 [9]. | The Netherlands.  RCT. N=185 women in pre-term labour, 20-34 wks gestation, at 3 hospitals. | Compared calcium channel blocker (nifedipine 10mgs s/l, repeated if necessary po 10mg q15mins up to 40mg in the first hr. Maintenance 60-160mgs/day up to 34 wks)(intervention) vs. other tocolytic group (ritodrine commencing at 383µg/min increasing prn until cessation of contractions then decreasing as tocolysis is established (minimum 100 µg/min) and continued for 3 d. Maintenance 40mg po q8h up to 34 wks in 2 of 3 participating hospitals.)(controls). | PMR: RR=1.11 (95% CI: 0.39-3.16)**[NS]**  [7/95 vs. 6/90 in intervention vs. control groups, respectively]. |
| 9. Read 1986 [10]. | UK.  RCT. N=40 women in pre-term labour, 20-35 wks gestation. | Compared calcium channel blocker (nifedipine 30mgs po then 20mg q8h for 3 days; ritodrine started after 2 hrs if contractions were undiminished)(intervention) vs. other tocolytic group (ritodrine 50 µg/min increasing by 50µg q 10 mins to max=300µg. Maintained for 12 h then oral maintenance for 48 h)(controls). | PMR: [0/20 in both groups]  RR not estimable. |
| 10. Weerakul 2002 [11]. | Thailand.  RCT. N=90 women in pre-term labour with a singleton pregnancy, 28-34 wks gestation. | Compared impact of calcium channel blocker (nifedipine 10mgs s/l capsule crushed repeated after 15 mins, then 20mg after 30 mins to a maximum in the first hr of 40mg. Maintenance of 60-120 mg daily for 3 days.)(intervention) vs. other tocolytic group (terbutaline IV loading of 0.25mg, then infusion commencing at 5µg/min increasing by 5µg/min every 15 mins depending on contractions, max=15µg/min. Following uterine quiesence infusion maintained for 2 hrs then subcutaneous injection 0.25mg q4h for 24hrs.)(controls) | PMR: [0/45 vs. 0/44 in intervention vs. control groups, respectively.] RR not estimable. |

References

1. King JF, Flenady VJ, Papatsonis DN, Dekker GA, Carbonne B: **Calcium channel blockers for inhibiting preterm labour**. *Cochrane Database Syst Rev* 2003(1):CD002255.

2. Bracero LA, Leikin E, Kirshenbaum N, Tejani N: **Comparison of nifedipine and ritodrine for the treatment of preterm labor**. *Am J Perinatol* 1991, **8**(6):365-369.

3. Ferguson JE, 2nd, Dyson DC, Schutz T, Stevenson DK: **A comparison of tocolysis with nifedipine or ritodrine: analysis of efficacy and maternal, fetal, and neonatal outcome**. *Am J Obstet Gynecol* 1990, **163**(1 Pt 1):105-111.

4. Garcia-Velasco JA, Gonzalez Gonzalez A: **A prospective, randomized trial of nifedipine vs. ritodrine in threatened preterm labor**. *Int J Gynaecol Obstet* 1998, **61**(3):239-244.

5. Glock JL, Morales WJ: **Efficacy and safety of nifedipine versus magnesium sulfate in the management of preterm labor: a randomized study**. *Am J Obstet Gynecol* 1993, **169**(4):960-964.

6. Janky E, Leng JJ, Cormier PH, Salamon R, Meynard J: **[A randomized study of the treatment of threatened premature labor. Nifedipine versus ritodrine]**. *J Gynecol Obstet Biol Reprod (Paris)* 1990, **19**(4):478-482.

7. Kupferminc M, Lessing JB, Yaron Y, Peyser MR: **Nifedipine versus ritodrine for suppression of preterm labour**. *Br J Obstet Gynaecol* 1993, **100**(12):1090-1094.

8. Larmon JE, Ross BS, May WL, Dickerson GA, Fischer RG, Morrison JC: **Oral nicardipine versus intravenous magnesium sulfate for the treatment of preterm labor**. *Am J Obstet Gynecol* 1999, **181**(6):1432-1437.

9. Papatsonis DN, Van Geijn HP, Ader HJ, Lange FM, Bleker OP, Dekker GA: **Nifedipine and ritodrine in the management of preterm labor: a randomized multicenter trial**. *Obstet Gynecol* 1997, **90**(2):230-234.

10. Read MD, Wellby DE: **The use of a calcium antagonist (nifedipine) to suppress preterm labour**. *Br J Obstet Gynaecol* 1986, **93**(9):933-937.

11. Weerakul W, Chittacharoen A, Suthutvoravut S: **Nifedipine versus terbutaline in management of preterm labor**. *Int J Gynaecol Obstet* 2002, **76**(3):311-313.
