# Supplementary material for: Reducing stillbirths: prevention and management of medical disorders and infections during pregnancy
Source: BMC Pregnancy Childbirth. 2009 May 7;9(Suppl 1):S4. doi: 10.1186/1471-2393-9-S1-S4 (PMC2679410; doi:10.1186/1471-2393-9-S1-S4)
Supplement: Additional file 7 — Web Table 7. Component studies in Say et al. 1996 meta-analysis: impact of anti-hypertensive drugs for chronic maternal hypertension. Component studies in Say et al. 1996 meta-analysis reporting impact on stillbirths/perinatal mortality [file 1471-2393-9-S1-S4-S7.doc]

**Web Table 7. Component studies in Say et al. 1996 [1] meta-analysis: impact of anti-hypertensive drugs for chronic maternal hypertension**

| **Source** | **Location and Type of Study** | **Intervention** | **Stillbirths / Perinatal Outcomes** |
| --- | --- | --- | --- |
| 1. Janssens 1985 [2]. | The Netherlands.  RCT. N=100 smoking pregnant women 16-20 wks gestation (N=50 intervention, N=50 control). | Compared the impact of 10 mg flunarizine (intervention) or placebo (control) administered orally daily from 16-20th wk until delivery. | PMR: OR=0.14 (95% CI: 0.00-6.82)**[NS]**  [0/50 vs. 1/50 in intervention vs. control groups, respectively.] |

References

1. Say L, Gülmezoglu AM, Hofmeyr GJ: **Calcium channel blockers for potential impaired fetal growth**. *Cochrane Database of Systematic Reviews;* 1996(1):CD000049.

2. Janssens D: **Prevention of low birth weight by flunarizine given to smoking mothers**. *Archives of Gynecology;* 1985, **237**:397.
