# Supplementary material for: Reducing stillbirths: prevention and management of medical disorders and infections during pregnancy
Source: BMC Pregnancy Childbirth. 2009 May 7;9(Suppl 1):S4. doi: 10.1186/1471-2393-9-S1-S4 (PMC2679410; doi:10.1186/1471-2393-9-S1-S4)
Supplement: Additional file 8 — Web Table 8. Component studies in Meher and Duley. 2007 meta-analysis: impact of anti-hypertensive drugs for chronic maternal hypertension. Component studies in Meher and Duley. 2007 meta-analysis reporting impact on stillbirths/perinatal mortality [file 1471-2393-9-S1-S4-S8.doc]

**Web Table 8. Component studies in Meher and Duley 2007 [1] meta-analysis: impact of anti-hypertensive drugs for chronic maternal hypertension**

| Source | Location and Type of Trial | Intervention | Stillbirths/Perinatal Outcomes |
| --- | --- | --- | --- |
| **Nitric oxide vs. placebo/no intervention** | | | |
| 1. Facchinetti F, et al. 2002 [2] | Italy.  RCT. N=74 women, 24-36 wks gestation. | Compared an intervention group receiving L-arginine 20 g/500 ml IV daily for 5 days, then 4 g/d oral for 2 weeks to control group receiving placebo. | PMR+NMR: RR=0.24 (95% CI: 0.01-5.55)  [0/27 vs. 1/19 in intervention vs. placebo groups, respectively.] |
| 2. Picciolo C, et al. 2000 [3] | Italy.  RCT. N=68 women <16 wks gestation. | Compared an intervention group receiving glyceryl trinitrate skin patch 5 mg for 14-16 hrs/day, from 16-38 wks gestation, to controls who were merely observed. | PMR or NMR: RR = 0.26 (95% CI: 0.01 – 6.28) **[NS]**.  [0/38 vs. 1/30 in intervention and control groups, respectively]. |
| **Nitric oxide vs. nifedipine** | | | |
| 3. Neri I, et al.1999 [4] | Italy.  RCT. N=36 women. | Compared a group receiving glyceryl trinitrate skin patch 10 mg continuously (intervention #1) to a group receiving glyceryl trinitrate skin patch 10 mg for 16 hrs/day (intervention #2) to a group receiving oral nifedipine 40 mg/d (controls). | PMR: [0/24 vs. 0/12 in trinitrate intervention groups vs. controls, respectively.] RR not estimable. |
| **Nitric oxide vs. anti-platelet agents** | | | |
| 4. Zozulia O, et al. 1997 [5] | Italy.  RCT. N=76 women. | Compared an intervention group receiving glyceryl trinitrate skin patch 5 mg, increasing to 20 mg if tolerated, for 12 hrs/day, from 16-20 weeks' gestation until delivery to controls administered anti-platelet agents (acetyl salicylic acid 125 mg/day and curantil (dipyridamole) 150-225 mg/day). | NMR: RR=0.71 (95% CI: 0.17-2.97)**[NS]**  [3/39 vs. 4/37 intervention vs. control groups, respectively.] |

References

1. Meher S, Duley L: **Nitric oxide for preventing pre-eclampsia and its complications**. *Cochrane Database Syst Rev* 2007(2):CD006490.

2. Facchinetti F, Piccinini F, Pizzi C, Bukowski R, Volpe A, Saade G: **Effect of arginine supplementation in patients with gestational hypertension**. *American Journal of Obstetrics and Gynecology* 2002, **187**(6 Pt 2):S213.

3. Picciolo C, Roncaglia N, Neri I, Pasta F, Arreghini A, Facchinetti F: **Nitric oxide in the prevention of pre-eclampsia**. *Prenatal and Neonatal Medicine;* 2000, **5**(4):212-215.

4. Neri I, Valensise H, Facchinetti F, Menghini S, Romanini C, Volpe A: **24-hour ambulatory blood pressure monitoring: a comparison between transdermal glyceryl-trinitrate and oral nifedipine**. *Hypertens Pregnancy* 1999, **18**(1):107-113.

5. Zozulia OV, Rogov VA, Piatakova NV, Tareeva IE: **[Nitric oxide: its role in the development of pregnancy complications and in their prevention in women with hypertension and chronic glomerulonephritis]**. *Ter Arkh* 1997, **69**(6):17-20.
