# Supplementary material for: Reducing stillbirths: prevention and management of medical disorders and infections during pregnancy
Source: BMC Pregnancy Childbirth. 2009 May 7;9(Suppl 1):S4. doi: 10.1186/1471-2393-9-S1-S4 (PMC2679410; doi:10.1186/1471-2393-9-S1-S4)
Supplement: Additional file 9 — Web Table 9. Component studies in Duley et al. 2007 meta-analysis: impact of anti-platelet agents. Component studies in Duley et al. 2007 meta-analysis reporting impact on stillbirths/perinatal mortality [file 1471-2393-9-S1-S4-S9.doc]

**Web Table 9. Component studies in Duley et al. 2007 [1] meta-analysis: impact of anti-platelet agents**

| **Source** | **Location and Type of Study** | **Intervention** | **Stillbirths / Perinatal Outcomes** |
| --- | --- | --- | --- |
| 1. Anonymous 1996 [2]. | Brazil.  RCT, N=985 women. | Compared the impact of aspirin (60 mg daily; intervention) vs. placebo (controls). | Fetal death rate (miscarriage+SB): RR=1.27 (95% CI: 0.74-2.17)**[NS]**  [28/482 vs. 23/503 in intervention vs. control groups, respectively.] |
| 2. August et al. 1994 [3]. | USA.  RCT. N=49 women. | Compared the impact of aspirin (100 mg sustained release daily until 37 wks; intervention) vs. placebo (controls). | Fetal death rate (miscarriage+SB): RR=1.04 (95% CI: 0.07-15.73)  [1/24 vs. 1/25 in intervention vs. control groups, respectively.] |
| 3. Authors not known, 1993 [4]. | Italy.  RCT. N=1,172 women. | Compared the impact of aspirin (50 mg daily; intervention) vs. no treatment (controls). | Fetal death rate (miscarriage+SB): RR=0.79 (95% CI: 0.37-1.66)**[NS]**  [13/634 vs. 14/538 in intervention vs. control groups, respectively.] |
| 4. Azar and Turpin 1990 [5]. | France.  RCT. Women (N=91) at high risk of PIH because of previous early onset PE, severe IUGR or fetal death due to placental insufficiency. | Compared the impact of aspirin (100 mg) and dipyridamole (300 mg) daily until delivery; intervention) vs. placebo (controls). | Fetal death rate (miscarriage+SB): RR=0.98 (0.14-6.65)**[NS]**  [2/46 vs. 2/45 in intervention vs. control groups, respectively.] |
| 5. Beaufils et al. 1985 [6]. | France.  RCT. N=93 women. | Compared the impact of aspirin (150 mg) and dipyridamole (300 mg) daily from 3 mos until delivery; intervention) vs. placebo (controls). | Fetal death rate (miscarriage+SB): RR=0.10 (95% CI: 0.0-1.88)**[NS]**  [0/48 vs. 4/45 in intervention vs. control groups, respectively.] |
| 6. Benigni et al. 1989 [7]. | Italy.  RCT. Women (N=33) at risk of hypertension because of essential hypertension or a significant previous obstetric history (placental insufficiency causing fetal death, severe IUGR or pre-eclampsia < 32 wks). | Compared the impact of aspirin (60 mg daily from 12 wks until delivery; intervention) vs. placebo (controls). | PMR+IMR: RR=0.31 (95% CI: 0.01-7.21)**[NS]**  [0/17 vs. 1/16 in intervention vs. control groups, respectively.] |
| 7. Byaruhanga et al. 1998 [8] | Zimbabwe.  RCT. Women (N=250) 20-28 wks gestation with a history of pre-eclampsia in a previous pregnancy, especially if at < 32 wks, or chronic hypertension. | Compared the impact of aspirin (75 mg daily; intervention) vs. placebo (controls). | PMR: RR=0.41 (95% CI: 0.15-1.12)  [5/114 vs. 13/122 in intervention vs. control groups, respectively.] |
| 8. Caritis et al. 1998 [9]. | USA.  RCT. N=3,216 women. | Compared the impact of aspirin (60 mg daily; intervention) vs. placebo (controls). | Fetal death rate (miscarriage+SB): RR=0.71 (95% CI: 0.48-1.06)[**NS]**  [40/1612 vs. 56/1604 in intervention vs. control groups, respectively.] |
| 9. Caspi et al. 1994 [10]. | Israel.  RCT. Women (N=48) with twin pregnancies ~18 wks gestation. | Compared the impact of aspirin (100 mg daily; intervention) vs. placebo (controls). | Fetal death rate (miscarriage+SB): RR=0.20 (95% CI: 0.01-4.06)**[NS]**  [0/48 vs. 2/48 in intervention vs. control groups, respectively.] |
| 10. Chiaffarino et al. 2004 [11]. | Italy.  RCT. Women (N=40) < 14 wks' gestation with chronic HT +/- nephropathy or history of severe pre-eclampsia or eclampsia or IUGR or stillbirth. | Compared the impact of aspirin (100 mg daily until delivery; intervention) vs. no treatment (controls). | Fetal death rate (miscarriage+SB): RR=0.59 (95% CI: 0.06-5.96)**[NS]**  [1/16 2/19 in intervention vs. control groups, respectively.] |
| 11. CLASP (Collaborative Low-dose Aspirin Study in Pregnancy) 1994 [12]. | 16 countries.  RCT. Women (N=9364) women at risk of pre-eclampsia or IUGR, or women with established pre-eclampsia or IUGR, 12-32 wks' gestation. | Compared the impact of aspirin (60 mg daily until delivery; intervention) vs. placebo (controls). | PMR+IMR: RR=0.80 (95% CI: 0.59-1.07)**[NS]**  [77/4123 vs. 97/4134 in intervention vs. control groups, respectively.] |
| 12. Cowchock and Reece 1997 [13]. | USA.  RCT. N=19 women with anti-phospholipid antibodies and ≤ 2 previous miscarriages with no other anti-phospholipid antibody related complications. | Compared the impact of aspirin (81 mg daily; intervention) vs. usual care (controls). | PMR+IMR: RR=2.25 (95% CI: 0.10-49.04)**[NS]**  [1/11 vs. 0/8 in intervention vs. control groups, respectively.] |
| 13. Davies et al. 1995 [14] | UK.  RCT. N=160 women. | Compared the impact of aspirin (75 mg daily from 18 wks until delivery; intervention) vs. placebo (controls). | PMR: RR=0.47 (95% CI: 0.19-1.18)**[NS]**  [6/79 vs. 13/81 in intervention vs. control groups, respectively.] |
| 14. Dekker 1989 [15]. | Netherlands.  RCT. Primigravid women (N=10) with chronic hypertension and a positive angiotensin II sensitivity test at 26 wks' gestation. No proteinuria, BP < 90 mmHg diastolic, serum creatinine < 70 umol/L and an adequately grown fetus. | Compared the impact of aspirin (60 mg daily; intervention) vs. placebo (controls). | Fetal death rate (miscarriage+SB): 0/5 in both groups. RR not estimable. |
| 15. Essinger 1992a [16] | Brazil.  RCT. Women (N=56) who were young primigravidas, or had chronic HT, diabetes, previous PIH, twin pregnancy, or a family history of HT. | Compared the impact of aspirin (60 mg/day) in a solution of 50% D-lisine [intervention] vs. no intervention [controls]. | PMR+IMR: RR=0.20 (95% CI: 0.01-3.97)**[NS]**  [0/26 vs. 2/26 in intervention vs. control groups, respectively.] |
| 16. Gallery et al. 1997 [17] | Australia.  RCT. Women (N=120) at high risk of pre-eclampsia because of one of the following: pre-existing hypertension (BP greater ≥ 140/90 prior to pregnancy on at least 2 occasions, or on anti-hypertensive therapy), renal disease, previous early severe PE. | Compared the impact of aspirin (100 mg modified release daily from 17-19 weeks until delivery; intervention) vs. placebo (controls). | Fetal death rate (miscarriage+SB): RR=7.78 (95% CI: 0.43-141.0)  [54/58 vs. 0/50 in intervention vs. control groups, respectively.] |
| 17. Golding 1998 [18]. | Jamaica.  RCT. Primiparous women (N=6275) 12-32 wks and no contraindication to aspirin. | Compared the impact of aspirin (60 mg daily until delivery; intervention) vs. placebo (controls). | PMR: RR=0.84 (95% CI: 0.63-1.11)**[NS]**  [86/3023 vs. 103/3026 in intervention vs. control groups, respectively.] |
| 18. Hauth et al. 1993 [19] | USA.  RCT. Primiparous women (N=604) at 24 wks’ gestation, in single antenatal clinic. | Compared the impact of aspirin (60 mg daily from 22 wks; intervention) vs. placebo (controls). | Fetal death rate (miscarriage+SB): RR=1.00 (95% CI: 0.06-15.91)**[NS]**  [1/302 vs. 1/302 in intervention vs. control groups, respectively.] |
| 19. Herabutya et al. 1996 [20] | Thailand.  RCT. N=1348 women. | Compared the impact of aspirin (60 mg daily until birth; intervention) vs. placebo (controls). | SB: OR=3.21 (95% CI: 0.13-78.70) **[NS]**  [1/651 vs 0/697 in intervention vs. control groups, respectively.] |
| 20. Hermida et al. 1997 [21]. | Spain.  RCT. Women (N=107) age 18-40, < 16 wks' gestation and at moderate risk of pre-eclampsia (history of PIH, PE, chronic HT, cardiovascular or endocrine problem, bleeding or endocrine disease). | Compared the impact of aspirin (100 mg daily; intervention) vs. placebo (controls), administered to three groups of patients at different times of the day. | PMR+IMR: 0/50 in both the groups. RR not estimable. |
| 21. Kincaid-Smith P 1991, 1996 [22]. | Australia.  RCT. Primigravid women (N=52) with abnormal uterine artery waveforms on doppler examination at 22-24 wks. | Compared the impact of aspirin (60 mg daily; intervention) vs. placebo (controls). | Fetal death rate (miscarriage+SB): 0/27 vs. 0/25 in intervention vs. control groups, respectively. |
| 22. McParland et al. 1990 [23]. | UK.  RCT. N=100 women. | Compared the impact of aspirin (75 mg daily; intervention) vs. placebo (controls). | Fetal death rate (miscarriage+SB): RR=0.54 (95% CI: 0.05-5.78)**[NS]**  [1/48 vs. 2/52 in intervention vs. control groups, respectively.] |
| 23. Quenby et al. 1992b [24]. | UK.  RCT. Women (N=26) with history of recurrent miscarriage or connective tissue disorder, and positive anti-cardiolipin antibodies. | Compared the impact of aspirin (75 mg daily; intervention) vs. no treatment (controls). | PMR+IMR: RR=0.52 (95% CI: 0.02-11.54)**[NS]**  [0/10 vs. 1/16 in intervention vs. control groups, respectively.] |
| 24. Rai et al. 1993 [25] | India.  RCT.Women (N=100) with PIH, 24-36 wks' gestation. | Compared the impact of aspirin (60 mg daily until delivery; intervention) vs. “standard treatments only” (controls). | PMR+IMR: RR=0.09 (95% CI: 0.01-1.60)**[NS]**.  [0/50 vs. 5/50 in intervention vs. control groups, respectively.] |
| 25. Railton A, Davey A 1988, 1988 [26, 27]*.* | South Africa.  RCT. Women (N=44) with elevated mid-trimester BP, 12-28 wks' gestation, DBP 80-105 mmHg, and otherwise normal. | Compared the impact of aspirin (81 mg; intervention group #1), or aspirin (81 mg) plus dipyridamole (200 mg)(intervention group #2) daily vs. no treatment (controls). | Fetal death rate (miscarriage+SB): RR=0.93 (95% CI: 0.09-9.45)**[NS]**  [2/30 vs. 1/14 in intervention groups vs. control group, respectively.] |
| 26. Rogov et al. 1993 [28]. | Russia.  RT. Women (N=76) with chronic glomerulonephritis or essential hypertension. | Compared the impact of aspirin (125 mg) plus dipyridamole (150-225 mg) daily from 12-19 wks; intervention) vs. no treatment (controls). Control: no treatment. | Fetal death rate (miscarriage+SB): RR=0.27 (95% CI: 0.03-2.25)**[NS]**  [1/31 vs. 4/33 in intervention vs. control groups, respectively.] |
| 27. Rotchell et al. 1998 [29] | Barbados.  RCT. N=3675 women analysed. | Compared the impact of aspirin (75 mg controlled release daily until delivery; intervention) vs. placebo (controls). | Fetal death rate (miscarriage+SB): RR=1.08 (95% CI: 0.64-1.81)**[NS]**  [29/1834 vs. 27/1841 in intervention vs. control groups, respectively.] |
| 28. Roy and Pan 1994 [30]. | India.  RCT. Nulliparous women (N=94) with PIH in the 3rd trimester (SBP ≥140 mmHg, or DBP ≥90 mmHg, or both, on 2 occasions ≥6 hr but ≤ 24 hr apart). | Compared the impact of aspirin (75 mg daily until 10 days prior to EDD; intervention) vs. no anti-platelet agent (controls). | PMR+IMR: 0/46 vs. 0/48 in intervention vs. control groups, respectively. RR not estimable. |
| 29. Schiff et al. 1989 [31]. | Israel.  RCT. Women (N=65) with either twin pregnancy or a history of PE, and a positive roll-over test at 28-29 wks' gestation. | Compared the impact of aspirin (100 mg daily; intervention) vs. placebo (controls). | Fetal death rate (miscarriage+SB): 0/34 vs. 0/32 in intervention vs. control groups, respectively. RR not estimable. |
| 30. Schrocksnadel et al. 1992 [32, 33]. | Austria.  RCT. N=41 women. | Compared the impact of aspirin (80 mg daily until 37 wks; intervention) vs. placebo (controls). | Fetal death rate (miscarriage+SB): RR=0.29 (95% CI: 0.01-6.72)**[NS]**  [0/22 vs. 1/19 in intervention vs. control groups, respectively.] |
| 31. Shenoy et al. 1999 [34]. | India.  RCT. Women (N=163) with PIH, 20-32 wks gestation. | Compared the impact of aspirin (60 mg daily until 38 wks; intervention) vs. placebo (controls). | PMR+IMR: RR=0.47 (95% CI: 0.19-1.18)**[NS]**  [0/79 vs. 13/81 in intervention vs. control groups, respectively.] |
| 32. Sibai et al. 1993a [35]. | USA.  RCT. N=3026. | Compared the impact of aspirin (60 mg daily; intervention) vs. placebo (controls). | Fetal death rate (miscarriage+SB): RR=2.45 (95% CI: 1.02-5.89)**[NS]**  [17/1505 vs. 7/1519 in intervention vs. control groups, respectively.] |
| 33. Subtil et al. (ERASME) 2003 [36] | France, Belgium.  Multicentre RCT. 28 centres in France and 1 in Belgium. Primiparous women (N=3294) 14-20 wks' gestation. Singleton or multiple pregnancy. | Compared the impact of aspirin (100 mg daily until 34 wks; intervention) vs. placebo (controls). | Fetal death rate (miscarriage+SB): RR=1.14 (95% CI: 0.44-2.94)**[NS]**  [9/1645 vs. 8/1660 in intervention vs. control groups, respectively.] |
| 34. Trudinger et al. 1988 [37]. | Australia.  RCT. Women (N=46) with singleton pregnancy at 28-36 wks and concern about fetal welfare, in whom umbilical artery velocity waveform systolic/diastolic ratio > 95^th^ percentile. | Compared the impact of aspirin (150 mg daily; intervention) vs. placebo (controls). | PMR+IMR: 0/22 vs. 0/24 in intervention vs. control groups, respectively. RR not estimable. |
| 35. Tulppala et al. 1997 [38, 39]. | Finland.  RCT. Women (N=66) ~5 wks' gestation with a history of recurrent spontaneous miscarriage. | Compared the impact of aspirin (50 mg daily as soon as positive pregnancy test; intervention) vs. placebo (controls). | Fetal death rate (miscarriage+SB): RR=1.00 (95% CI: 0.48-2.08)**[NS]**  [10/33 vs. 10/33 in intervention vs. control groups, respectively.] |
| 36. Uzan et al. EPREDA 1991 [40]. | France.  RCT. Women (N=323), 15-18 wks' gestation with poor outcome during previous 2 pregnancies, at least 1 being IUGR, or IUGR in 1 previous pregnancy. | Compared the impact of aspirin (150 mg) and dipuridamole (225 mg) daily until delivery (intervention) vs. aspirin placebo (controls). | Fetal death rate (miscarriage+SB): RR=0.47 (95% CI: 0.07-3.26)**[NS]**  [2/156 vs. 2/73 in intervention (anti-platelet) vs. control groups, respectively.] |
| 37. Vainio et al. 2002 [41]. | Finland.  RCT. Women (N=90) at risk of pre-eclampsia or IUGR with abnormal uterine doppler, 12-14 wks' gestation. | Compared the impact of aspirin (0.5 mg/kg daily as soon as positive pregnancy test; intervention) vs. placebo (controls). | Fetal death rate (miscarriage+SB): 0/43 in both groups. RR not estimable. |
| 38. Viinikka et al. 1993 [42] | Finland.  RCT. N=197 women. | Compared the impact of aspirin (50 mg daily; intervention) vs. placebo (controls). | Fetal death rate (miscarriage+SB): RR=3.09 (95% CI: 0.13-74.98)**[NS]**  [1/97 vs 0/100 in intervention vs. control groups, respectively.] |
| 39. Wallenburg et al. 1986 [43]. | Netherlands.  RCT. Primigravid women (N=46) angiotensin II sensitive at 28 wks' gestation with uncomplicated pregnancies, no history of hypertension, cardiovascular or renal disease, DBP < 80 mmHg and taking no drugs except iron. | Compared the impact of aspirin (60 mg daily until delivery; intervention) vs. placebo (controls). | Fetal death rate (miscarriage+SB): RR=3.00 (95% CI: 0.13-70.02)**[NS]**  [1/23 vs 0/23 in intervention vs. control groups, respectively.] |
| 40. Wallenburg et al. 1991a [44]. | Netherlands.  RCT. Women (N=36) with a positive angiotensin II sensitivity test at 28 wks gestation. | Compared the impact of aspirin (60 mg daily from 28-32 wks; intervention) vs. placebo (controls). | Fetal death rate (miscarriage+SB): 0/17 vs. 0/18 in intervention vs. control groups, respectively. RR not estimable. |
| 41. Wang et al.1996 [45] | China.  RCT. Women (N=84) with a singleton pregnancy at high risk of IUGR, 28-34 wks' gestation. | Compared the impact of aspirin (75 mg daily from 28-34 wks, 6-8 wks duration; intervention) vs. placebo (controls). | PMR+IMR: RR=0.12 (95% CI: 0.01-2.20)**[NS]**  [0/40 vs. 4/44 in intervention vs. control groups, respectively.] |
| 42. Yu et al. 2003 [46]. | UK.  RCT. Women (N=560) with singleton pregnancy, 22-24 wks and Doppler pulsatility index > 1.6 (95th percentile). | Compared the impact of aspirin (150 mg daily; intervention) vs. placebo (controls). | Fetal death rate (miscarriage+SB): RR=1.51 (95% CI: 0.43-5.30)**[NS]**  [6/276 vs. 4/278 in intervention vs. control groups, respectively.] |
| 43. Zimmermann et al. 1997 [47] | Finland.  RCT. High-risk women (N=26) with uterine artery bilateral notches on doppler, 22-24 wks gestation. | Compared the impact of aspirin (50 mg daily; intervention) vs. placebo (controls). | Fetal death rate (miscarriage+SB): RR= 0.33 (95% CI: 0.01-7.50)**[NS]**  [0/13 vs. 1/13 in intervention vs. control groups, respectively.] |

References

1. Duley L, Henderson-Smart DJ, Meher S, King JF: **Antiplatelet agents for preventing pre-eclampsia and its complications**. *Cochrane Database Syst Rev* 2007(2):CD004659.

2. **ECPPA: randomised trial of low dose aspirin for the prevention of maternal and fetal complications in high risk pregnant women. ECPPA (Estudo Colaborativo para Prevencao da Pre-eclampsia com Aspirina) Collaborative Group**. *Br J Obstet Gynaecol* 1996, **103**(1):39-47.

3. August P, Helseth G, Edersheim TG, Hutson JM, Druzin M: **Sustained release, low-dose aspirin ameliorates but does not prevent preeclampsia (PE) in a high risk population.** In: *Proceedings of 9th International Congress, International Society for the Study of Hypertension in Pregnancy: 1994; Sydney, Australia.*; 1994.

4. **Low-dose aspirin in prevention and treatment of intrauterine growth retardation and pregnancy-induced hypertension. Italian study of aspirin in pregnancy**. *Lancet* 1993, **341**(8842):396-400.

5. Azar R, Turpin D: **Effect of antiplatelet therapy in women at high risk for pregnancy-induced hypertension.** In: *Proceedings of 7th World Congress of Hypertension in Pregnancy: 1990 October; Perugia, Italy.*; 1990 October.

6. Beaufils M, Uzan S, Donsimoni R, Colau JC: **Prevention of pre-eclampsia by early antiplatelet therapy**. *Lancet* 1985, **1**(8433):840-842.

7. Benigni A, Gregorini G, Frusca T, Chiabrando C, Ballerini S, Valcamonico A, Orisio S, Piccinelli A, Pinciroli V, Fanelli R *et al*: **Effect of low-dose aspirin on fetal and maternal generation of thromboxane by platelets in women at risk for pregnancy-induced hypertension**. *N Engl J Med* 1989, **321**(6):357-362.

8. Byaruhanga RN, Chipato T, Rusakaniko S: **A randomized controlled trial of low-dose aspirin in women at risk from pre-eclampsia**. *Int J Gynaecol Obstet* 1998, **60**(2):129-135.

9. Caritis S, Sibai B, Hauth J, Lindheimer MD, Klebanoff M, Thom E, VanDorsten P, Landon M, Paul R, Miodovnik M *et al*: **Low-dose aspirin to prevent preeclampsia in women at high risk. National Institute of Child Health and Human Development Network of Maternal-Fetal Medicine Units**. *N Engl J Med* 1998, **338**(11):701-705.

10. Caspi E, Raziel A, Sherman D, Arieli S, Bukovski I, Weinraub Z: **Prevention of pregnancy-induced hypertension in twins by early administration of low-dose aspirin: a preliminary report**. *Am J Reprod Immunol* 1994, **31**(1):19-24.

11. Chiaffarino F, Parazzini F, Paladini D, Acaia B, Ossola W, Marozio L, Facchinetti F, Del Giudice A: **A small randomised trial of low-dose aspirin in women at high risk of pre-eclampsia**. *Eur J Obstet Gynecol Reprod Biol* 2004, **112**(2):142-144.

12. **CLASP: a randomised trial of low-dose aspirin for the prevention and treatment of pre-eclampsia among 9364 pregnant women. CLASP (Collaborative Low-dose Aspirin Study in Pregnancy) Collaborative Group**. *Lancet* 1994, **343**(8898):619-629.

13. Cowchock S, Reece EA: **Do low-risk pregnant women with antiphospholipid antibodies need to be treated? Organizing Group of the Antiphospholipid Antibody Treatment Trial**. *Am J Obstet Gynecol* 1997, **176**(5):1099-1100.

14. Davies NJ, Gazvani MR, Farquharson RG, Walkinshaw SA: **Low-dose aspirin in the prevention of hypertensive disorders of pregnancy in relatively low-risk nulliparous women**. *Hypertension in Pregnancy;* 1995, **14**:49-55.

15. Dekker G: **Prediction and prevention of pregnancy-induced hypertensive disorders: a clinical and pathophysiologic study**. *MD thesis.* Rotterdam, The Netherlands: University Medical School; 1989.

16. Essinger S: **The use of low dose acetylsalicylic acid in prevention of pregnancy-induced hypertension [Uso do acido acetilsalicilico em baixa dosagem na prevencao da doenca hipertensiva especifica da gestacao (DHEG)]**. *Revista do Colegio Brasileiro de Cirurgioes* 1992, **19**:58-62.

17. Gallery EDM, Ross MR, Hawkins M, Leslie GI, Gyory AZ: **Low-dose aspirin in high-risk pregnancy**. *Hypertension in Pregnancy* 1997, **16**:229-238.

18. Golding J: **A randomised trial of low dose aspirin for primiparae in pregnancy. The Jamaica Low Dose Aspirin Study Group**. *Br J Obstet Gynaecol* 1998, **105**(3):293-299.

19. Hauth JC, Goldenberg RL, Parker CR, Jr., Philips JB, 3rd, Copper RL, DuBard MB, Cutter GR: **Low-dose aspirin therapy to prevent preeclampsia**. *Am J Obstet Gynecol* 1993, **168**(4):1083-1091; discussion 1091-1083.

20. Herabutya Y, Jetsawangsri T, Saropala N: **The use of low-dose aspirin to prevent preeclampsia**. *Int J Gynaecol Obstet* 1996, **54**(2):177-178.

21. Hermida RC, Ayala DE, Iglesias M, Mojon A, Silva I, Ucieda R, Fernandez JR: **Time-dependent effects of low-dose aspirin administration on blood pressure in pregnant women**. *Hypertension* 1997, **30**(3 Pt 2):589-595.

22. Kincaid-Smith P: **Trial to evaluate the role of aspirin (60mg) in the prevention of idiopathic intrauterine growth retardation and pregnancy induced hypertension in primigravid women with abnormal uterine artery waveforms on Doppler examination at 22-24 weeks gestation. Personal communication.** In*.*; October 30 1991.

23. McParland P, Pearce JM, Chamberlain GV: **Doppler ultrasound and aspirin in recognition and prevention of pregnancy-induced hypertension**. *Lancet* 1990, **335**(8705):1552-1555.

24. Quenby S, Farquharson R, Ramsden G: **The obstetric outcome of patients with positive anticardiolipin antibodies: aspirin vs no treatment.** In: *Proceedings of 26th British Congress of Obstetrics and Gynaecology: 1992 July 7-10; Manchester, UK.*; 1992 July 7-10.

25. Rai U, et al: **Role of low dose aspirin in PIH**. *Journal of Obstetrics and Gynaecology of India* 1993:883-886.

26. Railton A, Davey A: **Aspirin and dipyridamole in the prevention of pre-eclampsia: effect on plasma prostanoids 6 keto PG1a and TXB2 and clinical outcome of pregnancy**. In: *Proceedings of the 6th world congress of the International Society for the Study of Hypertension in Pregnancy: 1988 May 22-26; Montreal, Quebec, Canada*; 1988 May 22-26.

27. Railton A, Davey DA: **Aspirin and dipyridamole in the prevention of pre-eclampsia: effect on plasma 6 keto PGF1alpha and TxB2 and clinical outcome of pregnancy**. In: *Proceedings of 1st European Congress on Prostaglandins in Reproduction: 1988; Vienna, Austria*; 1988.

28. Rogov V, Tareeva I, Sidorova S, Androsova S: **Prevention of pregnancy complications with acetylsalicylic acid (ASA) and dipyridamol (DP) in women with chronic glomerulonephritis (CGN) and essential hypertension (EH)**. In: *Proceedings of 9th International Congress of the International Society for the Study of Hypertension in Pregnancy: 1994 March 15-18; Sydney, Australia*; 1994 March 15-18.

29. Rotchell YE, Cruickshank JK, Gay MP, Griffiths J, Stewart A, Farrell B, Ayers S, Hennis A, Grant A, Duley L *et al*: **Barbados Low Dose Aspirin Study in Pregnancy (BLASP): a randomised trial for the prevention of pre-eclampsia and its complications**. *Br J Obstet Gynaecol* 1998, **105**(3):286-292.

30. Roy UK, Pan S: **A study of use of low dose aspirin in prevention of pregnancy induced hypertension**. *J Indian Med Assoc* 1994, **92**(6):188-191.

31. Schiff E, Peleg E, Goldenberg M, Rosenthal T, Ruppin E, Tamarkin M, Barkai G, Ben-Baruch G, Yahal I, Blankstein J *et al*: **The use of aspirin to prevent pregnancy-induced hypertension and lower the ratio of thromboxane A2 to prostacyclin in relatively high risk pregnancies**. *N Engl J Med* 1989, **321**(6):351-356.

32. Schrocksnadel H, Sitte B, Alge A, Steckel-Berger G, Daxenbichler G, Dapunt O: **[Low-dose aspirin in prevention and therapy of hypertension in pregnancy]**. *Gynakol Geburtshilfliche Rundsch* 1992, **32 Suppl 1**:90-91.

33. Schrocksnadel H, Sitte B, Alge A, Steckel-Berger G, Schwegel P, Pastner E, Daxenbichler G, Hansen H, Dapunt O: **Low-dose aspirin in primigravidae with positive roll-over test**. *Gynecol Obstet Invest* 1992, **34**(3):146-150.

34. Shenoy S, Chandrika D, Pisharody R: **RCT of low dose aspirin to prevent the progression of pregnancy induced hypertension grade A to B**. *Journal of Clinical Epidemiology;* 1999, **52**(Suppl 1):28S.

35. Sibai BM, Caritis SN, Thom E, Klebanoff M, McNellis D, Rocco L, Paul RH, Romero R, Witter F, Rosen M *et al*: **Prevention of preeclampsia with low-dose aspirin in healthy, nulliparous pregnant women. The National Institute of Child Health and Human Development Network of Maternal-Fetal Medicine Units**. *N Engl J Med* 1993, **329**(17):1213-1218.

36. Subtil D, Goeusse P, Puech F, Lequien P, Biausque S, Breart G, Uzan S, Marquis P, Parmentier D, Churlet A: **Aspirin (100 mg) used for prevention of pre-eclampsia in nulliparous women: the Essai Regional Aspirine Mere-Enfant study (Part 1)**. *BJOG* 2003, **110**(5):475-484.

37. Trudinger B, Cook CM, Thompson R, Giles W, Connelly A: **Low-dose aspirin improves fetal weight in umbilical placental insufficiency**. *Lancet* 1988, **2**(8604):214-215.

38. Tulppala M, Marttunen M, Soderstrom-Anttila V, Ailus K, Palosuo T, Ylikorkala O: **Low dose aspirin in the prevention of miscarriage in women with unexplained or autoimmune related recurrent miscarriage: effect on prostacyclin and thromboxane A2 production**. *Human Reproduction;* 1997, **12**:191.

39. Tulppala M, Marttunen M, Soderstrom-Anttila V, Foudila T, Ailus K, Palosuo T, Ylikorkala O: **Low-dose aspirin in prevention of miscarriage in women with unexplained or autoimmune related recurrent miscarriage: effect on prostacyclin and thromboxane A2 production**. *Hum Reprod* 1997, **12**(7):1567-1572.

40. Uzan S, Beaufils M, Breart G, Bazin B, Capitant C, Paris J: **Prevention of fetal growth retardation with low-dose aspirin: findings of the EPREDA trial**. *Lancet* 1991, **337**(8755):1427-1431.

41. Vainio M, Kujansuu E, Iso-Mustajarvi M, Maenpaa J: **Low dose acetylsalicylic acid in prevention of pregnancy-induced hypertension and intrauterine growth retardation in women with bilateral uterine artery notches**. *BJOG* 2002, **109**(2):161-167.

42. Viinikka L, Hartikainen-Sorri AL, Lumme R, Hiilesmaa V, Ylikorkala O: **Low dose aspirin in hypertensive pregnant women: effect on pregnancy outcome and prostacyclin-thromboxane balance in mother and newborn**. *Br J Obstet Gynaecol* 1993, **100**(9):809-815.

43. Wallenburg HC, Dekker GA, Makovitz JW, Rotmans P: **Low-dose aspirin prevents pregnancy-induced hypertension and pre-eclampsia in angiotensin-sensitive primigravidae**. *Lancet* 1986, **1**(8471):1-3.

44. Wallenburg HC, Dekker GA, Makovitz JW, Rotmans N: **Effect of low-dose aspirin on vascular refractoriness in angiotensin-sensitive primigravid women**. *Am J Obstet Gynecol* 1991, **164**(5 Pt 1):1169-1173.

45. Wang Z, Li W: **A prospective randomized placebo-controlled trial of low-dose aspirin for prevention of intra-uterine growth retardation**. *Chin Med J (Engl)* 1996, **109**(3):238-242.

46. Yu CK, Papageorghiou AT, Parra M, Palma Dias R, Nicolaides KH: **Randomized controlled trial using low-dose aspirin in the prevention of pre-eclampsia in women with abnormal uterine artery Doppler at 23 weeks' gestation**. *Ultrasound Obstet Gynecol* 2003, **22**(3):233-239.

47. Zimmermann P, Eirio V, Koskinen J, Niemi K, Nyman R, Kujansuu E, al e: **Effect of low dose aspirin treatment on vascular resistance in the uterine, uteroplacental, renal and umbilical arteries - a prospective longitudinal study on a high risk population with persistent notch in the uterine arteries**. *European Journal of Ultrasound;* 1997, **5**:17-30.
