# Supplementary material for: Reducing stillbirths: prevention and management of medical disorders and infections during pregnancy
Source: BMC Pregnancy Childbirth. 2009 May 7;9(Suppl 1):S4. doi: 10.1186/1471-2393-9-S1-S4 (PMC2679410; doi:10.1186/1471-2393-9-S1-S4)
Supplement: Additional file 10 — Web Table 10. Component studies in Askie et al. 2007 meta-analysis: impact of anti-platelet agents. Component studies in Askie et al. 2007 meta-analysis reporting impact on stillbirths/perinatal mortality [file 1471-2393-9-S1-S4-S10.doc]

**Component studies in Askie et al. 2007 [1] meta-analysis: impact of anti-platelet agents**

| **Source** | **Location and Type of Study** | **Intervention** | **Stillbirths / Perinatal Outcomes** |
| --- | --- | --- | --- |
| 1. August et al. 1994 [2]. | USA.  RCT. N=49 women. | Compared the impact of aspirin (100 mg sustained release daily until 37 wks; intervention) vs. placebo (controls). | Fetal death rate (miscarriage+SB): RR=1.04 (95% CI: 0.07-15.73)  [1/24 vs. 1/25 in intervention vs. control groups, respectively.] |
| 2. Authors not known 1993 [3]. | Italy.  RCT. N=1,172 women. | Compared the impact of aspirin (50 mg daily; intervention) vs. no treatment (controls). | Fetal death rate (miscarriage+SB): RR=0.79 (95% CI: 0.37-1.66)**[NS]**  [13/634 vs. 14/538 in intervention vs. control groups, respectively.] |
| 3. Byaruhanga et al. 1998 [4] | Zimbabwe.  RCT. Women (N=250) 20-28 wks gestation with a history of pre-eclampsia in a previous pregnancy, especially if at < 32 wks, or chronic hypertension. | Compared the impact of aspirin (75 mg daily; intervention) vs. placebo (controls). | PMR: RR=0.41 (95% CI: 0.15-1.12)  [5/114 vs. 13/122 in intervention vs. control groups, respectively.] |
| 4. Caritis et al. 1998 [5]. | USA.  RCT. N=3216 women. | Compared the impact of aspirin (60 mg daily; intervention) vs. placebo (controls). | Fetal death rate (miscarriage+SB): RR=0.71 (95% CI: 0.48-1.06)[**NS]**  [40/1612 vs. 56/1604 in intervention vs. control groups, respectively.] |
| 5. Chiaffarino et al. 2004 [6]. | Italy.  RCT. Women (N=40) < 14 wks' gestation with chronic HT +/- nephropathy or history of severe pre-eclampsia or eclampsia or IUGR or stillbirth. | Compared the impact of aspirin (100 mg daily until delivery; intervention) vs. no treatment (controls). | Fetal death rate (miscarriage+SB): RR=0.59 (95% CI: 0.06-5.96)**[NS]**  [1/16 2/19 in intervention vs. control groups, respectively.] |
| 6. CLASP (Collaborative Low-dose Aspirin Study in Pregnancy) 1994 [7]. | 16 countries.  Multicenter RCT. Women (N=9364) women at risk of pre-eclampsia or IUGR, or women with established pre-eclampsia or IUGR, 12-32 wks' gestation. | Compared the impact of aspirin (60 mg daily until delivery; intervention) vs. placebo (controls). | PMR+IMR: RR=0.80 (95% CI: 0.59-1.07)**[NS]**  [77/4123 vs. 97/4134 in intervention vs. control groups, respectively.] |
| 7. ECPPA 1996 [8] | Brazil.  Multicenter RCT. 12 hospitals and 182 doctors’ offices. Women (N=1009) at high risk for PE, 12-32 wks’ gestation. | Compared the impact of aspirin (60 mg daily until delivery; intervention) vs. placebo (controls). | PMR: 7.0% vs. 6.3% in intervention vs. control groups, respectively **[NS]**. |
| 8. Ferrier et al. [9] | Study details unavailable (conference proceedings). |  |  |
| 9. Gallery et al. 1997 [10] | Australia.  RCT. Women (N=120) at high risk of pre-eclampsia because of one of the following: pre-existing hypertension (BP greater ≥ 140/90 prior to pregnancy on at least 2 occasions, or on anti-hypertensive therapy), renal disease, previous early severe PE. | Compared the impact of aspirin (100 mg modified release daily from 17-19 weeks until delivery; intervention) vs. placebo (controls). | Fetal death rate (miscarriage+SB): RR=7.78 (95% CI: 0.43-141.0)  [54/58 vs. 0/50 in intervention vs. control groups, respectively.] |
| 10. Golding J 1998 [11]. | Jamaica.  RCT. Primiparous women (N=6275) 12-32 wks and no contraindication to aspirin. | Compared the impact of aspirin (60 mg daily until delivery; intervention) vs. placebo (controls). | PMR: RR=0.84 (95% CI: 0.63-1.11)**[NS]**  [86/3023 vs. 103/3026 in intervention vs. control groups, respectively.] |
| 11. Grover et al [12] | Study details unavailable. Full text not accessible. |  |  |
| 12. Hauth et al. 1993 [13] | USA.  RCT. Primiparous women (N=604) at 24 wks’ gestation, in single antenatal clinic. | Compared the impact of aspirin (60 mg daily from 22 wks; intervention) vs. placebo (controls). | Fetal death rate (miscarriage+SB): RR=1.00 (95% CI: 0.06-15.91)**[NS]**  [1/302 vs. 1/302 in intervention vs. control groups, respectively.] |
| 13. Hermida et al. 2003 [14]. | Spain.  RCT. Women (N=107) age 18-40, < 16 wks' gestation and at moderate risk of pre-eclampsia (history of PIH, PE, chronic HT, cardiovascular or endocrine problem, bleeding or endocrine disease). | Compared the impact of aspirin (100 mg daily; intervention) vs. placebo (controls), administered to three groups of patients at different times of the day. | PMR+IMR: 0/50 in both the groups. RR not estimable. |
| 14. Kincaid-Smith 1995; Ferrier 1996 [9, 15]. | Australia.  RCT. Primigravid women (N=52) with abnormal uterine artery waveforms on doppler examination at 22-24 wks. | Compared the impact of aspirin (60 mg daily; intervention) vs. placebo (controls). | Fetal death rate (miscarriage+SB): 0/27 vs. 0/25 in intervention vs. control groups, respectively. |
| 15. Louden 1992 [16]. | UK (Nottingham, England).  RCT. N=46 women (N=12 non-pregnant volunteers, N=18 normotensive primigravidae <18 wks gestation, N=16 women with PIH at mean gestation 38 wks. | Compared the impact of aspirin (60 mg/daily; intervention) vs. placebo (controls) | Fetal death rate (miscarriage+SB): No deaths in any group. RR not estimable. |
| 16. Michael and Walters [17] | Study details unavailable. Full text not accessible (book chapter). |  |  |
| 17. Morris et al. 1996 [18]. | Australia.  RCT. N = 186 women with abnormal uteroplacental resistance | Compared the impact of low-dose aspirin (100 mg/day) (intervention) vs. placebo (controls). | Pregnancy complications: no significant reduction in intervention group vs. controls. |
| 18. Railton , Davey 1988, 1988 [19]*.* | South Africa.  RCT. Women (N=44) with elevated mid-trimester BP, 12-28 wks' gestation, DBP 80-105 mmHg, and otherwise normal. | Compared the impact of aspirin (81 mg; intervention group #1), or aspirin (81 mg) plus dipyridamole (200 mg)(intervention group #2) daily vs. no treatment (controls). | Fetal death rate (miscarriage+SB): RR=0.93 (95% CI: 0.09-9.45)**[NS]**  [2/30 vs. 1/14 in intervention groups vs. control group, respectively.] |
| 19. Rivas-Echeverria 2000 [20] | Study details unavailable. Full text not accessible. |  |  |
| 20. Rogers et al. 1999 [21]. | China (Hong Kong).  RCT. Normotensive, primigravid women (N=500) recruited in 2nd trimester with 80 mm Hg > MAP < 106 mm Hg, screened by DinamapTM measuring MAP in the left lateral position after rest with cutoff value of 60 mm Hg. | Compared impact of low-dose aspirin to calcium supplementation and control groups. | Study findings unavailable. Full text not accessible. |
| 21. Rotchell et al. 1998 [22] | Barbados.  RCT. N=3675 women analysed. | Compared the impact of aspirin (75 mg controlled release daily until delivery; intervention) vs. placebo (controls). | Fetal death rate (miscarriage+SB): RR=1.08 (95% CI: 0.64-1.81)**[NS]**  [29/1834 vs. 27/1841 in intervention vs. control groups, respectively.] |
| 22. Schiff et al. 1989 [23]. | Israel.  RCT. Women (N=65) with either twin pregnancy or a history of PE, and a positive roll-over test at 28-29 wks' gestation. | Compared the impact of aspirin (100 mg daily; intervention) vs. placebo (controls). | Fetal death rate (miscarriage+SB): 0/34 vs. 0/32 in intervention vs. control groups, respectively. RR not estimable. |
| 23. Seki et al. 1999 [24] | Japan.  RCT. N=40 women at high risk of PE. | Compared the impact of a thromboxane synthetase inhibitor, ozagrel hydrochloride (400 mg/day orally from 20 wks until delivery; intervention), vs. placebo (controls). | Study findings unavailable. Full text not accessible. |
| 24. Sibai et al. 1993a [25]. | USA.  RCT. N=3026. | Compared the impact of aspirin (60 mg daily; intervention) vs. placebo (controls). | Fetal death rate (miscarriage+SB): RR=2.45 (95% CI: 1.02-5.89)**[NS]**  [17/1505 vs. 7/1519 in intervention vs. control groups, respectively.] |
| 25. Subtil et al. (ERASME) 2003 [26] | France, Belgium.  Multicentre RCT. 28 centres in France and 1 in Belgium. Primiparous women (N=3294) 14-20 wks' gestation. Singleton or multiple pregnancy. | Compared the impact of aspirin (100 mg daily until 34 wks; intervention) vs. placebo (controls). | Fetal death rate (miscarriage+SB): RR=1.14 (95% CI: 0.44-2.94)**[NS]**  [9/1645 vs. 8/1660 in intervention vs. control groups, respectively.] |
| 26. Uzan 1989 [27] | Study findings unavailable. Full text not accessible. |  |  |
| 27. Uzan et al. EPREDA 1991 [28]. | France.  RCT. Women (N=323), 15-18 wks' gestation with poor outcome during previous 2 pregnancies, at least 1 being IUGR, or IUGR in 1 previous pregnancy. | Compared the impact of aspirin (150 mg) and dipuridamole (225 mg) daily until delivery (intervention) vs. aspirin placebo (controls). | Fetal death rate (miscarriage+SB): RR=0.47 (95% CI: 0.07-3.26)**[NS]**  [2/156 vs. 2/73 in intervention (anti-platelet) vs. control groups, respectively.] |
| 28. Vainio et al. 2002 [29]. | Finland.  RCT. Women (N=90) at risk of pre-eclampsia or IUGR with abnormal uterine doppler, 12-14 wks' gestation. | Compared the impact of aspirin (0.5 mg/kg daily as soon as positive pregnancy test; intervention) vs. placebo (controls). | Fetal death rate (miscarriage+SB): 0/43 in both groups. RR not estimable. |
| 29. Wang et al.1996 [30] | China.  RCT. Women (N=84) with a singleton pregnancy at high risk of IUGR, 28-34 wks' gestation. | Compared the impact of aspirin (75 mg daily from 28-34 wks, 6-8 wks duration; intervention) vs. placebo (controls). | PMR+IMR: RR=0.12 (95% CI: 0.01-2.20)**[NS]**  [0/40 vs. 4/44 in intervention vs. control groups, respectively.] |
| 30. Yu et al. 2003 [31]. | UK.  RCT. Women (N=560) with singleton pregnancy, 22-24 wks and Doppler pulsatility index > 1.6 (95th percentile). | Compared the impact of aspirin (150 mg daily; intervention) vs. placebo (controls). | Fetal death rate (miscarriage+SB): RR=1.51 (95% CI: 0.43-5.30)**[NS]**  [6/276 vs. 4/278 in intervention vs. control groups, respectively.] |
| 31. Zimmermann et al. 1997 [32] | Finland.  RCT. High-risk women (N=26) with uterine artery bilateral notches on doppler, 22-24 wks gestation. | Compared the impact of aspirin (50 mg daily; intervention) vs. placebo (controls). | Fetal death rate (miscarriage+SB): RR= 0.33 (95% CI: 0.01-7.50)**[NS]**  [0/13 vs. 1/13 in intervention vs. control groups, respectively.] |

References

1. Askie LM, Duley L, Henderson-Smart DJ, Stewart LA: **Antiplatelet agents for prevention of pre-eclampsia: a meta-analysis of individual patient data**. *Lancet* 2007, **369**(9575):1791-1798.

2. August P, Helseth G, Edersheim TG, Hutson JM, Druzin M: **Sustained release, low-dose aspirin ameliorates but does not prevent preeclampsia (PE) in a high risk population.** In: *Proceedings of 9th International Congress, International Society for the Study of Hypertension in Pregnancy: 1994; Sydney, Australia.*; 1994.

3. **Low-dose aspirin in prevention and treatment of intrauterine growth retardation and pregnancy-induced hypertension. Italian study of aspirin in pregnancy**. *Lancet* 1993, **341**(8842):396-400.

4. Byaruhanga RN, Chipato T, Rusakaniko S: **A randomized controlled trial of low-dose aspirin in women at risk from pre-eclampsia**. *Int J Gynaecol Obstet* 1998, **60**(2):129-135.

5. Caritis S, Sibai B, Hauth J, Lindheimer MD, Klebanoff M, Thom E, VanDorsten P, Landon M, Paul R, Miodovnik M *et al*: **Low-dose aspirin to prevent preeclampsia in women at high risk. National Institute of Child Health and Human Development Network of Maternal-Fetal Medicine Units**. *N Engl J Med* 1998, **338**(11):701-705.

6. Chiaffarino F, Parazzini F, Paladini D, Acaia B, Ossola W, Marozio L, Facchinetti F, Del Giudice A: **A small randomised trial of low-dose aspirin in women at high risk of pre-eclampsia**. *Eur J Obstet Gynecol Reprod Biol* 2004, **112**(2):142-144.

7. **CLASP: a randomised trial of low-dose aspirin for the prevention and treatment of pre-eclampsia among 9364 pregnant women. CLASP (Collaborative Low-dose Aspirin Study in Pregnancy) Collaborative Group**. *Lancet* 1994, **343**(8898):619-629.

8. **ECPPA: randomised trial of low dose aspirin for the prevention of maternal and fetal complications in high risk pregnant women. ECPPA (Estudo Colaborativo para Prevencao da Pre-eclampsia com Aspirina) Collaborative Group**. *Br J Obstet Gynaecol* 1996, **103**(1):39-47.

9. Ferrier C, North R, Kincaid-Smith P: **Low dose aspirin delays the onset of pre-eclampsia in pregnancies with abnormal uteroplacental circulation**. In: *Proceedings of the 10th World Congress of the International Society for the Study of Hypertension in Pregnancy: 1996 August 4-8; Seattle, Washington, USA*; 1996 August 4-8.

10. Gallery EDM, Ross MR, Hawkins M, Leslie GI, Gyory AZ: **Low-dose aspirin in high-risk pregnancy**. *Hypertension in Pregnancy* 1997, **16**:229-238.

11. Golding J: **A randomised trial of low dose aspirin for primiparae in pregnancy. The Jamaica Low Dose Aspirin Study Group**. *Br J Obstet Gynaecol* 1998, **105**(3):293-299.

12. Grover V, Shabnam S, Kumari S: **Evaluation of dipyridamole & aspirin in prevention and management of intrauterine growth retardation.** *J Perinat Med* 1991, **19**:104.

13. Hauth J, Goldenberg R, Philips J, Copper R, DuBard M, Cutter G: **Low-dose aspirin therapy to prevent preeclampsia: safety considerations**. *American Journal of Obstetrics and Gynecology;* 1993, **168**:389.

14. Hermida RC, Ayala DE, Iglesias M: **Administration time-dependent effects of aspirin on blood pressure in pregnant women**

*Hypertension* 2003, **2**(651-656).

15. Kincaid-Smith P, North RA, Fairly KF, Kloss M, Ihle BU: **Prevention of pre-eclampsia in women with renal disease: a prospective randomized trial of heparin and dipyridamole.** *Nephrology* 1995, **1**:297-300.

16. Louden KA, Broughton Pipkin F, Symonds EM, Tuohy P, O'Callaghan C, Heptinstall S, Fox S, Mitchell JR: **A randomized placebo-controlled study of the effect of low dose aspirin on platelet reactivity and serum thromboxane B2 production in non-pregnant women, in normal pregnancy, and in gestational hypertension**. *Br J Obstet Gynaecol* 1992, **99**(5):371-376.

17. Michael CA, Walters BNJ: **Low-dose aspirin in the prevention of pre-eclampsia: current evaluation**. In: *Maternal physiology and pathology The current status of gynaecology and obstetrics series.* Edited by Teoh ES, Shan Ratnam S, Macnaughton M, vol. 4. Carnforth: Parthenon Pub Group Ltd.; 1993: 183-189.

18. Morris JM, Fay RA, Ellwood DA, Cook CM, Devonald KJ: **A randomized controlled trial of aspirin in patients with abnormal uterine artery blood flow**. *Obstet Gynecol* 1996, **87**(1):74-78.

19. Railton A, Davey A: **Aspirin and dipyridamole in the prevention of pre-eclampsia: effect on plasma prostanoids 6 keto PG1a and TXB2 and clinical outcome of pregnancy**. In: *Proceedings of the 6th world congress of the International Society for the Study of Hypertension in Pregnancy: 1988 May 22-26; Montreal, Quebec, Canada*; 1988 May 22-26.

20. Rivas-Echeverria CA, Echeverria Y, Molina L, Novoa D: **Synergic use of aspirin, fish oil and vitamins C and E for the prevention of pre-eclampsia**. *Hypertens Preg* 2000, **19**:30.

21. Rogers MS, Fung HY, Hung CY: **Calcium and low-dose aspirin prophylaxis in women at high risk of pregnancy-induced hypertension**. *Hypertens Pregnancy* 1999, **18**(2):165-172.

22. Rotchell YE, Cruickshank JK, Gay MP, Griffiths J, Stewart A, Farrell B, Ayers S, Hennis A, Grant A, Duley L *et al*: **Barbados Low Dose Aspirin Study in Pregnancy (BLASP): a randomised trial for the prevention of pre-eclampsia and its complications**. *Br J Obstet Gynaecol* 1998, **105**(3):286-292.

23. Schiff E, Peleg E, Goldenberg M, Rosenthal T, Ruppin E, Tamarkin M, Barkai G, Ben-Baruch G, Yahal I, Blankstein J *et al*: **The use of aspirin to prevent pregnancy-induced hypertension and lower the ratio of thromboxane A2 to prostacyclin in relatively high risk pregnancies**. *N Engl J Med* 1989, **321**(6):351-356.

24. Seki H, Kuromaki K, Takeda S, Kinoshita K, Satoh K: **Trial of prophylactic administration of TXA2 synthetase inhibitor, ozagrel hydrochloride, for preeclampsia**. *Hypertens Pregnancy* 1999, **18**(2):157-164.

25. Sibai BM, Caritis SN, Thom E, Klebanoff M, McNellis D, Rocco L, Paul RH, Romero R, Witter F, Rosen M *et al*: **Prevention of preeclampsia with low-dose aspirin in healthy, nulliparous pregnant women. The National Institute of Child Health and Human Development Network of Maternal-Fetal Medicine Units**. *N Engl J Med* 1993, **329**(17):1213-1218.

26. Subtil D, Goeusse P, Puech F, Lequien P, Biausque S, Breart G, Uzan S, Marquis P, Parmentier D, Churlet A: **Aspirin (100 mg) used for prevention of pre-eclampsia in nulliparous women: the Essai Regional Aspirine Mere-Enfant study (Part 1)**. *BJOG* 2003, **110**(5):475-484.

27. Uzan S, Beaufils M, Bazin B, Danays T: **Idiopathic recurrent fetal growth retardation and aspirin-dipyridamole therapy**. *Am J Obstet Gynecol* 1989, **160**(3):763-764.

28. Uzan S, Beaufils M, Breart G, Bazin B, Capitant C, Paris J: **Prevention of fetal growth retardation with low-dose aspirin: findings of the EPREDA trial**. *Lancet* 1991, **337**(8755):1427-1431.

29. Vainio M, Kujansuu E, Iso-Mustajarvi M, Maenpaa J: **Low dose acetylsalicylic acid in prevention of pregnancy-induced hypertension and intrauterine growth retardation in women with bilateral uterine artery notches**. *BJOG* 2002, **109**(2):161-167.

30. Wang Z, Li W: **A prospective randomized placebo-controlled trial of low-dose aspirin for prevention of intra-uterine growth retardation**. *Chin Med J (Engl)* 1996, **109**(3):238-242.

31. Yu CK, Papageorghiou AT, Parra M, Palma Dias R, Nicolaides KH: **Randomized controlled trial using low-dose aspirin in the prevention of pre-eclampsia in women with abnormal uterine artery Doppler at 23 weeks' gestation**. *Ultrasound Obstet Gynecol* 2003, **22**(3):233-239.

32. Zimmermann P, Eirio V, Koskinen J, Niemi K, Nyman R, Kujansuu E, al e: **Effect of low dose aspirin treatment on vascular resistance in the uterine, uteroplacental, renal and umbilical arteries - a prospective longitudinal study on a high risk population with persistent notch in the uterine arteries**. *European Journal of Ultrasound;* 1997, **5**:17-30.
