# Supplementary material for: Reducing stillbirths: prevention and management of medical disorders and infections during pregnancy
Source: BMC Pregnancy Childbirth. 2009 May 7;9(Suppl 1):S4. doi: 10.1186/1471-2393-9-S1-S4 (PMC2679410; doi:10.1186/1471-2393-9-S1-S4)
Supplement: Additional file 11 — Web Table 11. Component studies in Empson et al. 2005 meta-analysis: Impact of treatment during pregnancy for lupus anti-coagulant or anti-phospholipid syndrome. Component studies in Empson et al. 2005 meta-analysis reporting impact on stillbirths/perinatal mortality [file 1471-2393-9-S1-S4-S11.doc]

**Web Table 11. Component studies in Empson et al. 2005 [1] meta-analysis: Impact of treatment during pregnancy for lupus anti-coagulant or anti-phospholipid syndrome**

| **Source** | **Location and Type of Study** | | | **Intervention** | **Stillbirths / Perinatal Outcomes** | |
| --- | --- | --- | --- | --- | --- | --- |
| **Aspirin vs. placebo or usual care** | | | | | | |
| 1. Cowchock and Reece 1997 [2]. | | USA.  RCT. N=19 women with anti-phospholipid anti-bodies and ≤ 2 previous miscarriages with no other anti-phospholipid antibody related complications. | Compared the impact of aspirin (81 mg daily; intervention) vs. usual care (controls). | | | PMR+IMR: RR=2.25 (95% CI: 0.10-49.04)**[NS]**  [1/11 vs. 0/8 in intervention vs. control groups, respectively.] |
| 2. Pattison et al. 2000. Merrill 2001 [3, 4] | | New Zealand.  RCT. N=40 women with ≥3 fetal losses and persistently positive results for anti-phospholipid antibodies. | Compared the impact of aspirin (75 mg daily; intervention) vs. placebo (controls). | | | Fetal death rate (miscarriage+SB): RR=1.33 (95% CI: 0.34-5.21)**[NS]**  [4/20 vs. 3/20 in intervention vs. control groups, respectively.] |
| 3. Tulppala 1997 [5, 6]. | | Finland.  RCT. N=82 women. | Compared the impact of aspirin (50 mg daily; intervention) vs. placebo (controls). | | | Fetal death rate (miscarriage+SB): RR=1.00 (95% CI: 0.60-1.68)**[NS]** |
| ***Heparin (LMWH and unfractionated) plus aspirin vs. aspirin or IVIG*** | | | | | | |
| 4. Farquharson et al. 2002 [7] | UK. Single centre.  Non-blinded, non-placebo-controlled RCT. N=98 women. | | | Compared the impact of subcutaneous LMWH 5000 IU/day plus aspirin 75 mg/day (intervention) vs. aspirin 75 mg/day (controls). | Fetal death (miscarriage+SB): RR=0.78 (95% CI: 0.39-1.57) **[NS]**  [11/51 vs. 13/47 in intervention vs. control groups, respectively]. | |
| 5. Kutteh 1996a [8] | USA (Texas). Single centre.  Non-blinded, non-placebo-controlled quasi-RCT. N=50 women. | | | Compared the impact on pregnancy loss of subcutaneous heparin 5000 IU 2x/day plus aspirin 81 mg/day (intervention) vs. aspirin 81 mg/day (controls). | Fetal death (miscarriage+SB): RR=0.36 (95% CI: 0.15-0.84).  [5/25 vs. 14/25 in intervention vs. control groups, respectively]. | |
| 6. Rai et al. 1997 [9] | UK (London). Single centre.  Non-blinded, non-placebo-controlled RCT. N=90 women. | | | Compared the impact on pregnancy loss of subcutaneous calcium heparin 5000 IU 2x/day plus aspirin 75 mg/day (intervention) vs. aspirin 75 mg/day alone (controls). | Fetal death (miscarriage+SB): RR=0.50 (95% CI: 0.30-0.84).  [13/45 vs. 26/45 in intervention vs. control groups, respectively]. | |
| 7. Triolo et al. 2003 [10] | Italy. Single centre.  Non-blinded, non-placebo-controlled RCT. N=40 women. | | | Compared the impact on pregnancy loss of LMW heparin (Seleparina) 5700 IU/day and aspirin 75 mg/day (intervention) vs. IVIG 400 mg/kg/day for 2 days then single monthly dose (controls). | Fetal death (miscarriage+SB): RR=0.37 (95% CI: 0.12-1.16) **[NS]**  [3/19 vs. 9/21 in intervention vs. control groups, respectively]. | |
| ***High-dose heparin plus aspirin versus low-dose heparin plus aspirin*** | | | | | | |
| 8. Kutteh et al. 1996b. [11] | USA (Texas). Single centre.  Non-blinded, non-placebo-controlled quasi-randomised trial. N=50 women. | | | Compared the impact on pregnancy loss of heparin 5000 units twice daily sc adjusted to maintain the PTT at 1.2 to 1.5 times the baseline (high-dose) plus aspirin 81 mg/day (intervention) vs. heparin 5000 units twice daily adjusted to maintain the PTT at the upper limit of normal (low-dose) plus aspirin 81 mg/day (controls). | Fetal death (miscarriage+SB): RR=0.83 (95% CI: 0.29-2.38) **[NS]**  [5/25 vs. 6/25 in intervention vs. control groups, respectively]. | |
| ***Prednisone and aspirin vs. heparin and aspirin*** | | | | | | |
| 9. Cowchock et al. 1992 [12] | USA (Pennsylvania, Connecticut, Utah, Georgia). Multicentre, hospital-based.  Non-blinded, non-placebo-controlled RCT. N=45 women. | | | Compared the impact on pregnancy loss of prednisone 20 mg 2x/day plus aspirin 80 mg/day (intervention) vs. subcutaneous heparin 10,000 IU 2x/day plus aspirin 80 mg/day (controls). | Fetal death (miscarriage+SB): RR=1.17 (95% CI: 0.47-2.93) **[NS]**  [6/19 vs. 7/26 in intervention versus control groups, respectively]. | |
| ***IVIG (+/- heparin and aspirin) vs. heparin (LMWH or unfractionated) and aspirin*** | | | | | | |
| 10. Branch et al. 2000 [13] | USA (Salt Lake City, Utah). Multicentre.  Double-blind, placebo-controlled RCT. N=16 women. | | | Compared the impact on pregnancy loss of IVIG (10%) 1 g/kg (intervention) plus heparin 7500 IU 2x/day plus aspirin 81 mg/day vs. placebo (albumin 5%) plus plus heparin 7500 IU 2x/day plus aspirin 81 mg/day (controls), on 2 days every 4 wks. | Fetal death (miscarriage+SB): RR not estimable.  [0/7 vs. 0/9 in intervention vs. control groups, respectively]. | |
| 11. Triolo et al. 2003 [10] | Italy. Single centre.  Non-blinded, non-placebo-controlled RCT. N=40 women. | | | Assessed the impact on pregnancy loss of IVIG 400 mg/kg/day for 2 consecutive days then single monthly dose (intervention) vs. LMW heparin (Seleparina) 5700 IU/day and aspirin 75 mg/day (controls). | Fetal death (miscarriage+SB): RR=2.71 (95% CI: 0.86-8.57) [NS]  [9/21 vs. 3/19 in intervention vs. control groups, respectively]. | |
| 12. Vaquero et al. 2001. [14] | Italy. Two centres.  Non-blinded, non-placebo-controlled quasi-RCT. N=82 women. | | | Compared the impact on pregnancy loss of IVIG 0.5 g/kg 2 days/mo (intervention) vs. aspirin 100 mg/day and prednisone 15-20 mg/day decreasing to 10-15 mg/day after wk 28 (controls). | Fetal death (miscarriage+SB): RR=0.94 (95% CI: 0.42-2.12) **[NS]**  [12/53 vs. 7/29 in IVIG versus aspirin + prednisone groups]. | |

References

1. Empson M, Lassere M, Craig J, Scott J: **Prevention of recurrent miscarriage for women with antiphospholipid antibody or lupus anticoagulant**. *Cochrane Database Syst Rev* 2005(2):CD002859.

2. Cowchock S, Reece EA: **Do low-risk pregnant women with antiphospholipid antibodies need to be treated? Organizing Group of the Antiphospholipid Antibody Treatment Trial**. *Am J Obstet Gynecol* 1997, **176**(5):1099-1100.

3. Pattison NS, Chamley LW, Birdsall M, Zanderigo AM, Liddell HS, McDougall J: **Does aspirin have a role in improving pregnancy outcome for women with the antiphospholipid syndrome? A randomized controlled trial**. *Am J Obstet Gynecol* 2000, **183**(4):1008-1012.

4. Merrill JT: **Appropriate management of antiphospholipid-related pregnancy in women without lupus who have low titer autoantibodies**. *Curr Rheumatol Rep* 2001, **3**(4):269-270.

5. Tulppala M, Marttunen M, Soderstrom-Anttila V, Ailus K, Palosuo T, Ylikorkala O: **Low dose aspirin in the prevention of miscarriage in women with unexplained or autoimmune related recurrent miscarriage: effect on prostacyclin and thromboxane A2 production**. *Human Reproduction;* 1997, **12**:191.

6. Tulppala M, Marttunen M, Soderstrom-Anttila V, Foudila T, Ailus K, Palosuo T, Ylikorkala O: **Low-dose aspirin in prevention of miscarriage in women with unexplained or autoimmune related recurrent miscarriage: effect on prostacyclin and thromboxane A2 production**. *Hum Reprod* 1997, **12**(7):1567-1572.

7. Farquharson RG, Quenby S, Greaves M: **Antiphospholipid syndrome in pregnancy: a randomized, controlled trial of treatment**. *Obstet Gynecol* 2002, **100**(3):408-413.

8. Kutteh WH: **Antiphospholipid antibody-associated recurrent pregnancy loss: treatment with heparin and low-dose aspirin is superior to low-dose aspirin alone**. *Am J Obstet Gynecol* 1996, **174**(5):1584-1589.

9. Rai R, Cohen H, Dave M, Regan L: **Randomised controlled trial of aspirin and aspirin plus heparin in pregnant women with recurrent miscarriage associated with phospholipid antibodies (or antiphospholipid antibodies)**. *BMJ* 1997, **314**(7076):253-257.

10. Triolo G, Ferrante A, Ciccia F, Accardo-Palumbo A, Perino A, Castelli A, Giarratano A, Licata G: **Randomized study of subcutaneous low molecular weight heparin plus aspirin versus intravenous immunoglobulin in the treatment of recurrent fetal loss associated with antiphospholipid antibodies**. *Arthritis Rheum* 2003, **48**(3):728-731.

11. Kutteh WH, Ermel LD: **A clinical trial for the treatment of antiphospholipid antibody-associated recurrent pregnancy loss with lower dose heparin and aspirin**. *Am J Reprod Immunol* 1996, **35**(4):402-407.

12. Cowchock FS, Reece EA, Balaban D, Branch DW, Plouffe L: **Repeated fetal losses associated with antiphospholipid antibodies: a collaborative randomized trial comparing prednisone with low-dose heparin treatment**. *Am J Obstet Gynecol* 1992, **166**(5):1318-1323.

13. Branch DW, Peaceman AM, Druzin M, Silver RK, El-Sayed Y, Silver RM, Esplin MS, Spinnato J, Harger J: **A multicenter, placebo-controlled pilot study of intravenous immune globulin treatment of antiphospholipid syndrome during pregnancy. The Pregnancy Loss Study Group**. *Am J Obstet Gynecol* 2000, **182**(1 Pt 1):122-127.

14. Vaquero E, Lazzarin N, Valensise H, Menghini S, Di Pierro G, Cesa F, Romanini C: **Pregnancy outcome in recurrent spontaneous abortion associated with antiphospholipid antibodies: a comparative study of intravenous immunoglobulin versus prednisone plus low-dose aspirin**. *Am J Reprod Immunol* 2001, **45**(3):174-179.
