# Supplementary material for: Reducing stillbirths: prevention and management of medical disorders and infections during pregnancy
Source: BMC Pregnancy Childbirth. 2009 May 7;9(Suppl 1):S4. doi: 10.1186/1471-2393-9-S1-S4 (PMC2679410; doi:10.1186/1471-2393-9-S1-S4)
Supplement: Additional file 13 — Web Table 13. Component studies in Gates et al. 2002 meta-analysis: Impact of thromboprophylaxis during pregnancy. Component studies in Gates et al. 2002 meta-analysis reporting impact on stillbirths/perinatal mortality [file 1471-2393-9-S1-S4-S13.doc]

**Web Table 13. Component studies in Gates et al. 2002 [1] meta-analysis: Impact of thromboprophylaxis during pregnancy**

| **Source** | **Location and Type of Study** | **Intervention** | **Stillbirths / Perinatal Outcomes** |
| --- | --- | --- | --- |
| ***LMW heparin versus unfractionated heparin*** | | | |
| 1. Pettila et al. 1999 [2] | Finland. 8 centres.  RCT. N=107 women. | Compared the effect of LMWH (dalteparin “Fragmin”) 1x daily (starting dose 5000 or 7500 IU, dose adjusted for anti-Xa measurements) (intervention) vs. unfractionated heparin (7500 iu, adjusted according to APTT target values) 2x daily (controls) from before wk 20 of gestation until 6 wks after delivery. | Fetal death (miscarriage + SB): RR=1.10 (95% CI: 0.07-17.12) **[NS]**  [1/50 vs. 1/55 in intervention vs. control groups, respectively]. |
| ***Aspirin + heparin versus aspirin alone*** | | | |
| 2. Rai et al. 1997 [3] | UK. One centre.  RCT. N=90 women. | Compared the impact of calcium heparin (5000 IU 12 hourly) plus low dose aspirin (intervention), vs. low dose aspirin alone (controls). Aspirin started at positive pregnancy test, heparin at randomisation. | Fetal death (miscarriage + SB): RR=0.50 (95% CI: 0.30-0.84).  [13/45 vs. 26/45 in intervention vs. control groups, respectively]. |
| ***Unfractionated heparin versus no treatment*** | | | |
| 3. Howell et al. 1983 [4] | UK. One centre.  RCT. N=40 women. | Compared the impact on fetal deaths of calcium heparin antenatally (10,000 IU twice daily) and for 6 wks postpartum (8000iu 2x daily) (intervention) vs. 6 wks postpartum administration only (controls). | Fetal death (miscarriage + SB): RR=1.00 (95% CI: 0.07-14.90) **[NS]**  [1/20 vs. 1/20 in intervention vs. control groups, respectively]. |

References

1. Gates S, Brocklehurst P, Davis LJ: **Prophylaxis for venous thromboembolic disease in pregnancy and the early postnatal period**. *Cochrane Database Syst Rev* 2002(2):CD001689.

2. Pettila V, Kaaja R, Leinonen P, Ekblad U, Kataja M, Ikkala E: **Thromboprophylaxis with low molecular weight heparin (dalteparin) in pregnancy**. *Thromb Res* 1999, **96**(4):275-282.

3. Rai R, Cohen H, Dave M, Regan L: **Randomised controlled trial of aspirin and aspirin plus heparin in pregnant women with recurrent miscarriage associated with phospholipid antibodies (or antiphospholipid antibodies)**. *BMJ* 1997, **314**(7076):253-257.

4. Howell R, Fidler J, Letsky E, de Swiet M: **The risks of antenatal subcutaneous heparin prophylaxis: a controlled trial**. *Br J Obstet Gynaecol* 1983, **90**(12):1124-1128.
