# Supplementary material for: Reducing stillbirths: prevention and management of medical disorders and infections during pregnancy
Source: BMC Pregnancy Childbirth. 2009 May 7;9(Suppl 1):S4. doi: 10.1186/1471-2393-9-S1-S4 (PMC2679410; doi:10.1186/1471-2393-9-S1-S4)
Supplement: Additional file 14 — Web Table 14. Component studies in Rumbold et al. 2008: impact of anti-oxidant supplements. Component studies in Rumbold et al. 2008 meta-analysis reporting impact on stillbirths/perinatal mortality [file 1471-2393-9-S1-S4-S14.doc]

**Web Table 14. Component studies in Rumbold et al. 2008 [1]: impact of anti-oxidant supplements**

| **Source** | **Location and Type of Study** | **Intervention** | **Stillbirths / Perinatal Outcomes** |
| --- | --- | --- | --- |
| 1. Chappell et al. 1999 [2]. | UK (London).  RCT. N = 283 women between 16 – 22 weeks’ gestation with an abnormal uterine artery Doppler waveform, or a history of pre-eclampsia, eclampsia or HELLP in the preceding pregnancy. | Compared impact of anti-oxidant (1000 mg vitamin C plus 400 IU vitamin E daily; intervention) vs. placebo (controls). | Fetal death rate (miscarriage+SB): RR=0.50 (95% CI: 0.05-5.49)**[NS]**  **[**1/141 vs. 2/142 in intervention vs. control groups, respectively.] |
| 1. Poston et al 2006 [3]. | UK.  Double-blind RCT. N=2395 women (N=1196 intervention; N=1199 controls). | Compared impact of anti-oxidant (1000 mg vitamin C and 400 IU vitamin E daily) vs. placebo (controls). | Fetal death rate (miscarriage+SB): RR=1.59 (95% CI: 0.99-2.56)**[NS]**  [43/1393 vs. 27/1391 in intervention vs. control groups, respectively.] |
| 1. Rumbold et al. 2006, for the ACTS Study Group [4]. | Australia.  Double-blind RCT. Nulliparous women (N=1877; N=935 intervention, N=942 controls) with a singleton pregnancy. | Compared impact of anti-oxidant (1000 mg vitamin C and 400 IU vitamin E daily; intervention) vs. placebo (controls). | Fetal death rate (miscarriage+SB): RR=0.85 (95% CI: 0.38-1.89)**[NS]**  [11/935 vs. 13/942 in intervention vs. control groups, respectively.] |
| 1. Steyn et al. 2002 [5]. | South Africa.  Double-blind RCT. N= 200 women (N=100 intervention, N=100 controls). | Compared impact of anti-oxidant (250 mg vitamin C 2x/daily until 34 wks gestation; intervention) vs. placebo (controls). | Fetal death rate (miscarriage+SB): 1.38 (95% CI: 0.58-3.27)**[NS]**  [11/100 vs. 8/100 in intervention vs. control groups, respectively.] |

References

1. Rumbold A, Duley L, Crowther CA, Haslam RR: **Antioxidants for preventing pre-eclampsia**. *Cochrane Database Syst Rev* 2008(1):CD004227.

2. Chappell L, Seed P, Briley A, Kelly F, Lee R, Hunt B: **Effect of antioxidants on the occurrence of pre-eclampsia in women at increased risk: a randomised controlled trial**. *Lancet* 1999, **354**:810-816.

3. Poston L, Briley AL, Seed PT, Kelly FJ, Shennan AH, for the Vitamins in Pre-eclampsia (VIP) Trial Consortium: **Vitamin C and vitamin E in pregnant women at risk for pre-eclampsia (VIP trial): randomised placebo-controlled trial.** *Lancet* 2006, **367**(9517):1145-1154.

4. Rumbold AR, Crowther CA, Haslam RR, Dekker GA, Robinson JS: **Vitamins C and E and the risks of preeclampsia and perinatal complications**. *N Engl J Med* 2006, **354**(17):1796-1806.

5. Steyn PS, Odendaal HJ, Schoeman J, Stander C, Fanie N, Grove D: **A randomised, double-blind placebo-controlled trial of ascorbic acid supplementation for the prevention of preterm labour**. *J Obstet Gynaecol* 2003, **23**(2):150-155.
