# Supplementary material for: Reducing stillbirths: prevention and management of medical disorders and infections during pregnancy
Source: BMC Pregnancy Childbirth. 2009 May 7;9(Suppl 1):S4. doi: 10.1186/1471-2393-9-S1-S4 (PMC2679410; doi:10.1186/1471-2393-9-S1-S4)
Supplement: Additional file 15 — Web Table 15. Component studies in Rumbold and Crowther 2005: Impact of anti-oxidants. Component studies in Rumbold and Crowther 2005 meta-analysis reporting impact on stillbirths/perinatal mortality [file 1471-2393-9-S1-S4-S15.doc]

**Web Table 15. Component studies in Rumbold and Crowther 2005 [1, 2]: Impact of anti-oxidants**

| **Source** | **Location and Type of Study** | **Intervention** | **Stillbirths / Perinatal Outcomes** |
| --- | --- | --- | --- |
| 1. Chappell et al. 1999 [3]. | UK (London).  RCT. N = 283 women between 16 – 22 weeks’ gestation with an abnormal uterine artery Doppler waveform, or a history of pre-eclampsia, eclampsia or HELLP in the preceding pregnancy. | Compared impact of anti-oxidant (1000 mg vitamin C plus 400 IU vitamin E daily; intervention) vs. placebo (controls). | Fetal death rate (miscarriage+SB): RR=0.50 (95% CI: 0.05-5.49)**[NS]**  [1/141 vs. 2/142 in intervention vs. control groups, respectively.] |
| 1. Gulmezoglu et al. 1997 [4] | South Africa.  Double-blind RCT. N=56 women (N=27 intervention, N=29 controls). | Compared the impact of an anti-oxidant complex (500 mg vitamin C, 400 IU vitamin E, and 100 mg allopurinol, administered twice daily; intervention) vs. placebo (controls). | SBR: RR=0.84 (95% CI: 0.36-1.93)**[NS]**  [7/27 vs. 9/29 in intervention vs. control groups, respectively.] |
| 1. Steyn et al. 2003 [5] | South Africa.  Double-blind RCT. N=200 women. | Compared the impact of vitamin C (250 mg vitamin C twice daily; intervention) vs. placebo (controls) from trial entry until 34 wks' gestation. | SBR: RR=3.00 (95% CI: 0.12-72.77)**[NS]**  [1/100 vs. 0/100 in intervention vs. control groups, respectively.] |

**References**

1. Rumbold A, Crowther CA: **Vitamin E supplementation in pregnancy**. *Cochrane Database Syst Rev* 2005(2):CD004069.

2. Rumbold A, Crowther CA: **Vitamin C supplementation in pregnancy**. *Cochrane Database Syst Rev* 2005(2):CD004072.

3. Chappell L, Seed P, Briley A, Kelly F, Lee R, Hunt B: **Effect of antioxidants on the occurrence of pre-eclampsia in women at increased risk: a randomised controlled trial**. *Lancet* 1999, **354**:810-816.

4. Gulmezoglu AM, Hofmeyr GJ, Oosthuisen MM: **Antioxidants in the treatment of severe pre-eclampsia: an explanatory randomised controlled trial**. *Br J Obstet Gynaecol* 1997, **104**(6):689-696.

5. Steyn PS, Odendaal HJ, Schoeman J, Stander C, Fanie N, Grove D: **A randomised, double-blind placebo-controlled trial of ascorbic acid supplementation for the prevention of preterm labour**. *J Obstet Gynaecol* 2003, **23**(2):150-155.
