# Supplementary material for: Reducing stillbirths: prevention and management of medical disorders and infections during pregnancy
Source: BMC Pregnancy Childbirth. 2009 May 7;9(Suppl 1):S4. doi: 10.1186/1471-2393-9-S1-S4 (PMC2679410; doi:10.1186/1471-2393-9-S1-S4)
Supplement: Additional file 16 — Web Table 16. Component studies in Drakeley et al. 2003 meta-analysis: Impact of cervical cerclage on stillbirths and perinatal mortality. Component studies in Drakeley et al. 2003 meta-analysis reporting impact on stillbirths/perinatal mortality [file 1471-2393-9-S1-S4-S16.doc]

**Web Table 16. Component studies in Drakeley et al. 2003 [1] meta-analysis: Impact of cervical cerclage on stillbirths and perinatal mortality**

| **Source** | **Location and Type of Study** | **Intervention** | **Stillbirths / Perinatal Outcomes** |
| --- | --- | --- | --- |
| ***Elective cerclage versus no cerclage or bed rest*** | | | |
| 1. Althuisius et al. 2001a [2] | Netherlands.  RCT. N=70 women at risk of pre-term labour based on pregnancy history. | Compared the impact on perinatal mortality of cervical cerclage (McDonald technique with polyester thread; intervention) vs. no cerclage (controls). | PMR: RR=0.60 (95% CI: 0.07-5.43) **[NS]**  [1/24 vs. 3/43 in intervention vs. control groups, respectively]. |
| 2. Lazar et al. 1984 [3] | France.  RCT. N=506 women at moderate risk of pre-term delivery based on score recalculated at each visit. Excluded high- and low-risk women. | Compared the impact on perinatal mortality of elective cerclage (McDonald technique with nylon; intervention) vs. no cerclage (controls). | PMR: RR=1.78 (95% CI: 0.16-19.46) **[NS]**  [2/268 vs. 1/238 in intervention vs. control groups, respectively]. |
| 3. MRC/RCOG Working Party on Cervical Cerclage. 1993 [4] | Multiple countries.. Multicentre.  RCT. N=1292 women at risk of pre-term delivery. | Compared the impact on perinatal mortality of cerclage (80% McDonald technique and 74% mersilene; intervention) vs. no cerclage unless clearly indicated (controls). | PMR: RR=0.66 (95% CI: 0.32-1.37) **[NS]**  [12/647 vs. 18/645 in intervention vs. control groups, respectively]. |
| 4. Rush et al. 1984 [5] | South Africa. Teaching hospital.  RCT. N=194 women at high risk. N=8 women recruited had therapeutic cerclage. 37% had previous pre-term deliveries. | Compared the impact on perinatal mortality of elective cerclage (McDonald technique with monofilament nylon; intervention) vs. no cerclage (controls). | PMR: RR=1.02 (95% CI: 0.42-2.46) **[NS]**  [9/96 vs. 9/98 in intervention vs. control groups, respectively]. |
| ***Cerclage versus no cerclage for short cervix by ultrasound*** | | | |
| 5. Althuisius et al. 2001b [6] | Netherlands.  RCT. N=35 women who developed short cervix by ultrasound who initially were randomised to "no cerclage" in a prophylactic cerclage study. | Compared the impact on perinatal mortality of secondary randomisation to therapeutic cerclage using McDonald technique with polyester thread (intervention) vs. no cerclage (controls) if cervical length <25mm <27 wks' gestation.  All women who had secondary randomisation (short cervix) were prescribed bed rest. | PMR: RR=0.28 (95% CI: 0.01-6.51) **[NS]**  [0/19 vs. 1/16 in intervention vs. control groups, respectively]. |
| 6. Rust et al. 2001[7] | USA.  RCT. N=113 women at risk of pre-term birth by pregnancy history underwent transvaginal ultrasound assessment. Any low risk women who had ultrasound evaluation were also assessed for abnormality of the lower uterine segment. | Compared the impact of elective cerclage with McDonald technique (intervention) versus no cerclage (controls). | PMR: RR=1.05 (95% CI: 0.40-2.81) [NS]  [7/55 vs. 7/58 in intervention vs. control groups, respectively]. |

References

1. Drakeley AJ, Roberts D, Alfirevic Z: **Cervical stitch (cerclage) for preventing pregnancy loss in women**. *Cochrane Database Syst Rev* 2003(1):CD003253.

2. Althuisius SM, Dekker GA, van Geijn HP, Bekedam DJ, Hummel P: **Cervical incompetence prevention randomized cerclage trial (CIPRACT): study design and preliminary results**. *Am J Obstet Gynecol* 2000, **183**(4):823-829.

3. Lazar P, Gueguen S, Dreyfus J, Renaud R, Pontonnier G, Papiernik E: **Multicentred controlled trial of cervical cerclage in women at moderate risk of preterm delivery**. *Br J Obstet Gynaecol* 1984, **91**(8):731-735.

4. **Final report of the Medical Research Council/Royal College of Obstetricians and Gynaecologists multicentre randomised trial of cervical cerclage. MRC/RCOG Working Party on Cervical Cerclage**. *Br J Obstet Gynaecol* 1993, **100**(6):516-523.

5. Rush RW, Isaacs S, McPherson K, Jones L, Chalmers I, Grant A: **A randomized controlled trial of cervical cerclage in women at high risk of spontaneous preterm delivery**. *Br J Obstet Gynaecol* 1984, **91**(8):724-730.

6. Althuisius SM, Dekker GA, Hummel P, Bekedam DJ, van Geijn HP: **Final results of the Cervical Incompetence Prevention Randomized Cerclage Trial (CIPRACT): therapeutic cerclage with bed rest versus bed rest alone**. *Am J Obstet Gynecol* 2001, **185**(5):1106-1112.

7. Rust OA, Atlas RO, Reed J, van Gaalen J, Balducci J: **Revisiting the short cervix detected by transvaginal ultrasound in the second trimester: why cerclage therapy may not help**. *Am J Obstet Gynecol* 2001, **185**(5):1098-1105.
