# Supplementary material for: Reducing stillbirths: prevention and management of medical disorders and infections during pregnancy
Source: BMC Pregnancy Childbirth. 2009 May 7;9(Suppl 1):S4. doi: 10.1186/1471-2393-9-S1-S4 (PMC2679410; doi:10.1186/1471-2393-9-S1-S4)
Supplement: Additional file 17 — Web Table 17. Component studies in Jorgensen et al. 2007 meta-analysis: Impact of cervical cerclage. Component studies in Jorgensen et al. 2007 meta-analysis reporting impact on stillbirths/perinatal mortality [file 1471-2393-9-S1-S4-S17.doc]

**Web Table 17. Component studies in Jorgensen et al. 2007 [1] meta-analysis: Impact of cervical cerclage**

| **Source** | **Location and Type of Study** | **Intervention** | **Stillbirths / Perinatal Outcomes** |
| --- | --- | --- | --- |
| 1. Althuisius et al. 2001b. [2] | Netherlands.  RCT. N=35 women who developed short cervix by ultrasound who initially were randomised to "no cerclage" in a prophylactic cerclage study. | Compared the impact on perinatal mortality of secondary randomisation to therapeutic cerclage using McDonald technique with polyester thread (intervention) vs. no cerclage (controls) if cervical length <25mm <27 wks' gestation.  All women who had secondary randomisation (short cervix) were prescribed bed rest. | Pregnancy loss or death before hospital discharge: OR=0.10 (95% CI: 0.01-1.02) **[NS]**  [0/19 vs. 3/16 in intervention vs. control groups, respectively]. |
| 2. Berghella et al. 2004 [3] | USA.  RCT. N=57 women. (N=28 intervention group, N=29 controls). | Compared the impact on perinatal outcomes of McDonald technique cerclage plus bedrest (intervention) vs. bedrest alone (controls). | Pregnancy loss or death before hospital discharge: OR=0.76 (95% CI: 0.16-3.62) **[NS]**  [3/28 vs. 4/29 in intervention vs. control groups, respectively]. |
| 3. Ezechi et al. 2004 [4] | Nigeria.  RCT. N=81 women. (N=39 intervention group, N=42 controls). | Compared the impact on perinatal outcomes of McDonald technique cerclage (intervention) vs. no intervention (controls). | Pregnancy loss or death before hospital discharge: OR=0.14 (95% CI: 0.01-2.31) **[NS]**  [0/39 vs. 2/42 in intervention vs. control groups, respectively]. |
| 4. MRC/RCOG Working Party on Cervical Cerclage. 1993 [5] | UK/international. Multicentre.  RCT. N=1292 women deemed at risk of pre-term delivery. | Compared the impact on perinatal mortality of cerclage (80% McDonald technique and 74% mersilene; intervention) vs. no cerclage unless clearly indicated (controls). | Pregnancy loss or death before hospital discharge: OR=0.78 (95% CI: 0.53-1.13) **[NS]**  [53/635 vs. 66/629 in intervention vs. control groups, respectively]. |
| 5. Rush et al. 1984 [6] | South Africa. Teaching hospital.  RCT. N=194 women at high risk of pre-term delivery (N=8 women recruited already had cerclage; 37% had previous pre-term deliveries). | Compared the impact on perinatal mortality of elective McDonald cerclage using monofilament nylon (intervention) vs. no cerclage (controls). | Pregnancy loss or death before hospital discharge: OR=1.02 (95% CI: 0.39-2.69) **[NS]**  [9/96 vs. 9/98 in intervention vs. control groups, respectively]. |
| 6. Rust et al. 2001 [7] | USA.  RCT. N=207 women at risk of pre-term birth by history; N=113 underwent transvaginal ultrasound assessment. Any low-risk women who had ultrasound evaluation were also assessed for abnormality of the lower uterine segment. | Compared the impact of elective McDonald technique cerclage (intervention) vs. no cerclage (controls). | Pregnancy loss or death before hospital discharge: OR=1.76 (95% CI: 0.69-4.52) **[NS]**  [12/104 vs. 7/103 in intervention vs. control groups, respectively]. |
| 7. To et al. 2004 [8] | UK, Brazil, South Africa, Slovenia, Greece and Chile.  RCT. N=253 women (N=127 intervention, N=126 controls). | Compared the impact on perinatal outcomes of Shirodkar suture (intervention) vs. expectant management (controls). | Pregnancy loss or death before hospital discharge: OR=0.73 (95% CI: 0.30-1.77) **[NS]**  [9/127 vs. 12/126 in intervention vs. control groups, respectively]. |

References

1. Jorgensen AL, Alfirevic Z, Tudur Smith C, Williamson PR: **Cervical stitch (cerclage) for preventing pregnancy loss: individual patient data meta-analysis**. *BJOG* 2007, **114**(12):1460-1476.

2. Althuisius SM, Dekker GA, Hummel P, Bekedam DJ, van Geijn HP: **Final results of the Cervical Incompetence Prevention Randomized Cerclage Trial (CIPRACT): therapeutic cerclage with bed rest versus bed rest alone**. *Am J Obstet Gynecol* 2001, **185**(5):1106-1112.

3. Berghella V, Odibo AO, Tolosa JE: **Cerclage for prevention of preterm birth in women with a short cervix found on transvaginal ultrasound examination: a randomized trial**. *Am J Obstet Gynecol* 2004, **191**(4):1311-1317.

4. Ezechi OC, Kalu BK, Nwokoro CA: **Prophylactic cerclage for the prevention of preterm delivery**. *Int J Gynaecol Obstet* 2004, **85**(3):283-284.

5. **Final report of the Medical Research Council/Royal College of Obstetricians and Gynaecologists multicentre randomised trial of cervical cerclage. MRC/RCOG Working Party on Cervical Cerclage**. *Br J Obstet Gynaecol* 1993, **100**(6):516-523.

6. Rush RW, Isaacs S, McPherson K, Jones L, Chalmers I, Grant A: **A randomized controlled trial of cervical cerclage in women at high risk of spontaneous preterm delivery**. *Br J Obstet Gynaecol* 1984, **91**(8):724-730.

7. Rust OA, Atlas RO, Reed J, van Gaalen J, Balducci J: **Revisiting the short cervix detected by transvaginal ultrasound in the second trimester: why cerclage therapy may not help**. *Am J Obstet Gynecol* 2001, **185**(5):1098-1105.

8. To MS, Alfirevic Z, Heath VC, Cicero S, Cacho AM, Williamson PR, Nicolaides KH: **Cervical cerclage for prevention of preterm delivery in women with short cervix: randomised controlled trial**. *Lancet* 2004, **363**(9424):1849-1853.
