# Supplementary material for: Reducing stillbirths: prevention and management of medical disorders and infections during pregnancy
Source: BMC Pregnancy Childbirth. 2009 May 7;9(Suppl 1):S4. doi: 10.1186/1471-2393-9-S1-S4 (PMC2679410; doi:10.1186/1471-2393-9-S1-S4)
Supplement: Additional file 18 — Web Table 18. Component studies in King and Flenady 2002 meta-analysis: impact of anti-biotics in high-risk pregnancy. Component studies in King and Flenady 2002 meta-analysis reporting impact on stillbirths/perinatal mortality [file 1471-2393-9-S1-S4-S18.doc]

**Web Table 18. Component studies in King and Flenady 2002 [1] meta-analysis: impact of anti-biotics in high-risk pregnancy**

| **Source** | **Location and Type of Study** | **Intervention** | **Stillbirths / Perinatal Outcomes** |
| --- | --- | --- | --- |
| 1. Cox et al. 1996 [2, 3] | USA (Texas), Dallas hospital.  RCT. Women (N=86) 24-34 wks gestation (mean 30 wks), in pre-term labour (cervical change with contractions). Exclusions: ruptured membranes, fetal or maternal complications necessitating delivery. | Assessed the impact of IV ampicillin 2 g with sulbactam 1g every 6 hr x 8 doses, followed by ampicillin - clavulanate 250mg every 8 hr x 5 days (intervention), vs. placebo (controls). | PMR: RR=3.15 (95% CI: 0.13-75.05)**[NS]**  [1/40 vs. 0/42 in intervention vs. control groups, respectively.] |
| 2. McGregor et al. 1991 [4] | USA.  RCT. Women (N=117) <35 wks gestation (mean 30.5 wks) in pre-term labour receiving tocolysis. Exclusions: ruptured membranes, multiple pregnancies, suspected fetal compromise, maternal infection and other maternal medical conditions. | Assessed the impact of IV clindamycin 900mg every 8 hr x 9 doses followed by oral clindamycin 300mg every 6 hr x 4 days (intervention) vs. placebo (controls). | PMR: RR=4.72 (95% CI: 0.23-96.01)**[NS]**  [2/53 vs. 0/50 in intervention vs. control groups, respectively.] |
| 3. Newton et al. 1991 [5] | USA.  RCT. Women (N=91) 24-33 wks gestation (mean 30 wks) in pre-term labour receiving tocolysis. | Assessed the impact of IV ampicillin 2 g/sulbactam 1g every 6 hr x 12 doses plus oral indomethacin (50mg load, then 25mg every 6 hr x 7 doses)(intervention), vs. placebo (controls). | PMR: RR=4.79 (95% CI: 0.24- 97.14)**[NS]**  [2/47 vs. 0/45 in intervention vs. control groups, respectively.] |
| 4. Norman et al. 1994 [6] | South Africa.  Multicenter RCT. 3 centres. Women (N=82) 26-34 wks gestation (mean 31 wks) in pre-term labour receiving tocolysis. Exclusions: ruptured membranes, antepartum haemorrhage, infection, maternal medical conditions, multiple pregnancy. | Assessed the impact of IV Ampicillin 1 g every 6 hr x 4 doses followed by oral amoxicillin 500mg every 8 hr x 5 days, plus metronidazole 1gm stat then 400mg orally every 8 hours for 5 days (intervention), vs. placebo (controls). | PMR: RR=0.88 (95% CI: 0.13-5.97)**[NS]**  [2/43 vs. 2/38 in intervention vs. control groups, respectively.] |
| 5. ORACLE II Kenyon et al. 2001 [7] | UK.  Multicenter RCT. 161 centres (2x2 factorial design). Women (N=6295) < 37 wks gestation. (mean 31 wks) with intact membranes, suspected pre-term labour, and clinical uncertainty as to whether to use anti-biotics. | Compared the impact of oral administration of: 1. 325 mg co-amoxiclav + 250mg erythromycin; 2. 325 mg co-amoxiclav plus erythromycin placebo; 3. 250mg erythromycin plus co-amoxiclav placebo; 4. co-amoxiclav placebo plus erythromycin placebo. All study medication was given orally every 6 hr for 10 days or until delivery, whichever was earlier. | PMR: RR=1.09 (95% CI: 0.77-1.55)**[NS]**  [128/4685 vs. 39/1556 in intervention vs. control groups, respectively.] |
| 6. Oyarzun et al. 1998 [8] | Chile.  RCT. Women (N=196) in suspected labour, 22-36 wks gestation, singleton pregnancy, with intact membranes, and cervical dilatation <5 cm. | Oral amoxicillin 250 mg every 8 hr and erythromycin 500 mg orally every 6 hr for 7 days (intervention), vs. placebo (controls). | PMR: RR=2.31 (95% CI: 0.21-24.97)**[NS]**  **[**2/78 vs. 1/90 in intervention vs. control groups, respectively.] |
| 7. Romero et al. 1993 [9] | USA.  Multicenter RCT. 6 centres. Women (N=277) 24-34 wks gestation (mean 30.5 wks) in pre-term labour receiving tocolysis. Exclusions: ruptured membranes, multiple pregnancies, suspected fetal compromise, suspected imminent delivery, suspected maternal infection, recent anti-biotic use. | Assessed the impact of IV ampicillin 1 g every 4 hrs with concomitant IV erythromycin 250 mg every 6 hr, both for 48 hr, followed by oral amoxicillin 250 mg every 8 hr and erythromycin 333 mg every 8 hr for 5 days (intervention) vs. placebo (control). | PMR: RR=5.49 (95% CI: 0.27-113.36)**[NS]**  [2/131 vs. 0/144 in intervention vs. control groups, respectively.] |
| 8. Svare et al. 1997 [10] | Denmark.  RCT. Women (N=112) in suspected labour, 26-34 wks gestation, singleton pregnancy, cervical dilatation < 4cm. | Assessed the impact of IV ampicillin 2 g every 6 hr for 24 hr, followed by pivampicin 500mg orally for 7 days, plus IV metronidazole 500 mg every 8 hr for 24 hr, followed by metronidazole 400 mg orally every 8 hr for 7 days (intervention), vs. placebo (controls). | PMR: 0/59 vs. 0/51 in intervention vs. control groups, respectively. RR not estimable. |
| 9. Watts et al. 1994 [11] | USA.  RCT. Women (N=56) < 34 wks gestation (mean 31 wks) in pre-term labour receiving tocolysis. | Assessed the impact of IV mezlocillin 3 g IV every 6 hrs for 5 days and oral erythromycin 333 mg every 8 hr for 10 days. | PMR: RR=2.61 (95% CI: 0.11-61.51)**[NS]**  [1/30 vs. 0/26 in intervention vs. control groups, respectively.] |

References

1. King J, Flenady V: **Prophylactic antibiotics for inhibiting preterm labour with intact membranes**. *Cochrane Database Syst Rev* 2002(4):CD000246.

2. Cox SM, Bohman VR, Sherman ML, Leveno KJ: **Randomized investigation of antimicrobials for the prevention of preterm birth**. *Am J Obstet Gynecol* 1996, **174**(1 Pt 1):206-210.

3. Cox SM, Boham V, Sherman ML, Leveno KJ: **Single-center randomized placebo controlled trial of antimicrobials for prevention of preterm birth [abstract]**. *American Journal of Obstetrics and Gynecology* 1994, **170**:388.

4. McGregor JA, French JI, Seo K: **Adjunctive clindamycin therapy for preterm labor: results of a double-blind, placebo-controlled trial**. *Am J Obstet Gynecol* 1991, **165**(4 Pt 1):867-875.

5. Newton ER, Shields L, Ridgway LE, 3rd, Berkus MD, Elliott BD: **Combination antibiotics and indomethacin in idiopathic preterm labor: a randomized double-blind clinical trial**. *Am J Obstet Gynecol* 1991, **165**(6 Pt 1):1753-1759.

6. Norman K, Pattinson RC, de Souza J, de Jong P, Moller G, Kirsten G: **Ampicillin and metronidazole treatment in preterm labour: a multicentre, randomised controlled trial**. *Br J Obstet Gynaecol* 1994, **101**(5):404-408.

7. Kenyon SL, Taylor DJ, Tarnow-Mordi W: **Broad-spectrum antibiotics for spontaneous preterm labour: the ORACLE II randomised trial**. *Lancet* 2001, **357**:991-996.

8. Oyarzun E, Gomez R, Rioseco A, Gonzalez P, Gutierrez P, Donoso E, Montiel F: **Antibiotic treatment in preterm labor and intact membranes: a randomized, double-blinded, placebo-controlled trial**. *J Matern Fetal Med* 1998, **7**(3):105-110.

9. Romero R, Sibai B, Caritis S, Paul R, Depp R, Rosen M, Klebanoff M, Sabo V, Evans J, Thom E *et al*: **Antibiotic treatment of preterm labor with intact membranes: a multicenter, randomized, double-blinded, placebo-controlled trial**. *Am J Obstet Gynecol* 1993, **169**(4):764-774.

10. Svare J, Langhoff-Roos J, Andersen LF, Kryger-Baggesen N, Borch-Christensen H, Heisterberg L, Kristensen J: **Ampicillin-metronidazole treatment in idiopathic preterm labour: a randomised controlled multicentre trial**. *Br J Obstet Gynaecol* 1997, **104**(8):892-897.

11. Watts DH, Krohn MA, Hillier SL, Eschenbach DA: **Randomized trial of antibiotics in addition to tocolytic therapy to treat preterm labor**. *Infect Dis Obstet Gynecol* 1994, **1**(5):220-227.
