# Supplementary material for: Reducing stillbirths: prevention and management of medical disorders and infections during pregnancy
Source: BMC Pregnancy Childbirth. 2009 May 7;9(Suppl 1):S4. doi: 10.1186/1471-2393-9-S1-S4 (PMC2679410; doi:10.1186/1471-2393-9-S1-S4)
Supplement: Additional file 19 — Web Table 19. Component studies in McDonald et al. 2007 meta-analysis: impact of anti-biotics in high-risk pregnancy. Component studies in McDonald et al. 2007 meta-analysis reporting impact on stillbirths/perinatal mortality [file 1471-2393-9-S1-S4-S19.doc]

**Web Table 19. Component studies in McDonald et al. 2007 [1] meta-analysis: impact of anti-biotics in high-risk pregnancy**

| **Source** | **Location and Type of Study** | **Intervention** | **Stillbirths / Perinatal Outcomes** |
| --- | --- | --- | --- |
| 1. Lamont et al. (2003) [2] | UK.  Cluster RCT. Asymptomatic pregnant women (N=409; N=208 intervention, N=201 controls), 13-20 wks’ gestation with BV or intermediate flora by Nugent’s criteria. | Compared the impact of 5 g of 2% clindamycin intravaginal cream (intervention) vs. placebo (controls) for 3 nights. Treatment given for 7 more days if vaginal swab still positive (BV/intermediate flora) at follow-up visit. | PMR: OR=0.35 (95% CI: 0.05-2.52)**[NS]**  [1/208 vs. 3/201 in intervention vs. control groups, respectively.] |
| 2. McDonald et al. (1997) [3] | Australia.  RCT. Pregnant women (N=480; N=242 intervention at 18 weeks’ gestation with BV or Gardnerella vaginalis. BV positive randomised to 242 anti-biotic vs. 238 placebo. | Compared the impact of metronidazole 400 mg x 2/day for 2 days at 24 wks’ gestation (intervention) vs. placebo (controls). | PMR: OR=0.98 (95% CI: 0.06-15.77)**[NS]**  [1/242 vs. 1/238 in intervention vs. control groups, respectively.] |
| 1. NICHD MFMU 2000.   Carey et al. [4] | USA.  RCT. Pregnant women (N=1919; N=953 intervention, N=966 controls), 16-23 wks gestation with asymptomatic BV (not TV+) for at least 6 weeks. | Compared the impact of 8 x 250 mg dose oral metronidazole plus repeat dose 48 hrs later @ 16-23 wks’ gestation with second treatment @ 24-30 wks’ gestation (intervention) vs. placebo (controls). | PMR: OR=0.69 (95% CI: 0.34- 1.39)**[NS]**  [13/952 vs. 19/965 in intervention vs. control groups, respectively.] |
| 1. Odendaal et al. 2002 [5] | South Africa (Tygerberg, West Cape). Tertiary academic hospital.  Cluster RCT. Women (N=155; N=128 intervention, N=127 controls) with BV; intention-to-treat analysis. | Compared the impact of oral metronidazole 400 mg 2x/daily for 2 days, repeated if still BV positive after 4 wks (intervention); vs. vitamin C placebo (controls). | PMR: OR=2.51 (95% CI: 0.75- 8.38)**[NS]**  [8/136 vs. 3/133 in intervention vs. control groups, respectively.] |
| 1. Ugwumadu et al. 2003 [6] | UK.  RCT. Pregnant women (N=285; N=244 intervention, N=241 controls), 12-22 wks’ gestation with asymptomatic intermediate flora (Nugent score 4-6) or BV (Nugent 7-10). | Compared the impact of oral clindamycin 300 mg twice daily for 5 days (intervention) vs. placebo (controls). | PMR: OR=0.99 (95% CI: 0.06- 15.84)**[NS]**  [1/244 vs. 1/241 in intervention vs. control groups, respectively.] |

References

1. McDonald HM, Brocklehurst P, Gordon A: **Antibiotics for treating bacterial vaginosis in pregnancy**. *Cochrane Database Syst Rev* 2007(1):CD000262.

2. Lamont RF, Duncan SL, Mandal D, Bassett P: **Intravaginal clindamycin to reduce preterm birth in women with abnormal genital tract flora**. *Obstet Gynecol* 2003, **101**(3):516-522.

3. McDonald HM, O'Loughlin JA, Vigneswaran R, Jolley PT, Harvey JA, Bof A, McDonald PJ: **Impact of metronidazole therapy on preterm birth in women with bacterial vaginosis flora (Gardnerella vaginalis): a randomised, placebo controlled trial**. *Br J Obstet Gynaecol* 1997, **104**(12):1391-1397.

4. Carey JC, Klebanoff MA, Hauth JC, Hillier SL, Thom EA, Ernest JM, Heine RP, Nugent RP, Fischer ML, Leveno KJ *et al*: **Metronidazole to prevent preterm delivery in pregnant women with asymptomatic bacterial vaginosis. National Institute of Child Health and Human Development Network of Maternal-Fetal Medicine Units**. *N Engl J Med* 2000, **342**(8):534-540.

5. Odendaal H, et al: **Preterm labour - is bacterial vaginosis involved?** *South African Medical Journal* 2002, **92**:231-234.

6. Ugwumadu A, Manyonda I, Reid F, Hay P: **Effect of early oral clindamycin on late miscarriage and preterm delivery in asymptomatic women with abnormal vaginal flora and bacterial vaginosis: a randomised controlled trial**. *Lancet* 2003, **361**(9362):983-988.
