# Supplementary material for: Reducing stillbirths: prevention and management of medical disorders and infections during pregnancy
Source: BMC Pregnancy Childbirth. 2009 May 7;9(Suppl 1):S4. doi: 10.1186/1471-2393-9-S1-S4 (PMC2679410; doi:10.1186/1471-2393-9-S1-S4)
Supplement: Additional file 20 — Web Table 20. Component studies in Lumbiganon et al. 2004 meta-analysis: impact of anti-biotics in high-risk pregnancy. Component studies in Lumbiganon et al. 2004 meta-analysis reporting impact on stillbirths/perinatal mortality [file 1471-2393-9-S1-S4-S20.doc]

**Web Table 20. Component studies in Lumbiganon et al. 2004 [1] meta-analysis: impact of anti-biotics in high-risk pregnancy**

| **Source** | **Location and Type of Study** | **Intervention** | **Stillbirths / Perinatal Outcomes** |
| --- | --- | --- | --- |
| 1. Rouse et al. (1997) [2] | USA.  RCT. Women (N=1024; N=508 in the chlorhexidine group and 516 in the placebo group). USA. | Assessed the impact of vaginal irrigation with 200 ml of 0.2% chlorhexidine (intervention), vs. sterile water placebo (controls). | PMR: RR=0.34 (95% CI: 0.01- 8.29)**[NS]**  [0/508 vs. 1/516 in intervention vs. control groups, respectively.] |
| 2. Rouse et al. (2003) [3] | RCT. 1041 patients  (525 in the chlorhexidine group and 516 in the placebo group). USA. | Assessed the impact of vaginal irrigation with 200 ml of 0.2% chlorhexidine every 6 hr during labour (intervention) vs. sterile water placebo (controls). | PMR: RR=1.98 (95% CI: 0.18- 21.78).  [2/526 vs. 1/521 in intervention vs. control groups, respectively.] |

References

1. Lumbiganon P, Thinkhamrop J, Thinkhamrop B, Tolosa JE: **Vaginal chlorhexidine during labour for preventing maternal and neonatal infections (excluding Group B Streptococcal and HIV)**. *Cochrane Database Syst Rev* 2004(4):CD004070.

2. Rouse DJ, Hauth JC, Andrews WW, Mills BB, Maher JE: **Chlorhexidine vaginal irrigation for the prevention of peripartal infection: a placebo-controlled randomized clinical trial**. *Am J Obstet Gynecol* 1997, **176**(3):617-622.

3. Rouse DJ, Cliver S, Lincoln TL, Andrews WW, Hauth JC: **Clinical trial of chlorhexidine vaginal irrigation to prevention peripartal infection in nulliparous women**. *American Journal of Obstetrics and Gynecology* 2003, **189**:166-170.
