# Supplementary material for: Reducing stillbirths: prevention and management of medical disorders and infections during pregnancy
Source: BMC Pregnancy Childbirth. 2009 May 7;9(Suppl 1):S4. doi: 10.1186/1471-2393-9-S1-S4 (PMC2679410; doi:10.1186/1471-2393-9-S1-S4)
Supplement: Additional file 21 — Web Table 21. Component studies in Goldenberg et al. 2006 meta-analysis: Impact of vaginally administered chlorhexidine during labour on stillbirths/perinatal mortality. Component studies in Goldenberg et al. 2006 meta-analysis reporting impact on stillbirths/perinatal mortality [file 1471-2393-9-S1-S4-S21.doc]

**Web Table 21. Component studies in Goldenberg et al. 2006 [1] meta-analysis: Impact of vaginally administered chlorhexidine during labour on stillbirths/perinatal mortality**

| **Source** | **Location and Type of Study** | **Intervention** | **Stillbirths / Perinatal Outcomes** |
| --- | --- | --- | --- |
| Bakr and Karkour 2005 [2] | Egypt.  Non-randomized, non-blinded trial. N = 4415 women. | Assessed the impact of chlorhexidine vaginal wash (intervention) vs. placebo (controls) in preventing maternal and neonatal infections and neonatal death. | Infant death: 2.8 vs. 4.22% in intervention vs. control groups, respectively (P=0.01).    Infant death due to infection: 0.22% vs. 0.84% in intervention vs. control groups, respectively (P=0.004) |
| Taha et al. 1997 [3] | Malawi (Africa). Hospital-based.  Non-randomized, non-blinded trial. N = 6,965 women. | Assessed the impact of chlorhexidine vaginal wash and a neonatal wipe soon after birth (intervention) vs. placebo (controls) in preventing maternal and neonatal infections and neonatal death. | ENND: RR=0.78 (95% CI: 0.60 – 1.00)  [29/1000 vs. 37/1000 in intervention vs. control groups, respectively].  NM due to sepsis: RR=0.33 (95% CI: 0.15 – 0.70)  [2.4 vs. 7.3 per 1000 in intervention vs. control groups, respectively]. |

References

1. Goldenberg RL, McClure EM, Saleem S, Rouse D, Vermund S: **Use of vaginally administered chlorhexidine during labor to improve pregnancy outcomes**. *Obstet Gynecol* 2006, **107**(5):1139-1146.

2. Bakr AF, Karkour T: **Effect of predelivery vaginal antisepsis on maternal and neonatal morbidity and mortality in Egypt**. *J Womens Health (Larchmt)* 2005, **14**(6):496-501.

3. Taha TE, Biggar RJ, Broadhead RL, Mtimavalye LA, Justesen AB, Liomba GN, Chiphangwi JD, Miotti PG: **Effect of cleansing the birth canal with antiseptic solution on maternal and newborn morbidity and mortality in Malawi: clinical trial**. *BMJ* 1997, **315**(7102):216-219; discussion 220.
