# Supplementary material for: Reducing stillbirths: prevention and management of medical disorders and infections during pregnancy
Source: BMC Pregnancy Childbirth. 2009 May 7;9(Suppl 1):S4. doi: 10.1186/1471-2393-9-S1-S4 (PMC2679410; doi:10.1186/1471-2393-9-S1-S4)
Supplement: Additional file 22 — Web Table 22. Component studies in Thinkhamrop et al. 2002 meta-analysis: impact of anti-biotics in high-risk pregnancy. Component studies in Thinkhamrop et al. 2002 meta-analysis reporting impact on stillbirths/perinatal mortality [file 1471-2393-9-S1-S4-S22.doc]

**Web Table 22. Component studies in Thinkhamrop et al. 2002 [1] meta-analysis: impact of anti-biotics in high-risk pregnancy**

| **Source** | **Location and Type of Study** | **Intervention** | **Stillbirths / Perinatal Outcomes** |
| --- | --- | --- | --- |
| 1. Gichangi et al. (1997) [2] | Kenya (Nairobi), Belgium (Ghent).  RCT. 1995-1996. High-risk pregnant women (N=253; N=134 intervention, N=119 controls), 28-32 wks gestation with history of LBW (< 2500 g), stillbirth or early perinatal death. | Compared the impact of a single dose of 2 gm cefetamet-pivoxil (intervention), vs. placebo (controls). | PMR: OR=0.53 (95% CI: 0.13- 2.16)**[NS]**  [3/134 vs. 5/119 in intervention vs. control groups, respectively.]  Mean birth weight: higher in intervention vs. control groups. |
| 2. McGregor et al. (1990) [3] | USA (Denver, Colorado; Seattle, Washington)  RCT. Pregnant women (N=235), 26-30 wks gestation. | Compared the impact of erythromycin 333 mg (intervention) vs. placebo (controls), one tablet three times a day for 7 days. | PMR: OR=0.12 (95% CI: 0.01-1.99)**[NS]**  [0/119 vs. 2/110 in intervention vs. control groups, respectively.] |
| 3. Vermeulen et al. (1999) [4] | The Netherlands.  RCT. 12 hospitals.  Pregnant women (N=168) 26-32 wks gestation with history of pre-term delivery in preceding pregnancy. | Compared the impact of clindamycin 2% vaginal cream (intervention), vs. placebo cream (controls), applied daily for 7 days. | PMR: OR=7.60 (95% CI: 0.15- 383.33)**[NS]**  [1/70 vs. 1/72 in intervention vs. control groups, respectively.] |

References

1. Thinkhamrop J, Hofmeyr GJ, Adetoro O, Lumbiganon P: **Prophylactic antibiotic administration in pregnancy to prevent infectious morbidity and mortality**. *Cochrane Database Syst Rev* 2002(4):CD002250.

2. Gichangi PB, Ndinya-Achola JO, Ombete J, Nagelkerke NJ, Temmerman M: **Antimicrobial prophylaxis in pregnancy: a randomized, placebo-controlled trial with cefetamet-pivoxil in pregnant women with a poor obstetric history**. *Am J Obstet Gynecol* 1997, **177**(3):680-684.

3. McGregor JA, French JI, Richter R, Vuchetich M, Bachus V, Seo K, Hillier S, Judson FN, McFee J, Schoonmaker J *et al*: **Cervicovaginal microflora and pregnancy outcome: results of a double-blind, placebo-controlled trial of erythromycin treatment**. *Am J Obstet Gynecol* 1990, **163**(5 Pt 1):1580-1591.

4. Vermeulen GM, Bruinse HW: **Prophylactic administration of clindamycin 2% vaginal cream to reduce the incidence of spontaneous preterm birth in women with an increased recurrence risk: a randomised placebo-controlled double-blind trial**. *Br J Obstet Gynaecol* 1999, **106**(7):652-657.
