# Supplementary material for: Reducing stillbirths: prevention and management of medical disorders and infections during pregnancy
Source: BMC Pregnancy Childbirth. 2009 May 7;9(Suppl 1):S4. doi: 10.1186/1471-2393-9-S1-S4 (PMC2679410; doi:10.1186/1471-2393-9-S1-S4)
Supplement: Additional file 23 — Web Table 23. Component studies in Kenyon et al. 2003 meta-analysis: Impact of antibiotics for PPROM/PROM. Component studies in Kenyon et al. 2003 meta-analysis reporting impact on stillbirths/perinatal mortality [file 1471-2393-9-S1-S4-S23.doc]

**Web Table 23. Component studies in Kenyon et al. 2003 [1] meta-analysis: Impact of antibiotics for PPROM/PROM**

| **Source** | **Location and Type of Study** | **Intervention/Study objectives** | **Stillbirths/Perinatal Outcomes** |
| --- | --- | --- | --- |
| 1. Almeida et al. 1996 [2] | Mozambique (where women were recruited), Sweden and Norway.  Double-blind, placebo-controlled RCT. N=110 women 30-36 wks gestation with clinically evident ROM, not in labour. | Compared the impact on PMR of amoxicillin 75 g 3 x daily (intervention) vs. placebo for 7 days or until delivery (controls). | PMR/Death before hospital discharge: RR=0.43 (95% CI: 0.16-1.18) **[NS]**  [5/59 vs. 10/51 in intervention vs. control groups, respectively]. |
| 2. Amon et al. 1988 [3] | USA.  RCT. N=82 women 20-34 wks gestation (N=43 intervention group, N=39 controls). | Assessed the impact on perinatal outcomes of intervention with ampicillin 1 gm IV every 6 hrs for 24 hrs. Maintained on oral 500 mg ampicillin 6 hourly until delivery. In labour, subject were recommenced on 1 gm intravenous ampicillin. | PMR/death before hospital discharge: RR=0.30 (95% CI: 0.06-1.41) **[NS]**  [2/43 vs. 6/39 in intervention vs. control groups, respectively]. |
| 3. Camli et al. 1997 [4] | Turkey.  RCT. N=31 women with PPROM between 28-34 wks gestation. PPROM confirmed by speculum. | Assessed the impact on PMR of oral ampicillin 1 gm 4 x daily (intervention). No placebo arm or tocolysis used (controls). | PMR/death before hospital discharge: RR=0.80 (95% CI: 0.21-3.00) **[NS]**  [3/15 vs. 4/16 in intervention vs. control groups, respectively]. |
| 4. Christmas et al. 1992 [5]  . | USA.  RCT. N=94 women with singleton pregnancies, 20 -34 wks with PPROM confirmed by sterile speculum (N=48 intervention group, N=46 controls). | Assessed the impact on PMR of 24 hrs IV ampicillin (2 g every 6 hrs for 4 doses); gentamycin 90 mg (loading dose 60 mg every 8 hrs for 3 doses); then oral amoxicillin + clavulanic acid (500 mg 3x/day for 7 days) (intervention) vs. IV fluids without antibiotics for 24 hrs (controls). | PMR/death before hospital discharge: RR=0.32 (95% CI: 0.03-2.96) **[NS]**  [1/48 vs. 3/46 in intervention vs. control groups, respectively]. |
| 5. Cox et al. 1995 [6] | USA (Dallas, TX).  RCT. N=62 women (pPROM) between 24 and 29 wks pregnant. | Compared the impact on PMR of co-amoxiclav 3 gm 6 hourly for 4 doses then co-amoxiclav 500 mg 6 hourly for 5 days (intervention) or matching placebo (controls). | PMR/Death before hosp italdischarge: RR=0.20 (95% CI: 0.02-1.61) **[NS]**  [1/31 vs. 5/31 in intervention vs. control groups, respectively]. |
| 6. Garcia-Burguillo et al. 1995 [7] | Spain.  Double-blind, placebo-controlled RCT. N=60 singleton pregnancy women under 36 wks gestation with pre-term PROM. Ruptured membranes confirmed by sterile speculum examination, ferning test and nitrazine test. No steroids or tocolytics given post-randomisation. | Compared the impact on PMR of erythromycin 500 mg 6 hourly orally until delivery (intervention) vs. matched controls given placebo until delivery (controls). | PMR/Death before hospital discharge: RR=0.40 (95% CI: 0.08-1.90) **[NS]**  [2/30 vs. 5/30 in intervention vs. control groups, respectively].  RR= |
| 7. Grable IA, et al. (1996) [8] | USA.  Double-blind, placebo-controlled RCT. N=60 women. | Assessed the effect on PMR of treatment with IV ampicillin (2 gm every 6 hrs for 24 hrs followed by 500 mg oral ampicillin) until delivery or discharge (intervention) vs. matched controls given placebo (controls). | PMR/Death before hospital discharge: RR=0.19 (95% CI: 0.01-3.75) **[NS]**  [0/31 vs. 2/29 in intervention vs. control groups, respectively]. |
| 8. Johnston MM, et al. 1990 [9] | USA (Florida).  Double-blind, placebo-controlled RCT. N=85 mothers with singleton gestations between 20-34 wks with PPROM confirmed by sterile speculum for pooling, ferning and nitrazine paper testing. | Compared the impact on PMR of IV mezlocillin for 48 hours followed by oral ampicillin until delivery (intervention) or matched controls given IV + oral placebo (controls). | PMR/Death before hospital discharge: RR=0.84 (95% CI: 0.20-3.54) **[NS]**  [3/40 vs. 4/45 in intervention vs. control groups, respectively]. |
| 9. Kenyon SL et al. 2001 [10] | UK + other countries. Multicentre.  Double-blind, placebo-controlled RCT. N=4826 women < 37 wks pregnant with PPROM. Multiple pregnancies included. | Compared the impact on PMR of co-amoxiclav 375 mg QDS, erythromycin 250 mg QDS orally for 10 days or until delivery (intervention) vs. matched placebo (controls); (2 x 2 factorial design). | PMR/Death before hospital discharge: RR=0.94 (95% CI: 0.74-1.20) **[NS]**  [226/3584 vs. 82/1225 in intervention vs. control groups, respectively]. |
| 10. Kurki T, et al. 1992 [11] | Finland. Hospital-based.  Double-blind, placebo-controlled RCT. N=101 women between 23-36 wks pregnant with visible leakage of amniotic fluid who did not go into labour within 12 hrs of admission. | Compared the impact on PMR of 2 doses of IV penicillin (5 mu; intervention) vs. matched placebo (controls). | PMR/Death before hospital discharge: RR=1.02 (95% CI: 0.07-15.88) **[NS]**  [1/57 vs. 1/58 in intervention vs. control groups, respectively]. |
| 11. Lockwood CJ, et al. 1993 [12] | USA.  Double-blind, placebo-controlled RCT. N=75 women with a single fetus at 24-34 wks gestation with PROM. No digital examination unless active labour. Women had infection screening. | Compared the impact on PMR of iperacillin 3 gm IV 6 hourly 72 hours (intervention) vs. placebo (controls). | PMR/Death before hospital discharge: RR=0.95 (95% CI: 0.20-4.38) **[NS]**  [3/37 vs. 3/35 in intervention vs. control groups, respectively]. |
| 12. Magwali TL, et al. 1999 [13] | Zimbabwe.  RCT. N=171 women (N=84 intervention group, N=87 controls). | Assessed the effect on PMR of co-amoxiclav for 5 days. No mention of daily frequency or mg of drugs. | PMR/Death before hospital discharge: RR=0.76 (95% CI: 0.32-1.80) **[NS]**  [8/82 vs. 11/86 in intervention vs. control groups, respectively]. |
| 13. McGregor JA, et al. 1991 [14] | USA (Denver). University hospital.  Double-blind, placebo-controlled RCT. N=65 women with singleton pregnancies between 23-34 wks' gestation with PPROM. Sterile speculum. No corticosteroids administered. (N=55 analyzed: N=28 intervention group, N=27 controls). | Compared the impact on PMR of erythromycin 333 mg 3 x daily (intervention) vs. placebo (controls) 7 days or until active labour started. | PMR/Death before hospital discharge: RR=12.55 (95% CI: 0.74-212.52) **[NS]**  [6/28 vs. 0/27 in intervention vs. control groups, respectively].  RR= |
| 14. Mercer BM, et al. 1992 [15] | USA.  Double-blind, placebo-controlled RCT. N=220 women 20-34/6 wks pregnant with pPROM - sterile speculum and evaluation of cervix. Amniocentesis done for infection screen. Multiple pregnancies included. | Compared the impact on PMR of oral 333 mg erythromycin 8 hourly from randomisation to delivery (intervention) vs. matched placebo (controls). | PMR/Death before hospital discharge: RR=0.65 (95% CI: 0.24-1.71) **[NS]**  [6/106 vs. 10/114 in intervention vs. control groups, respectively]. |
| 15. Mercer B, et al. 1997 [16-19] | USA.  Double-blind, placebo-controlled RCT. N=614 women with PPROM, 24-32 wks' gestation. | Compared the impact on PMR of ampicillin 2 g 6 hourly and erythromycin 250 mg 6 hourly IV for 48 hours, then oral amoxacillin 250 mg every 8 hours and erythromycin 333 mg 8 hourly for 5 days (intervention) vs. a matching placebo regimen (controls). | PMR/Death before hospital discharge: RR=1.10 (95% CI: 0.59-2.06) **[NS]**  [19/299 vs. 18/312 in intervention vs. control groups, respectively]. |
| 16. Morales WJ, et al. 1989 [20] | USA.  RCT. N=165 women (N=41 group 1; N=43 group 2; N=37 group 3; N=44 group 4) with singleton pregnancies <34 wks gestation with PPROM. | Compared the impact on PMR of intervention with 2 g IV ampicillin every 6 hrs until results of cervical cultures negative. Group 1 given no treatment (controls), group 2 given steroids only, group 3 given antibiotic only, group 4 given steroids + antibiotic. | PMR/Death before hospital discharge: RR=1.47 (95% CI: 0.38-5.73) **[NS]**  [5/42 vs. 3/37 in intervention vs. control groups, respectively]. |
| 17. Ovalle-Salas A, et al. 1997 [21] | Chile and USA.  Double-blind, placebo-controlled RCT. N=88 women. | Compared the impact on PMR of intervention with clindamycin 600 mg IV every 6 hours for 48 hours + 4 mg/kg/day gentamycin IV for 48 hours followed by clindamycin 300 mg orally every 6 hours for 5 days + gentamycin 2 mg/kg/day IM every 12 hrs for 5 days (intervention) vs. matching placebo (controls). | PMR/Death before hospital discharge: RR=1.19 (95% CI: 0.44-3.26) **[NS]**  [7/42 vs. 6/43 in intervention vs. control groups, respectively]. |
| 18. Owen J, et al. 1993 [22] | USA.  RCT, not placebo-controlled. N=118 women (N=59 intervention group, N=58 controls). | Assessed the effect on PMR of IV 1 gm ampicillin 6 hourly for 24 hours then 500 mg ampicillin orally every 6 hours. If allergic to penicillin 500 mg erythromycin used 6 hourly. Treatment continued with delivery or diagnosis of chorioamnionitis. | PMR/Death before hospital discharge: RR=0.56 (95% CI: 0.17-1.82) **[NS]**  [4/59 vs. 7/58 in intervention vs. control groups, respectively]. |
| 19. Svare J. 1997 [23] | Denmark (Copenhagen).  Double-blind placebo-controlled RCT. N=67 women with singleton pregnancies 26-34 wks gestation with ROM & leakage of amniotic fluid at vaginal speculum examination (preceding onset of uterine contractions). | Compared the impact on PMR of intervention with ampicillin 2 gm IV 6 hourly. 24 hrs - pivampicillin 500 g orally 8 hourly for 7 days plus IV metronidazole 500 mg every 8 hours for 24 hrs, followed by metronidazole 400 mg orally every 8 hrs for 7 days (intervention) vs. identical placebo (controls). | PMR/Death before hospital discharge: RR=1.23 (95% CI: 0.18-8.25) **[NS]**  [2/30 vs. 2/37 in intervention vs. control groups, respectively]. |

References

1. Kenyon S, Boulvain M, Neilson J: **Antibiotics for preterm rupture of membranes**. *Cochrane Database Syst Rev* 2003(2):CD001058.

2. Almeida L, Schmauch A, Bergstrom S: **A randomised study on the impact of peroral amoxicillin in women with prelabour rupture of membranes preterm**. *Gynecol Obstet Invest* 1996, **41**(2):82-84.

3. Amon E, Lewis SV, Sibai BM, Villar MA, Arheart KL: **Ampicillin prophylaxis in preterm premature rupture of the membranes: a prospective randomized study**. *Am J Obstet Gynecol* 1988, **159**(3):539-543.

4. Camli L, Mavunagacioglu S, Bostanci A, Camli S, Soylu F: **Antibiotherapy in preterm premature rupture of membrane. Does it affect the latent period and infectious morbidity?** *Jinekoloji Ve Obstetrik Dergisi* 1997, **11**:138-142.

5. Christmas JT, Cox SM, Andrews W, Dax J, Leveno KJ, Gilstrap LC: **Expectant management of preterm ruptured membranes: effects of antimicrobial therapy**. *Obstet Gynecol* 1992, **80**(5):759-762.

6. Cox SM, Leveno KJ, Sherman ML, Travis L, De Plama R **Ruptured membranes at 24 to 29 weeks: a randomized double blind trial of antimicrobials versus placebo**. *American Journal of Obstetrics and Gynecology* 1995, **172**:412.

7. Garcia-Burguillo A, Hernandez-Garcia JM, P dlF: **Erythromycin prophylaxis in preterm pregnancies with rupture of amniotic membranes [Profilaxis con eritromicina en gestaciones pretermino con rotura prematura de las membranas amnioticas]**. *Clinica e Investigacion en Ginecologia y Obstetricia* 1995, **23**:96-100.

8. Grable IA, Garcia PM, Perry D, Socol ML: **Group B Streptococcus and preterm premature rupture of membranes: a randomized, double-blind clinical trial of antepartum ampicillin**. *Am J Obstet Gynecol* 1996, **175**(4 Pt 1):1036-1042.

9. Johnston MM, Sanchez-Ramos L, Vaughn AJ, Todd MW, Benrubi GI: **Antibiotic therapy in preterm premature rupture of membranes: a randomized, prospective, double-blind trial**. *Am J Obstet Gynecol* 1990, **163**(3):743-747.

10. Kenyon SL, Taylor DJ, Tarnow-Mordi W: **Broad-spectrum antibiotics for preterm, prelabour rupture of fetal membranes: the ORACLE I randomised trial. ORACLE Collaborative Group**. *Lancet* 2001, **357**(9261):979-988.

11. Kurki T, Hallman M, Zilliacus R, Teramo K, Ylikorkala O: **Premature rupture of the membranes: effect of penicillin prophylaxis and long-term outcome of the children**. *Am J Perinatol* 1992, **9**(1):11-16.

12. Lockwood CJ, Costigan K, Ghidini A, Wein R, Chien D, Brown BL, Alvarez M, Cetrulo CL: **Double-blind; placebo-controlled trial of piperacillin prophylaxis in preterm membrane rupture**. *Am J Obstet Gynecol* 1993, **169**(4):970-976.

13. Magwali TL, Cipato T, Majoko F, Rusakaniko S, Mujaji C: **Prophylactic augmentin in prelabour preterm rupture of the membranes**. *International Journal of Gynecology & Obstetrics* 1999, **65**:261-265.

14. McGregor JA, French JI, Seo K: **Antimicrobial therapy in preterm premature rupture of membranes: results of a prospective, double-blind, placebo-controlled trial of erythromycin**. *Am J Obstet Gynecol* 1991, **165**(3):632-640.

15. Mercer BM, Moretti ML, Prevost RR, Sibai BM: **Erythromycin therapy in preterm premature rupture of the membranes: a prospective, randomized trial of 220 patients**. *Am J Obstet Gynecol* 1992, **166**(3):794-802.

16. Mercer B, Miodovnik M, Thurnau G, Goldenberg R, Das A, Merenstein G, al e: **A multicentre randomized controlled trial of antibiotic therapy versus placebo therapy after preterm premature rupture of the membranes**. *American Journal of Obstetrics and Gynecology* 1996, **174**:304.

17. Mercer B: **The NICHD-MFMU antibiotic treatment of PPROM study: evaluation of factors associated with successful outcome**. *American Journal of Obstetrics and Gynecology* 1997, **176**(1 Pt 2):S8.

18. Mercer BM, Miodovnik M, Thurnau GR, Goldenberg RL, Das AF, Ramsey RD, Rabello YA, Meis PJ, Moawad AH, Iams JD *et al*: **Antibiotic therapy for reduction of infant morbidity after preterm premature rupture of the membranes. A randomized controlled trial. National Institute of Child Health and Human Development Maternal-Fetal Medicine Units Network**. *JAMA* 1997, **278**(12):989-995.

19. Ramsey P: **Preterm premature rupture of membranes (PPROM): latency and neonatal outcome**. *American Journal of Obstetrics and Gynecology* 2002, **187**(6 Pt 2 ):S113.

20. Morales WJ, Angel JL, O'Brien WF, Knuppel RA: **Use of ampicillin and corticosteroids in premature rupture of membranes: a randomized study**. *Obstetrics & Gynecology* 1989, **73**:721-726.

21. Ovalle-Salas A, Gomez R, Martinez MA, Rubio R, Fuentes A, Valderrama O, et al: **Antibiotic therapy in patients with preterm premature rupture of membranes: a prospective, randomized, placebo-controlled study with microbiological assessment of the amniotic cavity and lower gential tract.** . *Prenatal and Neonatal Medicine* 1997, **2**:213-222.

22. Owen J, Groome LJ, Hauth JC: **Randomised trial of prophylactic therapy after preterm amnion rupture.** . *American Journal of Obstetrics and Gynecology* 1993, **169**:976-981.

23. Svare J: **Preterm delivery and subclinical uro-genital infection [thesis]**. Denmark: Department of Obstetrics and Gynaecology Rigshospitalet

University of Copenhagen; 1997.
