# Supplementary material for: Reducing stillbirths: prevention and management of medical disorders and infections during pregnancy
Source: BMC Pregnancy Childbirth. 2009 May 7;9(Suppl 1):S4. doi: 10.1186/1471-2393-9-S1-S4 (PMC2679410; doi:10.1186/1471-2393-9-S1-S4)
Supplement: Additional file 24 — Web Table 24. Component studies in Flenady and King 2002 meta-analysis: Impact of anti-biotics for PROM at or near term. Component studies in Flenady and King 2002 meta-analysis reporting impact on stillbirths/perinatal mortality [file 1471-2393-9-S1-S4-S24.doc]

**Web Table 24. Component studies in Flenady and King 2002 [1] meta-analysis: Impact of anti-biotics for PROM at or near term**

| **Source** | **Location and Type of Study** | **Intervention/Study objectives** | **Stillbirths/Perinatal Outcomes** |
| --- | --- | --- | --- |
| 1. Cararach et al. (1998) [2] | Spain. Multicentre, hospital-based.  RCT. N=733 women with gestational age ≥ 36 wks, membranes ruptured < 12 hrs, and without uterine contractions. | Compared the impact on PMR of anti-biotics (IV ampicillin 1g every 6 hrs + IM gentamicin 80mgs every 8 hrs or IM erythromycin 500mg every 6 hrs for women with penicillin allergy) on admission following vaginal and endocervical culture, versus no treatment (controls). | PMR: RR=0.98 (95% CI: 0.14-6.89) **[NS]**  [2/371 vs. 2/362 in intervention vs. control groups, respectively]. |
| 2. Ovalle et al. (1998) [3]  . | Chile (Santiago). Hospital.  RCT. N=105 women at 37-42 wks gestation, singleton pregnancy, duration of MR less than 12 hrs, no labour. | Compared the impact on PMR of administering antibiotics (IV clindamycin 600 mg every 6 hrs and IV cefuroxime 750mgs every 8 hrs for 48 hrs then oral cefuroxime 250mgs every 12 hrs and clindamycin 300mgs every 6 hrs for a further 24 hrs.) on admission following cervicovaginal and amniotic fluid culture (intervention), vs. placebo following admission cultures (controls). | PMR: RR=not estimable **[NS]**  [0/55 vs. 0/50 in intervention vs. control groups, respectively]. |

References

1. Flenady V, King J: **Antibiotics for prelabour rupture of membranes at or near term**. *Cochrane Database of Systematic Reviews* 2002, **3**:CD001807.

2. Cararach V, Botet F, Sentis J, Almirall R, Perez-Picanol E: **Administration of antibiotics to patients with rupture of membranes at term: a prospective, randomized, multicentric study. Collaborative Group on PROM**. *Acta Obstetricia et Gynecologica Scandinavica* 1998, **77**:298-302.

3. Ovalle A, Gomez R, Martinez MA, Giglio MS, Bianchi R, Diaz J, et al: **Antibiotic treatment of patients with term premature rupture of membranes: a randomized clinical trial**. *Prenatal and Neonatal Medicine* 1998, **3**:599-606.
