# Supplementary material for: Reducing stillbirths: prevention and management of medical disorders and infections during pregnancy
Source: BMC Pregnancy Childbirth. 2009 May 7;9(Suppl 1):S4. doi: 10.1186/1471-2393-9-S1-S4 (PMC2679410; doi:10.1186/1471-2393-9-S1-S4)
Supplement: Additional file 25 — Web Table 25. Component studies in Garner and Gulmezoglu 2006 meta-analysis: impact of anti-malarials in malaria-endemic areas. Component studies in Garner and Gulmezoglu 2006 meta-analysis reporting impact on stillbirths/perinatal mortality [file 1471-2393-9-S1-S4-S25.doc]

**Web Table 25. Component studies in Garner and Gulmezoglu 2006 [1] meta-analysis: impact of anti-malarials in malaria-endemic areas**

| **Source** | **Location and Type of Study** | **Intervention** | **Stillbirths / Perinatal Outcomes** |
| --- | --- | --- | --- |
| 1. Cot et al. 1992 [2] | Burkina Faso  Quasi-RCT. Pregnant women (N=1464). | Compared a group given weekly dosages of chloroquine (intervention) vs. unmedicated group (controls). | SBR: RR=1.11 (95% CI: 0.50-2.45)**[NS]**  [13/612 vs. 11/573 in intervention vs. control groups, respectively.]  PMR: RR=1.10 (95% CI: 0.50-2.44) **[NS]**  [13/594 vs. 11/554 in intervention vs. control groups, respectively.] |
| 2. Cot et al. 1995 [3] | Cameroon  Quasi-RCT. Pregnant primigravidae (N=266) attending ANC. | Compared a group given weekly 300 mg dose of chloroquine (intervention) vs. unmedicated group (controls). | SBR: RR=0.56 (95% CI: 0.11-2.93)**[NS]**  [2/63 vs. 4/70 in intervention vs. control groups, respectively.] |
| 3. Greenwood et al. 1989 [4] | The Gambia  Cluster-RCT. All women (N=1049) who became pregnant in study villages (some sub-studies only followed up primigravidae) | Compared a group given weekly dose of pyrimethamine and dapsone (intervention) vs. placebo (controls). | SBR: *Among women in 1^st^ or 2^nd^ pregnancy:* RR=0.54 (95% CI: 0.27-1.05)**[NS]**  [12/193 vs. 22/190 in intervention vs. control groups, respectively.]  PMR: RR=0.77 (95% CI: 0.48-1.24)  [28/501 vs. 37/511 in intervention vs. control groups, respectively.]  *Among women in 1^st^ or 2^nd^ pregnancy:*  RR=0.67 (95% CI: 0.41-1.09)**[NS]**  **[**23/193 vs. 34/190 in intervention vs. control groups, respectively.] |
| 4. Morley et al. 1964 [5]  . | Nigeria  Quasi-RCT. Women (N=429) registered at dispensary for ANC | Compared a group given monthly pyrimethamine (intervention) vs. placebo (controls). | PMR: RR=1.07 (95% CI: 0.52-2.22)**[NS]**  [14/210 vs. 13/209 in intervention vs. control groups, respectively.] |
| 5. Ndyomugyenyi et al. 2000 [6] | Uganda  RCT. Primigravidae (N=860). | Compared a group given chloroquine only (intervention #1), a group given chloroquine plus iron-folate (intervention #2), and a group given iron-folate only (controls). | SBR: RR=1.61 (95% CI: 0.60-4.35) **[NS]**  [10/186 vs. 6/180 in intervention vs. control groups, respectively.]  PMR: RR=0.48 (95% CI: 0.04-5.29) **[NS]**  [1/186 vs. 2/180 in intervention vs. control groups, respectively.] |
| 6. Nosten et al. 1994 [7] | Thailand.  RCT. Women (N=339) > 20 wks’ gestation attending ANC. | Compared a group given weekly mefloquine (intervention) vs. no treatment (controls). | SBR: RR=2.61 (95% CI: 0.85-8.02)**[NS]**  [11/158 vs. 4/150 in intervention vs. control groups, respectively.]  PMR: RR=3.51 (95% CI: 1.00-12.32)**[NS]**  [11/159 vs. 3/152 in intervention vs. control groups, respectively.] |
| 7. Parise et al. 1998i [8] | Kenya.  Quasi-RCT. Women (N=2077) in first or second pregnancy attending ANC. | Compared impact of SP treatment dose at study entry, repeated in late pregnancy (intervention) vs. SP given only for recent history of fever or parasitaemia [*not* IPT](controls). | SBR: RR=1.20 (95% CI: 0.42-3.42)**[NS]**  [11/432 vs. 5/236 in intervention vs. control groups, respectively.] |
| 8. Parise et al. 1998ii [8] | Kenya.  Quasi-RCT. Women (N=2077) in first or second pregnancy attending ANC. | Compared monthly sulfadoxine-pyrimethamine (intervention) vs. SP given only for recent history of fever or parasitaemia [*not* IPT](controls). | SBR: RR=0.99 (95% CI: 0.33-2.91)**[NS]**  [9/431 vs. 5/236 in intervention vs. control groups, respectively.] |
| 9. Shulman 1999 [9] | Kenya (Kilifi). Rural setting.  RCT. Primigravidae (N=1264) attending antenatal clinics at a health centre (1) or hospital (1); singleton pregnancy; 16-30 wks’ gestation | Compared intermittent SP at recruitment at 16-19 wks (2 doses); 20-26 wks (2 doses); 27-30 wks (1 dose) to prevent severe anaemia secondary to malaria in pregnancy (intervention), vs. placebo (controls). Ferrous sulphate and impregnated bed nets were also in use in the area. | SBR: RR=0.90 (95% CI: 0.52-1.55)**[NS]**  [24/626 vs. 26/611 in intervention vs. control groups, respectively.]  PMR: RR=0.78 (0.52-1.17)**[NS]**  [39/626 vs. 49/611 in intervention (prevention) vs. control groups, respectively.] |

References

1. Garner P, Gulmezoglu AM: **Drugs for preventing malaria in pregnant women**. *Cochrane Database Syst Rev* 2006(4):CD000169.

2. Cot M, Roisin A, Barro D, Yada A, Verhave JP, Carnevale P, Breart G: **Effect of chloroquine chemoprophylaxis during pregnancy on birth weight: results of a randomized trial**. *Am J Trop Med Hyg* 1992, **46**(1):21-27.

3. Cot M, Le Hesran JY, Miailhes P, Esveld M, Etya'ale D, Breart G: **Increase of birth weight following chloroquine chemoprophylaxis during the first pregnancy: results of a randomized trial in Cameroon**. *Am J Trop Med Hyg* 1995, **53**(6):581-585.

4. Greenwood BM, Greenwood AM, Snow RW, Byass P, Bennett S, Hatib-N'Jie AB: **The effects of malaria chemoprophylaxis given by TBAs on the course and outcome of pregnancy**. *Transactions of the Royal Society of Tropical Medicine and Hygiene* 1989, **83**:589-594.

5. Morley D, Woodland M, Cuthbertson WF: **Controlled Trial of Pyrimethamine in Pregnant Women in an African Village**. *Br Med J* 1964, **1**(5384):667-668.

6. Ndyomugyenyi R, Magnussen P: **Chloroquine prophylaxis, iron-folic acid supplementation or case management of malaria attacks in primigravidae in western Uganda: effects on maternal parasitaemia and haemoglobin levels and on birthweight**. *Trans R Soc Trop Med Hyg* 2000, **94**(4):413-418.

7. Nosten F, ter Kuile F, Maelankiri L, Chongsuphajaisiddhi T, Nopdonrattakoon L, Tangkitchot S, et al: **Mefloquine prophylaxis in pregnancy: a double blind placebo controlled trial**. *Journal of Infectious Diseases* 1994, **169**:595-603.

8. Parise ME, Ayisi JG, Nahlen BL, Schultz LJ, Roberts JM, Misore A, Muga R, Oloo AJ, Steketee RW: **Efficacy of sulfadoxine-pyrimethamine for prevention of placental malaria in an area of Kenya with a high prevalence of malaria and human immunodeficiency virus infection**. *Am J Trop Med Hyg* 1998, **59**(5):813-822.

9. Shulman CE, Dorman EK, Cutts F, Kawuondo K, Bulmer JN, Peshu N, et al: **Intermittent sulpadoxine-pyrimethamine to prevent severe anaemia secondary to malaria in pregnancy: a randomised placebo-controlled trial**. *Lancet* 1999, **353**:632-636.
