# Supplementary material for: Reducing stillbirths: prevention and management of medical disorders and infections during pregnancy
Source: BMC Pregnancy Childbirth. 2009 May 7;9(Suppl 1):S4. doi: 10.1186/1471-2393-9-S1-S4 (PMC2679410; doi:10.1186/1471-2393-9-S1-S4)
Supplement: Additional file 26 — Web Table 26. Component studies in Gamble et al. 2006 & 2007 meta-analyses: impact of ITNs. Component studies in Gamble et al. 2006, 2007 meta-analysis reporting impact on stillbirths/perinatal mortality [file 1471-2393-9-S1-S4-S26.doc]

**Web Table 26. Component studies in Gamble et al. 2006 & 2007 [1, 2] meta-analyses: impact of ITNs**

| **Source** | **Location and Type of Study** | **Intervention** | **Stillbirths / Perinatal Outcomes** |
| --- | --- | --- | --- |
| 1. Njagi et al, 2002 [3] | Kenya.  RCT. Women (N=963) in first or second week of pregnancy were recruited, N=752 followed up. | Assessed the impact of polyester ITNs plus SP (intervention #1), ITNs only (intervention #2), SP only (intervention #3), or no nets/placebo (intervention #4). | *For 1^st^ or 2^nd^ pregnancy:*  Fetal death (miscarriage+SB): ITN+SP (8/205), SP (11/191), ITN (12/206), no net (17/188).  RR=0.68 (95% CI: 0.28-1.65)  ITNs (+/- SP) vs. no net (+/- SP):  RR=0.64 (95% CI: 0.32-1.29) |
| 2. Shulman et al. (1998) [4] | Kenya. Community based.  RCT. Women (N=462) in a population already randomised to receive or not receive ITNs. | Compared the impact of permethrin-treated [500g/m^2^] ITNs (intervention) vs. no nets (controls). All women with haemoglobin <10 g/dL were given hematins, women with parasitemia were treated with SP; women with severe anaemia also given SP to prevent placental parasitaemia. Nets provided for all members of intervention households. | SBR: adj. RR=0.67 (95% CI: 0.19–2.32, P=0.51)**[NS]**  [45/1000 vs. 70/1000 in intervention vs. control groups, respectively.]  PMR: 75/1000 vs. 80/1000 in intervention vs. control groups, respectively. No statistical significance data. |
| 3. ter Kuile 2003 [5] | Kenya. Rural, community-based.  RCT. Village-randomised. 1997-1998. Women (N=780; N=381 intervention, N=399 controls). | Compared the impact of permethrin-treated ITNs (intervention) vs. no nets (controls). | Fetal death (miscarriage+SB):  *For 1^st^-4th pregnancy:*  RR=0.69 (95% CI: 0.41-1.16)**[NS]**  *For 5^th^ or higher-order pregnancy:*  RR=1.02 (95% CI: 0.17- 6.23)**[NS]**  Protective efficacy=31% (95% CI:-16–59%, P=0.17)**[NS]** *in 1^st^-4^th^ pregnancy.*  [2.3% vs. 3.3% in intervention vs. control groups, respectively.]  *In 5^th^ or higher-order pregnancy*: 2.2% vs. 2.1% in intervention vs. control groups, respectively.**[NS]** |
| 4. Dolan et al, 1993 [6] | Thailand (Thai-Burmese border).  RCT. 3 adjacent study sites. Pregnant women (N=307; N=103 intervention #1, N=100 intervention #2, N=104 controls) of Karen ethnic group. | Compared the impact of permethrin-treated [500g/m^2^] nylon bed nets (intervention #1), untreated bed nets (intervention #2) or no study nets (controls; *NB—*some control families already possessed untreated nets from NGOs). Women who developed malaria treated with quinine or chloroquine depending on strain; severe cases of *P. falciparum* treated with IV quinine in a local hospital. | Fetal death (miscarriage+SB): RR=0.21 (95% CI: 0.05-0.92)  [2/102 (2%) vs. 10/97 (10%) in intervention #1 group vs. controls, respectively.] |

References

1. Gamble C: **Insecticide-treated nets for the prevention of malaria in pregnancy: a systematic review of randomized controlled trials**. *PLoS Med* 2007 Mar 27, **4**:e107.

2. Gamble C, Ekwaru JP, ter Kuile FO: **Insecticide-treated nets for preventing malaria in pregnancy**. *Cochrane Database Syst Rev* 2006(2):CD003755.

3. Njagi J: **The effects of sulfadoxine-pyrimethamine intermittent treatment and pyrethroid impregnated bed nets on malaria morbidity and birth weight in Bondo district, Kenya [dissertation]**. Nairobi: University of Nairobi; Copenhagen .Danish Bilharziasis Laboratory .129p

2002.

4. Shulman CE DE, Talisuna AO, Lowe BS, Nevill C, Snow RW, et al: **A community randomized controlled trial of insecticide-treated bednets for the prevention of malaria and anaemia among primigravid women on the Kenyan coast**. *Tropical Medicine and International Health* 1998, **3**:197-204.

5. ter Kuile FO, Terlouw DJ, Phillips-Howard PA, Hawley WA, Friedman JF, Kariuki SK, Shi YP, Kolczak MS, Lal AA, Vulule JM *et al*: **Reduction of malaria during pregnancy by permethrin-treated bed nets in an area of intense perennial malaria transmission in western Kenya**. *Am J Trop Med Hyg* 2003, **68**(4 Suppl):50-60.

6. Dolan G, ter Kuile FO, Jacoutot V, White NJ, Luxemburger C, Malankirii L, Chongsuphajaisiddhi T, Nosten F: **Bed nets for the prevention of malaria and anaemia in pregnancy**. *Trans R Soc Trop Med Hyg* 1993, **87**(6):620-626.
