# Supplementary material for: Reducing stillbirths: prevention and management of medical disorders and infections during pregnancy
Source: BMC Pregnancy Childbirth. 2009 May 7;9(Suppl 1):S4. doi: 10.1186/1471-2393-9-S1-S4 (PMC2679410; doi:10.1186/1471-2393-9-S1-S4)
Supplement: Additional file 27 — Web Table 27. Component studies in Volmink et al. 2007 meta-analysis: impact of PMTCT. Component studies in Volmink et al. 2007 meta-analysis reporting impact on stillbirths/perinatal mortality [file 1471-2393-9-S1-S4-S27.doc]

**Web Table 27. Component studies in Volmink et al. 2007** **[1] meta-analysis: impact of PMTCT**

| **Source** | **Location and Type of Study** | **Intervention** | **Stillbirths / Perinatal Outcomes** |
| --- | --- | --- | --- |
| 1. Bhoopat et al. 2005 [2] | Thailand.  RCT. Women (N=50) recruited from 2 hospitals. | Assessed impact of long-term maternal treatment with zidovudine 300mg BD 62-92 days before labour (median 76 days), then 300mg at labour onset + every 3 hr until delivery (intervention) vs. short-term zidovudine (same dose and administration as long-term, but initiated 14-35 days before labour)(controls). | SBR: 0/23 vs. 0/27 in intervention vs. control groups, respectively. |
| 2. DITRAME : Dabis et al. 1999 [3] | Côte d’Ivoire (Abidjan), Burkina Faso (Bobo-Dioulasso). Public clinics.  RCT.  . | Assessed impact of maternal treatment with zidovudine 300mg twice daily from 36-38 wks’ gestation until onset of labour, then 600 mg at start of labour and 300mg twice daily until 7 days after birth (intervention), vs. no treatment (controls). | SBR: RR=0.14 (95% CI: 0.02-1.17)**[NS]**  [1/214 vs. 7/217 in intervention vs. control groups, respectively.] |
| 3. HIVNET 012: Guay et al. 1999 [4] | Uganda (Kampala). Hospital setting.  RCT. Women (N=645) at ANC clinics from 1997-1999. | Compared effectiveness of maternal treatment with nevirapine 200mg oral dose at onset of labour plus infant treatment with oral dose nevirapine 2mg/kg 72 hr after birth or at hospital discharge, whichever was soonest (intervention #1) vs. zidovudine 600mg orally at onset of labour and 300mg every 3 hr during labour plus infant treatment with oral dose zidovudine syrup 4mg/kg twice daily for 7 days after birth (intervention #2) vs. placebo (controls). Placebo (N=19) discontinued after trial proved zidovudine effective. | SBR: RR=2.00 (95% CI: 0.18, 21.94)**[NS]**  [2/313 vs. 1/313 in intervention #1 vs. intervention #2 groups respectively.] |
| 4. Kiarie et al. 2003 [5] | Kenya (Nairobi).  Cluster (block) RCT. Women (N=188) recruited from ANC at a tertiary hospital. | Compared 'THAI CDC' arm: zidovudine (intervention) to 'HIVNET 012' – nevirapine tablet + syrup (controls). | SBR: RR=1.48 (95% CI: 0.25-8.58)**[NS]**  **[**3/70 vs. 2/69 in intervention vs. control groups respectively.] |
| 5. Limpongsanurak et al. 2001 [6] | Thailand (Bangkok).  Multicenter RCT. | Assessed impact of maternal treatment with zidovudine 250 mg orally twice daily from 38 weeks gestation until onset of labour then IV zidovudine 2mg/kg for 1st hour of labour followed by 1mg/kg/hr until delivery (intervention), vs. placebo (oral capsules, then 5% IV dextrose in saline; controls). | SBR: RR=3.07 (95% CI: 0.13-74.28)**[NS]**  [1/90 vs. 0/92 in intervention vs. control groups, respectively.] |
| 6. PACTG 076 : Connor et al. 1994 [7] | USA, France.  RCT. 59 centers. Women (N=477) recruited from 1991-1993. | Assessed impact of maternal treatment of zidovudine 100mg orally 5x/day from time of presentation (14-34 wks) until labour onset, then IV zidovudine 2mg/kg loading dose over one hour followed by 1 mg/kg/hr until delivery, plus infant treatment with zidovudine syrup 2mg/kg every 6 hrs for 6 wks, beginning 8-12 hrs after birth (intervention), vs. maternal and infant placebos (controls). | SBR: RR=0.33 (95% CI: 0.01-8.11)**[NS]**  [0/239 vs. 1/238 in intervention vs. control groups, respectively.] |
| 7. PACTG 316: Dorenbaum 2001 [8] | USA, Europe, Brazil and Bahamas.  RCT. Women (N=1506) receiving standard ARV therapy recruited from PACTG study sites from 1997-2000. | Assessed impact of maternal treatment with nevirapine 200mg dose orally at labour onset (with additional dose in prolonged labour) plus infant treatment with single oral dose nevirapine 2mg/kg 48-72 hrs after birth (intervention), vs. placebos (controls). All mothers received 'standard' ARV therapy as determined by clinicians, which could include any licensed ARV except NNRTI. | SBR: RR=2.99 (95% CI: 0.12-73.33).  [1/754 vs. 0/752 in intervention vs. control groups, respectively.] |
| 8. PETRA study team.  2002 [9] | South Africa, Uganda, and Tanzania.  RCT. Women (N=1797 HIV-1-infected women) enrolled from 1996-2000. | Assessed impact of maternal treatment with oral zidovudine 300mg + lamivudine 150 mg 2x/day from 36 wks gestation until labour onset, oral zidovudine 300mg + lamivudine 150 mg at onset of labour with zidovudine 300mg every 3 hrs + lamivudine every 12 hrs during labour, then zidovudine 300 mg + lamivudine 150 mg 2x/day for 7 days postpartum, plus infant treatment with zidovudine 4mg/kg + lamivudine 2mg/kg 2x/day 7 days after birth (intervention #1), vs. maternal and infant treatment with oral zidovudine as in intervention #1 except zidovudine at labour onset increased to 600 mg (intervention #2) vs. maternal treatment with zidovudine + lamivudine during labour only (zidovudine 600mg + lamivudine 50mg at labour onset, then zidovudine 300mg every 3 hrs + lamivudine 150mg every 12 hrs until delivery (intervention #3), vs. placebo (controls). | SBR:  **[**2/380 (1%) vs. 6/382 (2%) vs. 4/377 (1%) vs. 4/362 (1%) in intervention #1, intervention #2, intervention #3, and control groups, respectively.] No statistical significance data given. |
| 9. PHPT-1 Lallemant et al. 2000 [10]  . | Thailand.  RCT. 27 sites. Women (N=1437) recruited from 1997-99. | Assessed impact of maternal treatment with oral zidovudine 300mg 2x/day from 28 wks gestation, then 300mg at labour onset and every 3 hrs until delivery, plus infant treatment with zidovudine 2mg/kg orally every 6 hr for 6 wks from birth (intervention #1), vs. oral maternal and infant treatment as in intervention #1 except a shorter infant course of 3 days (intervention #2), vs. oral maternal and infant treatment as in intervention #1 except mothers initiating treatment at 35 wks gestation (intervention #3), vs. oral maternal and infant treatment as in intervention #1 except mothers initiating treatment at 35 wks gestation and infant treatment duration=3 days (intervention #4). Placebo used to ensure blinding to treatment regimen. | SBR:  *PHPT-1 a:* RR=0.55 (95% CI: 0.23-1.33)**[NS]**  8/419 vs.12/345 in intervention vs. control groups, respectively.]  *PHPT-1 b:* RR=0.33 (0.11-1.01)**[NS]**  [4/350 vs.12/345 in intervention vs. control groups, respectively.] |
| 10. PHPT-2: Lallemant et al. 2004, Cressey et al. 2005 [11] | South Africa, Tanzania, Uganda.  RCT. Women (N=1844) recruited from four large public hospitals and one missionary hospital from 1996-2000. | Assessed impact of maternal treatment with nevirapine single 200mg dose orally at labour onset plus infant treatment with single-dose oral suspension (6mg in 0.6 ml) 48-72 hours after birth (intervention #1), vs. maternal treatment as in intervention #1 but placebo for infants (intervention #2), vs. maternal and infant placebo (controls). | SBR: RR=0.25 (95% CI: 0.05-1.17)**[NS]**  [2/724 vs. 8/721 in intervention #1 vs. intervention # 2, respectively.] |
| 11. RETRO-C1: Wiktor et al. 1999 [12] | Cote d’Ivoire (Abidjan).  RCT. N=280 consenting, eligible HIV-1-seropositive pregnant women at 36 wks’ gestation. | Assessed impact of maternal oral treatment with zidovudine 300mg 2x/day from 36 wks gestation until labour onset, 300mg at onset of labour then 300mg every 3 hr until delivery (intervention), vs. placebo (controls). | SBR: RR=3.50 (95% CI: 0.74-16.55)**[NS]**  [7/140 vs. 2/140 in intervention vs. control groups, respectively.]. |
| 12. Thai-CDC : Shaffer et al. (1999) [13] | Thailand (Bangkok).  RCT. N=397 HIV-1-infected pregnant women. | Assessed impact of maternal treatment with oral zidovudine 300mg 2x/day from 36 wks until labour onset, 300 mg at onset of labour and every 3 hr until delivery (intervention), vs. placebo (controls). | SBR: RR=3.02 (95% CI: 0.12-73.57)**[NS]**  [1/198 vs. 0/199 in intervention vs. control groups, respectively.] |
| 13. Thistle et al. 2004 [14] | Zimbabwe. Rural hospital.  RCT. Women (N=222) enrolled 1999-2000. | Assessed the "Thai Regimen" of maternal treatment with zidovudine 300mg po 2x/day from 36 wks until labour onset, then 300mg po every 3 hr until delivery, with infants given placebo (intervention #1), vs. "Ultrashort Regimen" of maternal placebo from 36 wks through delivery plus infant treatment with zidovudine suspension 2mg/kg po 4x/day for 1st 3 days of life (comparison group). | SBR: 0/111 in both groups. RR not estimable. |

1. Volmink J, Siegfried NL, van der Merwe L, Brocklehurst P: **Antiretrovirals for reducing the risk of mother-to-child transmission of HIV infection**. *Cochrane Database Syst Rev* 2007(1):CD003510.

2. Bhoopat L, Khunamornpong S, Lerdsrimongkol P, Sirivatanapa P, Sethavanich S, Limtrakul A, Gomutbuthra V, Kajanavanich S, Thorner PS, Bhoopat T: **Effectiveness of short-term and long-term zidovudine prophylaxis on detection of HIV-1 subtype E in human placenta and vertical transmission**. *J Acquir Immune Defic Syndr* 2005, **40**(5):545-550.

3. Dabis F, Msellati P, Meda N, Welffens-Ekra C, You B, Manigart O, Leroy V, Simonon A, Cartoux M, Combe P *et al*: **6-month efficacy, tolerance, and acceptability of a short regimen of oral zidovudine to reduce vertical transmission of HIV in breastfed children in Cote d'Ivoire and Burkina Faso: a double-blind placebo-controlled multicentre trial. DITRAME Study Group. DIminution de la Transmission Mere-Enfant**. *Lancet* 1999, **353**(9155):786-792.

4. Guay LA, Musoke P, Fleming T, Bagenda D, Allen M, Nakabiito C, Sherman J, Bakaki P, Ducar C, Deseyve M *et al*: **Intrapartum and neonatal single-dose nevirapine compared with zidovudine for prevention of mother-to-child transmission of HIV-1 in Kampala, Uganda: HIVNET 012 randomised trial**. *Lancet* 1999, **354**(9181):795-802.

5. Kiarie JN, Kreiss JK, Richardson BA, John-Stewart GC: **Compliance with antiretroviral regimens to prevent perinatal HIV-1 transmission in Kenya**. *AIDS* 2003, **17**(1):65-71.

6. Limpongsanurak S, Thaithumyanon P, Chaithongwongwatthana S, Thisyakorn U, Ruxrungtham K, Kongsin P, Tarounotai U, Chantheptaewan N, Triratwerapong T, Ubolyam S *et al*: **Short course zidovudine maternal treatment in HIV-1 vertical transmission: randomized controlled multicenter trial**. *J Med Assoc Thai* 2001, **84 Suppl 1**:S338-345.

7. Connor EM, Sperling RS, Gelber R, Kiselev P, Scott G, O'Sullivan MJ, VanDyke R, Bey M, Shearer W, Jacobson RL *et al*: **Reduction of maternal-infant transmission of human immunodeficiency virus type 1 with zidovudine treatment. Pediatric AIDS Clinical Trials Group Protocol 076 Study Group**. *N Engl J Med* 1994, **331**(18):1173-1180.

8. Dorenbaum A: **Report of results of PACTG 316: An international phase III trial of standard antiretroviral (ARV) prophylaxis plus nevirapine (NVP) for prevention of perinatal HIV transmission**. In: *8th Conference on Retroviruses and Opportunistic Infections.* Chicago, Illinois; February 2001.

9. **Efficacy of three short-course regimens of zidovudine and lamivudine in preventing early and late transmission of HIV-1 from mother to child in Tanzania, South Africa, and Uganda (Petra study): a randomised, double-blind, placebo-controlled trial**. *Lancet* 2002, **359**(9313):1178-1186.

10. Lallemant M, Jourdain G, Le Coeur S, Kim S, Koetsawang S, Comeau AM, Phoolcharoen W, Essex M, McIntosh K, Vithayasai V: **A trial of shortened zidovudine regimens to prevent mother-to-child transmission of human immunodeficiency virus type 1. Perinatal HIV Prevention Trial (Thailand) Investigators**. *N Engl J Med* 2000, **343**(14):982-991.

11. Lallemant M, Jourdain G, Le Coeur S, Mary JY, Ngo-Giang-Huong N, Koetsawang S, Kanshana S, McIntosh K, Thaineua V: **Single-dose perinatal nevirapine plus standard zidovudine to prevent mother-to-child transmission of HIV-1 in Thailand**. *N Engl J Med* 2004, **351**(3):217-228.

12. Wiktor SZ, Ekpini E, Karon JM, Nkengasong J, Maurice C, Severin ST, Roels TH, Kouassi MK, Lackritz EM, Coulibaly IM *et al*: **Short-course oral zidovudine for prevention of mother-to-child transmission of HIV-1 in Abidjan, Cote d'Ivoire: a randomised trial**. *Lancet* 1999, **353**(9155):781-785.

13. Shaffer N, Chuachoowong R, Mock PA, Bhadrakom C, Siriwasin W, Young NL, Chotpitayasunondh T, Chearskul S, Roongpisuthipong A, Chinayon P *et al*: **Short-course zidovudine for perinatal HIV-1 transmission in Bangkok, Thailand: a randomised controlled trial. Bangkok Collaborative Perinatal HIV Transmission Study Group**. *Lancet* 1999, **353**(9155):773-780.

14. Thistle P, Gottesman M, Pilon R, Glazier RH, Arbess G, Phillips E, Wald RL, Chitsike I, Simor A, Chipato T *et al*: **A randomized control trial of an Ultra-Short zidovudine regimen in the prevention of perinatal HIV transmission in rural Zimbabwe**. *Cent Afr J Med* 2004, **50**(9-10):79-84.
