# Supplementary material for: Reducing stillbirths: prevention and management of medical disorders and infections during pregnancy
Source: BMC Pregnancy Childbirth. 2009 May 7;9(Suppl 1):S4. doi: 10.1186/1471-2393-9-S1-S4 (PMC2679410; doi:10.1186/1471-2393-9-S1-S4)
Supplement: Additional file 28 — Web Table 28. Component studies in Suksomboon et al. 2007 meta-analysis: impact of PMTCT. Component studies in Suksomboon et al. 2007 meta-analysis reporting impact on stillbirths/perinatal mortality [file 1471-2393-9-S1-S4-S28.doc]

**Web Table 28. Component studies in Suksomboon et al. 2007** **[1] meta-analysis: impact of PMTCT**

| **Source** | **Location and Type of Study** | **Intervention** | **Stillbirths / Perinatal Outcomes** |
| --- | --- | --- | --- |
| 1. Connor et al. 1994 [2] | USA, France.  RCT. 59 centers. Women (N=477) recruited from 1991-1993. | Assessed impact of maternal treatment of zidovudine 100mg orally 5x/day from time of presentation (14-34 wks) until labour onset, then IV zidovudine 2mg/kg loading dose over one hour followed by 1 mg/kg/hr until delivery, plus infant treatment with zidovudine syrup 2mg/kg every 6 hrs for 6 wks, beginning 8 - 12 hrs after birth (intervention), vs. maternal and infant placebos (controls). | SBR: RR=0.33 (95% CI: 0.01-8.11)**[NS]**  [0/239 vs. 1/238 in intervention vs. control groups, respectively.] |
| 2. Dabis et al. 1999 [3, 4] | Côte d’Ivoire (Abidjan), Burkina Faso (Bobo-Dioulasso). Public clinics.  RCT.  . | Assessed impact of maternal treatment with zidovudine 300mg twice daily from 36-38 wks’ gestation until onset of labour, then 600 mg at start of labour and 300mg twice daily until 7 days after birth (intervention), vs. no treatment (controls). | SBR: RR=0.14 (95% CI: 0.02-1.17)**[NS]**  [1/214 vs. 7/217 in intervention vs. control groups, respectively.] |
| 3. Limpongsanurak et al. 2001 [5]. | Thailand (Bangkok).  Multicenter RCT. N = 182 HIV-1 infected pregnant women who were asymptomatic and naïve to anti-retroviral agents. | Assessed impact of maternal treatment with zidovudine 250 mg orally twice daily from 38 weeks gestation until onset of labour then IV zidovudine 2mg/kg for 1st hour of labour followed by 1mg/kg/hr until delivery (intervention), vs. placebo (oral capsules, then 5% IV dextrose in saline; controls). | SBR: RR=3.07 (95% CI: 0.13-74.28)**[NS]**  [1/90 vs. 0/92 in intervention vs. control groups, respectively.] |
| 4. SAINT, Moodley et al 2003 [6] | South Africa.  RCT. HIV-infected pregnant women (N=1919) screened at 11 maternity health institutions (N = 662 in intervention group, N = 657 comparison group). | Assessed impact of open-label short course ARV regimen of either multiple-dose zidovudine and lamivudine (AZT 600 mg and lamivudine 150 mg orally, followed by AZT 300 mg every 3 h and lamivudine 150 mg every 12 h until delivery) (intervention) vs. nevirapine (200 mg administered orally in labour + additional dose 48 h later if still in labour) (comparison). | SBR: RR=4.95 (95% CI: 0.24-102.85)**[NS]**  [2/662 vs.0/655 in intervention vs. comparison groups, respectively.] |
| 5. Shaffer et al. 1999 [7] | Thailand (Bangkok).  RCT. N=397 HIV-1-infected pregnant women. | Assessed impact of maternal treatment with oral zidovudine 300mg 2x/day from 36 wks until labour onset, 300 mg at onset of labour and every 3 hr until delivery (intervention), vs. placebo (controls). | SBR: RR=3.02 (95% CI: 0.12-73.57)**[NS]**  [1/198 vs. 0/199 in intervention vs. control groups, respectively.] |
| 6. Wiktor et al. 1999 [8] | Côte d’Ivoire (Abidjan).  RCT. N=280 consenting, eligible HIV-1-seropositive pregnant women 36 wks’ gestation. | Assessed impact of maternal oral treatment with zidovudine 300mg 2x/day from 36 wks gestation until labour onset, 300mg at onset of labour then 300mg every 3 hr until delivery (intervention), vs. placebo (controls). | SBR: RR=3.50 (95% CI: 0.74-16.55)**[NS]**  [7/140 vs. 2/140 in intervention vs. control groups, respectively.]. |

References

1. Suksomboon N, Poolsup N, Ket-Aim S: **Systematic review of the efficacy of antiretroviral therapies for reducing the risk of mother-to-child transmission of HIV infection**. *J Clin Pharm Ther* 2007, **32**(3):293-311.

2. Connor EM, Sperling RS, Gelber R, Kiselev P, Scott G, O'Sullivan MJ, VanDyke R, Bey M, Shearer W, Jacobson RL *et al*: **Reduction of maternal-infant transmission of human immunodeficiency virus type 1 with zidovudine treatment. Pediatric AIDS Clinical Trials Group Protocol 076 Study Group**. *N Engl J Med* 1994, **331**(18):1173-1180.

3. Dabis F, Elenga N, Meda N, Leroy V, Viho I, Manigart O, Dequae-Merchadou L, Msellati P, Sombie I: **18-Month mortality and perinatal exposure to zidovudine in West Africa**. *AIDS* 2001, **15**(6):771-779.

4. Dabis F, Msellati P, Meda N, Welffens-Ekra C, You B, Manigart O, Leroy V, Simonon A, Cartoux M, Combe P *et al*: **6-month efficacy, tolerance, and acceptability of a short regimen of oral zidovudine to reduce vertical transmission of HIV in breastfed children in Cote d'Ivoire and Burkina Faso: a double-blind placebo-controlled multicentre trial. DITRAME Study Group. DIminution de la Transmission Mere-Enfant**. *Lancet* 1999, **353**(9155):786-792.

5. Limpongsanurak S, Thaithumyanon P, Chaithongwongwatthana S, Thisyakorn U, Ruxrungtham K, Kongsin P, Tarounotai U, Chantheptaewan N, Triratwerapong T, Ubolyam S *et al*: **Short course zidovudine maternal treatment in HIV-1 vertical transmission: randomized controlled multicenter trial**. *J Med Assoc Thai* 2001, **84 Suppl 1**:S338-345.

6. Moodley D, Moodley J, Coovadia H, Gray G, McIntyre J, Hofmyer J, Nikodem C, Hall D, Gigliotti M, Robinson P *et al*: **A multicenter randomized controlled trial of nevirapine versus a combination of zidovudine and lamivudine to reduce intrapartum and early postpartum mother-to-child transmission of human immunodeficiency virus type 1**. *J Infect Dis* 2003, **187**(5):725-735.

7. Shaffer N, Chuachoowong R, Mock PA, Bhadrakom C, Siriwasin W, Young NL, Chotpitayasunondh T, Chearskul S, Roongpisuthipong A, Chinayon P *et al*: **Short-course zidovudine for perinatal HIV-1 transmission in Bangkok, Thailand: a randomised controlled trial. Bangkok Collaborative Perinatal HIV Transmission Study Group**. *Lancet* 1999, **353**(9155):773-780.

8. Wiktor SZ, Ekpini E, Karon JM, Nkengasong J, Maurice C, Severin ST, Roels TH, Kouassi MK, Lackritz EM, Coulibaly IM *et al*: **Short-course oral zidovudine for prevention of mother-to-child transmission of HIV-1 in Abidjan, Cote d'Ivoire: a randomised trial**. *Lancet* 1999, **353**(9155):781-785.
