# Supplementary material for: Reducing stillbirths: prevention and management of medical disorders and infections during pregnancy
Source: BMC Pregnancy Childbirth. 2009 May 7;9(Suppl 1):S4. doi: 10.1186/1471-2393-9-S1-S4 (PMC2679410; doi:10.1186/1471-2393-9-S1-S4)
Supplement: Additional file 29 — Web Table 29. Component studies in Wiysonge et al. 2005 meta-analysis: impact of PMTCT. Component studies in Wiysonge et al. 2005 meta-analysis reporting impact on stillbirths/perinatal mortality [file 1471-2393-9-S1-S4-S29.doc]

**Web Table 29. Component studies in Wiysonge et al. 2005** **[1] meta-analysis: impact of PMTCT**

| **Source** | **Location and Type of Study** | **Intervention** | **Stillbirths / Perinatal Outcomes** |
| --- | --- | --- | --- |
| 1. Coutsoudis et al, 1999 [2] | South Africa (KwaZulu-Natal).  RCT. HIV-infected women (N=728) enrolled at 17-39 wks' gestation; 30.6% of whom had serum retinol levels <20 µg/dl. | Assessed impact of maternal treatment with daily oral vitamin A (5000 IU retinyl palmitate and 30 mg beta-carotene) plus 200,000 IU at delivery (intervention) or placebo (controls). | SBR: OR=1.46 (95% CI: 0.41-5.22) **[NS]**  [6/341 vs. 4/330 in intervention vs. control groups, respectively.] |
| 2. Fawzi et al, 2002 [3] | Tanzania (Dar es Salaam).  RCT. HIV-infected pregnant women (N=1075) enrolled at 12-27 wks' gestation. | Assessed impact of daily oral dose of one of: vitamin A (30mg beta carotene + 5000 IU retinyl palmitate) alone (intervention #1), multivitamins (20mg B1, 20mg B2, 25mg B6, 100mg niacin, 50microg B12, 500mg C, 30 mg E, and 0.8 mg folic) plus vitamin A (intervention #2), multivitamins without vitamin A (intervention #3), or placebo (controls). At delivery, women receiving any vitamin A were given an additional 200,000 IU oral dose of vitamin A while the others received an extra dose of placebo. | SBR: OR=0.86 (95% CI: 0.54-1.36)**[NS]**  [36/521 vs. 41/514 in intervention (all vitamin A groups) vs. control groups, respectively.] |
| 3. Friis et al, 2004 [4] | Zimbabwe (Harare).  RCT. HIV-infected pregnant women (N=533) enrolled at 22-35 wks' gestation. | Assessed impact of maternal treatment with daily oral tablet containing vitamin A (3000 micrograms retinol equivalents and 3.5 mg beta-carotene) and the recommended daily allowance of 11 micronutrients (1.5mg thiamine, 1.6mg riboflavin, 2.2mg B-6, 4.0 mcg B12, 17mg niacin, 80mg C, 10 mcg D, 10mg E, 15mg Zn 1.2 mcg Cu 65 mcg Se) (intervention) vs. placebo (controls). | SBR: OR=1.39 (95% CI: 0.23-8.41)**[NS]**  [3/273 vs. 2/253 in intervention vs. control groups, respectively.] |
| 4. Kumwenda et al. 2002 [5] | Malawi (Blantyre).  RCT. HIV-infected pregnant women (N=697) 18-28 wks’ gestation. Prevalence of vitamin A deficiency (<0.70 µmol/L) was 51% during the 2nd trimester. | Assessed impact of daily doses of orally administered vitamin A (10,000 IU) plus iron (30mg of elemental iron) and folate (400 µg) from enrollment until delivery (intervention), vs. iron-folate only (controls). | SBR: OR=1.39 (95% CI: 0.48-4.06)**[NS]**  [8/306 vs. 6/317 in intervention vs. control groups, respectively.] |

References

1. Wiysonge CS, Shey MS, Sterne JA, Brocklehurst P: **Vitamin A supplementation for reducing the risk of mother-to-child transmission of HIV infection**. *Cochrane Database Syst Rev* 2005(4):CD003648.

2. Coutsoudis A, Pillay K, Spooner E, Kuhn L, Coovadia HM: **Randomized trial testing the effect of vitamin A supplementation on pregnancy outcomes and early mother-to-child HIV-1 transmission in Durban, South Africa. South African Vitamin A Study Group**. *AIDS* 1999, **13**(12):1517-1524.

3. Fawzi WW, Msamanga GI, Hunter D, et al: **Randomized trial of vitamin supplements in relation to transmission of HIV-1 through breastfeeding and early child mortality**. *AIDS* 2002, **16**:1935-1944.

4. Friis H, Gomo E, Nyasema N, et al: **Effect of multinutrient supplementation on gestational length and birth size: a randomized, placebo-controlled, double-blind effectiveness trial in Zimbabwe**. *Am J Clin Nutr* 2004, **80**:178-184.

5. Kumwenda N, Miotti PG, Taha TE, et al: **Antenatal vitamin A supplementation increases birth weight and decreases anemia among infants born to human immunodeficiency virus-infected women in Malawi.** . *Clin Infect Dis* 2002, **35**:618-624. .
