# Supplementary material for: Reducing stillbirths: prevention and management of medical disorders and infections during pregnancy
Source: BMC Pregnancy Childbirth. 2009 May 7;9(Suppl 1):S4. doi: 10.1186/1471-2393-9-S1-S4 (PMC2679410; doi:10.1186/1471-2393-9-S1-S4)
Supplement: Additional file 30 — Web Table 30. Component studies in Xiong et al. 2006: impact of periodontal disease. Component studies in Xiong et al. 2006 reporting impact on stillbirths/perinatal mortality [file 1471-2393-9-S1-S4-S30.doc]

**Web Table 30. Component studies in Xiong et al. 2006 [1]: impact of periodontal disease**

| **Source** | **Location and Type of Study** | **Intervention** | **Stillbirths / Perinatal Outcomes** |
| --- | --- | --- | --- |
| 1. Moore 2004 [2] | UK.  Cohort study. Pregnant women (N=3738). | Assessed women with and without periodontal disease for associations between disease status and adverse pregnancy outcome. | Fetal death (Miscarriage+SB): adj OR=2.54 (95% CI: 1.20–5.39) |

References

1. Xiong X, Buekens P, Fraser WD, Beck J, Offenbacher S: **Periodontal disease and adverse pregnancy outcomes: a systematic review**. *BJOG* 2006, **113**(2):135-143.

2. Moore S, Ide M, Coward PY, Randhawa M, Borkowska E, Baylis R, Wilson RF: **A prospective study to investigate the relationship between periodontal disease and adverse pregnancy outcome**. *Br Dent J* 2004, **197**(5):251-258; discussion 247.
