# Supplementary material for: Reducing stillbirths: prevention and management of medical disorders and infections during pregnancy
Source: BMC Pregnancy Childbirth. 2009 May 7;9(Suppl 1):S4. doi: 10.1186/1471-2393-9-S1-S4 (PMC2679410; doi:10.1186/1471-2393-9-S1-S4)
Supplement: Additional file 31 — Web Table 31. Component studies in Xiong et al. 2007: impact of periodontal disease. Component studies in Xiong et al. 2007 reporting impact on stillbirths/perinatal mortality [file 1471-2393-9-S1-S4-S31.doc]

**Web Table 31. Component studies in Xiong et al. 2007 [1]: impact of periodontal disease**

| **Source** | **Location and Type of Study** | **Intervention** | **Stillbirths / Perinatal Outcomes** | |
| --- | --- | --- | --- | --- |
| 1. Farrell et al. 2006 [2] | UK.  Prospective cohort study. Pregnant women (N=1793) who reported never previously smoking, recruited at 12 wks gestation. 7.3% had a pre-term birth and 0.9% a stillbirth. | Survey instrument plus periodontal examination performed to investigate possible associations between periodontal measures of disease and adverse pregnancy outcomes. | Pre-term: No association. **[NS]**  LBW: No association **[NS]**  SB: Higher mean probing depth at mesial sites among subjects with stillbirth vs. subjects with live birth at term (2.69 mm vs. 2.41 mm, P=0.006).  Higher mean probing depth for all sites in subjects who experienced a stillbirth (2.15mm vs. 2.02 mm, P=0.054)**[NS]**. | |
| 2. Moore 2004 [3] | UK.  Cohort study. Pregnant women (N=3738). | Assessed women with and without periodontal disease for associations between disease status and adverse pregnancy outcome. | | Fetal death (Miscarriage+SB): adj OR=2.54 (95% CI: 1.20–5.39) |

References

1. Xiong X, Buekens P, Vastardis S, Yu SM: **Periodontal disease and pregnancy outcomes: state-of-the-science**. *Obstet Gynecol Surv* 2007, **62**(9):605-615.

2. Farrell S, Ide M, Wilson RF: **The relationship between maternal periodontitis, adverse pregnancy outcome and miscarriage in never smokers**. *J Clin Periodontol* 2006, **33**(2):115-120.

3. Moore S, Ide M, Coward PY, Randhawa M, Borkowska E, Baylis R, Wilson RF: **A prospective study to investigate the relationship between periodontal disease and adverse pregnancy outcome**. *Br Dent J* 2004, **197**(5):251-258; discussion 247.
