# Supplementary material for: Reducing stillbirths: prevention and management of medical disorders and infections during pregnancy
Source: BMC Pregnancy Childbirth. 2009 May 7;9(Suppl 1):S4. doi: 10.1186/1471-2393-9-S1-S4 (PMC2679410; doi:10.1186/1471-2393-9-S1-S4)
Supplement: Additional file 12 — Web Table 12. Component studes in Di Nisio et al. 2005: Impact of LMWH (enoxaparin) versus aspirin. Component studies in Di Nisio et al. 2005 review reporting impact on stillbirths/perinatal mortality [file 1471-2393-9-S1-S4-S12.doc]

**Web Table 12. Component studies in Di Nisio et al. 2005 [1]: Impact of anticoagulant therapies**

| **Source** | **Location and Type of Study** | **Intervention** | **Stillbirths / Perinatal Outcomes** |
| --- | --- | --- | --- |
| 1. Gris J-C et al. 2004 [2] | France.  Quasi-RCT. N=160 women with history of 1 fetal loss > 10 wks gestation and factor V Leiden, FII 20210A mutation, or protein S deficiency. | Compared the impact of subcutaneous LMWH (enoxaparin 40 mg/daily); intervention) vs. aspirin (100 mg/daily) (controls) from 8th wk of amenorrhoea after positive pregnancy test until delivery. | Live birth rate: RR=10.00 (95% CI: 1.56-64.20).  [10/10 vs. 1/10 in intervention vs. control groups, respectively]. |
| 2. Tulppala et al. 1997, 1997 [3, 4]. | Finland.  RCT. Women (N=82) with a history of ≥3 consecutive miscarriages in whom no obvious cause for their previous pregnancy losses was found. | Compared the impact of aspirin (50 mg/daily) vs. placebo, initiated immediately after positive urinary pregnancy test. | Live-birth rate: RR=1.00 (95% CI: 0.78-1.29)**[NS]**  [22/27 in both groups.] |

# References

1.	Di Nisio M, Peters L, Middeldorp S: **Anticoagulants for the treatment of recurrent pregnancy loss in women without antiphospholipid syndrome**. *Cochrane Database Syst Rev* 2005(2):CD004734.

2.	Gris JC, Mercier E, Quere I, Lavigne-Lissalde G, Cochery-Nouvellon E, Hoffet M, Ripart-Neveu S, Tailland ML, Dauzat M, Mares P: **Low-molecular-weight heparin versus low-dose aspirin in women with one fetal loss and a constitutional thrombophilic disorder**. *Blood* 2004, **103**(10):3695-3699.

3.	Tulppala M, Marttunen M, Soderstrom-Anttila V, Ailus K, Palosuo T, Ylikorkala O: **Low dose aspirin in the prevention of miscarriage in women with unexplained or autoimmune related recurrent miscarriage: effect on prostacyclin and thromboxane A2 production**. *Human Reproduction;* 1997, **12**:191.

4.	Tulppala M, Marttunen M, Soderstrom-Anttila V, Foudila T, Ailus K, Palosuo T, Ylikorkala O: **Low-dose aspirin in prevention of miscarriage in women with unexplained or autoimmune related recurrent miscarriage: effect on prostacyclin and thromboxane A2 production**. *Hum Reprod* 1997, **12**(7):1567-1572.
